# Synthesis of Atropisomeric Hydrazides by One‐Pot Sequential Enantio‐ and Diastereoselective Catalysis

**DOI:** 10.1002/anie.202209895

**Published:** 2022-09-12

**Authors:** Chiara Portolani, Giovanni Centonze, Sara Luciani, Andrea Pellegrini, Paolo Righi, Andrea Mazzanti, Alessia Ciogli, Andrea Sorato, Giorgio Bencivenni

**Affiliations:** ^1^ Department of Industrial Chemistry “Toso Montanari” Alma Mater Studiorum-University of Bologna viale del Risorgimento 4 40136 Bologna Italy; ^2^ Center for Chemical Catalysis, C3 Alma Mater Studiorum-University of Bologna viale del Risorgimento 4 40136 Bologna Italy; ^3^ Department of Chemistry and technologies of drug Sapienza University of Rome piazzale A. Moro 5 00185 Rome Italy

**Keywords:** Diastereoselectivity, Enantioselectivity, N−N Atropisomers, Organocatalysis, Phase-Transfer Catalysis

## Abstract

The first catalytic enantioselective and diastereoselective synthesis of atropisomeric hydrazides was achieved using a sequential catalysis protocol. This strategy is based on a one‐pot sequence of two organocatalytic cycles featuring the enamine amination of branched aldehydes followed by nitrogen alkylation under phase‐transfer conditions. The resulting axially chiral hydrazides were obtained directly from commercially available reagents in high yields and with good stereocontrol. The permutation of organocatalysts allowed easy access to all stereoisomers, enabling a stereodivergent approach to enantioenriched atropisomeric hydrazides.

## Graphical abstract

The first enantioselective and diastereoselective synthesis of atropisomeric hydrazides was performed using one‐pot sequential catalysis based on the enamine amination of branched aldehydes followed by phase‐transfer N‐alkylation. Chiral hydrazides were obtained with high yields and stereocontrol using commercially available reagents and catalysts. The permutation of catalysts allowed for a stereodivergent approach to a new class of atropisomers.

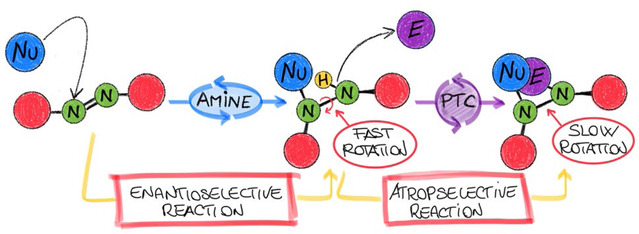

Chiral molecules with stereogenic axes are present in natural bioactive products and functional materials.^[1]^ In recent years, we have observed the rapid development of atroposelective catalysis, a new branch of asymmetric synthesis that has become a powerful tool for the construction of an important class of rotationally constrained compounds.^[2]^ However, currently available catalytic methods are restricted to C−C and C−X atropisomers (X=N, S, O), thus limiting the pool of achievable atropisomers. Atropisomers featuring hindered rotation around the N−N single‐bond have been known for a long time. However, despite their ever‐increasing importance as bioactive molecules, materials, and chiral ligands^[3]^ (Figure 1a), direct enantioselective synthesis was not explored until recently, when several preparations of N−N atropisomers were disclosed in rapid succession (Figure 1b). Liu and Lu synthesized 1,1′‐bipyrrole atropisomers through dissymmetric Friedel–Crafts alkylation using a Cu‐bisoxazoline‐catalyst.^[4]^ The groups of Lu and Houk reported access to 1‐aminopyrroles and 3‐aminoquinazolinones by Brønsted base catalysis.^[5]^ Li realized the *N*‐acylation and *N*‐alkylation reactions of quinazolinone‐type benzamides.^[6]^ The group of Zhang and Shi realized a chiral phosphoric acid‐catalyzed synthesis of N−N axially chiral indoles and pyrroles.^[7]^ Zhao and Yang realized a similar approach, reporting an interesting example of an enantiodivergent Paal–Knorr reaction.^[8]^ Recently, Rinaldi isolated a stable atropisomeric hydrazide as an intermediate for the preparation of isosteres of amino acids and conformationally restricted γ‐lactams.^[9]^

**Figure 1:**
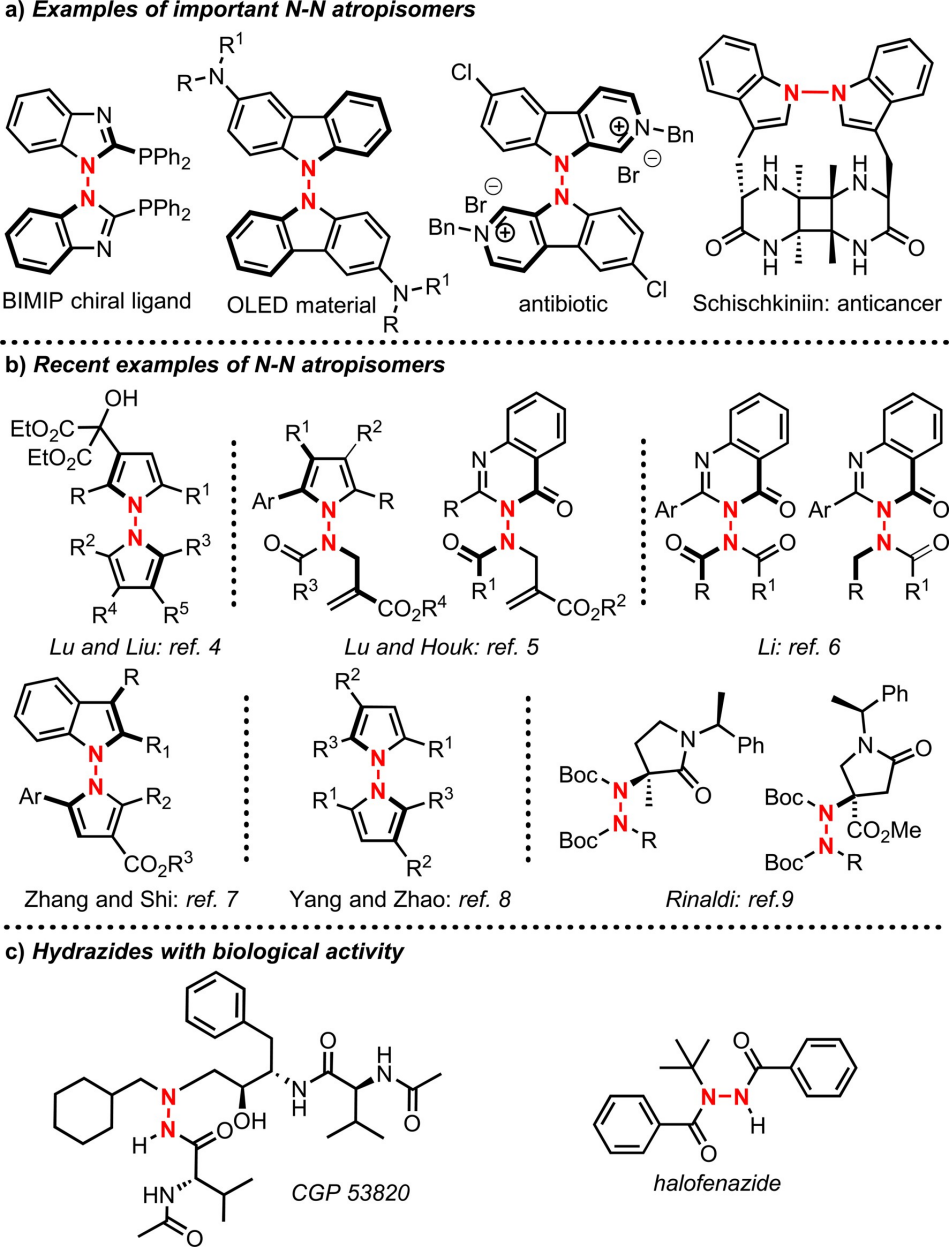
[a] Examples of important N−N atropisomers. [b] Recent examples of N−N atropisomers. [c] Examples of bioactive hydrazides.

In the search for novel processes for the enantioselective preparation of new classes of atropisomers,^[10]^ we focused our attention on the first organocatalytic synthesis of axially chiral hydrazides. Hydrazides are a class of compounds that have received attention particularly for their properties as bioactive products, such as aza‐peptide analog CGP 53820, which is a potent inhibitor of HIV‐1 and HIV‐2 protease, and as environmentally safe insecticides, such as halofenozide and its derivatives (Figure 1c).^[11]^ We investigated whether atropisomeric hydrazides can be prepared using an organic catalyst that controls the stereogenic axis configuration under mild reaction conditions. Ideally, such a transformation should be realized using reagents with the appropriate bulkiness and electronic properties to guarantee high rotational stability of the N−N single bond through the destabilization of the rotation transition state (TS) (Figure 2a). This presents a challenging goal for the synthesis of N−N atropisomers, whose configuration stability has been demonstrated to be dependent on the size of the substituents, electronic repulsion of the substituents’ unpaired electrons (if any), and the sp^2^ hybridization of the nitrogen atoms.^[3]^

**Figure 2:**
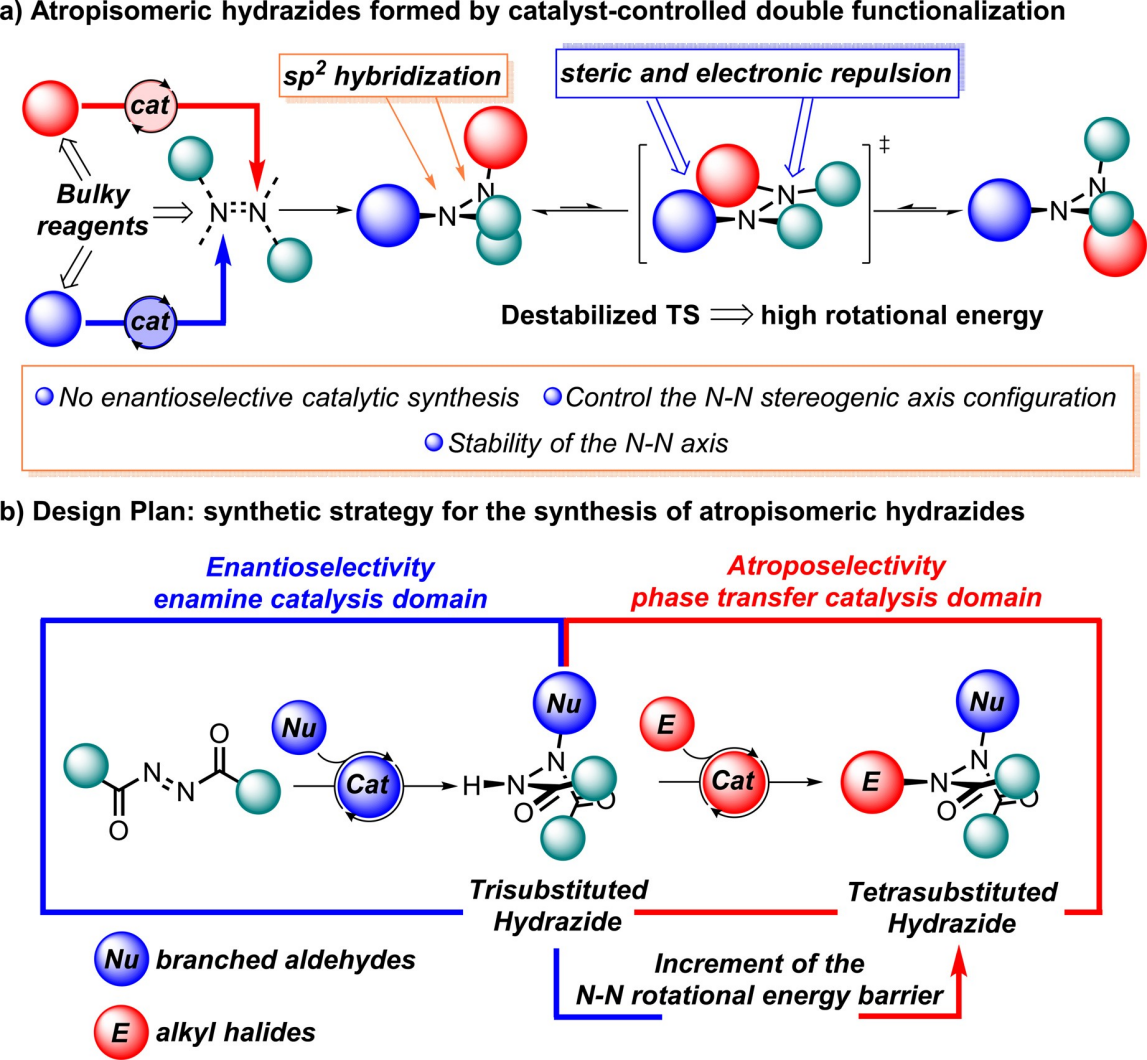
[a] Atropisomeric hydrazides formed by catalyst‐controlled double functionalization. [b] Design strategy for the synthesis of atropisomeric hydrazides.

A rational catalytic strategy involves using azodicarboxylates as precursors for the N−N single bond (Figure 2b). We reasoned that they could act as reagents in a process comprising two distinct catalytic steps that sequentially increase the steric hindrance around the N−N single bond, leading to a rotationally stable chiral tetrasubstituted hydrazides. In the first step, azodicarboxylates undergo a nucleophilic addition reaction to give trisubstituted hydrazides. In the second step, the alkylation of the second nitrogen atom affords a rotationally hindered hydrazide. We also envisioned the possibility of a one‐pot sequential catalytic process.^[12]^ Herein, we report the first enantioselective and diastereoselective synthesis of atropisomeric hydrazides by combining two stereoselective steps: 1) enamine‐catalyzed α‐amination of branched aldehydes, followed by 2) nitrogen alkylation under phase transfer (PT) conditions. The multicomponent one‐pot sequence enables catalyst‐controlled stereodivergent^[13]^ access using commercially available starting materials to all four N−N atropisomers with high stereocontrol of two distinct elements of chirality.

Our investigation began with the preparation of the trisubstituted hydrazide **3** **a** through the amination reaction of racemic 2‐phenylpropanal (**1** **a**) with di‐*tert*‐butyl azodicarboxylate (DTBA) (**2** **a**) in chloroform (CHCl_3_) using 9‐*epi*‐9‐amino‐9‐deoxy‐quinine (**A**) as the catalyst and TFA as the co‐catalyst.^[14]^ The hydrazide **3** **a** was isolated with a 90 % yield and 94 % enantiomeric excess (ee) and was used for alkylation optimization. A thorough screening of PT catalysts, of which cinchona alkaloid derivatives **B**–**N** were the most effective, was performed using benzyl bromide **4** **a** as the alkylating agent^[15]^ (Figure 3). *N*‐Benzyl‐quininium chloride (**D**) produced the atropisomeric hydrazide with a 60 % yield and 99 % ee as a 1 : 6.3 mixture of diastereoisomers **5** **a** and **5** **a′**. We then explored the feasibility of the one‐pot process. We performed the first step in CHCl_3_ and then switched the solvent to toluene for the alkylation step. Under these conditions, hydrazide was isolated with a 65 % yield, 1 : 6 diastereomeric ratio (d.r.), and 99 % ee (Figure 3, entry 1). We then attempted to unify the two reactions by using the same solvent to make the entire process more manageable and practical. Using CHCl_3_, the hydrazide was isolated with a 35 % yield, 1 : 1.2 d.r., and 96 % ee (Figure 3, entry 2). Surprisingly, hydrazide was isolated with a 60 % yield at 1 : 6.5 d.r. and 97 % ee in toluene (Figure 3, entry 3). We then further improved the first step of the reaction by diluting the reaction mixture to 0.1 M.^[15]^ Under these new conditions, hydrazide was isolated with a 65 % yield at 1 : 6.5 d.r. and 99 % ee (Figure 3, entry 4). The final optimization was performed by changing the counter anion of the catalyst: using *N*‐benzyl‐quinidinium bromide **N** increased the yield to 70 %, and the d.r. switched to 9 : 1 in favor of **5** **a** as expected (Figure 3, entry 5). Finally, by setting the temperature of the alkylation step to −5 °C, hydrazide could be obtained in 73 % yield, >99 % ee, and 12 : 1 d.r., with **5** **a** as the major diastereoisomer (Figure 3, entry 6).

**Figure 3:**
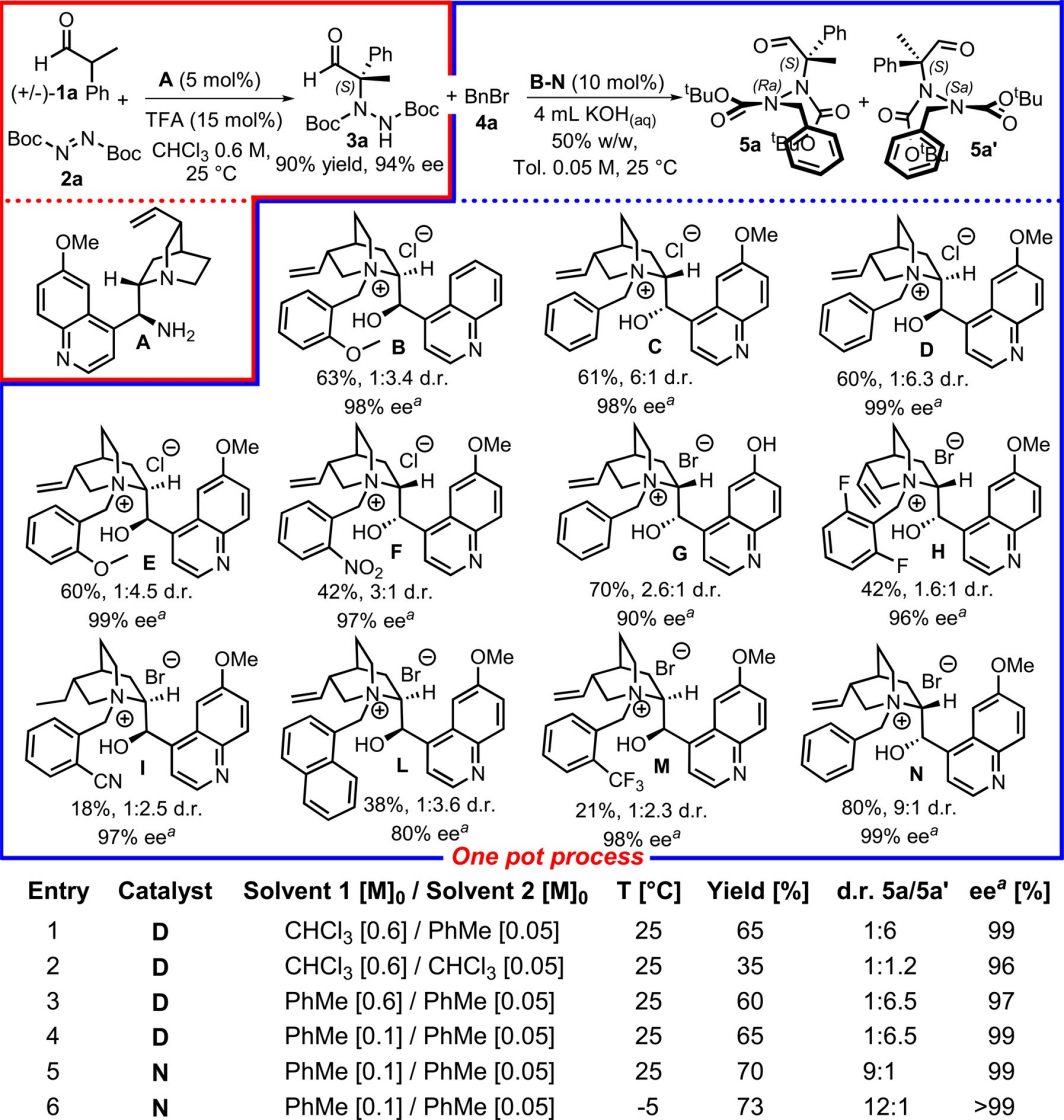
Optimization of the atroposelective synthesis of hydrazides. The amination reaction was performed on a 1.2 mmol scale. The alkylation was performed on a 0.1 mmol scale using 4 mL of a 50 % w/w KOH aqueous solution and 2 mL of toluene at room temperature. The yield refers to the sum of diastereoisomers. The ee % and d.r. were determined by HPLC analysis on a chiral stationary phase. [a] ee % of major diastereoisomer.

We then explored the scope of the atroposelective synthesis of hydrazides by varying the substituents on the aldehyde (Figure 4).This methodology applies to a large series of branched aldehydes bearing aryl substituents of different natures. In particular, good reactivity was observed when aromatic groups with electron‐rich alkyl (**5** **b**–**d**), aryl (**5** **e**–**f**), and oxoalkyl (**5** **g**–**h**) groups were employed. In addition, high values of yield, enantioselectivity, and diastereocontrol were obtained in most cases. In some of these cases, it was necessary to conduct the alkylation step at room temperature to improve the reaction rate and isolate the product with a better yield, albeit with a lower d.r. (**5** **b** and **5** **e**). The use of an aldehyde with a heteroaromatic substituent gave a poor yield (**5** **i**), and the same trend was observed when the methyl group was replaced with an ethyl group. In this last case, to push the alkylation to a decent yield, an excess of **4** **a** was used (**5** **j**). When *p*‐Cl‐, *o*‐Br‐, or *o*‐NO_2_‐ substituted aromatic rings were used along with branched aldehydes with two alkyl groups, the hydrazide intermediate unexpectedly decomposed during the alkylation step. We attempted the enantioselective synthesis of tetrasubstituted hydrazides by using isobutyraldehyde **1** **k** in the first step of the process. The resulting atropisomer **5** **k** was obtained with a 40 % yield and 54 % ee, indicating the possibility of broadening the scope of the process to other chiral hydrazides. The methodology was easily scalable to 3.0 mmol, albeit with lower diastereoselectivity. The process was explored using different benzylic electrophiles (Figure 5).

**Figure 4:**
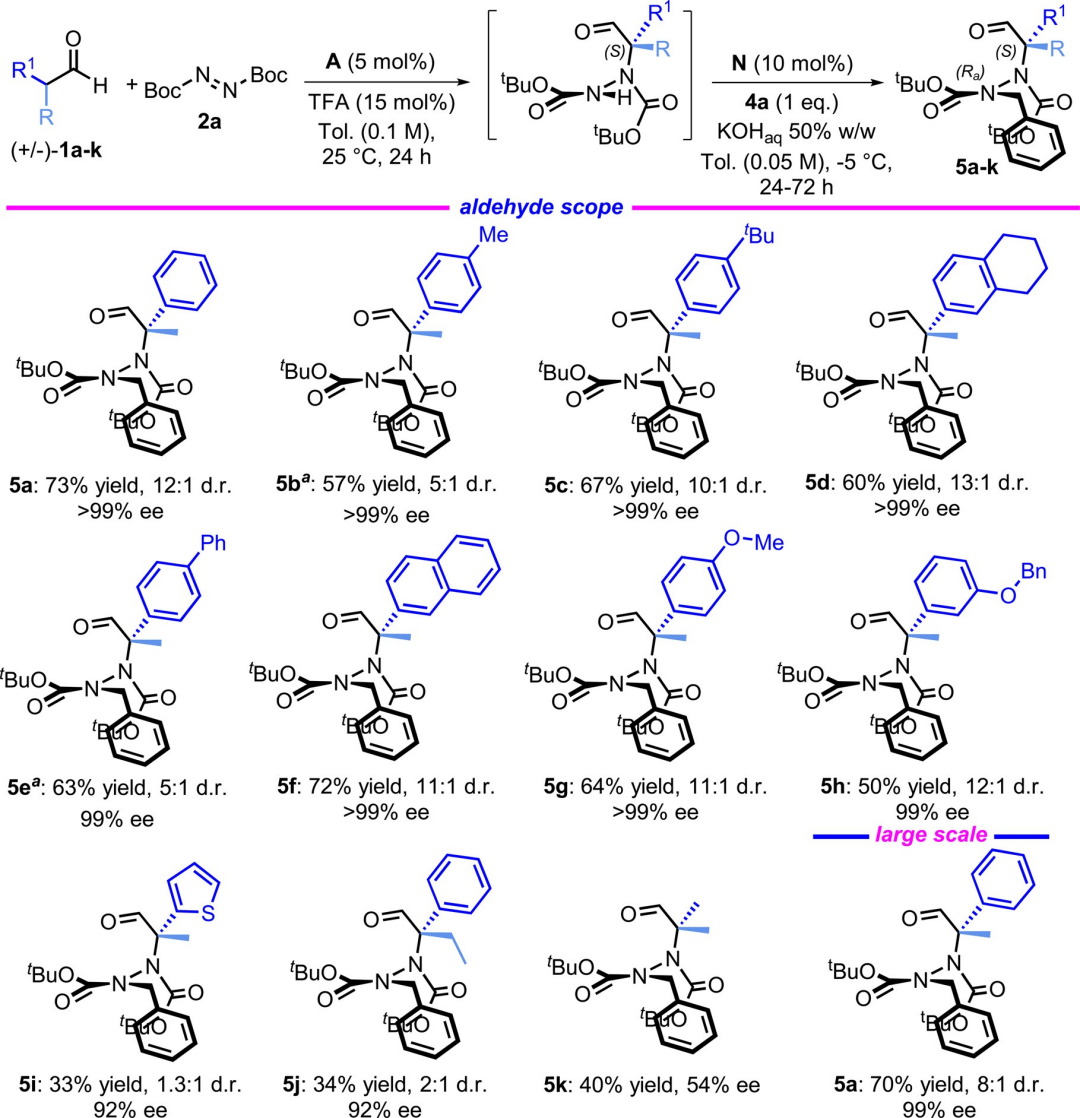
Scope of the atroposelective synthesis of hydrazides. The amination reaction was performed using **1** **a**–**k** (0.3 mmol) and **2** **a** (0.33 mmol) in 3 mL of toluene. The alkylation reaction was performed using **4** **a** (0.3 mmol) in 3 mL of toluene. The yield refers to the sum of diastereoisomers. Only the major diastereoisomer is reported. The ee % and d.r. were determined by HPLC analysis on a chiral stationary phase. [a] Reaction performed at 25 °C. When the reaction was performed at −5 °C, **5** **b** was obtained with a 17 % yield, >99 % ee and 12 : 1 d.r., and **5** **e** was obtained with a 35 % yield, >99 % ee and 8 : 1 d.r.

**Figure 5:**
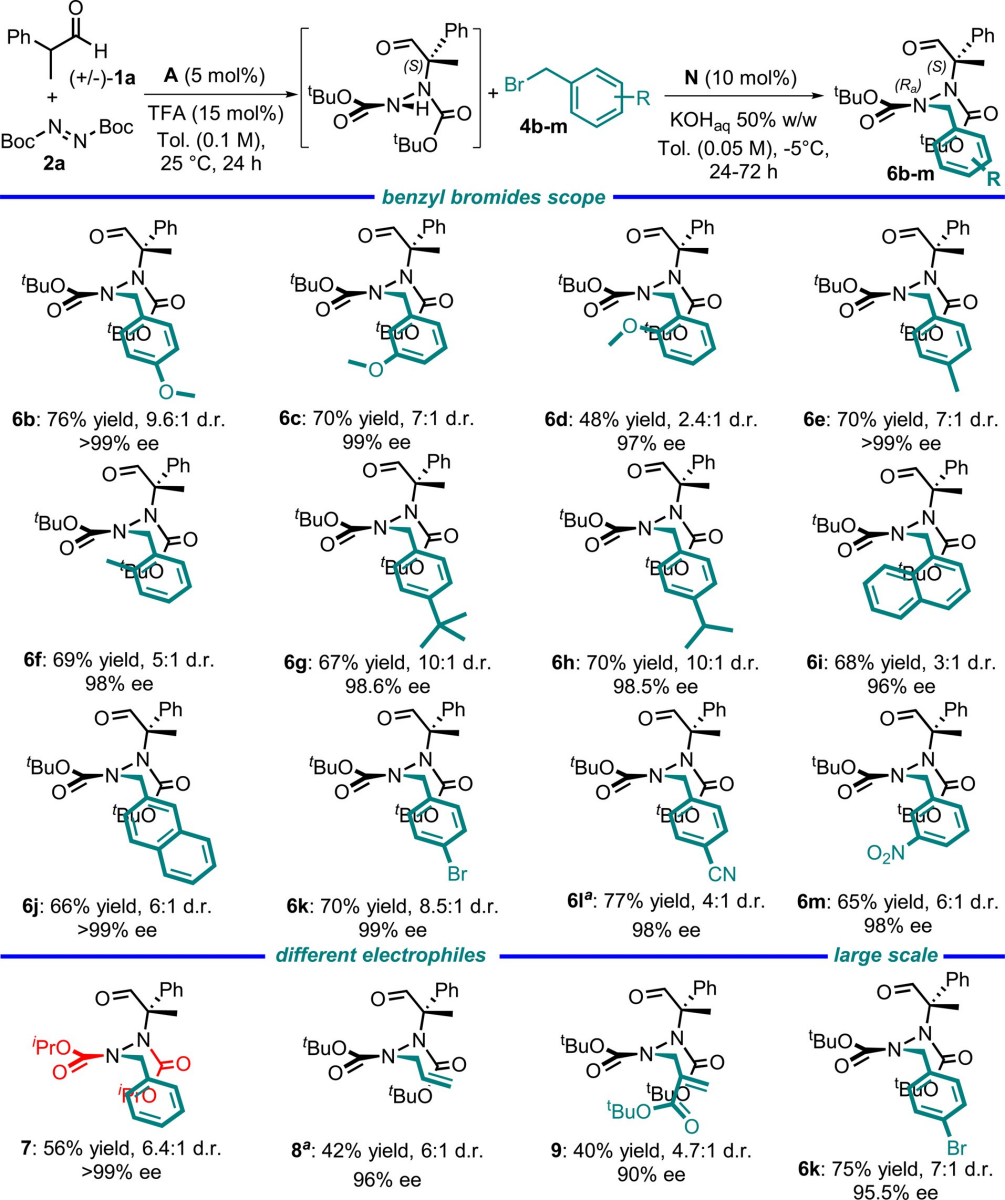
Scope of the atroposelective synthesis of hydrazides. The amination reaction was performed using **1** **a** (0.3 mmol) and **2** **a** (0.33 mmol) in 3 mL of toluene. The alkylation reaction was performed using **4** **b**–**m** (0.3 mmol) in 3 mL of toluene. The yield refers to the sum of diastereoisomers. Only the major diastereoisomer is reported. The ee % and d.r. were determined by HPLC analysis on a chiral stationary phase. [a] Reaction performed at 25 °C. When the reaction was performed at −5 °C, **6** **l** was obtained with a 42 % yield, 99 % ee, and 6.5 : 1 d.r., and **8** was obtained with a 20 % yield, >99 % ee, and 15 : 1 d.r.

Hydrazides **6** **b**–**m** were obtained with high yields and excellent enantioselectivities. Electron‐donating methoxy groups were well‐tolerated as the *ortho*, *meta*, and *para* substituents on the benzylic aromatic ring (**6** **b**–**d**). In addition, the presence of alkyl groups of different sizes resulted in good yields and high stereocontrol (**6** **e**–**h**). Interestingly, 1‐ and 2‐(bromomethyl) naphthalenes as well as benzylbromides with electron‐withdrawing substituents gave good results (**6** **i**–**m**). To expand the general scope of the reaction, we explored the reactivities of different electrophiles: diisopropylazodicarboxylate could be employed as an alternative to DTBA (**7**), and allyl iodide (**8**) and Morita–Baylis–Hillman carbonate (**9**) were interesting partners for the alkylation reaction. As observed when the aldehyde scope was studied, hydrazides **6** **d**, **6** **l**, and **8** were obtained by conducting the alkylation at room temperature. In this case, the entire process can be scaled up to 2.5 mmol giving the corresponding hydrazide **6** **k** with good yield and enantioselectivity.

The development of strategies that provide access to the entire set of stereoisomers of a compound with multiple elements of chirality is a challenging task; one‐pot hydrazide synthesis by sequential relay catalysis lends itself excellently to this strategy. As shown in Figure 6, access to all four stereoisomers can be achieved by catalyst permutation, resulting in good yields and enantioselectivities with variable diastereoisomeric ratios (Figure 6).

**Figure 6:**
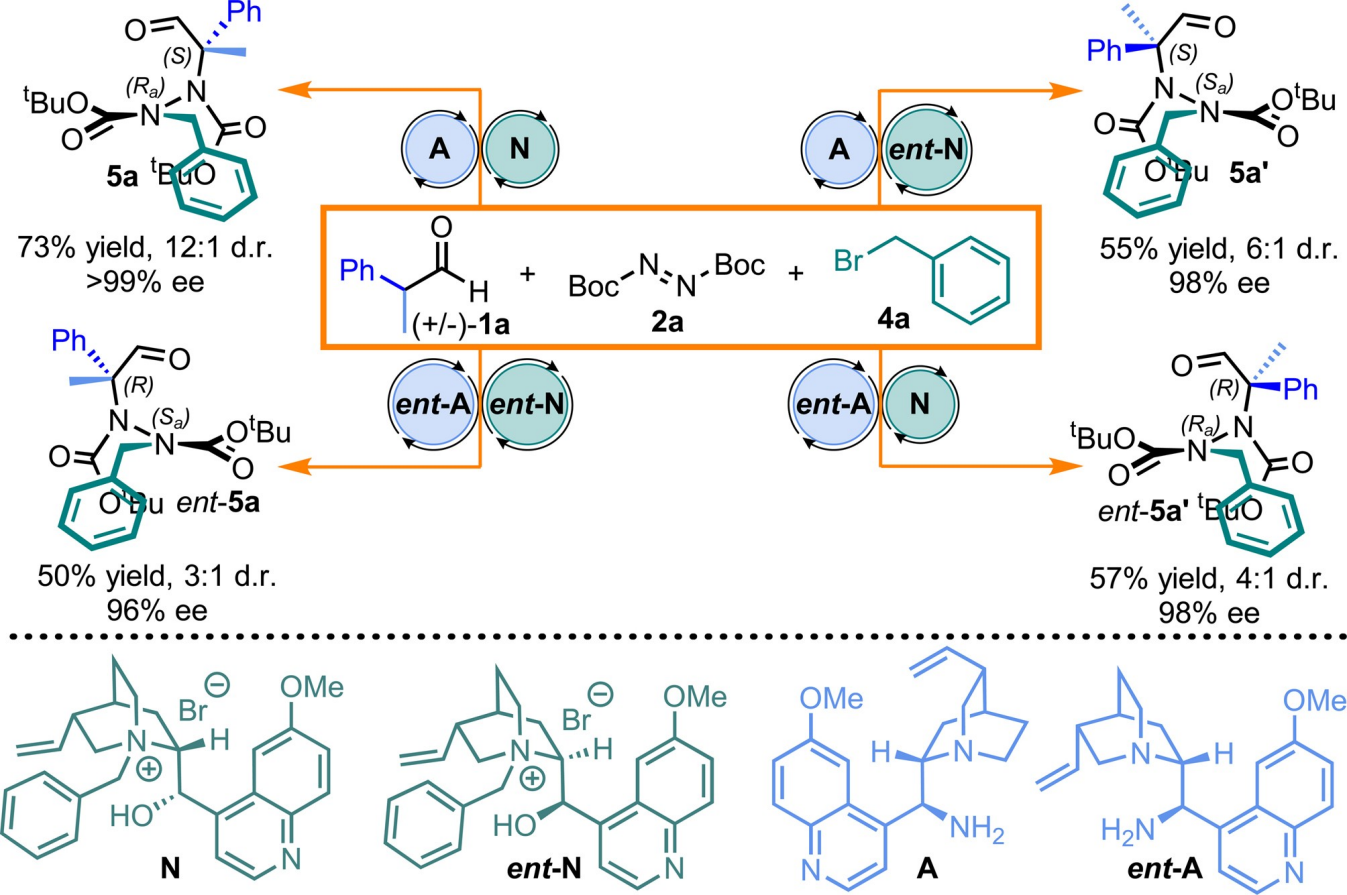
Stereodivergent synthesis of hydrazides by catalyst permutation.

We then explored the derivatization of atropisomeric hydrazides by reducing and oxidizing a 12 : 1 mixture of **5** **a** and **5** **a′** to obtain the corresponding alcohol **5** **ar** with a 90 % yield and >99 % ee, and acid **5** **ao** as a single diastereoisomer with a 56 % yield and >99 % ee. In line with the scope of our work, we attempted derivatization directly from the crude mixture, realizing a three‐step reaction process that gave the alcohol **10** **ekr** with high yield and good stereoselectivity (Figure 7).

**Figure 7:**
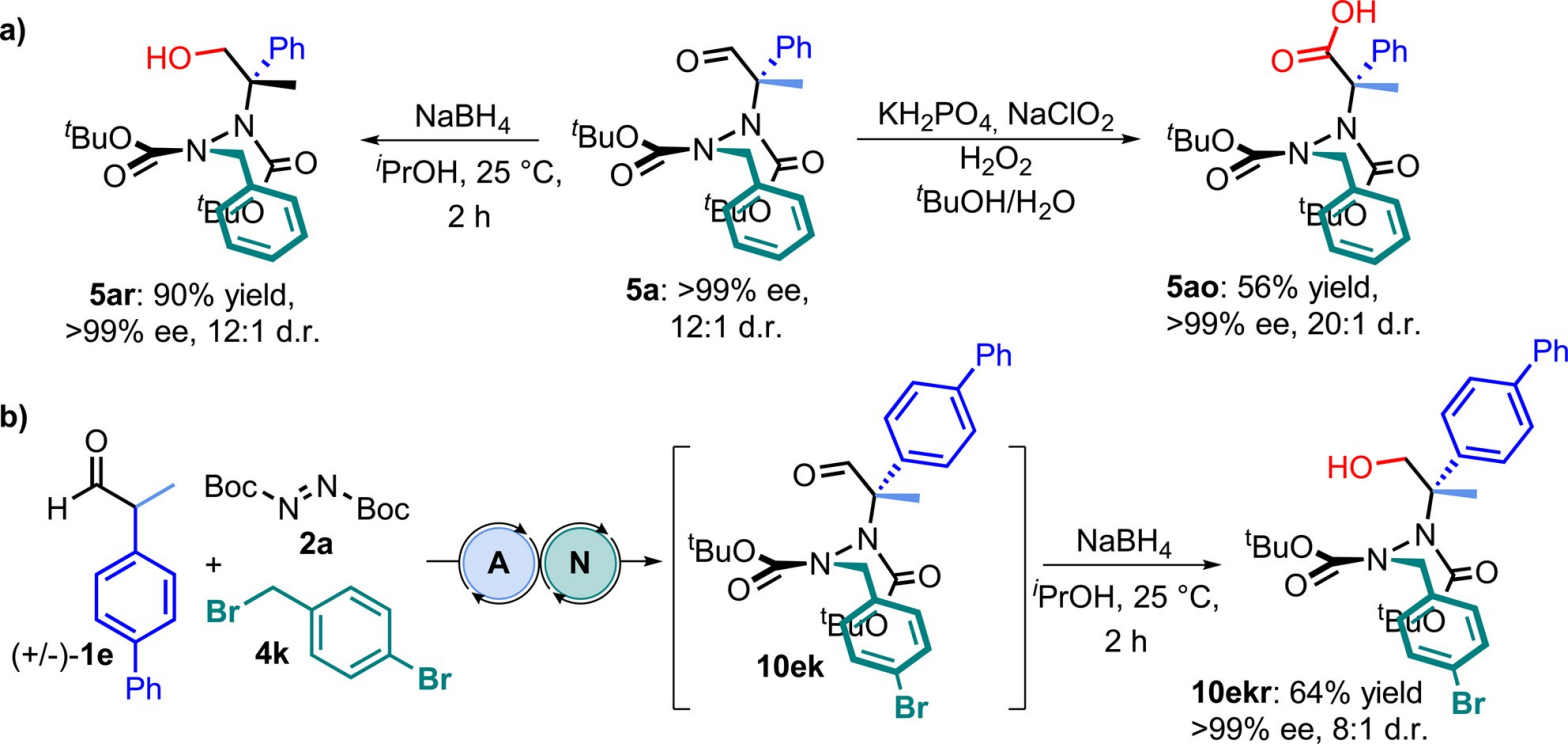
[a] Derivatization of hydrazides **5** **a**. [b] Three‐step reaction process: enamine, PTC, and reduction.

The stability of the N−N stereogenic axis was investigated^[15]^ by heating a decalin solution of pure **5** **a** to 88 °C and monitoring its diastereoisomerization conversion to **5** **a′** by HPLC on a chiral stationary phase. The results evidenced a ${\Delta G}_{rot}^{\neq }$ =28.32 kcal mol^−1^ with an equilibrium ratio of 57 : 43 in favor of **5** **a**. The diastereoisomerization of **5** **a′** to **5** **a** was determined to have a ${\Delta G}_{rot}^{\neq }$ =28.42 kcal mol^−1^. The DFT calculation estimated a ${\Delta G}_{rot}^{\neq }$ of 33.7 kcal mol^−1^ for the interconversion of **5** **a** to **5** **a′** at 88 °C, which was in agreement with the experimental value. In addition, the DFT calculation for **3** **a** evaluated a ${\Delta G}_{rot}^{\neq }$ of 20.6 kcal mol^−1^ in toluene at 25 °C, a result that suggests transient atropisomeric properties for tertiary hydrazides^[16]^ and reinforces the hypothesis that our two‐step catalytic process sequentially increases the steric hindrance around the N−N single bond, thus leading to atropisomeric hydrazides.

The absolute configuration of **5** **a′**, the major diastereosiomer obtained from the reaction of (*S*)‐**3** **a** with **4** **a** using catalyst **D**, was determined to be (*S,S*_a_) using single‐crystal X‐ray analysis.^[17]^ Consequently, the absolute configuration of **5** **a** was (*S,R*_a_). This result allowed us to propose a hypothesis for the reaction mechanism: once the primary amine catalyzes the formation of **3** **a** as an equilibrium mixture of rotamers, the ammonium cation of catalyst **N** associates favorably with the anion precursor of the *R_a_* stereogenic axis configuration in a way that minimizes steric repulsion and establishes cooperative and strong hydrogen bonds. The resulting stable cyclic structure may be responsible for the favored TS, leading to **5** **a** as the major diastereoisomer (Scheme 1).

**Scheme 1:**
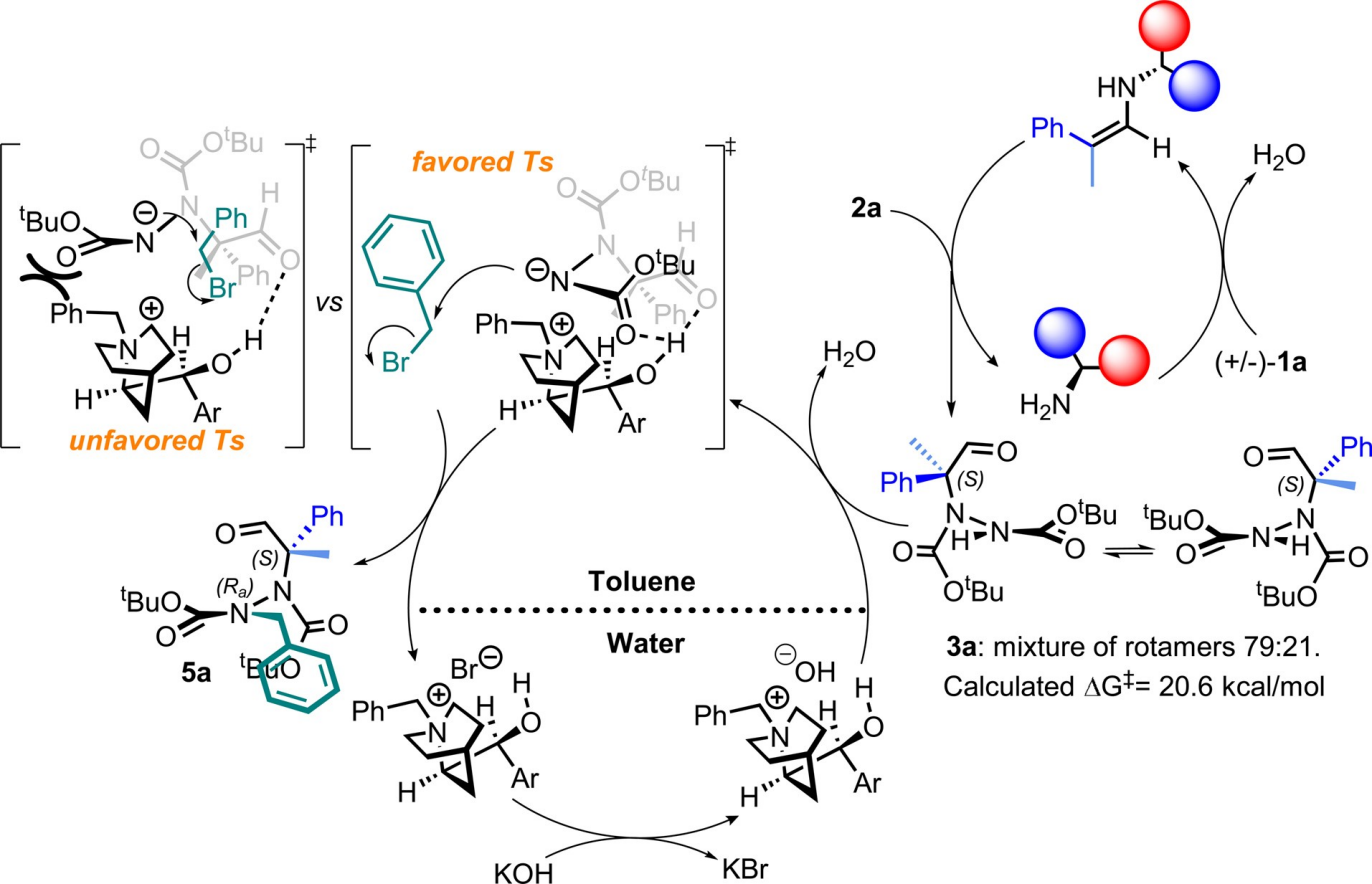
Proposed reaction mechanism.

In conclusion, we synthesized atropisomeric hydrazides for the first time using an efficient and easy one‐pot process based on an enantioselective and diastereoselective sequential organocatalytic protocol. This methodology comprises an unusual sequence of reactions that combine two catalytic steps, each dedicated to the construction of a particular element of chirality, providing easy access to all possible diastereoisomers in a stereodivergent fashion.

## Conflict of interest

The authors declare no conflict of interest.

## Supporting information

As a service to our authors and readers, this journal provides supporting information supplied by the authors. Such materials are peer reviewed and may be re‐organized for online delivery, but are not copy‐edited or typeset. Technical support issues arising from supporting information (other than missing files) should be addressed to the authors.

Supporting Information

Supporting Information

## Data Availability

The data that support the findings of this study are available in the supplementary material of this article.
