# Supplementary material for: Synthesis of Atropisomeric Hydrazides by One‐Pot Sequential Enantio‐ and Diastereoselective Catalysis
Source: Angew Chem Int Ed Engl. 2022 Sep 12;61(42):e202209895. doi: 10.1002/anie.202209895 (PMC9826270; doi:10.1002/anie.202209895)
Supplement: Supplementary file 2 — Supporting Information [file ANIE-61-0-s001.pdf]

Angewandte Chemie
Eine Zeitschrift der Gesellschaft Deutscher Chemiker

Supporting Information

Synthesis of Atropisomeric Hydrazides by One-Pot Sequential Enantio- and Diastereoselective Catalysis

*C. Portolani, G. Centonze, S. Luciani, A. Pellegrini, P. Righi, A. Mazzanti, A. Ciogli, A. Sorato, G. Bencivenni**

Synthesis of Atropisomeric Hydrazides by One-Pot Sequential Enantio- and Diastereoselective Catalysis

Chiara Portolani,[§,†] Giovanni Centonze,[§,†] Sara Luciani,[§] Andrea Pellegrini,[§,†] Paolo Righi,[§,†] Andrea Mazzanti,[§,†] Alessia Ciogli,[#] Andrea Sorato[#] and Giorgio Bencivenni[*,§,†]

[§]Department of Industrial Chemistry "Toso Montanari", University of Bologna, viale del Risorgimento 4, 40136, Bologna – Italy.

[†]Center for Chemical Catalysis – C^3, University of Bologna, Viale del Risorgimento 4, 40136, Bologna — Italy.

[#]Department of Chemistry and technologies of drug, Nuovo Edificio di Chimica, NEC- CU032, 3rd floor, room N° 1 Sapienza University of Rome piazzale A. Moro 5, 00185 Rome – Italy.

Contents

General 3
Optimization of the reaction conditions 4
First step 4
Second step 5
One-pot Sequential Reaction 6
Final optimization 8
Role of the catalyst in the control of stereoselectivity 9
Tests with different catalyst combinations 9
Synthesis of 5k 10
Elucidations on ^{1}H NMR spectra 11
Determination of the rotational energy barrier 14
Determination of the d.r. of 5a – HPLC peaks attribution 17
Determination of absolute configuration 22
Computational details 30
Synthesis of starting materials 38
Experimental procedures 39
General procedure for the sequential catalysis one-pot reactions 39
General procedure for reduction reactions 40
Oxidation reaction - 5ao 40
Three-step reaction process synthesis of 10ekr 41
Large scale reaction for 5a and 6k 41
Stereodivergent synthesis 41
Experimental details 43
NMR traces 72
HPCL traces 108
IR traces 165

General

All the NMR spectra were recorded on Inova 300 MHz, Gemini 400 MHz or Mercury 600 MHz Varian spectrometers for ^{1}H, 101 MHz for ^{13}C. The chemical shifts (δ) for ^{1}H, ^{13}C are given in ppm relative to internal standard TMS (0.0 ppm) or residual signals of $CHCl_3$ (7.26 ppm). The following abbreviations are used to indicate the multiplicity: s, singlet; d, doublet; t, triplet; q, quartet; m, multiplet; bs, broad signal. Purification of reaction products was carried out by flash chromatography (FC) on silica gel (230-400 mesh). Organic solutions were concentrated under reduced pressure on a Büchi rotary evaporator. X-ray data were acquired on a Bruker APEX-2 diffractometer. High Resolution Mass spectra were obtained from the Mass Facility of the Department of Chemistry and Drug Technology of the University of Rome on a Orbitrap Exactive, source: ESI (+): capillary temp: 250°C, spray voltage: 4.0 (kV), capillary voltage: 65 V, tube lens: 125 V. HPLC analysis on chiral stationary phase was performed on an Agilent 1100-series instrumentation. HPLC chromatograms of enantioenriched products were compared to racemic ones obtained with achiral catalysts. Optical rotations have not been determined since a mixture of diastereoisomers has been obtained in all cases. Infrared (ATR) spectra were recorded on a Perkin Elmer Spectrum Two FT-IR spectrometer equipped with an ATR probe. Signals are reported as strong (s), medium (m), and weak (w). Melting points (uncorrected) were determined with a Stuart Scientific SMP3 apparatus. All reactions were carried out in air; all evaporations were performed without heating to avoid rotation of the chiral axis. Starting materials **1a**, **2a-b**, **4a**, **4e-h**, **4j**, **4k**, **4l**, **a** and chiral catalyst **D** were purchased from suppliers. Aldehydes **1b-j**[1] and electrophiles **4b**,[2] **4c**,[2] **4d**,[2] **4i**,[2] **4m**,[2] **b**,[3] that are all commercially available, were prepared following literature procedures and their ^{1}H-NMR spectra were consistent with those previously reported. Primary amine catalysts **A** and ***ent*-A**,[4] and phase-transfer catalysts **B-Q**[5] were obtained following the reported literature procedures. Catalyst **G** was prepared following the described procedure[6] and ^{1}H-NMR spectrum was consistent with literature. The off-column HPLC experiments were made to determine the energy barrier of N-N rotation. The pure **5a** (peak 4) or **5a'** (peak 1) were dissolved in decalin and heated in oven at 88 °C. The diastereomerization process has been studied over time by HPLC on chiral stationary phase. Analytical conditions: Chiralpak IC 5μm

[1] T. Baumann, H. Vogt, S. Bräse, *Eur. J. Org. Chem.,* **2007**, 266 – 282.
[2] E. Doni, B. Mondal, S. O'Sullivan, T. Tuttle, J. A. Murphy *J. Am. Chem. Soc.* **2013**, *135*, 10934 – 10937.
[3] a) A. Varela, L. K. B. Garve, D. Leonori, V. K. Aggarwal, *Angew. Chem. Int. Ed.* **2017**, *56*, 2127 – 2131. b) X. H. Yang, J. P. Li, D. C. Wang, M. S. Xie, G. R. Qu, H. M. Guo *Chem. Commun.* **2019,** *55*, 9144 – 9147.
[4] C. Cassani, R. Martín-Rapún, E. Arceo, F. Bravo, P. Melchiorre *Nat. Protoc.* **2013**, *8*, 325 – 344.
[5] a) S.-S. Jew, M.-S. Yoo, B.-S. Jeong, I.-Y. Park, H.-G. Park *Org. Lett.* **2002**, *4*, 4245. b) M. Lian, Z. Li, J. Du, Q. Meng, Z. Gao, *Eur. J. Org. Chem*. **2010**, 6525 – 653. c) For catalysts P and Q: Chinchilla, R., Mazón, P., Nájera, C., & Ortega, F. J. *Tetrahedron Asymmetry.* **2004**, *15*, 2603 – 2607.
[6] Y.-C. Chan, X. Wang, Y.-P. Lam, J. Wong, Y.-L. S. Tse, Y.-Y. Yeung *J. Am. Chem. Soc.* **2021**, *143*, 12745 – 12754.

(150x4.6mm); Flow rate: 1.00 mL/min; Eluent: 98/2 hexane/i-PrOH, UV 267 nm (corresponding to an isosbestic point).

Optimization of the reaction conditions

The two steps were first studied separately. 2-Phenyl-propionaldehyde **1a**, di-*tert*-butyl azodicarboxylate **2a** and benzyl bromide **4a** were used as reagents for the optimization of the reaction conditions.

First step

L-Proline catalysis was first tested for the amination reaction following Bräse's procedure[1] (Table S1, entry 1); better results were obtained exploiting an amino quinine catalysis using the protocol reported by Greck,[7] with 9-epi-deoxy-amino-quinine (**A**) as catalyst, TFA as additive and chloroform as solvent (entry 2). The racemic product was obtained using benzylamine as catalyst (entry 3).

Table S1 – First step optimization.

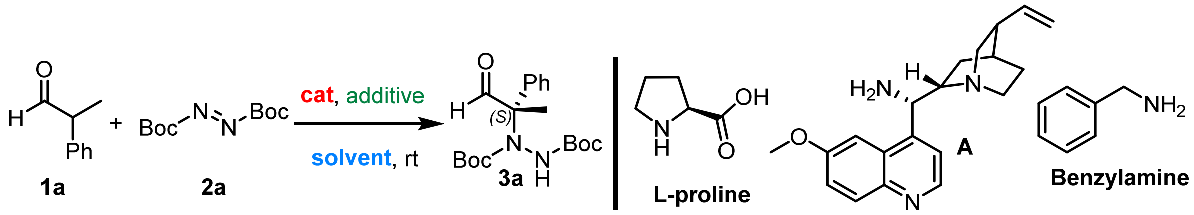

Entry	Cat (mol%)	Additive	Solvent (M)	Yield[c] (time)	e.e.[d]
**1**[a]	**L-Proline** (50 mol%)	-	CH_2Cl_2 (0.1M)	61% (5 days)	88% (*R*)
**2**[b]	**A** (5 mol%)	TFA	$CHCl_3$ (0.6M)	90% (6 h)	94% (*S*)
**3**[b]	**BnNH$_2$** (10 mol%)	TFA	$CHCl_3$ (0.6M)	90% (1 day)	rac

[a]The reaction was performed with 1 mmol of **1a**, 1.5 eq of **2a**, 50 mol% of catalyst in 10 mL of solvent. [b]The reaction was performed using 3 mmol of **2a**, 1.2 eq of **1a**, 5 mol% of catalyst, 0.15 eq of TFA and 6 mL of solvent. [c]Isolated yield. [d]Determined by HPLC using chiral stationary phase; absolute configuration known from literature.

[7] A. Desmarchelier, H. Yalgin, V. Coeffard, X. Moreau, C. Greck, *Tetrahedron Lett.* **2011**, *52*, 4430-4432.

Second step

Alkylation of the trisubstituted hydrazide **3a** (94% ee) was attempted exploiting a phase transfer catalysis using benzyl bromide **4a** with 10% mol of PTC catalyst, an aqueous solution of KOH (50% w/w) and toluene 0.05 M, as described in many PTC procedures[8].

Table S2 – Screening of PTC catalysts.[a]

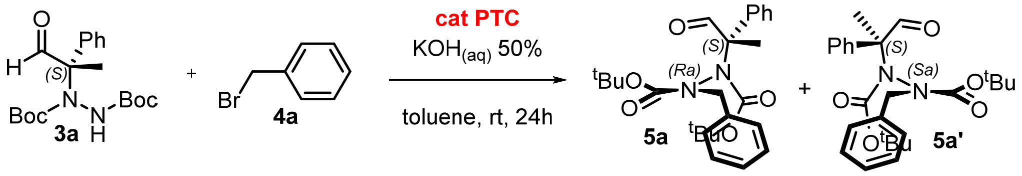

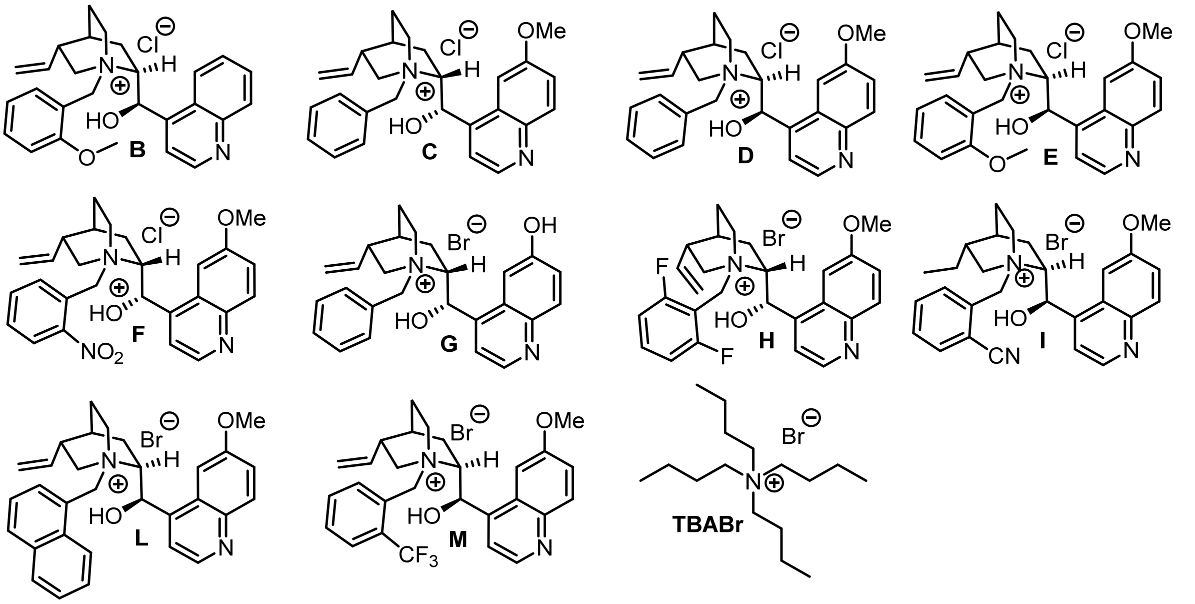

Entry	Cat	Yield[b]	d.r.(5a:5a')[c]	e.e. (major)[c]
**1**	**B**	63%	1:3.4	98%
**2**	**C**	61%	6:1	98%
**3**	**D**	60%	1:6.3	99%
**4**	**E**	60%	1:4.5	99%
**5**	**F**	42%	3:1	97%
**6**	**G**	70%	2.6:1	90%

[8] a) K. Maruoka, T. Ooi *Chem. Rev*. **2003**, *103*, 3013–3028. b) K. Maruoka *Org. Process. Res. Dev*. **2006**, *12*, 679-697. c) S. S. Jew, H. G. Park, *Chem. Commun.* **2009**, *46*, 7090-7103.

**7**	**H**	42%	1.6:1	96%
**8**	**I**	18%	1:2.5	97%
**9**	**L**	38%	1:3.6	80%
**10**	**M**	21%	1:2.3	98%
**11**	**TBABr**	63%	1:1	94%[d]

[a]The reactions were performed using 0.1 mmol of **3a**, 1.0 eq of **4a**, 10% mol of catalyst, 4 mL of an aqueous solution of KOH (50% w/w) and 2 mL of toluene. [b]Isolated yield as mixture of diastereoisomers. [c]Determined by HPLC using chiral stationary phase. [d]The ee% of the minor diastereoisomer is 94.

Quinidinium and quininium chloride **C** and **D** gave the best results, showing a similar behavior in terms of yield and atroposelectivity (with opposite d.r.); further tests were carried out using commercially available catalyst **D**.

One-pot Sequential Reaction

The two steps were carried out in sequence, without the isolation and purification of the intermediate. A first attempt was carried out directly adding the reagents for the second step to the flask, after the amination step was completed (Table S3- entry 1), but modest results were obtained. Evaporating chloroform before adding toluene led to the formation of **5a** + **5a'** with a yield and d.r. that are perfectly consistent with those obtained when the second step was performed separately, starting from **3a** (Table S3 - entry 2 vs Table S2 - entry 3); this suggests that the different conditions of the two steps are compatible when put together, except for the solvent: when traces of chloroform are present, lower yield and stereoselectivity are obtained. This was confirmed when, trying to use a common solvent for both steps, the whole reaction was run in chloroform (Table S3- entry 3). Promising results were obtained using 0.6 M toluene for the first step (entry 4) since it afforded the isolated trisubstituted hydrazide **3a** in 96% yield and 94% ee. In this case however it is necessary to change the reaction flask when switching from the first to the second step, as a higher volume is necessary. To bypass the problem, the first step was diluted to 0.1 M, surprisingly enhancing the enantioselectivity of the amination to 98%, with a 80% yield on **3a** obtained using 1,1 eq of **2a**; on the other hand, concentrating the second step to 0.1 M led to a lower d.r. (entry 5). Hence solvent concentrations were set as reported in entry 6.

Table S3 – Optimization of one-pot conditions.[a]

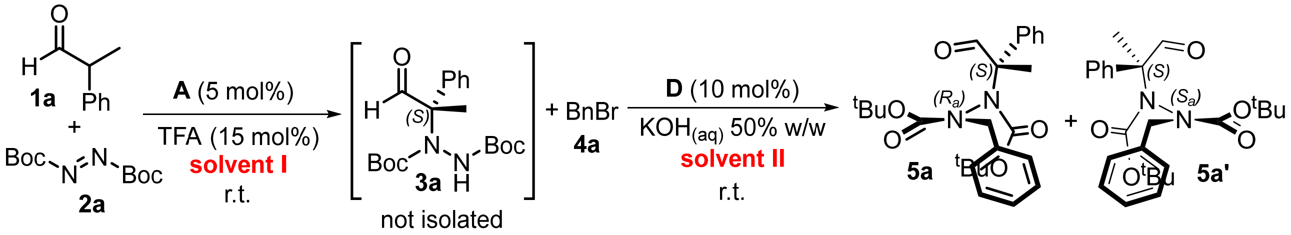

Entry	Solvent I	Solvent II	Yield[e]	d.r. (5a:5a')[f]	e.e. (major)[f]
1	$CHCl_3$ (0.6M)	Toluene (0.05M)	53%	1:5.4	97%
2[b]	$CHCl_3$ (0.6M)	Toluene (0.05M)	65%	1:6.2	99%
3	$CHCl_3$ (0.6M)	$CHCl_3$ (0.05M)	35%	1:1.2	96%
4[c]	Toluene (0.6M)	Toluene (0.05M)	60%	1:6.5	97%
5[d]	Toluene (0.1M)	Toluene (0.1M)	62%	1:5.5	98%
6[d]	Toluene (0.1M)	Toluene (0.05M)	65%	1:6.5	99%

[a]The reactions were performed using 0.1 mmol **1a**, 1.0 eq of **2a**, 5 mol% of catalyst **A**, 0.15 eq of TFA and solvent **I**; after 24 h, 1.0 eq of **4a** was added together with 10 mol% of catalyst **D**, 4 mL of an aqueous solutions of KOH (50% w/w) and solvent **II**. [b]The solution was concentrated before adding the reagents for the second step. [c]Intermediate **3a** was isolated with a yield of 96% and 94% ee. [d]First step conducted with 1.1 eq of **2a**; intermediate **3a** was isolated with a yield of 80% and 98% ee. [e]Isolated yield as mixture of diastereoisomers. [f]Determined by HPLC using chiral stationary phase.

Final optimization

Table S4 – Counterion screening.[a]

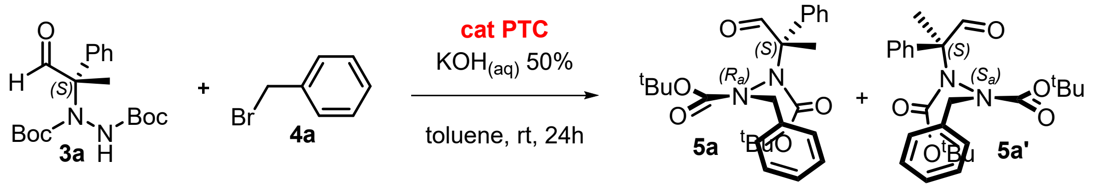

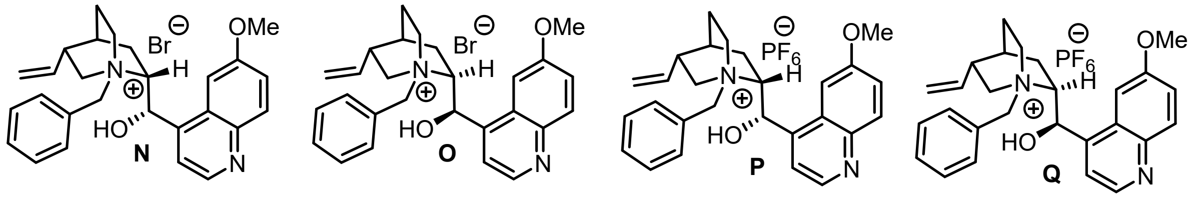

Entry	Catalyst	Yield[b]	d.r. (5a:5a')[c]	e.e. (major)[c]
**1**	**N**	80%	9:1	99%
**2**	**O**	49%	1:6	>99%
**3**	**P**	53%	7.5:1	98%
**4**	**Q**	50%	1:6	99%

[a]The reactions were performed using 0.1 mmol of **3a**, 1.0 eq of **4a**, 10% mol of catalyst, 4 mL of an aqueous solution of KOH (50% w/w) and 2 mL of toluene. [b]Isolated yield as mixture of diastereoisomers. [c]Determined by HPLC using chiral stationary phase.

Br^- and $PF6^-$ quininium and quinidinium catalysts were investigated instead of the chlorine correspondent **C** and **D**, obtaining equal to better results. Differences between quinine and quinidine scaffold were found in this case, leading to the choice of **N** as catalyst.

Table S5 – Screening of temperature.[a]

Entry	Cat	T (°C)	Yield[b]	d.r. (5a:5a')[c]	e.e. (major)[c]
**1**	**N**	0	64%	11:1	99%
**2**	**N**	-5	81%	13:1	98%
**3**	**N**	-20	63%	13:1	98%

[a]The reactions were performed using 0.1 mmol of **3a**, 1.0 eq of **4a**, 10% mol of catalyst, 4 mL of an aqueous solution of KOH (50% w/w) and 2 mL of toluene. [b]Isolated yield as mixture of diastereoisomers. [c] Determined by HPLC using chiral stationary phase.

Role of the catalyst in the control of stereoselectivity

Tests with different catalyst combinations

Some tests were carried out to verify how the stereoselectivity is controlled, expecially in the second step, and if the substrate has any role in that. Different combinations of achiral catalysts **$BnNH_2$** and **TBABr**, and chiral ones **A** and **D** were used (Table S6).

Table S6 – Tests with different combination of racemic or chiral catalysts.[a]

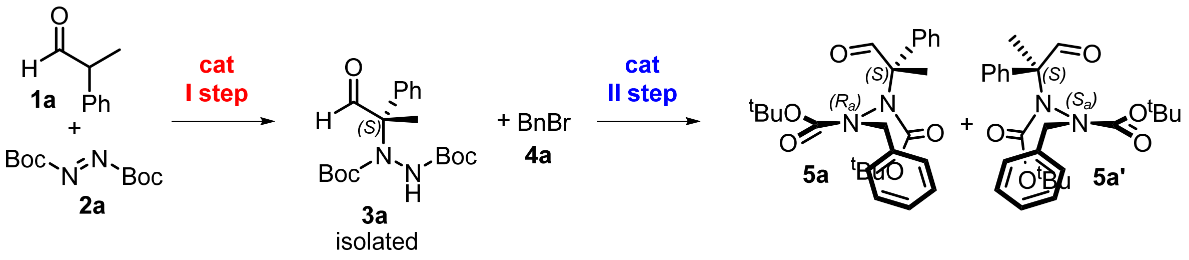

Entry	Cat I step	e.e. (3a)	Cat II step	d.r. (5a:5a')[b]	e.e. (5a)[b]	e.e. (5a')[b]
**1**	**$BnNH_2$**	0%	**TBABr**	1:1	0%	0%
**2**	**$BnNH_2$**	0%	**D**	1:1	75%	77%
**3**	**A**	94%	**TBABr**	1:1	94%	94%
**4**	**A**	94%	**D**	1:6.3	73%	99%

[a]The first step was performed using 0.1 mmol **1a**, 1.0 eq of **2a**, 5 mol% of catalyst, 0.15 eq of TFA and toluene 0.1M; after 24 h, the reaction was stopped and **3a** isolated by column chromatography. The second step was performed using 0.1 mmol of **3a**, 1.0 eq of **4a**, 10% mol of catalyst, 4 mL of an aqueous solution of KOH (50% w/w) and 2 mL of toluene. [b]Determined by HPLC using chiral stationary phase.

When catalyst **D** was used in the second step starting from racemic **3a** (entry 2), a 1:1 d.r was obtained, with both diastereoisomers having the same enantiomeric excess (small differences – 75%/77% - are due to imprecisions during HPLC peaks integration, as the first three peaks can not be perfectly separated). This result shows how catalyst **D** is able to control the formation of a specific axis configuration in the same way starting from (*R*)-**3a** and (*S*)-**3a**, independently by the chiral center.

When **TBABr** was employed for the alkylation of **3a** (94% e.e.), a 1:1 mixture of diastereoisomers was obtained (entry 3). This test confirmed that the stereogenic center on the trisubstituted hydrazide does not influence the choice of the chiral axis. Finally, using the two chiral catalysts (entry 4), a 1:6,3 d.r. was obtained, with a significant increase of the e.e. of the major diastereoisomer.

Synthesis of 5k

To better clarify this aspect, the enantioselective synthesis of **11** was carried out; the only stereogenic element in this case is the N-N axis, and catalyst **D** gave a 54% e.e., using the same reaction conditions as for the synthesis of **5a**. With no other stereogenic elements that could interfere, this result demonstrates that the catalyst is the only responsible of the stereoselectivity.

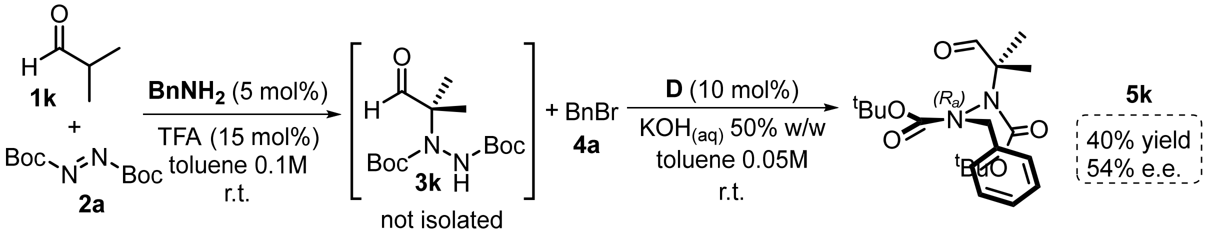

(R_a)-di-*tert*-butyl 1-benzyl-2-(2-methyl-1-oxopropan-2-yl)hydrazine-1,2-dicarboxylate (5k)

The reaction was performed starting from 0.3 mmol of **1k**. The crude mixture was purified by flash column chromatography (hexane:Et_2O = 8:2).

Yield= 40% (47 mg).

The e.e. was determined by HPLC analysis on a Daicel Chiralpak IC column: hexane/*i*-PrOH 98/2, flow rate 1 mL/min, 25 °C, λ = 220 nm: t_1= 11 min, t_2= 12 min.

^{1}H NMR (400 MHz, $CDCl_3$): δ 9.64 – 9.25 (m, 1H), 7.34 (m, 5H), 5.18 – 4.14 (m, 2H), 1.56 – 1.49 (m, 9H), 1.43 (s, 3H), 1.34 (s, 9H), 1.05 (s, 3H).

^{13}C NMR (101 MHz, $CDCl_3$): δ 201.09, 200.26, 200.17, 199.31, 156.90, 156.43, 155.70, 155.50, 154.70, 153.98, 153.06, 152.68, 136.61, 136.47, 136.39, 136.34, 130.38, 130.28, 129.96, 129.89, 128.50, 128.46, 128.43, 128.32, 128.03, 83.37, 82.11, 82.07, 82.00, 81.98, 81.91, 67.18, 67.12, 66.83, 66.69, 56.39, 55.59, 54.97, 54.64, 53.40, 30.30, 29.67, 28.32, 28.28, 28.25, 28.10, 28.07, 27.99, 27.97, 26.36, 21.33, 21.24, 21.11, 20.95, 20.77, 20.23, 20.07.

Elucidations on ^{1}H NMR spectra

A certain complexity of ^{1}H NMR spectra was found for every pure product: beyond the peaks of the two diastereoisomers and those correspondent to diastereotopic protons, other peaks appear in every spectrum. We attributed this phenomenon to different conformations arising from the rotation of the amidic single bonds of NBoc groups. This behavior has been well described by Rinaldi on tetrasubstituted hydrazides.[9] We indeed calculated a $\Delta G^{\ddagger}_{rot}$ of 18 kcal/mol for NBoc groups on **5a**, a value making new visible rotamers for the NMR acquisition times. A representative peak of the ^{1}H NMR spectrum is the proton of the aldehyde at around 9-10 ppm: it corresponds to a single proton that does not have any coupling and it is not diastereotopic. For these reasons more than two diastereomeric peaks are present in each case, and it is not possible to calculate the d.r. Some examples are reported to clarify the problem, highlighting the 9-10 ppm area in each case. Remarkably, also the ^{1}H NMR of product **5k**, that is a mixture of enantiomers, shows the presence of at least four conformations due to the NBoc groups rotamers (Spectrum 5).

Spectrum 1 - ^{1}H NMR of racemic **5a+5a'**.

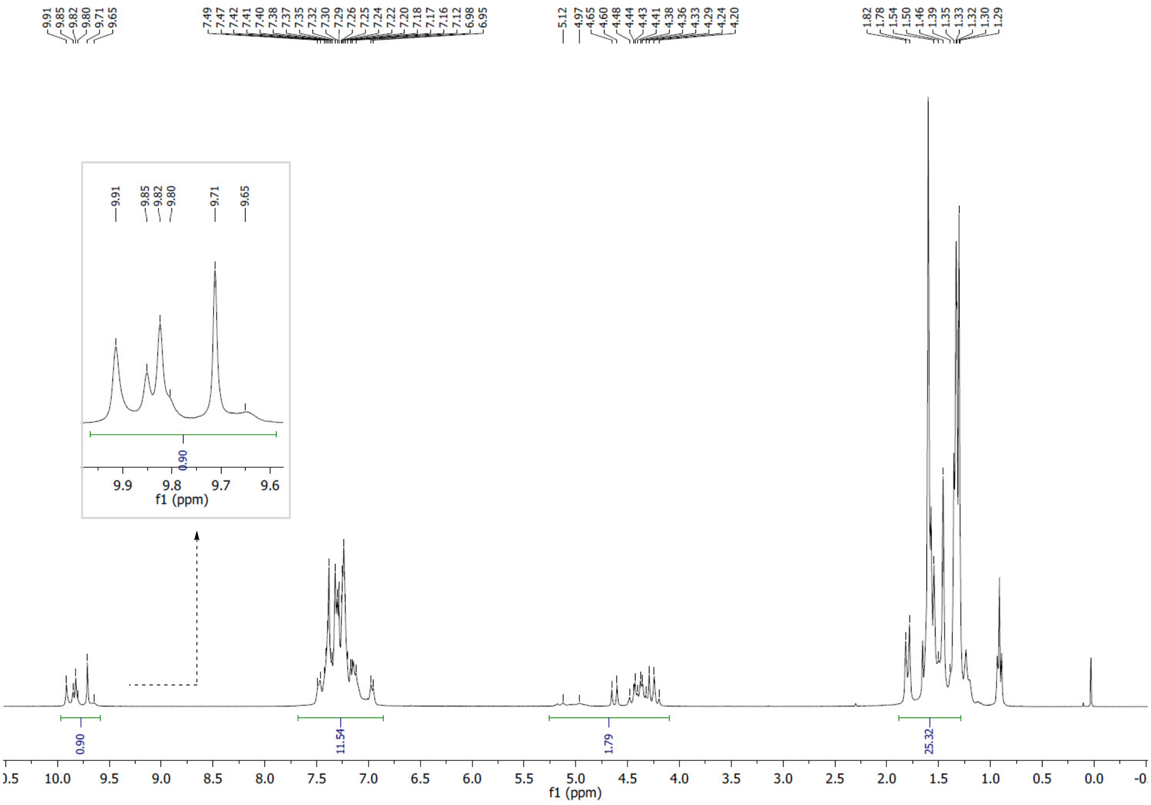

[9] P. Amabili, A. Amici, G. Campisi, G. Guerra, M. Monari, M. Orena, F. Piccinelli, S. Rinaldi, A. Tolomelli *Eur. J. Org. Chem.* **2018**, 6524.

Spectrum 2 - ^{1}H NMR of pure **5a'** (S,S_a).

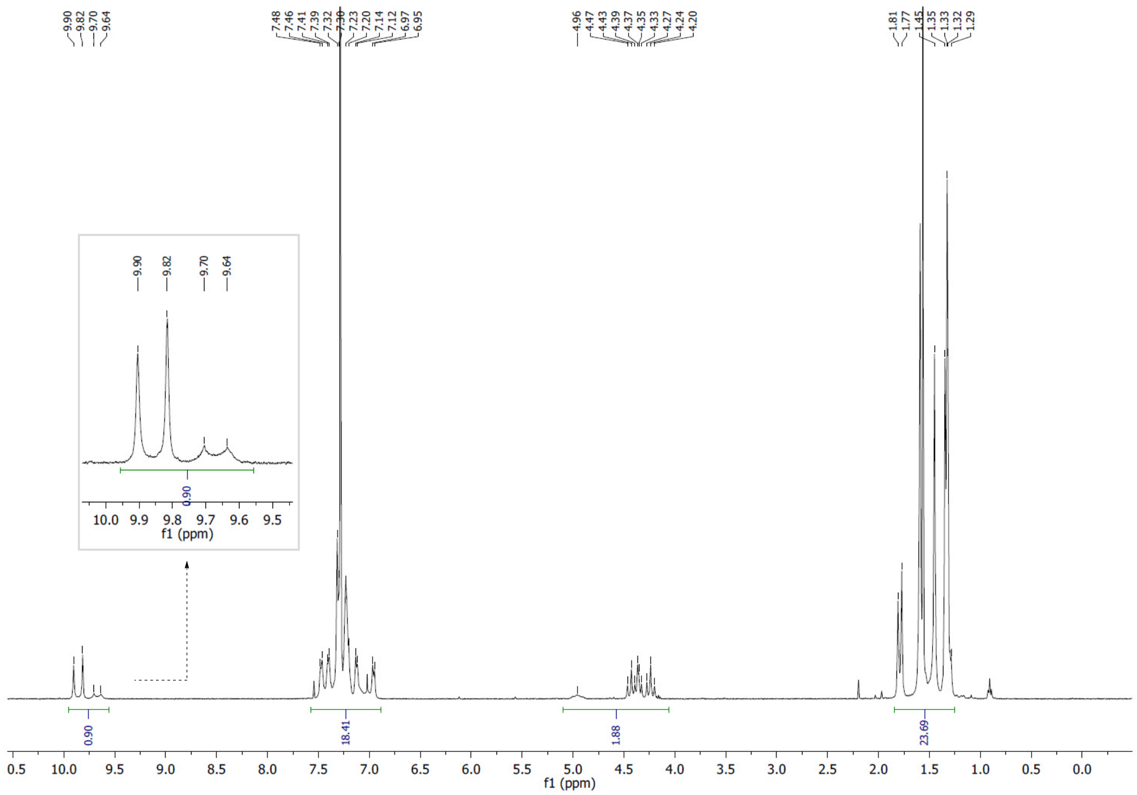

Spectrum 3 - ^{1}H NMR of pure **5a** (S,R_a).

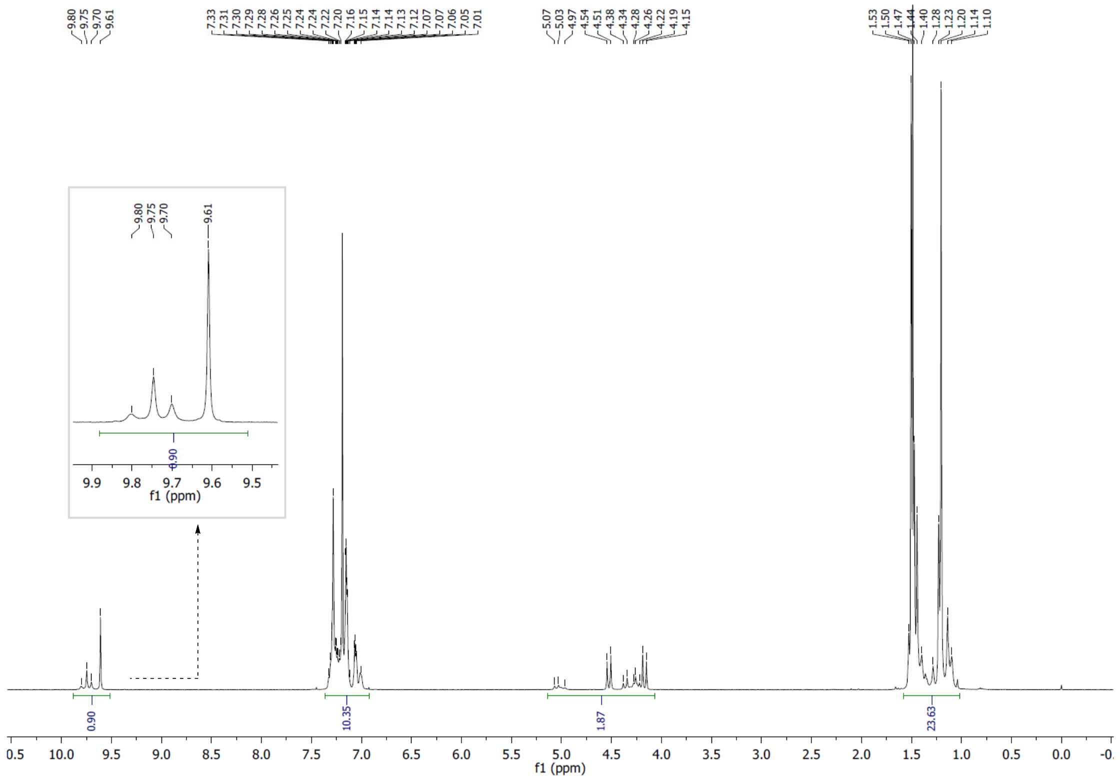

Spectrum 4 - ^{1}H NMR of product mixture **5a:5a'** with a 13:1 d.r.

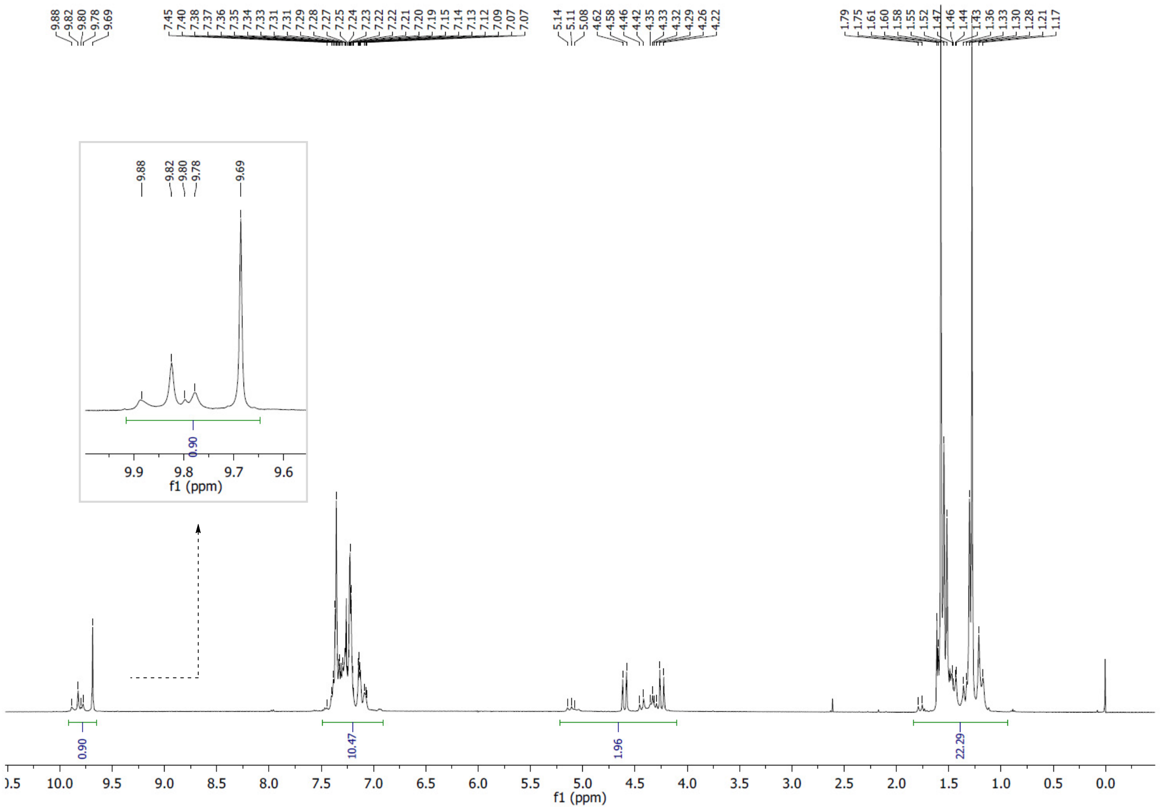

Spectrum 5 - ^{1}H NMR of product **5k** (racemic mixture).

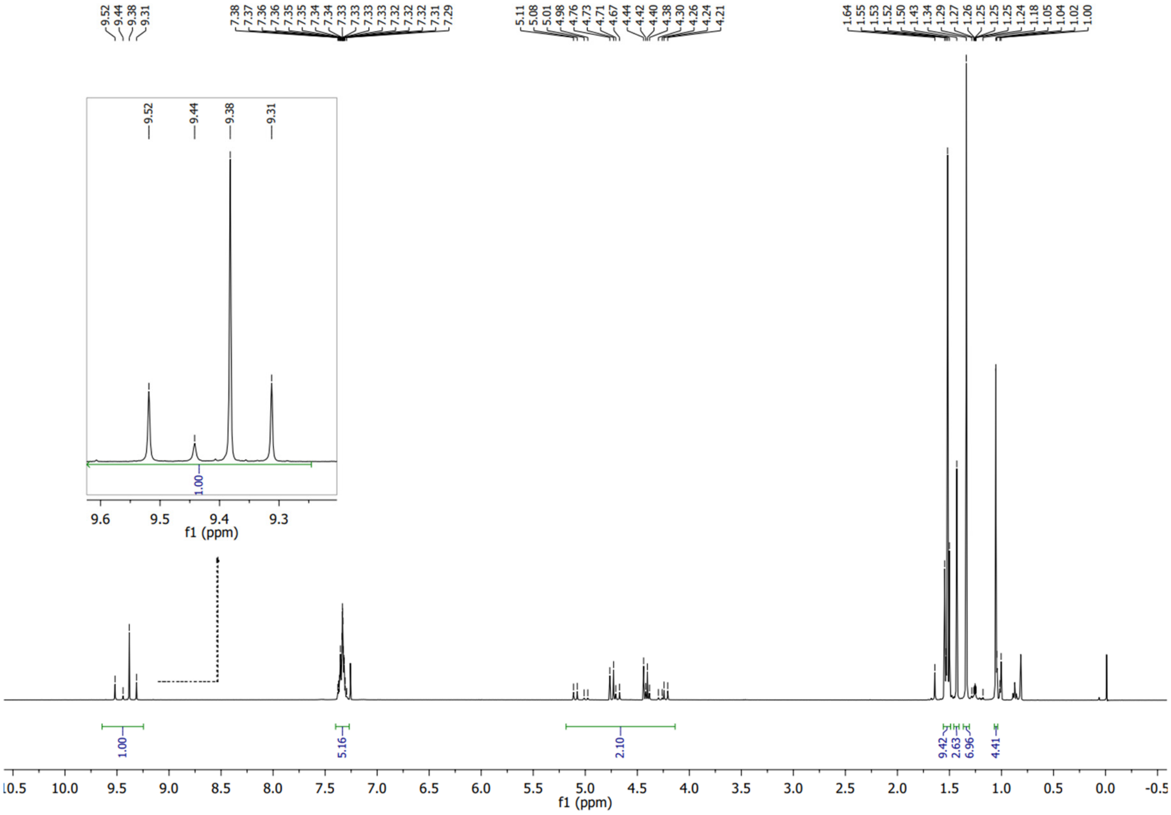

Determination of the rotational energy barrier

The pure **5a'** (peak 1) or **5a** (peak 4) were dissolved in decalin in a close vial and heated in oven at 88 °C. The diastereomerization process has been studied over time by HPLC on chiral stationary phase. Analytical conditions: Chiralpak IC 5µm (150x4.6mm); Flow rate: 1.00 mL/min; Eluent: 98/2 hexane/i-PrOH, UV 267 nm (corresponding to an isosbestic point) (Figure S1). Column temperature: 25 °C. From equations, rate constants were 0.0028 min^{-1} for the interconversion of **5a'** to **5a** (Figure S2) and 0.0033 min^{-1} for the interconversion of **5a** to **5a'** (Figure S3).

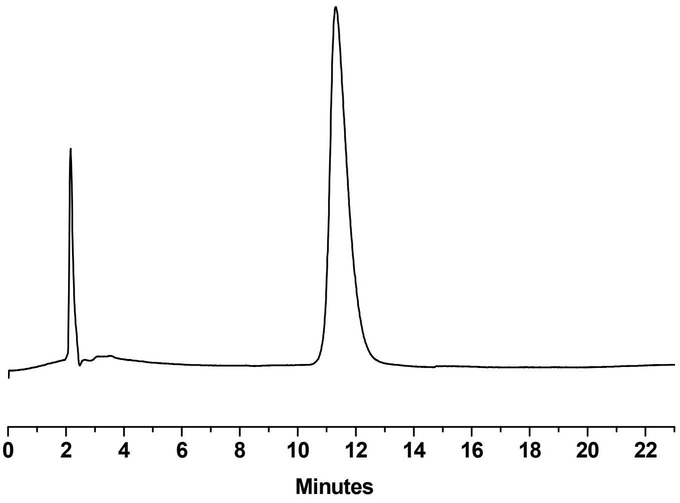

Chromatogram at start: 100% of **5a'**

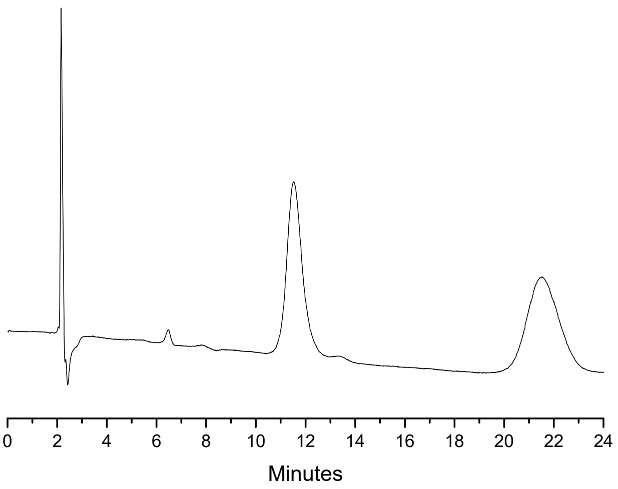

Chromatogram at equilibrium: 43% of **5a'** and 57% of **5a**

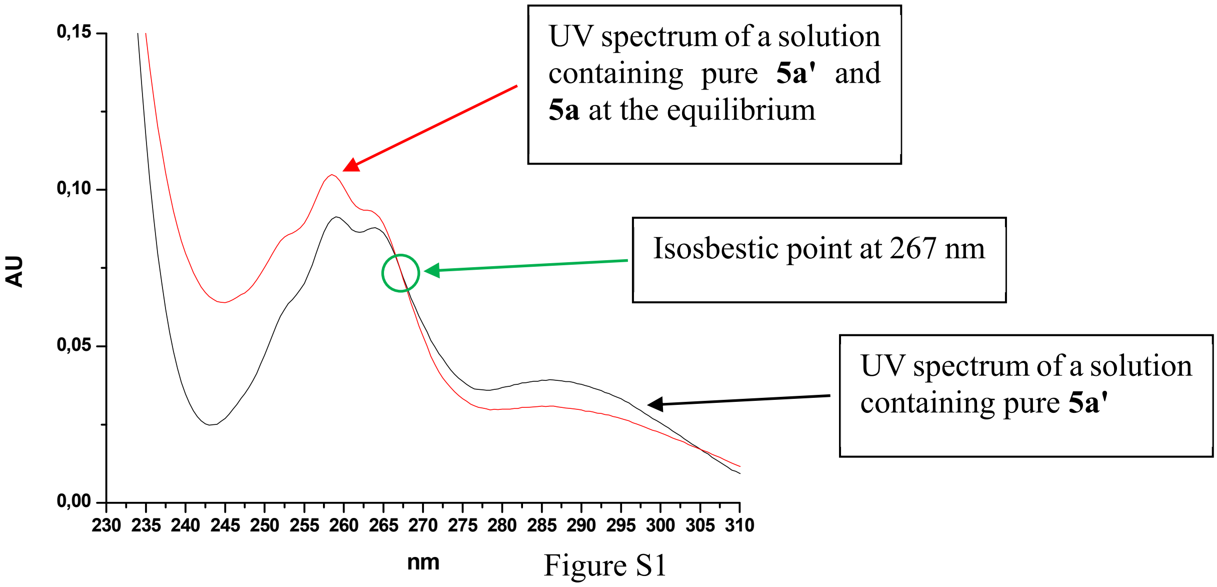

Figure S1

Peak 1 (5a') to peak 4 (5a)

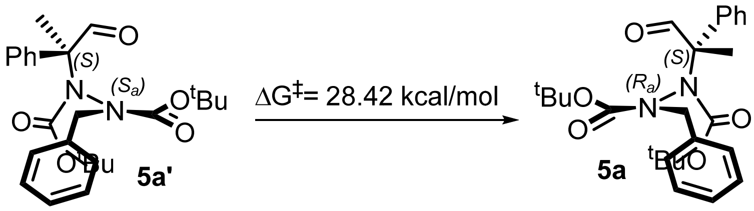

Temp.(°C)	K_{eq}	$k+k_{-1}$(min^{-1})	k(min^{-1})	k_{-1}	$\Delta G_{1\text{-}4}$ (Kcal/mol)	$\Delta G_{1\text{-}4}$(KJ/mol)
88	1,31	0,0050	0,00284	0,00216	28,42	118,82

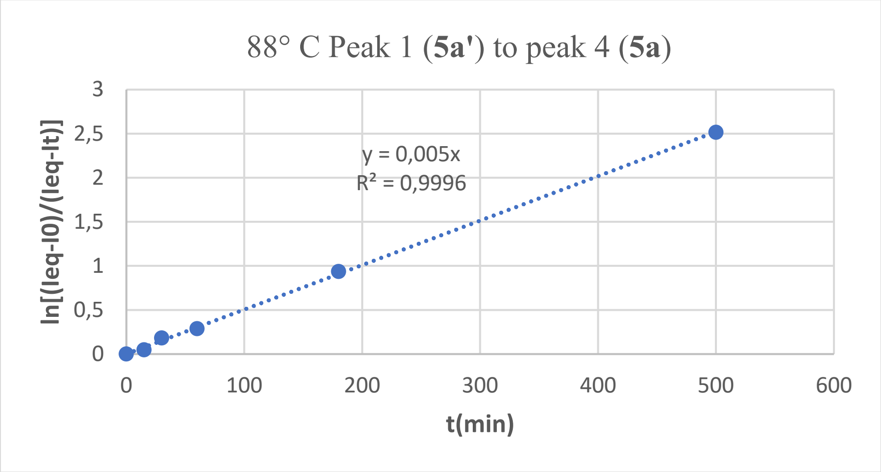

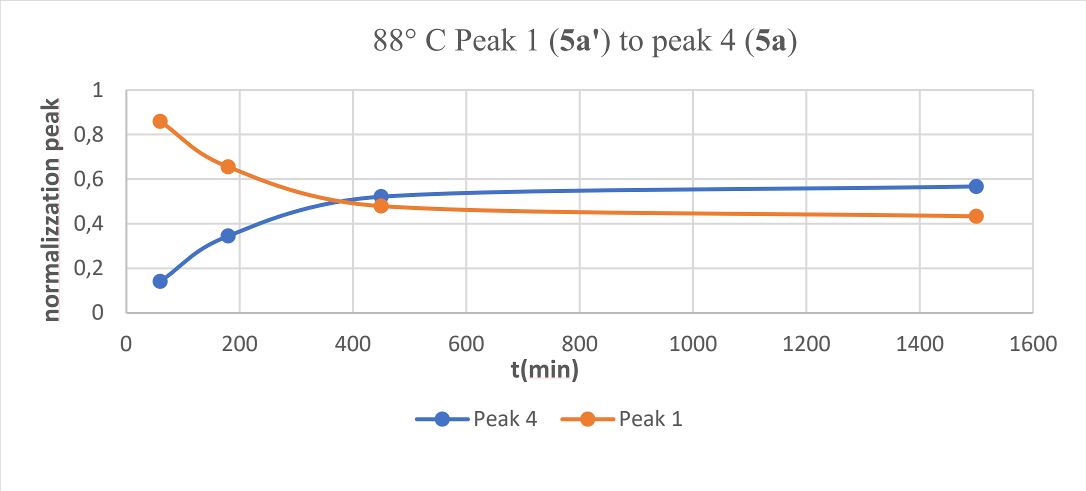

Figure S2: determination of the rotational energy barrier for the interconversion of **5a'** to **5a**.

Peak 4 (5a) to peak 1 (5a')

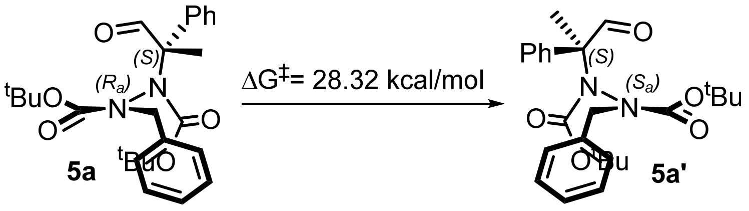

Temp. (°C)	K_{eq}	$k+k_{-1}(min^{-1})$	k (min^{-1})	k_{-1}	$\Delta G_{4\text{-}1}$(Kcal/mol)	$\Delta G_{4\text{-}1}$(KJ/mol)
88	0.78	0,0075	0,0033	0,0042	28,32	118,49

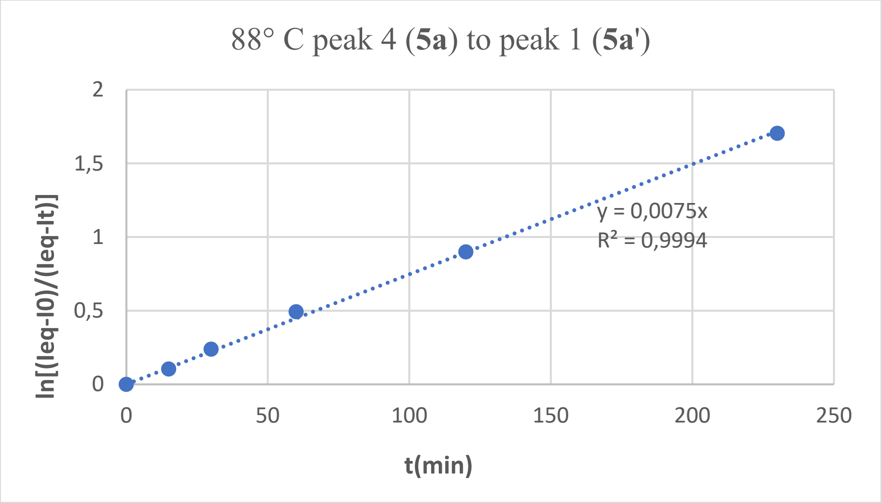

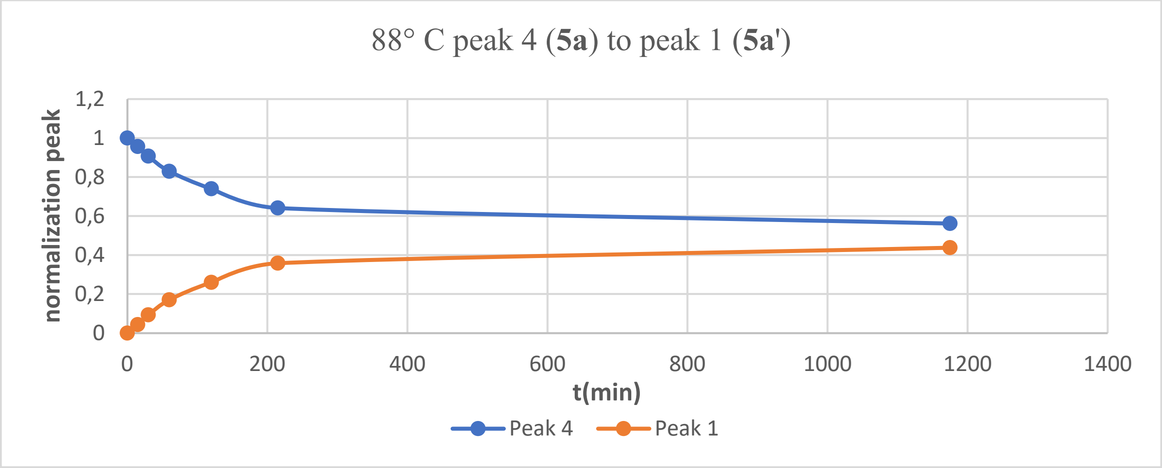

Figure S3: determination of the rotational energy barrier for the interconversion of **5a** to **5a'**.

Determination of the d.r. of 5a – HPLC peaks attribution

Racemic product **5a** + **5a'** was obtained with benzylamine and tetrabutylammonium bromide as catalysts for the first and second step, respectively. HPLC chromatogram is reported, showing the peaks of the four diastereoisomers (IC column.: hexane/*i*-PrOH 98/2, flow rate 1 mL/min, 25 °C, λ = 220 nm. t_1= 14 min, t_2= 15 min, t_3= 16 min, t_4= 26 min). (Figure S4).

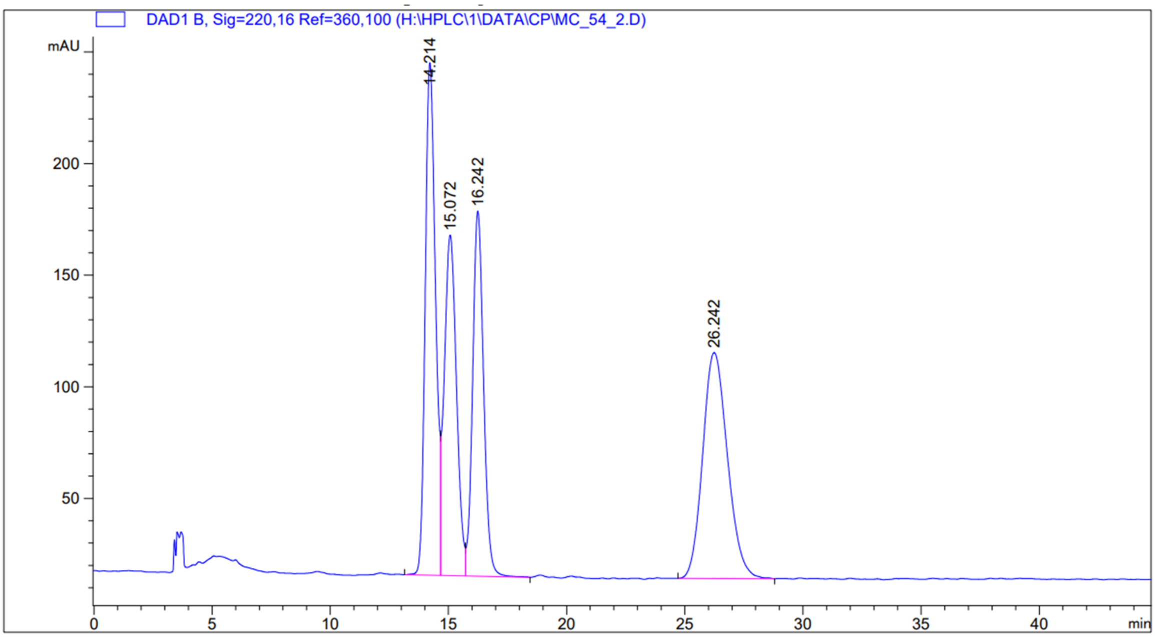

Peak #	RetTime [min]	Type	Width [min]	Area [mAU*s]	Height [mAU]	Area %
1	14.214	BV	0.4635	6916.86572	229.59039	27.6327
2	15.072	VV	0.5485	5472.29736	152.55441	21.8617
3	16.242	VB	0.4950	5255.90186	163.52090	20.9972
4	26.242	BB	1.1154	7386.37988	101.21966	29.5084

Figure S4 – HPLC chromatogram of racemic product **5a** + **5a'**.

Catalyst **D** was tested with racemic trisubstituted hydrazide **3a**. Major peaks 1 and 2 were assigned as a diastereomeric couple of **5a** and **5a'** with same axis and different stereocenter (Figure S5).

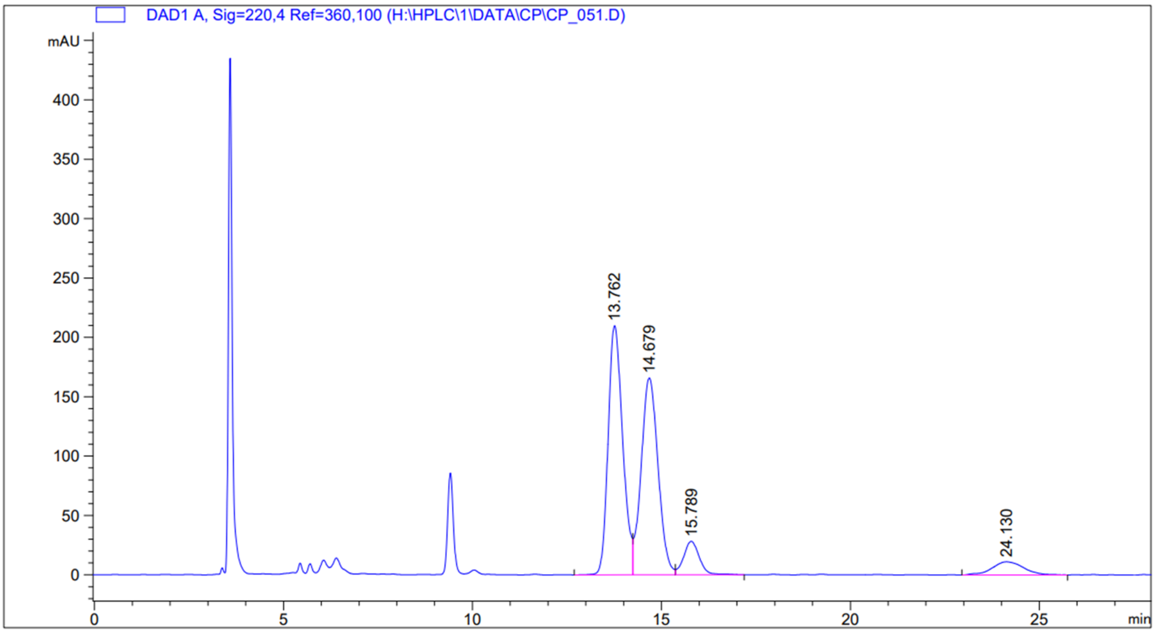

Peak #	RetTime [min]	Type	Width [min]	Area [mAU*s]	Height [mAU]	Area %
1	13.762	BV	0.4120	5613.02295	209.89731	45.8551
2	14.679	VV	0.4834	5165.57813	165.84851	42.1998
3	15.789	VB	0.4434	821.21252	28.23110	6.7088
4	24.130	BB	0.8786	640.96161	11.08535	5.2363

Figure S5 – HPLC chromatogram of **5a** + **5a'** obtained with benzylamine and **D**.

Catalyst **D** was tested with enantioenriched **3a** (94% ee); the third peak is absent and the second is minimal (Figure S6).

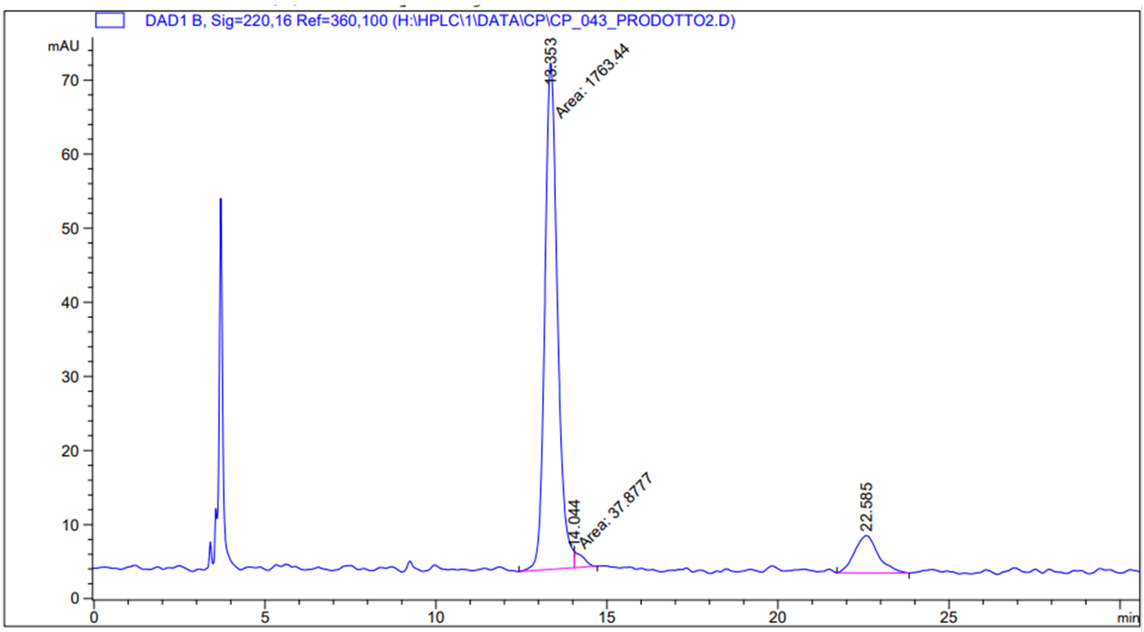

Peak #	RetTime [min]	Type	Width [min]	Area [mAU*s]	Height [mAU]	Area %
1	13.353	MF	0.4299	1763.44080	68.35848	86.2826
2	14.044	FM	0.2888	37.87766	2.18614	1.8533
3	22.585	BB	0.7581	242.47900	5.03303	11.8641

Figure S6 – HPLC chromatogram of **5a** + **5a'** obtained with **A** and **D** at r.t.

Catalyst **C** was tested with enantioenriched **3a** (94% ee). The second peak is absent and the third is minimal (Figure S7). Peaks 1 and 4 are thus assigned as a diastereomeric couple of **5a** and **5a'** with same stereocenter and different chiral axis.

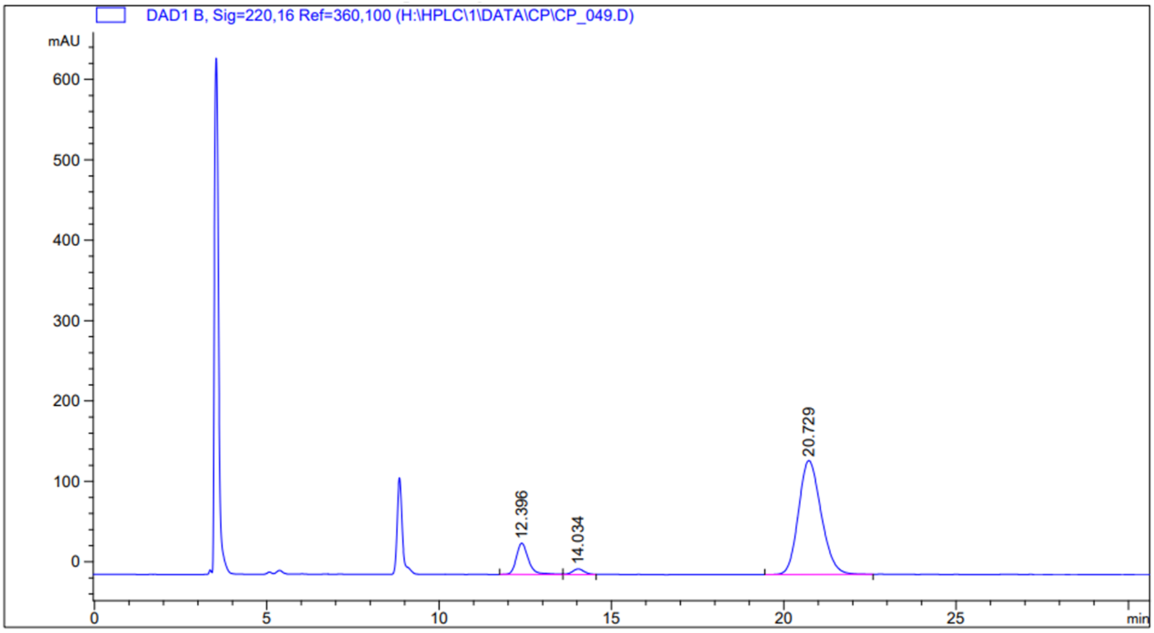

Peak #	RetTime [min]	Type	Width [min]	Area [mAU*s]	Height [mAU]	Area %
1	12.396	BV	0.3704	947.06860	38.85221	12.4846
2	14.034	VV	0.3643	161.21207	6.85589	2.1252
3	20.729	BV	0.7053	6477.62744	141.69710	85.3903

Figure S7 – HPLC chromatogram of **5a** + **5a'**obtained with **A** and **C** at r.t.

The two couples of enantiomers are peaks 1-3 and 2-4, confirmed also by analysis of UV spectra and by experimental ECD. From the kinetic experiments, it can be observed that, under heating, peak 1 converts into peak 4 thus showing that 1 and 4 are diastereoisomers possessing the chiral center with the same absolute configuration and the chiral axis with opposite absolute configuration. The same holds also for peak 2 and peak 3 which interconvert into one another upon heating. Moreover, it is known from literature[7, 10] that the starting trisubstituted hydrazide **3a**, obtained under the same conditions, predominantly has the (*S*) configuration at the chiral center. Therefore, the major product of the two-step sequence, peak 4, must have the (*S*) configuration at the chiral center and then also peak 1 must have the same absolute configuration at the chiral center.

[10] C. Liu, Q. Zhu, K.-W. Huang, Y. Lu *Org. Lett.* **2011**, *13*, 2638-2641.

Having established that peaks 1 and 4 are axial epimers, the enantiomer of 1 must be either peak 2 or peak 3. Chiral HPLC (Chiralpak IC column.: hexane/*i*-PrOH 98/2, flow rate 1 mL/min) with CD detection at 285 nm does not help since both peaks 2 and 3 have opposite CD sign with respect to that of peak 1 (Figure S8).

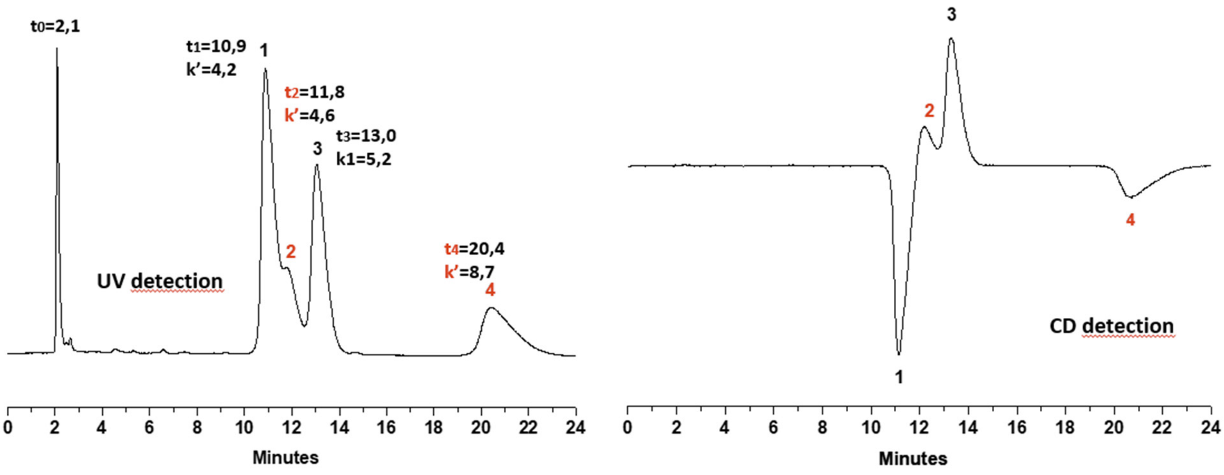

Figure S8: Chiral HPLC (Chiralpak I IC column.: hexane/*i*-PrOH 98/2, flow rate 1 mL/min) with CD detection at 285 nm.

The experimental ECD spectra of isolated peaks 1 and 3 show a nearly perfect inverted trend (below left) thus confirming that 1 (**5a'**) and 3 (*ent*-**5a'**) are enantiomers. The experimental ECD spectra of the two axial diastereoisomers [peak 1 (**5a'**) and peak 4 (**5a**), below right] are quite similar showing that the configuration at the chiral center is mainly responsible for the observed absorptions. However, it is possible to observe two main differences: a small peak around 250 nm present only in peak 4 (black circle), and a shoulder on the right at about 350 nm of only peak 1 (red circle) (Figure S9). Kinetic measurements in any case confirm that 1 and 4 are axial diastereoisomers.

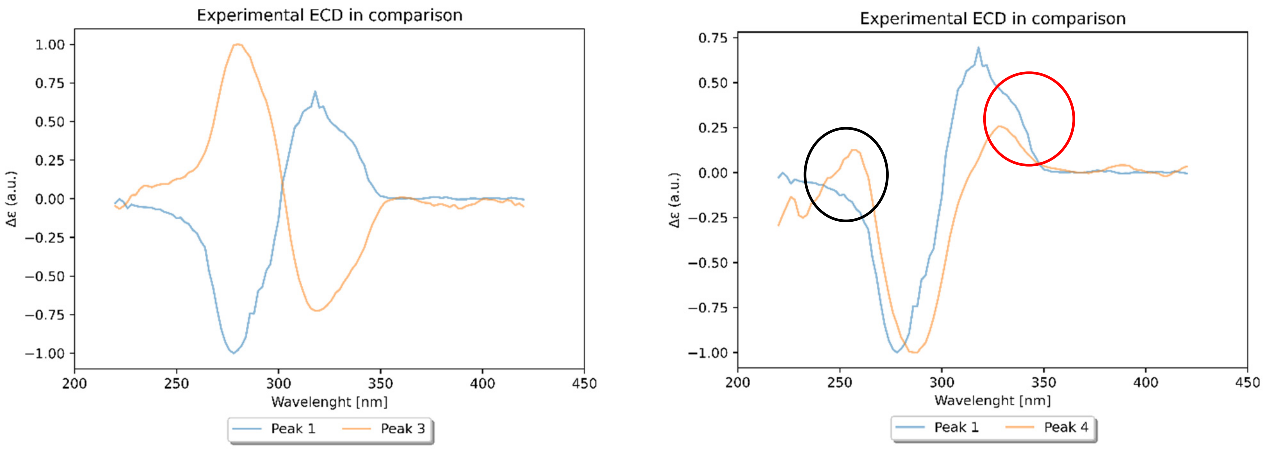

Figure S9. Experimental ECD comparison.

Determination of absolute configuration

A sample containing a mixture of **5a** and **5a'** in a d.r. of 1:6, obtained from the reaction of **3a** with **4a** using catalyst **D** (see reaction below), was dissolved in tert-butyl alcohol and let to slowly evaporate at r.t. Suitable crystals for X-Ray diffraction analysis were obtained.

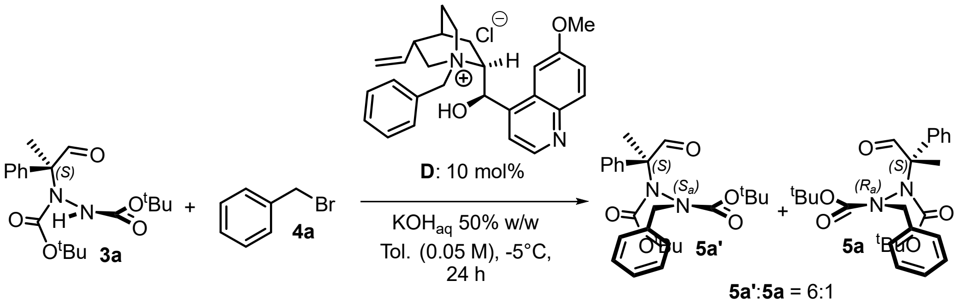

From X-Ray diffraction analysis the relative configuration of the crystalline compound was assigned as (S*,S_a*). The subsequent HPLC analysis on the analyzed crystal (IC column: hexane/i-PrOH 98/2, flow rate 1 mL/min, 25 °C, λ = 220 nm) associate the crystal to the first peak of the chromatogram. This peak is the axial-epimer of compound **5a** as revealed by kinetic epimerization studies.

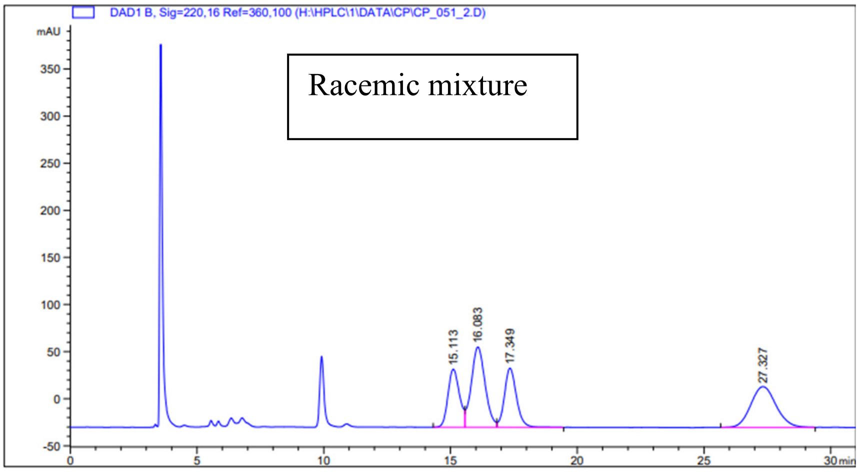

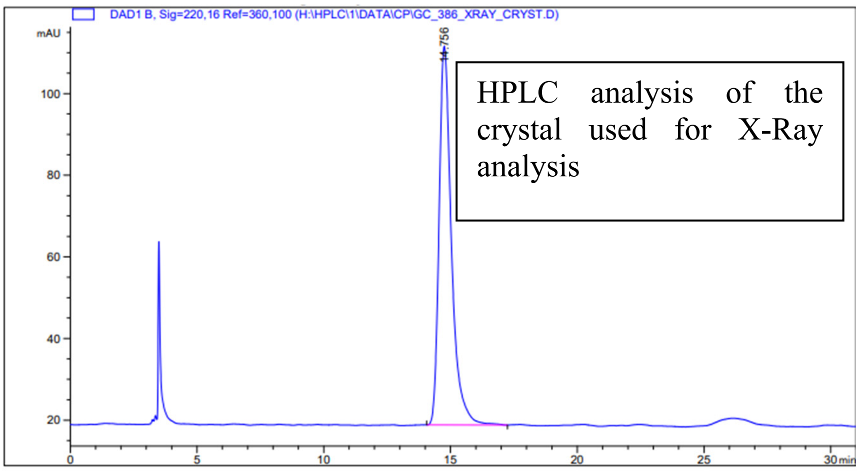

Because the absolute configuration of the quaternary stereocenter on **3a** is known to be (*S*) from literature,[7, 9] when hydrazide **3a** is prepared using catalyst **A**, the absolute configuration on compound **5a'** can be assigned to (S,S_a). Consequently, the absolute configuration of S,R_a can be assigned to **5a**.

Crystal data for compound **5a'**

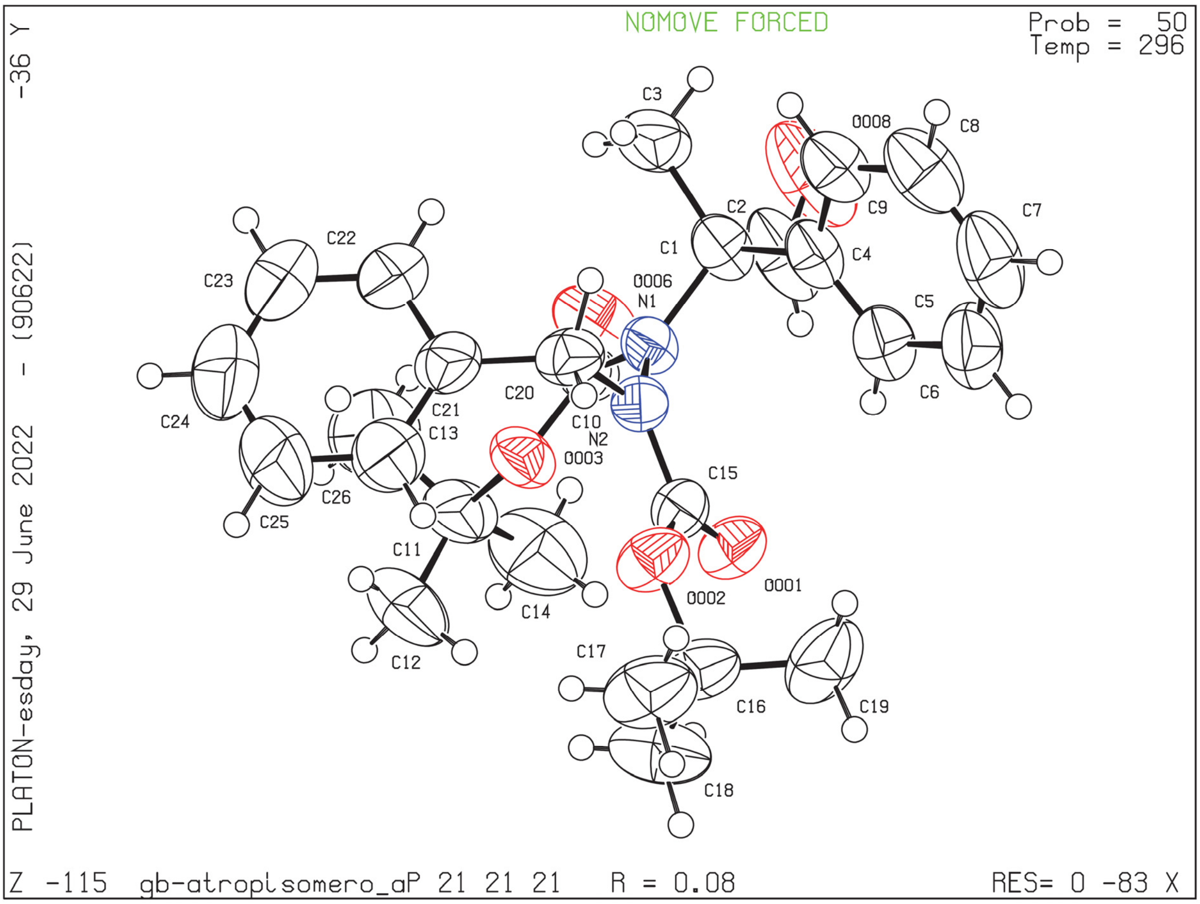

A specimen of $C_{26}H_{34}N_2O_5$ was used for the X-ray crystallographic analysis. The X-ray intensity data were measured (λ = 0.71073 Å). The total exposure time was 27.11 hours. The frames were integrated with the Bruker SAINT software package using a narrow-frame algorithm. The integration of the data using an orthorhombic unit cell yielded a total of 31101reflections to a maximum θ angle of 25.00° (0.84 Å resolution), of which 4455 were independent (average redundancy 6.981, completeness = 97.9%, R_{int} = 5.37%, R_{sig} = 3.90%) and 3740 (83.95%) were greater than 2σ(F^2). The final cell constants of a = 10.3138(8) Å, b = 12.6890(12) Å, c = 20.1061(18) Å, volume = 2631.3(4) $Å^3$, are based upon the refinement of the XYZ-centroids of 9929 reflections above 20 σ(I) with 5.097° < 2θ < 54.28°. Data were corrected for absorption effects using the Multi-Scan method (SADABS). The ratio of minimum to maximum apparent transmission was 0.924. The structure was solved and refined using the Bruker SHELXTL Software Package, using the space group P 21 21 21, with Z = 4 for the

formula unit, $C_{26}H_{34}N_2O_5$. The final anisotropic full-matrix least-squares refinement on F^2 with305 variables converged at R1 = 7.65%, for the observed data and wR2 = 14.59% for all data. The goodness-of-fit was 1.283. The largest peak in the final difference electron density synthesis was 0.220 $e^-/Å^3$ and the largest hole was -0.154 $e^-/Å^3$ with an RMS deviation of 0.029 $e^-/Å^3$. On the basis of the final model, the calculated density was 1.147 g/cm^3 and F(000), 976 e^-.

Table 1. Sample and crystal data

**Identification code**	gb2201	
**Chemical formula**	$C_{26}H_{34}N_2O_5$	
**Formula weight**	454.55 g/mol	
**Temperature**	296(2) K	
**Wavelength**	0.71073 Å	
**Crystal system**	orthorhombic	
**Space group**	P 21 21 21	
**Unit cell dimensions**	a = 10.3138(8) Å	α = 90°
	b = 12.6890(12) Å	β = 90°
	c = 20.1061(18) Å	γ = 90°
**Volume**	2631.3(4) $Å^3$	
**Z**	4	
**Density (calculated)**	1.147 g/cm^3	
**Absorption coefficient**	0.079 mm^{-1}	
**F(000)**	976	

Table 2. Data collection and structure refinement.

**Theta range for data collection**	1.90 to 25.00°	
**Index ranges**	-12<=h<=12, -14<=k<=15, -23<=l<=23	
**Reflections collected**	31101	
**Independent reflections**	4455 [R(int) = 0.0537]	
**Coverage of independent reflections**	97.9%	
**Absorption correction**	Multi-Scan	
**Structure solution technique**	direct methods	
**Structure solution program**	SHELXT 2014/5 (Sheldrick, 2014)	
**Refinement method**	Full-matrix least-squares on F^2	
**Refinement program**	SHELXL-2017/1 (Sheldrick, 2017)	
**Function minimized**	$\Sigma w(F_o^2 - F_c^2)^2$	
**Data / restraints / parameters**	4455 / 0 / 305	
**Goodness-of-fit on F^2**	1.283	
**Final R indices**	3740 data; I>2σ(I)	R1 = 0.0765, wR2 = 0.1416
	all data	R1 = 0.0970, wR2 = 0.1459

**Weighting scheme**	$w=1/[\sigma^2(F_o^2)+(0.0347P)^2+1.0412P]$ where $P=(F_o^2+2F_c^2)/3$
**Absolute structure parameter**	0.4(4)
**Largest diff. peak and hole**	0.220 and -0.154 eÅ^{-3}
**R.M.S. deviation from mean**	0.029 eÅ^{-3}

Table 3. Atomic coordinates and equivalent isotropic atomic displacement parameters (Å^2).

U(eq) is defined as one third of the trace of the orthogonalized U_{ij} tensor.

	x/a	y/b	z/c	U(eq)
O001	0.5134(3)	0.4471(3)	0.75160(18)	0.0637(9)
O002	0.6083(3)	0.2958(3)	0.78701(18)	0.0680(9)
O003	0.2892(3)	0.4072(3)	0.84172(15)	0.0666(9)
N1	0.2902(3)	0.3437(3)	0.73848(16)	0.0503(9)
N2	0.4037(3)	0.2916(3)	0.75475(17)	0.0493(9)
O006	0.1284(4)	0.4503(4)	0.77075(18)	0.0884(13)
C15	0.5108(5)	0.3542(4)	0.7638(2)	0.0527(11)
O008	0.0912(5)	0.4471(5)	0.6134(2)	0.129(2)
C4	0.3298(5)	0.2956(4)	0.6206(2)	0.0613(13)
C20	0.3942(5)	0.1802(4)	0.7730(2)	0.0632(13)
C9	0.3103(6)	0.2089(5)	0.5804(2)	0.0725(15)
C1	0.2293(5)	0.3279(4)	0.6721(2)	0.0616(13)
C10	0.2281(5)	0.4050(4)	0.7840(2)	0.0612(13)
C21	0.3342(5)	0.1613(4)	0.8402(3)	0.0700(15)
C5	0.4376(6)	0.3570(5)	0.6090(3)	0.0747(16)
C2	0.1857(7)	0.4349(6)	0.6483(3)	0.094(2)
C22	0.2046(6)	0.1393(5)	0.8475(3)	0.0926(19)
C11	0.2496(6)	0.4807(5)	0.8956(3)	0.0831(18)
C16	0.7403(5)	0.3408(5)	0.7922(3)	0.0759(15)
C7	0.5044(8)	0.2447(7)	0.5208(3)	0.103(2)
C3	0.1130(5)	0.2523(6)	0.6775(3)	0.0871(19)
C8	0.3987(8)	0.1837(6)	0.5307(3)	0.093(2)
C26	0.4070(7)	0.1658(6)	0.8974(3)	0.106(2)
C6	0.5247(6)	0.3309(6)	0.5592(3)	0.093(2)
C23	0.1485(8)	0.1222(7)	0.9087(4)	0.123(3)
C12	0.3542(8)	0.4591(7)	0.9468(3)	0.120(3)
C17	0.8168(6)	0.2485(6)	0.8182(4)	0.104(2)
C19	0.7849(6)	0.3751(6)	0.7239(3)	0.104(2)
C18	0.7379(7)	0.4312(6)	0.8419(4)	0.112(2)
C25	0.3508(10)	0.1495(8)	0.9593(4)	0.140(4)
C13	0.1188(8)	0.4485(9)	0.9208(4)	0.154(4)

	x/a	y/b	z/c	U(eq)
C24	0.2222(10)	0.1274(8)	0.9634(4)	0.149(4)
C14	0.2566(9)	0.5917(6)	0.8712(4)	0.125(3)

Table 4. Bond lengths (Å)

O001-C15	1.204(5)	O002-C15	1.334(6)
O002-C16	1.480(6)	O003-C10	1.320(5)
O003-C11	1.486(6)	N1-C10	1.362(6)
N1-N2	1.383(5)	N1-C1	1.489(6)
N2-C15	1.372(6)	N2-C20	1.464(6)
O006-C10	1.209(6)	O008-C2	1.211(7)
C4-C5	1.378(7)	C4-C9	1.379(7)
C4-C1	1.521(6)	C20-C21	1.505(7)
C20-H20A	0.97	C20-H20B	0.97
C9-C8	1.391(8)	C9-H9	0.93
C1-C2	1.507(8)	C1-C3	1.540(8)
C21-C22	1.373(8)	C21-C26	1.374(8)
C5-C6	1.384(8)	C5-H5	0.93
C2-H2	0.93	C22-C23	1.377(9)
C22-H22	0.93	C11-C14	1.493(9)
C11-C13	1.498(9)	C11-C12	1.517(9)
C16-C17	1.506(8)	C16-C19	1.512(8)
C16-C18	1.522(8)	C7-C8	1.351(10)
C7-C6	1.356(10)	C7-H7	0.93
C3-H3A	0.96	C3-H3B	0.96
C3-H3C	0.96	C8-H8	0.93
C26-C25	1.389(10)	C26-H26	0.93
C6-H6	0.93	C23-C24	1.339(11)
C23-H23	0.93	C12-H12A	0.96
C12-H12B	0.96	C12-H12C	0.96
C17-H17A	0.96	C17-H17B	0.96
C17-H17C	0.96	C19-H19A	0.96
C19-H19B	0.96	C19-H19C	0.96
C18-H18A	0.96	C18-H18B	0.96
C18-H18C	0.96	C25-C24	1.358(11)
C25-H25	0.93	C13-H13A	0.96
C13-H13B	0.96	C13-H13C	0.96
C24-H24	0.93	C14-H14A	0.96
C14-H14B	0.96	C14-H14C	0.96

Table 5. Bond angles (°).

C15-O002-C16	120.3(4)	C10-O003-C11	121.5(4)
C10-N1-N2	120.8(4)	C10-N1-C1	118.8(4)
N2-N1-C1	120.3(3)	C15-N2-N1	115.9(4)
C15-N2-C20	125.4(4)	N1-N2-C20	117.6(4)
O001-C15-O002	126.7(5)	O001-C15-N2	123.9(4)
O002-C15-N2	109.4(4)	C5-C4-C9	118.0(5)
C5-C4-C1	120.8(5)	C9-C4-C1	120.9(5)
N2-C20-C21	114.0(4)	N2-C20-H20A	108.8
C21-C20-H20A	108.8	N2-C20-H20B	108.8
C21-C20-H20B	108.8	H20A-C20-H20B	107.7
C4-C9-C8	120.5(6)	C4-C9-H9	119.7
C8-C9-H9	119.7	N1-C1-C2	106.8(4)
N1-C1-C4	111.0(4)	C2-C1-C4	103.3(4)
N1-C1-C3	110.4(4)	C2-C1-C3	110.5(5)
C4-C1-C3	114.2(4)	O006-C10-O003	126.2(5)
O006-C10-N1	121.6(4)	O003-C10-N1	112.3(4)
C22-C21-C26	116.8(6)	C22-C21-C20	122.0(5)
C26-C21-C20	121.2(5)	C4-C5-C6	120.7(6)
C4-C5-H5	119.6	C6-C5-H5	119.6
O008-C2-C1	122.7(7)	O008-C2-H2	118.7
C1-C2-H2	118.7	C21-C22-C23	122.4(6)
C21-C22-H22	118.8	C23-C22-H22	118.8
O003-C11-C14	109.9(5)	O003-C11-C13	108.8(5)
C14-C11-C13	114.3(7)	O003-C11-C12	100.8(5)
C14-C11-C12	111.0(6)	C13-C11-C12	111.2(6)
O002-C16-C17	101.9(4)	O002-C16-C19	109.1(5)
C17-C16-C19	112.3(5)	O002-C16-C18	108.8(5)
C17-C16-C18	111.5(5)	C19-C16-C18	112.6(6)
C8-C7-C6	120.2(6)	C8-C7-H7	119.9
C6-C7-H7	119.9	C1-C3-H3A	109.5
C1-C3-H3B	109.5	H3A-C3-H3B	109.5
C1-C3-H3C	109.5	H3A-C3-H3C	109.5
H3B-C3-H3C	109.5	C7-C8-C9	120.3(6)
C7-C8-H8	119.9	C9-C8-H8	119.9
C21-C26-C25	121.0(7)	C21-C26-H26	119.5
C25-C26-H26	119.5	C7-C6-C5	120.3(7)
C7-C6-H6	119.9	C5-C6-H6	119.9
C24-C23-C22	119.2(8)	C24-C23-H23	120.4
C22-C23-H23	120.4	C11-C12-H12A	109.5
C11-C12-H12B	109.5	H12A-C12-H12B	109.5

C11-C12-H12C	109.5	H12A-C12-H12C	109.5
H12B-C12-H12C	109.5	C16-C17-H17A	109.5
C16-C17-H17B	109.5	H17A-C17-H17B	109.5
C16-C17-H17C	109.5	H17A-C17-H17C	109.5
H17B-C17-H17C	109.5	C16-C19-H19A	109.5
C16-C19-H19B	109.5	H19A-C19-H19B	109.5
C16-C19-H19C	109.5	H19A-C19-H19C	109.5
H19B-C19-H19C	109.5	C16-C18-H18A	109.5
C16-C18-H18B	109.5	H18A-C18-H18B	109.5
C16-C18-H18C	109.5	H18A-C18-H18C	109.5
H18B-C18-H18C	109.5	C24-C25-C26	119.6(7)
C24-C25-H25	120.2	C26-C25-H25	120.2
C11-C13-H13A	109.5	C11-C13-H13B	109.5
H13A-C13-H13B	109.5	C11-C13-H13C	109.5
H13A-C13-H13C	109.5	H13B-C13-H13C	109.5
C23-C24-C25	121.0(8)	C23-C24-H24	119.5
C25-C24-H24	119.5	C11-C14-H14A	109.5
C11-C14-H14B	109.5	H14A-C14-H14B	109.5
C11-C14-H14C	109.5	H14A-C14-H14C	109.5
H14B-C14-H14C	109.5		

Table 6. Anisotropic atomic displacement parameters (Å^2)

The anisotropic atomic displacement factor exponent takes the form: $-2\pi^2[h^2 a^{*2} U_{11} + ... + 2 h k a^* b^* U_{12}]$

	U_{11}	U_{22}	U_{33}	U_{23}	U_{13}	U_{12}
O001	0.0559(19)	0.054(2)	0.081(2)	0.0068(19)	-0.0008(17)	-0.0033(17)
O002	0.0448(19)	0.065(2)	0.094(2)	0.0134(19)	-0.0063(18)	0.0031(18)
O003	0.061(2)	0.089(3)	0.0499(18)	-0.0156(17)	-0.0001(16)	0.002(2)
N1	0.041(2)	0.065(2)	0.0448(18)	-0.0062(18)	0.0026(16)	0.0060(18)
N2	0.043(2)	0.055(2)	0.0509(19)	0.0023(18)	-0.0018(17)	0.0015(19)
O006	0.059(2)	0.130(4)	0.076(2)	-0.033(2)	-0.0056(19)	0.036(2)
C15	0.047(3)	0.055(3)	0.056(3)	0.000(2)	0.006(2)	0.001(3)
O008	0.129(4)	0.177(5)	0.080(3)	0.002(3)	-0.023(3)	0.083(4)
C4	0.062(3)	0.078(4)	0.043(2)	0.003(3)	-0.002(2)	0.017(3)
C20	0.067(3)	0.050(3)	0.073(3)	-0.002(2)	-0.010(3)	-0.008(3)
C9	0.073(4)	0.088(4)	0.057(3)	-0.006(3)	-0.006(3)	0.019(3)
C1	0.051(3)	0.087(4)	0.047(2)	-0.005(2)	-0.001(2)	0.015(3)
C10	0.049(3)	0.083(4)	0.052(3)	-0.011(3)	0.003(2)	-0.005(3)
C21	0.074(4)	0.057(3)	0.079(3)	0.017(3)	-0.012(3)	-0.016(3)
C5	0.080(4)	0.086(4)	0.058(3)	0.004(3)	0.005(3)	0.009(3)
C2	0.097(5)	0.134(6)	0.050(3)	-0.001(3)	-0.003(3)	0.056(4)

	U11	U22	U33	U23	U13	U12
C22	0.090(5)	0.101(5)	0.088(4)	0.014(4)	-0.008(4)	-0.035(4)
C11	0.080(4)	0.106(5)	0.064(3)	-0.037(3)	0.007(3)	-0.011(4)
C16	0.044(3)	0.078(4)	0.105(4)	0.004(3)	-0.015(3)	0.001(3)
C7	0.099(5)	0.151(7)	0.057(4)	0.002(4)	0.021(4)	0.043(6)
C3	0.055(3)	0.139(6)	0.067(3)	-0.022(4)	0.000(3)	-0.008(4)
C8	0.107(5)	0.107(5)	0.064(3)	-0.022(3)	-0.003(4)	0.040(5)
C26	0.097(5)	0.126(6)	0.094(5)	0.039(4)	-0.027(4)	-0.025(4)
C6	0.082(4)	0.132(6)	0.067(4)	0.020(4)	0.018(3)	0.009(4)
C23	0.111(6)	0.146(7)	0.114(6)	0.032(5)	0.008(5)	-0.051(5)
C12	0.145(7)	0.156(7)	0.060(3)	-0.024(4)	-0.017(4)	-0.012(6)
C17	0.057(4)	0.111(5)	0.142(6)	0.014(5)	-0.024(4)	0.007(4)
C19	0.057(3)	0.124(6)	0.132(6)	0.024(5)	0.022(4)	-0.001(4)
C18	0.099(5)	0.109(5)	0.128(5)	-0.019(5)	-0.050(5)	0.000(5)
C25	0.134(7)	0.200(10)	0.087(5)	0.061(6)	-0.034(5)	-0.022(7)
C13	0.110(6)	0.239(11)	0.112(6)	-0.079(7)	0.061(5)	-0.036(7)
C24	0.151(9)	0.191(10)	0.105(6)	0.062(6)	0.000(6)	-0.052(8)
C14	0.162(8)	0.095(5)	0.118(6)	-0.036(4)	-0.011(6)	0.010(6)

Table 7. Hydrogen atomic coordinates and isotropic atomic displacement parameters (Å^2).

	x/a	y/b	z/c	U(eq)
H20A	0.4805	0.1497	0.7727	0.076
H20B	0.3432	0.1439	0.7397	0.076
H9	0.2373	0.1670	0.5866	0.087
H5	0.4519	0.4167	0.6348	0.09
H2	0.2332	0.4938	0.6610	0.113
H22	0.1527	0.1359	0.8097	0.111
H7	0.5633	0.2275	0.4875	0.123
H3A	0.1435	0.1825	0.6871	0.131
H3B	0.0664	0.2518	0.6361	0.131
H3C	0.0565	0.2755	0.7125	0.131
H8	0.3851	0.1247	0.5042	0.111
H26	0.4953	0.1799	0.8945	0.127
H6	0.5975	0.3727	0.5522	0.112
H23	0.0605	0.1072	0.9120	0.148
H12A	0.3496	0.3868	0.9606	0.18
H12B	0.3415	0.5042	0.9846	0.18
H12C	0.4378	0.4728	0.9277	0.18
H17A	0.7786	0.2239	0.8589	0.155
H17B	0.9046	0.2699	0.8264	0.155

	x/a	y/b	z/c	U(eq)
H17C	0.8160	0.1928	0.7860	0.155
H19A	0.7771	0.3172	0.6935	0.156
H19B	0.8738	0.3973	0.7261	0.156
H19C	0.7321	0.4327	0.7088	0.156
H18A	0.6895	0.4890	0.8237	0.168
H18B	0.8250	0.4537	0.8510	0.168
H18C	0.6977	0.4081	0.8824	0.168
H25	0.4009	0.1537	0.9977	0.168
H13A	0.0534	0.4706	0.8898	0.23
H13B	0.1033	0.4809	0.9632	0.23
H13C	0.1159	0.3732	0.9255	0.23
H24	0.1848	0.1158	1.0049	0.179
H14A	0.3388	0.6034	0.8501	0.188
H14B	0.2473	0.6391	0.9082	0.188
H14C	0.1881	0.6040	0.8399	0.188

Computational details

Structure of (*R,R$_a$*)-*ent*-**5a'** was subjected to conformational search, which was carried out using the recent CREST program.[11,12] at the GFN2-xTB[13,14] level of theory with the GBSA implicit solvation model. The conformational ensemble obtained from this search was visually inspected and it was noted that the ensemble contained members possessing an opposite axial configuration with respect to that of the input structure. Clearly, this axial rotation was induced by the metadynamic sampling performed by CREST. All the members of the ensemble presenting an inverted chiral axis were manually sorted out before the following step. Conformer ensemble obtained in the first step (457 conformers) was refined and sorted at the DFT level using the CENSO algorithm[15] interfaced with the ORCA quantum chemistry program package.[16] The refinement was performed in three parts with default thresholds. After each part, conformers lying outside the given threshold were discarded.

- Part0: cheap prescreening: single point energy at the B97-D3/def2-SV(P)//GFNn-xTB level

[11] P. Pracht, S. Grimme *Chem. Sci.*, **2021**, *12*, 6551–6568.

[12] P. Pracht , F. Bohle, S. Grimme *Phys. Chem. Chem. Phys.*, **2020**, *22* , 7169–7192

[13] C. Bannwarth, E. Caldeweyher, S. Ehlert, A. Hansen, P. Pracht, J. Seibert, S. Spicher, S. Grimme *WIREs Comput. Mol. Sci.*, **2020**, *11*, e01493.

[14] Bannwarth, C.; Ehlert, S.; Grimme, S. *J. Chem. Theory Comput.* **2019**, *15*, 1652–1671.

[15] Grimme, S.; Bohle, F.; Hansen, A.; Pracht, P.; Spicher, S.; Stahn, M. *J. Phys. Chem. A* **2021**, *125*, 4039–4054.

[16] Neese, F.; Wennmohs, F.; Becker, U.; Riplinger, C. *J. Chem. Phys.*, **2020**, *152*, 224108.

- Part1: prescreening: single point energy at the r2SCAN-3c/def2-mTZVPP/CPCM[toluene] + GmRRHO(GFN2[alpb]-bhess)//GFNn-xTB
- Part2: geometry optimization and free energy calculation at the r2SCAN -3c/def2-mTZVPP + CPCM[toluene] + GmRRHO(GFN2[alpb]-bhess) // r2SCAN -3c[SMD] /def2-mTZVPP

This gave final refined and optimized conformers ensembles (67 cumulative conformers). Part2 was also repeated at the ωb97x-d4/6-31g(d) + CPCM[hexane] + GmRRHO(GFN2[alpb]-bhess) // ωb97x-d4/6-31g(d)[SMD], with the aim to preoptimize the final ensemble for the following higher level DFT optimizations. Vibrational frequencies were computed at the same level with the default mRRHO approximation.[17]

Computation of the N–N rotational barriers of 5a and 3a

To determine the rotational barrier of **5a**, the lower energy conformer resulting from the conformational search already performed at the ωb97x-d4 level, has been used to explore the PES by using a relaxed scan about the N–N bond. This scan has been carried out at ωb97x-d4/3-21g + CPCM[toluene] level, taking 36 steps of 10°. Results are represented in Figure S10

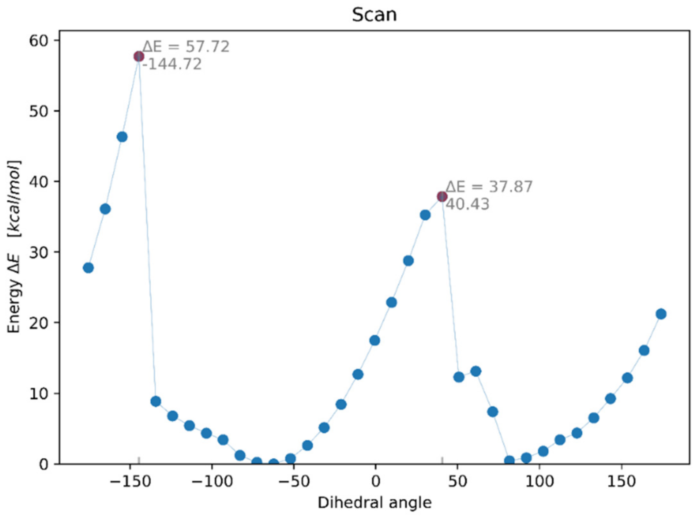

Figure S10. Clockwise N-N dihedral relaxed scan of (R,R_a)-*ent*-**5a'** at the ωB97x-d4/3-21g level.

[17] Grimme, S. Supramolecular Binding Thermodynamics by Dispersion-Corrected Density Functional Theory. *Chemistry – A European Journal* **2012**, *18* (32), 9955–9964.

Initial crude geometry of two TS - here called *cis* and *trans*, 40.34° and -144.72° respectively- have been pre-optimized by freezing the dihedral angle C-N-N-C (where carbon atoms are the Boc's ones), and then further optimized at the final ωB97x-d4/6-31g(d) level of theory, following the mode that represents the distortion on the bond. The same protocol has been followed to find the barrier of the tri-substituted hydrazide 3a. Vibrational frequency calculations were performed at the same level of theory to characterize all structures as either minima (no negative imaginary vibrational frequency) or saddle points (single negative imaginary frequency). Results for (R,Ra)-ent-5a' are shown in Table S9 and graphed in Figure S11, for molecule 3a in Table S10 and Figure S12.

Table S9. Optimized geometries[18] and rotational barriers [ΔG (kcal/mol)] calculated for (R,R_a)-*ent*-**5a'** at the ωb97x-d4/6-31g(d) CPCM[toluene] level of theory

(R,R_a)-*ent*-**5a'**	(R)-*cis*-TS-**5a**	(R,S_a)-*ent*-**5a**	(R)-*trans*-TS-**5a**
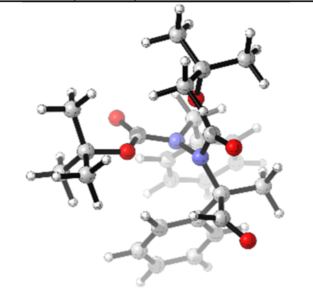	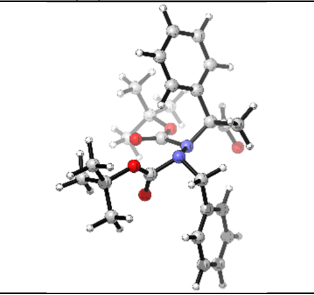	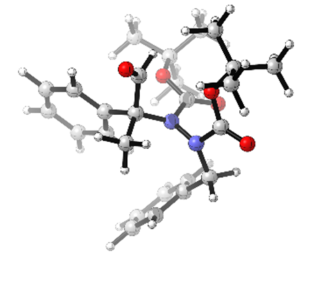	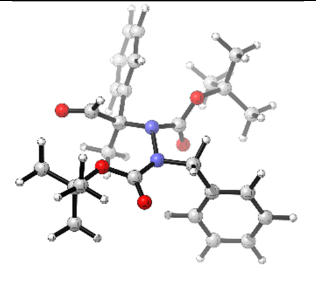
0.00	38.3	0.40	33.7

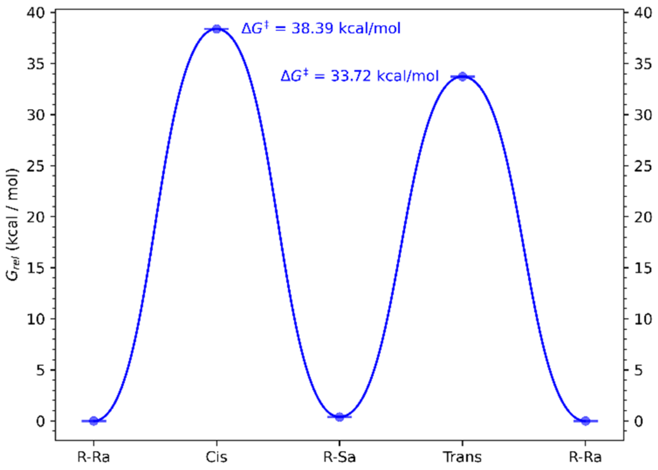

Figure S11. Rotational barriers of *ent*-**5a**.

The rotational barrier found is 33.7 kcal/mol, which is in fair agreement with the experimental one (28.32 kcal/mol). The energy separation between diastereomeric axial epimers of *ent*-**5a** was calculated to be 0.40 kcal/mol which corresponds to a Boltzmann population ratio of about **57:43 (*ent*-5a:*ent*-5a')**. This value is in very good agreement with the experimental value found during the kinetic equilibration experiments.

[18] CYLview, 1.0b; Legault, C. Y., Université de Sherbrooke, 2009 (http://www.cylview.org)

Table S10. Optimized geometries[18] and rotational barriers [ΔG (kcal/mol)] calculated for *ent*-**3a** at the ωb97x-d4/6-31g(d) CPCM[toluene] level of theory.

(R,R_a)-*ent*-**3a**	(R)-*cis*-TS-**3a**	(R,S_a)-*ent*-**3a**	(R)-*trans*-TS-**3a**
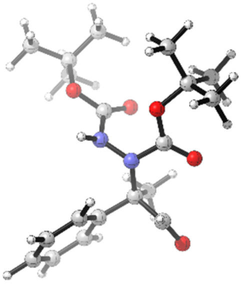	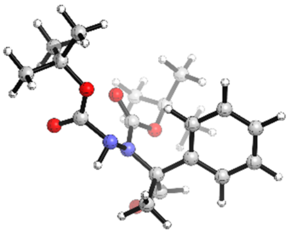	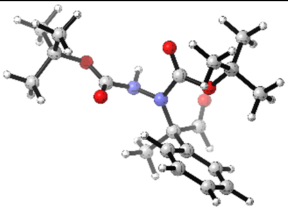	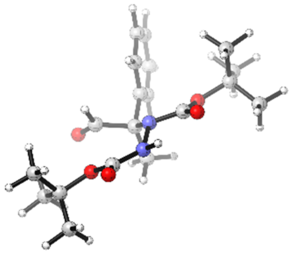
0.00	25.0	1.14	20.6

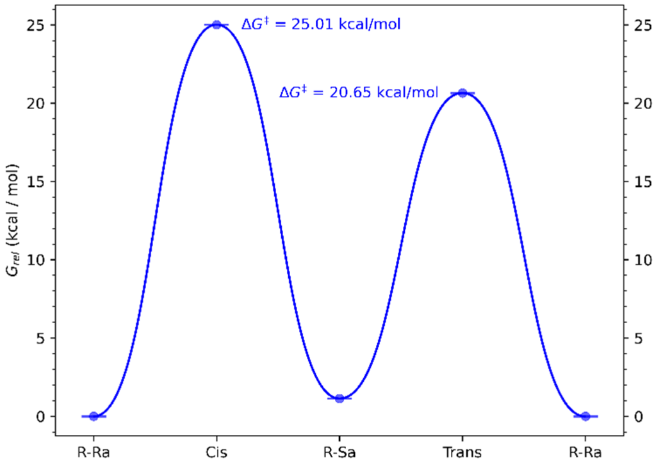

Figure S12. Rotational barrier of *ent*-**3a**.

The calculated rotational barrier of the trisubstituted hydrazide (R)-**3a** is of 20.6 kcal/mol, which corresponds to a half-life time of about two minutes at room temperature. This cannot be compared to any experimental value; however, it can be supported by two observations: the first is that the ^{1}H NMR spectrum shows two broadened peaks (Figure S13) for the aldehyde proton signal.

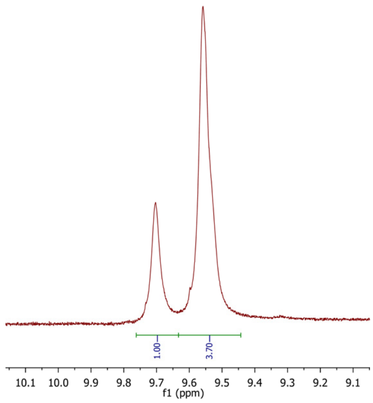

Figure S13. Broadened ^{1}H-NMR peaks attributed to the aldehyde's proton

(R,R_a)-*ent*-**5a'**

Zero Point Energy (Hartree)	:	0.5733011231
Inner Energy (Hartree)	:	-1496.9537316631
Enthalpy (Hartree)	:	-1496.9527874541
Rotational entropy	:	0.0171981631
Vibrational entropy	:	0.0533263995
Translational entropy	:	0.0171981631
Entropy	:	0.0915402877
Gibbs Energy (Hartree)	:	-1497.0443277418

N	-0.566527	0.791838	0.017796
N	-0.111998	-0.341807	-0.616904
C	-1.876725	1.184706	-0.168293
C	0.374478	1.756617	0.632045
C	-0.509027	-1.580106	-0.150541
C	0.365671	-0.226645	-1.999455
O	-2.327189	2.188345	0.363884
O	-0.246996	-2.607902	-0.754131
O	-2.530511	0.338880	-0.964272
C	-3.966314	0.509909	-1.222913
C	-4.745344	0.428049	0.088950
C	-4.205285	1.821247	-1.969419
C	-4.281413	-0.688703	-2.115535
H	-3.679062	-0.654388	-3.029982
H	-4.067069	-1.625967	-1.590548
H	-5.340804	-0.675986	-2.393116
H	-3.989270	2.683458	-1.335448
H	-3.573173	1.865315	-2.863937
H	-5.253238	1.869086	-2.286385
H	-4.543778	1.291031	0.726560
H	-5.818011	0.394579	-0.132713
H	-4.476373	-0.486826	0.629478
O	-1.180487	-1.484547	1.000537
C	-1.608268	-2.692184	1.715738
C	-2.631571	-3.456995	0.877719
C	-0.395438	-3.546669	2.085410
C	-2.262209	-2.113939	2.969525
H	0.339908	-2.945646	2.632308
H	-0.720082	-4.365095	2.737807
H	0.079708	-3.969951	1.198539
H	-2.643454	-2.925652	3.598164
H	-1.536504	-1.533864	3.550619
H	-3.096894	-1.457787	2.699018
H	-3.038857	-4.283107	1.471584
H	-3.458427	-2.794641	0.596734
H	-2.176343	-3.863493	-0.027407
C	-0.268243	2.260899	1.939044
C	0.635457	2.936784	-0.304561
O	-0.096866	3.374367	2.375196
H	-0.833698	1.489329	2.496948
H	1.219417	3.704854	0.207895
H	1.173754	2.610576	-1.198130
H	-0.318939	3.381578	-0.597791
C	2.674551	-0.727427	2.401569
C	1.535110	-0.076951	1.936927
C	1.641509	1.032945	1.089241
C	2.911103	1.489393	0.733351
C	4.052765	0.840258	1.201520
C	3.940382	-0.270219	2.033993
H	2.572458	-1.591684	3.054639
H	0.554873	-0.445372	2.221136
H	3.026574	2.343967	0.075943
H	5.033363	1.201700	0.902018
H	4.832385	-0.777409	2.394628
C	1.875525	-0.161433	-2.102691
H	-0.014303	-1.093533	-2.547424
H	-0.091587	0.671022	-2.426293
C	2.495370	0.857644	-2.826250
C	3.887494	0.914722	-2.924400
H	1.886767	1.615998	-3.317038
C	4.668548	-0.054134	-2.298673
H	5.753187	-0.011609	-2.370573
C	4.052093	-1.080849	-1.579371
H	4.357983	1.717143	-3.488541
C	2.665612	-1.135161	-1.482810
H	2.187578	-1.931740	-0.919337
H	4.656861	-1.838627	-1.086193

(R)-*cis*-TS-**5a**

Zero Point Energy (Hartree)	:	0.5735042962
Inner Energy (Hartree)	:	-1496.8953271407
Enthalpy (Hartree)	:	-1496.8943829316
Rotational entropy	:	0.0172875208
Vibrational entropy	:	0.0504665181
Translational entropy	:	0.0172875208
Entropy	:	0.0887697639
Gibbs Energy (Hartree)	:	-1496.9831526956
Imaginary frequency (cm^{-1})	:	-12.609945

N	-0.433124	-0.496758	0.885971
N	-1.103802	-0.707396	-0.382509
C	0.532892	0.524174	0.821292
C	-1.324173	-0.274211	2.080300
C	-0.184165	-0.532655	-1.506328
C	-1.604517	-2.106289	-0.584024
O	0.792497	1.179440	-0.168874
O	0.902841	-1.049429	-1.596811
O	1.100609	0.675417	2.019262
C	2.004544	1.809350	2.289764
C	3.276568	1.645514	1.460023
C	1.290984	3.134861	2.027885
C	2.301335	1.647354	3.778998
H	3.030436	2.402315	4.091485
H	1.389537	1.782037	4.371614
H	2.714779	0.653854	3.983484
H	1.944287	3.951310	2.356576
H	1.068442	3.270911	0.968249
H	0.357857	3.187006	2.598565
H	4.002742	2.408157	1.763322
H	3.717608	0.658119	1.636684
H	3.070675	1.757733	0.393884
O	-0.841280	0.134098	-2.450398
C	-0.224656	0.333885	-3.769720
C	-1.287959	1.142199	-4.510964
C	1.064335	1.142243	-3.625131
C	-0.001979	-1.017052	-4.450079
H	1.420305	1.428480	-4.621446
H	0.872384	2.052735	-3.047373
H	1.842245	0.564829	-3.122493
H	-0.938497	-1.587080	-4.471500
H	0.320317	-0.849687	-5.484117
H	0.761053	-1.603932	-3.935015
H	-0.947829	1.357577	-5.529535
H	-2.227738	0.581667	-4.566231
H	-1.475108	2.090577	-3.995578
C	-0.473463	-0.712112	3.301821
C	-2.576419	-1.161982	2.140316
O	-0.095386	-1.851523	3.446521
H	-0.276490	0.051913	4.070131
H	-3.268197	-0.918283	1.330584
H	-2.323499	-2.224396	2.139266
H	-3.086200	-0.940459	3.084310
C	-2.568202	3.044262	3.547042
C	-2.118957	1.728227	3.440451
C	-1.797805	1.179061	2.193338
C	-1.995311	1.958421	1.047813
C	-2.448932	3.272142	1.154813
C	-2.725078	3.826098	2.403682
H	-2.800631	3.454072	4.527343
H	-2.038561	1.134463	4.347845
H	-1.798960	1.540941	0.066273
H	-2.588946	3.863106	0.252527
H	-3.073326	4.853120	2.485047
C	-0.608940	-3.248802	-0.557612
H	-2.408505	-2.290622	0.120347
H	-2.076403	-2.060463	-1.571962
C	0.037164	-3.639304	0.619556
C	0.917020	-4.721057	0.611548
H	-0.127216	-3.092475	1.542655
C	1.153001	-5.433376	-0.564688
H	1.837209	-6.279181	-0.565009
C	0.504336	-5.055267	-1.739822
H	1.417613	-5.010993	1.533033
C	-0.368724	-3.968591	-1.731642
H	-0.868081	-3.671934	-2.652895

H	0.678800	-5.603660	-2.663057

(R,S_a)-*ent*-**5a**

Zero Point Energy (Hartree)	:	0.5736208847
Inner Energy (Hartree)	:	-1496.9529116618
Enthalpy (Hartree)	:	-1496.9519674528
Rotational entropy	:	0.0171658528
Vibrational entropy	:	0.0535404647
Translational entropy	:	0.0171658528
Entropy	:	0.0917220426
Gibbs Energy (Hartree)	:	-1497.0436894953

N	-0.041205	-0.178587	-0.384461
N	-0.079704	-0.984947	-1.506379
C	-0.314588	-0.841531	0.806718
C	-0.217308	1.286130	-0.575600
C	-1.306625	-1.453491	-1.945913
C	1.075144	-1.856039	-1.823861
O	-0.363839	-2.056561	0.881110
O	-1.396658	-2.313734	-2.802517
O	-0.478618	0.027971	1.801579
C	-0.466724	-0.402019	3.203375
C	-1.706635	-1.246932	3.489901
C	0.837942	-1.138538	3.505187
C	-0.518793	0.928835	3.951510
H	-0.517000	0.746164	5.031594
H	0.347963	1.546990	3.693268
H	-1.429646	1.479479	3.691465
H	0.908014	-1.316130	4.584201
H	0.881812	-2.100044	2.989015
H	1.693158	-0.525455	3.197811
H	-1.770860	-1.445988	4.565780
H	-2.608918	-0.703495	3.185348
H	-1.667133	-2.198119	2.955110
O	-2.314982	-0.816967	-1.336955
C	-3.711003	-1.136486	-1.672197
C	-4.503491	-0.271837	-0.692244
C	-3.988541	-0.718503	-3.114691
C	-3.987512	-2.618049	-1.415545
H	-5.055862	-0.842834	-3.329932
H	-3.727960	0.337016	-3.253997
H	-3.415449	-1.326679	-3.818194
H	-3.662779	-2.888243	-0.404018
H	-5.066021	-2.796903	-1.492050
H	-3.471711	-3.254569	-2.135798
H	-5.574762	-0.453971	-0.829211
H	-4.236121	-0.526672	0.340156
H	-4.304757	0.791105	-0.858704
C	-1.699047	1.628956	-0.342708
C	0.174450	1.685473	-2.000581
O	-2.319836	2.398178	-1.043954
H	-2.165235	1.145482	0.531393
H	1.214900	1.412091	-2.189568
H	-0.470669	1.215286	-2.746354
H	0.072433	2.769614	-2.090021
C	1.060976	4.112294	1.671109
C	0.249096	3.357119	0.822439
C	0.652394	2.088669	0.401301
C	1.889671	1.595889	0.821146
C	2.701670	2.349883	1.662942
C	2.287928	3.610446	2.099240
H	0.728990	5.096294	1.994535
H	-0.698945	3.773045	0.490122
H	2.213220	0.613663	0.501066
H	3.661855	1.946441	1.977980
H	2.920209	4.198260	2.760692
C	2.365528	-1.368225	-1.215024
H	0.854474	-2.869331	-1.472697
H	1.157848	-1.888987	-2.913379
C	2.778465	-1.839657	0.035190
C	3.973228	-1.394438	0.601125
H	2.152892	-2.551426	0.567549
C	4.770071	-0.474080	-0.081472
H	5.702501	-0.126779	0.357804
C	4.371152	-0.009367	-1.335212
H	4.282651	-1.768248	1.574735
C	3.178511	-0.460754	-1.899358
H	2.877494	-0.110251	-2.884828
H	4.992580	0.699240	-1.877589

(R)-*trans*-TS-**5a**

Zero Point Energy (Hartree)	:	0.5730411347
Inner Energy (Hartree)	:	-1496.9024024746
Enthalpy (Hartree)	:	-1496.9014582656
Rotational entropy	:	0.0173334465
Vibrational entropy	:	0.0507819683
Translational entropy	:	0.0173334465
Entropy	:	0.0891311398
Gibbs Energy (Hartree)	:	-1496.9905894054
Imaginary frequency (cm^{-1})	:	-5.464991

N	-0.716408	0.089342	0.446511
N	-0.584704	-0.996839	-0.514436
C	0.551240	0.086531	1.189590
C	-0.891752	1.478279	-0.090936
C	-1.390098	-1.225213	-1.610140
C	-0.108872	-2.241148	0.116564
O	1.635656	0.227483	0.678108
O	-1.333361	-2.300270	-2.189523
O	0.295530	-0.119615	2.471574
C	1.382848	-0.084045	3.468561
C	2.331551	-1.256506	3.226172
C	2.093561	1.268417	3.430802
C	0.621421	-0.257596	4.780654
H	1.325684	-0.260497	5.619374
H	-0.089587	0.563980	4.920307
H	0.070946	-1.204859	4.782855
H	2.787799	1.321183	4.277337
H	2.660710	1.400250	2.507340
H	1.369050	2.082955	3.526363
H	3.079362	-1.280293	4.026976
H	1.780204	-2.203258	3.244028
H	2.846606	-1.164197	2.267739
O	-2.165350	-0.198536	-1.980845
C	-2.950652	-0.326936	-3.233816
C	-3.646295	1.023711	-3.377770
C	-2.008343	-0.554245	-4.416780
C	-3.990585	-1.435665	-3.073003
H	-2.587129	-0.489248	-5.344983
H	-1.240905	0.228372	-4.439935
H	-1.525026	-1.530666	-4.370033
H	-4.586031	-1.258827	-2.169742
H	-4.665608	-1.416425	-3.936258
H	-3.524513	-2.419628	-3.009720
H	-4.245486	1.010474	-4.294764
H	-4.308731	1.222719	-2.530856
H	-2.918765	1.837362	-3.443971
C	-2.377995	1.729485	-0.386943
C	-0.019694	1.836072	-1.294793
O	-2.759685	2.691409	-1.012536
H	-3.075853	1.030409	0.110673
H	1.036731	1.857241	-1.023228
H	-0.157953	1.107983	-2.094854
H	-0.325253	2.815004	-1.672929
C	-1.325053	3.087542	3.357198
C	-1.427507	2.210798	2.281791
C	-0.685099	2.423997	1.110961
C	0.154050	3.536667	1.039106
C	0.258342	4.415388	2.118385
C	-0.480537	4.196175	3.278802
H	-1.906925	2.904371	4.257659
H	-2.070412	1.339152	2.359770
H	0.734214	3.732031	0.143672
H	0.920528	5.274980	2.046218
H	-0.400147	4.884063	4.117294
C	1.282967	-2.669640	-0.302417
H	-0.163044	-2.116070	1.201211
H	-0.814177	-3.035862	-0.138818
C	1.938259	-3.645162	0.453495
C	3.199897	-4.105375	0.079658
H	1.456445	-4.048686	1.343529
C	3.819693	-3.591435	-1.059861
H	4.804267	-3.947108	-1.355202

C	3.168199	-2.617793	-1.816862
H	3.699388	-4.863214	0.679371
C	1.905872	-2.159995	-1.440751
H	1.405354	-1.395471	-2.027126
H	3.644717	-2.211716	-2.706565

(*R*,*R*a)-*ent*-**3a**

Zero Point Energy (Hartree):	0.4618930274
Inner Energy (Hartree) :	-1226.5618766876
Enthalpy (Hartree) :	-1226.5609324786
Rotational entropy :	0.0168393109
Vibrational entropy :	0.0432792945
Translational entropy :	0.0168393109
Entropy :	0.0808213659
Gibbs Energy (Hartree) :	-1226.6417538445

N	0.612038	0.615167	0.172126
N	0.030999	-0.577121	0.550319
C	-0.175292	1.755416	0.089328
C	1.797973	0.527016	-0.702217
C	-1.090622	-1.019084	-0.130058
O	0.180422	2.730016	-0.553615
O	-1.465546	-0.558313	-1.190627
O	-1.278306	1.640453	0.824014
C	-2.333773	2.662229	0.738407
C	-1.821089	3.991316	1.291713
C	-2.827330	2.770127	-0.703963
C	-3.423459	2.086142	1.640491
H	-3.760053	1.114544	1.263086
H	-3.049321	1.957327	2.662125
H	-4.279958	2.768100	1.666003
H	-2.071740	3.219263	-1.351959
H	-3.078696	1.775517	-1.087830
H	-3.727697	3.394139	-0.729283
H	-2.655536	4.699335	1.352371
H	-1.416850	3.849813	2.300702
H	-1.044541	4.414418	0.651932
O	-1.638817	-2.023630	0.562031
C	-2.790741	-2.757285	0.021400
C	-3.048995	-3.812073	1.095945
C	-3.990717	-1.820717	-0.113813
C	-2.411409	-3.419383	-1.302827
H	-3.819789	-1.063451	-0.881699
H	-4.875215	-2.407589	-0.386492
H	-4.189390	-1.324251	0.843045
H	-2.231115	-2.676234	-2.082071
H	-1.510680	-4.030225	-1.173209
H	-3.228426	-4.075447	-1.623425
H	-3.900018	-4.436845	0.804691
H	-2.170406	-4.453941	1.221911
H	-3.277255	-3.336188	2.055953
C	1.421252	0.224974	-2.149789
C	2.560117	1.861466	-0.607951
O	3.195180	2.325037	-1.525505
C	3.936137	-1.298621	1.889088
C	2.998535	-0.450143	1.305971
C	2.778600	-0.469411	-0.077794
C	3.529357	-1.343463	-0.866154
C	4.468947	-2.194298	-0.282563
C	4.675758	-2.176196	1.095282
H	4.090248	-1.273193	2.965486
H	2.425618	0.229849	1.932743
H	3.387160	-1.371392	-1.941687
H	5.040926	-2.872449	-0.911771
H	5.408155	-2.840130	1.548645
H	2.580087	2.306683	0.405610
H	0.665142	0.941547	-2.478496
H	1.013202	-0.784836	-2.241557
H	2.295220	0.330379	-2.796692
H	0.103461	-0.816123	1.530408

(*R*)-cis-TS-**3a**

Zero Point Energy (Hartree):	0.4617151138
Inner Energy (Hartree) :	-1226.5238383438
Enthalpy (Hartree) :	-1226.5228941348
Rotational entropy :	0.0167435930
Vibrational entropy :	0.0415536106
Translational entropy :	0.0167435930
Entropy :	0.0789999641
Gibbs Energy (Hartree) :	-1226.6018940989
Imaginary frequency (cm^{-1}) :	-58.657851

N	0.084483	-1.034805	1.383626
N	-0.583171	-1.563009	0.219085
C	1.057937	-0.088738	1.152879
C	-0.805880	-0.857161	2.547825
C	0.323963	-1.784792	-0.909025
H	-0.805389	-2.518550	0.499385
O	1.566076	0.110841	0.061731
O	1.180441	-2.637779	-0.908493
O	1.360278	0.539515	2.294087
C	2.314415	1.657666	2.325477
C	3.704418	1.159073	1.937464
C	1.816042	2.787520	1.424859
C	2.270789	2.076622	3.793408
H	2.951002	2.918853	3.958369
H	1.256399	2.384312	4.071448
H	2.577583	1.246097	4.438815
H	2.459584	3.663611	1.563996
H	1.840936	2.498556	0.372207
H	0.790931	3.063220	1.696380
H	4.430548	1.965401	2.091998
H	3.990322	0.310672	2.569035
H	3.735412	0.850825	0.890418
O	-0.083687	-1.042045	-1.925236
C	0.613641	-1.103617	-3.219250
C	-0.137721	-0.062114	-4.046701
C	2.078986	-0.704304	-3.051816
C	0.450932	-2.497268	-3.826342
H	2.536636	-0.621019	-4.044306
H	2.151637	0.263355	-2.546549
H	2.630864	-1.445669	-2.470637
H	-0.609838	-2.770519	-3.867404
H	0.844228	-2.490794	-4.849230
H	0.992276	-3.248541	-3.247634
H	0.283917	-0.015735	-5.056492
H	-1.199038	-0.323512	-4.121380
H	-0.051510	0.927360	-3.584894
C	0.092292	-0.955742	3.809038
C	-1.790003	-2.036684	2.656331
O	0.749620	-1.939738	4.048961
H	0.050647	-0.096434	4.502321
H	-2.509987	-2.020448	1.834122
H	-1.241192	-2.984379	2.670783
H	-2.347462	-1.952055	3.594010
C	-2.952210	2.105207	3.648148
C	-2.241205	0.905447	3.654771
C	-1.574615	0.461761	2.507307
C	-1.657793	1.228561	1.341458
C	-2.368946	2.427947	1.334125
C	-3.013845	2.874481	2.487201
H	-3.458478	2.436249	4.552054
H	-2.215778	0.313932	4.568197
H	-1.180335	0.890801	0.426558
H	-2.420081	3.012942	0.418638
H	-3.566664	3.810929	2.478964

(*R*,*S*a)-*ent*-**3a**

Zero Point Energy (Hartree):	0.4616586453
Inner Energy (Hartree) :	-1226.5592501814
Enthalpy (Hartree) :	-1226.5583059724
Rotational entropy :	0.0167780746
Vibrational entropy :	0.0441492896
Translational entropy :	0.0167780746
Entropy :	0.0816301246
Gibbs Energy (Hartree) :	-1226.6399360970

N	0.093184	0.086623	-0.730045
N	-0.716205	-0.725340	-1.494050
C	-0.573634	1.104536	-0.063521
C	1.529998	0.112654	-1.019178
C	-1.359771	-1.750751	-0.811757
O	-1.787546	1.122456	0.040247

O	-0.973652	-2.208742	0.245700
O	0.289724	1.994772	0.420941
C	-0.139641	3.018311	1.379198
C	-1.105102	3.990746	0.702720
C	-0.743568	2.346822	2.611456
C	1.175891	3.708385	1.735644
H	1.641907	4.130286	0.838106
H	0.987126	4.520144	2.446674
H	1.872253	2.994072	2.187596
H	-0.933867	3.106106	3.378258
H	-1.684561	1.847165	2.371145
H	-0.040020	1.611712	3.018928
H	-2.052649	3.503576	0.464806
H	-1.297675	4.835657	1.373925
H	-0.661440	4.377218	-0.221969
O	-2.400674	-2.158572	-1.543029
C	-3.264684	-3.246254	-1.068772
C	-4.314434	-3.345248	-2.173969
C	-2.463627	-4.543997	-0.968423
C	-3.914042	-2.849086	0.256938
H	-3.146202	-5.371257	-0.743073
H	-1.969660	-4.756408	-1.923498
H	-1.709683	-4.484967	-0.180878
H	-4.399710	-1.871673	0.157869
H	-4.677609	-3.589743	0.520250
H	-3.177864	-2.799956	1.061591
H	-5.034701	-4.133392	-1.929898
H	-4.853269	-2.397041	-2.276859
H	-3.842634	-3.586844	-3.132720
C	1.948659	-1.200291	-1.712931
C	1.805757	1.231087	-2.029402
O	0.926508	1.891558	-2.534347
C	4.176752	1.086101	1.598600
C	3.459691	1.045820	0.400704
C	2.335397	0.228648	0.279092
C	1.942522	-0.557993	1.367448
C	2.655253	-0.512702	2.562617
C	3.776405	0.311300	2.684623
H	5.047669	1.732877	1.679574
H	3.788993	1.675505	-0.420938
H	1.061838	-1.189825	1.275906
H	2.333489	-1.124489	3.402624
H	4.332750	0.346622	3.618441
H	2.859711	1.353667	-2.343566
H	1.669715	-2.048657	-1.081211
H	3.036210	-1.199695	-1.838019
H	1.472970	-1.313411	-2.690986
H	-1.286231	-0.233918	-2.174800

(*R*)-trans-TS--**3a**

Zero Point Energy (Hartree)	:	0.4616502236
Inner Energy (Hartree)	:	-1226.5311051827
Enthalpy (Hartree)	:	-1226.5301609737
Rotational entropy	:	0.0167413496
Vibrational entropy	:	0.0412363540
Translational entropy	:	0.0167413496
Entropy	:	0.0786804639
Gibbs Energy (Hartree)	:	-1226.6088414376
Imaginary frequency (cm^{-1})	:	-36.116356

N	-0.491419	-0.068636	0.349666
N	-0.412398	-0.916346	-0.808172
C	0.597573	-0.414018	1.166411
C	-0.926547	1.334277	0.194012
C	-1.346867	-1.162875	-1.793421
H	0.006747	-1.786850	-0.497538
O	1.045885	-1.550314	1.170365
O	-1.415072	-2.272710	-2.293600
O	1.048762	0.604924	1.879007
C	1.982728	0.395000	2.998973
C	1.387569	-0.595558	3.998088
C	3.336014	-0.057454	2.454268
C	2.073606	1.790570	3.612151
H	2.772437	1.776383	4.455558
H	2.433796	2.512014	2.870416
H	1.091747	2.117475	3.968953
H	4.062206	-0.084735	3.274651
H	3.270579	-1.051140	2.006865
H	3.695170	0.651996	1.700190
H	2.026985	-0.628134	4.887248
H	0.388047	-0.266649	4.304015
H	1.323700	-1.601306	3.577390
O	-2.001164	-0.071185	-2.202284
C	-2.894306	-0.151700	-3.379953
C	-3.369804	1.286782	-3.573438
C	-2.094616	-0.602917	-4.603069
C	-4.072249	-1.071203	-3.063891
H	-2.727605	-0.505835	-5.492249
H	-1.218947	0.043144	-4.735700
H	-1.766696	-1.639068	-4.513875
H	-4.579005	-0.732069	-2.153074
H	-4.790483	-1.028586	-3.890688
H	-3.748212	-2.105379	-2.931888
H	-3.978755	1.339063	-4.482540
H	-3.967582	1.626873	-2.725425
H	-2.514596	1.961962	-3.686031
C	-2.406491	1.383721	-0.233262
C	-0.032041	2.109045	-0.768025
O	-2.900577	2.368923	-0.729637
H	-3.008413	0.511541	0.084276
H	0.950584	2.280855	-0.318181
H	0.090854	1.528333	-1.686411
H	-0.490085	3.065474	-1.029173
C	-2.026429	1.862740	3.824566
C	-1.752606	1.258671	2.600304
C	-1.115689	1.976371	1.580052
C	-0.795833	3.317004	1.792619
C	-1.074099	3.922368	3.017983
C	-1.686910	3.199325	4.039964
H	-2.511644	1.288227	4.610446
H	-2.013972	0.214882	2.444972
H	-0.319176	3.899435	1.010956
H	-0.809516	4.966206	3.170909
H	-1.904173	3.673498	4.994136

Synthesis of starting materials

Aldehydes 1b-j[1]

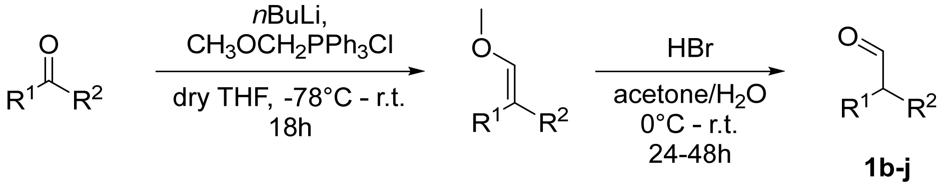

1b-j

Morita-Baylis-Hillman Reaction b[3]

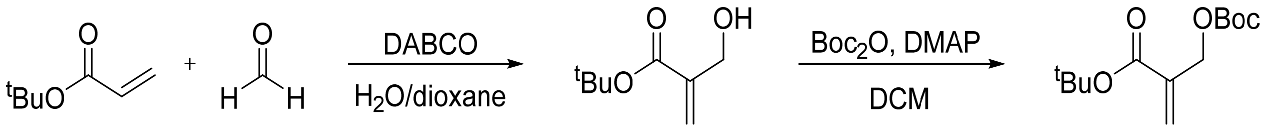

General procedure for the synthesis of 4b, 4c, 4d, 4i and 4m.[2]

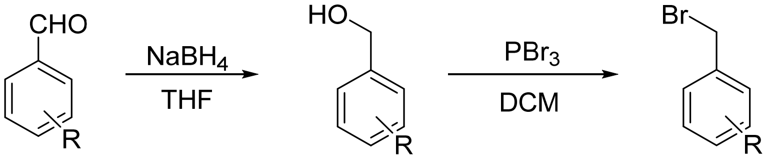

In a 10 mL round bottom flask equipped with a magnetic stirring bar 2 mmol of aldehyde, 4 mL of freshly distilled THF and 3 mmol of $NaBH_4$ were added. After three hours the reaction was quenched with a saturated solution of NH_4Cl and extracted three times with ethyl acetate. The organic fractions were collected, dried on Na_2SO_4, filtered, and concentrated with rotavapor. The crude product was pure enough to be used in the following step without further purification.

In a previously dried 10 mL two-necks round bottom flask, equipped with a magnetic stirring bar and flushed with nitrogen, the corresponding alcohol was added in DCM (0.5 M). After cooling the flask with an ice bath, 1.5 eq of PBr_3 were slowly added. The consumption of the starting compound was checked with TLC, then the reaction was stopped with the slow addition of distilled water. The two phases were separated, and the water phase was washed twice with fresh DCM. The organic phases were collected and washed twice with brine, dried on Na_2SO_4, filtered, and concentrated at low pressure. The crude product was purified through flash chromatography with a 9:1 hexane/diethyl ether mixture.

Experimental procedures

General procedure for the sequential catalysis one-pot reactions

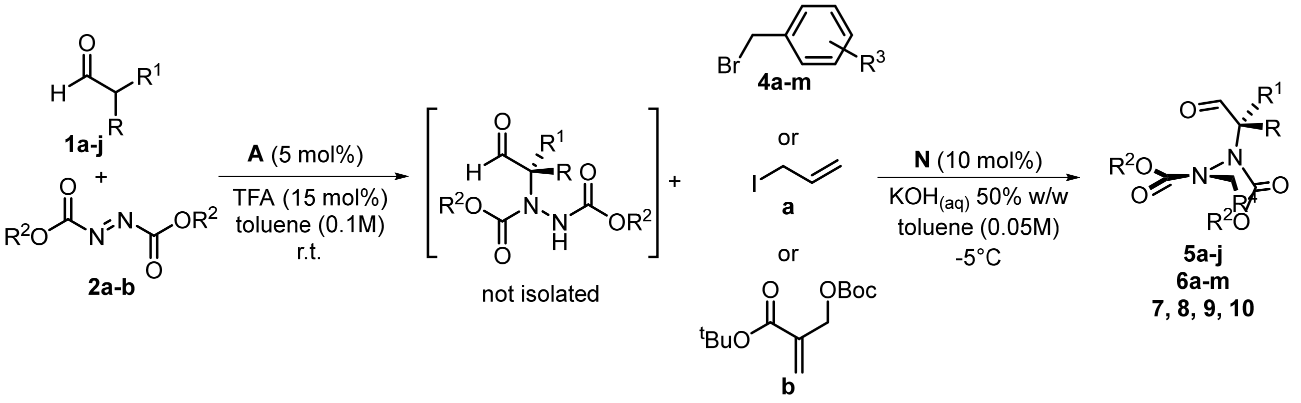

The reactions were carried out on a 0.3 mmol scale of aldehyde. To a 25 mL round bottom flask with a Teflon coated magnetic stirrer, 5% mol of catalyst **A** was added and dissolved in 3 mL of toluene; 0.3 mmol of aldehyde **1a-j**, TFA (15 mol%) and **2a-b** (1.1 eq) were added, and the solution was left stirring for 24h at 25 °C. An equal amount of toluene was added together with 12 mL of a solution of $KOH_{(aq)}$ (50% w/w), PTC catalyst **N** (10 mol%) and electrophile **4a-m** / **a** / **b** (1 eq). The flask was cooled to -5 °C and left stirring vigorously at this temperature. Completion of the reaction was checked by TLC using a solvent mixture of hexane:diethyl ether – 4:1. After the appropriate time, the two phases were separated and the water phase was washed twice with diethyl ether; the organic parts were collected, dried over Na_2SO_4, filtered and concentrated with rotavapor without heating the bath; eventual traces of toluene were removed by high vacuum pump. Column chromatography was used with the proper solvent mixture to isolate the product as a single spot containing both diastereoisomers. To determine the diastereomeric ratio and enantiomeric excess of the major diastereoisomer, in most cases reduction to alcohols was necessary as the four peaks could not be separated by HPLC.

General procedure for reduction reactions

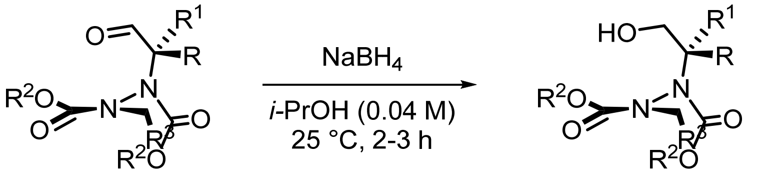

5a-j
6c, 6d, 6f, 6k, 6l
8, 10

5(a-j)r
6cr, 6dr, 6fr, 6kr, 6lr
8r, 10r

Reduction was performed with 1 eq of sodium borohydride in isopropanol (0.04 M) at r.t. After 2/3 hours, completion of the reaction was checked by TLC; a saturated solution of NH_4Cl was added and the solution was extracted with diethyl ether. Yield was quantitative in all cases. Crude mixture was directly analyzed through ^{1}H NMR and HPLC with chiral stationary phase.

The assignment of d.r. and e.e.% was done by identification of the enantiomeric couples by UV spectra.

Oxidation reaction - 5ao

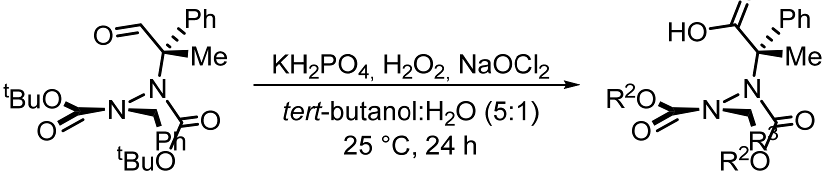

5a **5ao**

0.2 mmol of compound **5a** were dissolved in 3 mL of a mixture of *tert*-butanol:H_2O (5:1), then KH_2PO_4 (0.34 mmol, 46 mg), H_2O_2 (0.96 mmol, 0.1 mL of a 30% solution) and $NaOCl_2$ (0.69 mmol, 62 mg) were added. The solution was stirred at 25 °C for 24 hours, then water was added and EtOAc was used to extract. The organic parts were collected, dried over Na_2SO_4 and concentrated on the rotary evaporator. Crude ^{1}H NMR showed a mixture of diastereoisomers and complete conversion of the starting material. Column chromatography was used to get the clean final product **5ao**, that was obtained as a single diastereoisomer.

Three-step reaction process synthesis of 10ekr

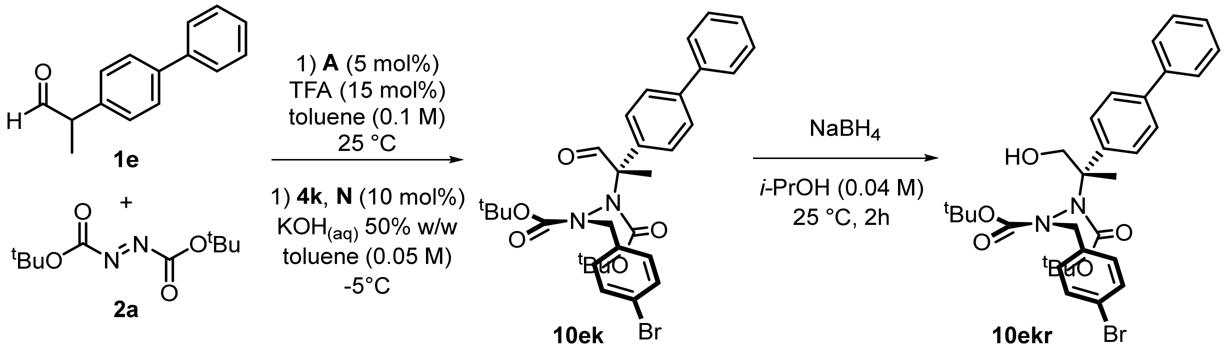

To a 25 mL round bottom flask with a Teflon coated magnetic stirrer, 5 mol % of catalyst **A** was added and dissolved in 3 mL of toluene; 0.3 mmol of aldehyde **1e**, TFA (15 mol %) and **2a** (1.1 eq) were added, and the solution was left stirring for 24h. An equal amount of toluene was added together with 12 mL of a solution of $KOH_{(aq)}$ (50% w/w), PTC catalyst **N** (10 mol%) and electrophile **4k** (1 eq). The flask was cooled to -5 °C and left stirring vigorously at this temperature. Completion of the reaction was checked by TLC using a solvent mixture of hexane:diethyl ether – 4:1. The two phases were separated and the water phase was washed twice with diethyl ether; the organic parts were collected, dried over Na_2SO_4, filtered and concentrated with rotavapor without heating the bath; eventual traces of toluene were removed by high vacuum pump. Reduction was then directly performed adding 1 eq of sodium borohydride in isopropanol (0.04 M) at r.t. After 2h, completion of the reaction was checked by TLC; a saturated solution of NH_4Cl was added and the solution was extracted with diethyl ether. Column chromatography was used to isolate the product as a single spot containing both diastereoisomers.

Large scale reaction for 5a and 6k

The reactions were carried out following the general procedure for the sequential catalysis one-pot reactions, using 3 mmol of aldehyde **1a** for compound **5a**, 2.5 mmol of aldehyde **1a** for compound **6k**.

Stereodivergent synthesis

Reactions were performed in a 0.2 mmol scale of **1a**, following general procedure. To obtain the four diastereoisomers different combinations of catalysts **A** and **N** and their correspondent pseudoenantiomers ***ent*-A** (9-*epi*-deoxy-amino-quinidine) and ***ent*-N** (catalyst **O**) were exploited. Regarding the amination step, catalyst ***ent*-A** gave the trisubstituted hydrazide (*R*)-**3a** with a 72% yield and 68% ee, hence showing a lower reactivity and enantioselectivity with respect to **A**.

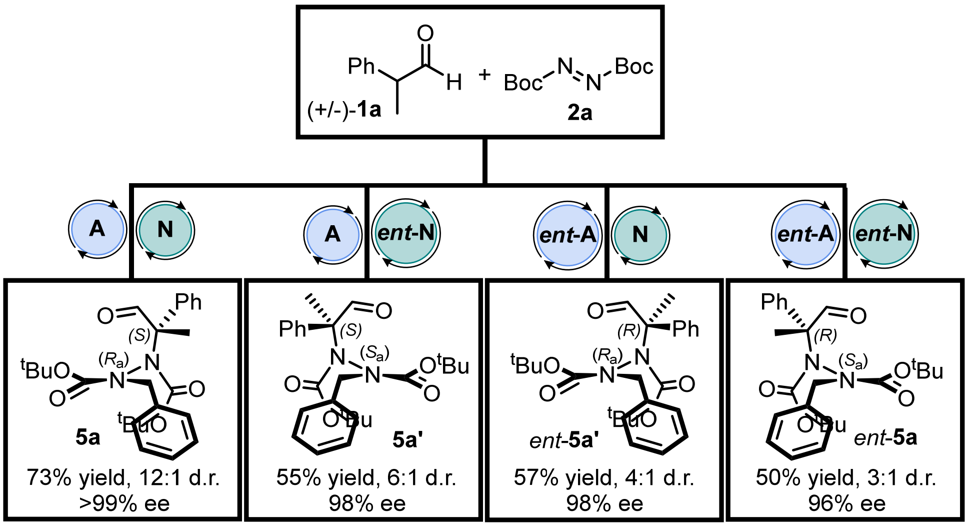

To better understand the diastereoselectivity, products **5a'**, ***ent*-5a'** and ***ent*-5a** were reduced following general procedure B.

Experimental details

(*S*, *R*a)-di-tert-butyl 1-benzyl-2-(1-oxo-2-phenylpropan-2-yl)hydrazine-1,2-dicarboxylate (5a)

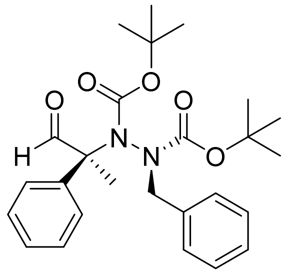

The reaction was carried out following the general procedure. Reaction time: 48h. The crude mixture was purified by flash column chromatography (hexane:Et_2O = 8:2). Yield= 73% (100 mg). Colorless oil. The d.r. and e.e. were determined by HPLC analysis on a Daicel Chiralpak IC column: hexane/*i*-PrOH 98/2, flow rate 1 mL/min, 25 °C, λ = 220 nm: t_1= 14 min, t_2= 15 min, t_3= 16 min, t_4= 26 min. d.r.= 13:1 and e.e. (major diastereoisomer) 99%. Peaks 1 and 3: minor diastereoisomer; peaks 2 and 4: major diastereoisomer.

HRMS-ESI-ORBITRAP (+): calculated for $[C_{26}H_{34}N_2NaO_5]^+$ 477.2360, found 477.2359 $[M+Na]^+$.

^{1}H NMR (400 MHz, $CDCl_3$) δ 9.93 – 9.61 (m, 1H), 7.50 – 6.89 (m, 10H), 5.17 – 4.13 (m, 2H), 1.83 – 1.08 (m, 21H).

^{13}C NMR (101 MHz, $CDCl_3$) δ 197.35, 196.25, 196.10, 195.94, 195.66, 155.49, 155.36, 155.10, 154.71, 154.39, 154.17, 152.79, 138.99, 138.84, 136.71, 136.59, 136.18, 135.89, 129.77, 129.17, 129.08, 128.49, 128.29, 128.25, 128.21, 128.18, 128.14, 127.87, 127.77, 127.72, 127.61, 127.50, 127.47, 127.45, 127.32, 127.29, 127.23, 126.81, 126.06, 82.59, 82.40, 82.32, 82.23, 82.13, 82.03, 81.94, 81.59, 72.78, 71.69, 71.54, 71.43, 56.41, 55.23, 54.44, 29.69, 29.65, 29.35, 28.34, 28.24, 28.14, 27.88, 27.82, 22.49, 20.27, 20.00, 19.69, 18.95, 18.42.

IR (ATR) ν(max)= 1735 (s) 1710 (s) 1681 (s) 1355 (s) 1150 (s) 1126 (s) cm^{-1}.

(*S*, *R*a)-di-tert-butyl 1-benzyl-2-(1-hydroxy-2-phenylpropan-2-yl)hydrazine-1,2-dicarboxylate (5ar)

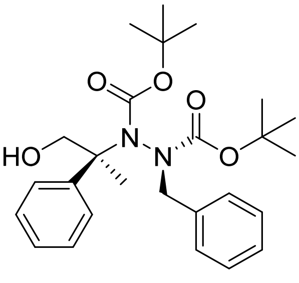

The reaction was carried out following the general reduction procedure. HPLC analysis were performed on a Daicel Chiralpak IC column: hexane/*i*-PrOH 98/2, flow rate 1 mL/min, 25 °C, λ =

220 nm: t_1= 14 min, t_2=18 min, t_3= 28 min, t_4= 43 min. The d.r. and e.e. values are consistent with those of **5a**. Peaks 1 and 2: couple of enantiomers; peaks 3 and 4: couple of enantiomers.

HRMS-ESI-ORBITRAP (+): calculated for $[C_{27}H_{36}N_2NaO_5]^+$ 479.2516, found 479.2509 $[M+Na]^+$.

^{1}H NMR (400 MHz, $CDCl_3$) δ 7.98 – 7.01 (m, 10H), 5.52 – 3.37 (m, 4H), 1.95 – 0.94 (m, 21H).

^{13}C NMR (101 MHz, $CDCl_3$) δ 159.08, 157.54, 156.15, 155.41, 153.69, 153.31, 146.16, 145.93, 144.25, 142.81, 137.70, 137.22, 137.00, 129.45, 128.83, 128.45, 128.28, 128.19, 127.47, 127.38, 127.27, 126.69, 126.61, 126.28, 125.37, 125.23, 125.03, 83.36, 83.00, 82.25, 81.73, 81.33, 70.33, 70.01, 69.63, 69.17, 66.90, 66.44, 58.65, 57.91, 55.79, 31.91, 30.32, 29.68, 28.34, 28.22, 27.87, 27.81, 26.77, 25.51, 25.21, 23.88.

IR (ATR) ν(max)= 3406 (br, m) 1694 (s) 1367 (s) 1152 (s) cm^{-1}.

(*S*, *R*a)-2-(2-benzyl-1,2-bis(tert-butoxycarbonyl)hydrazineyl)-2-phenylpropanoic acid (5ao)

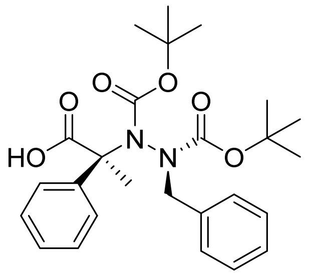

The reaction was carried out following the oxidation procedure. The crude mixture was purified by flash column chromatography (hexane:EtOAc = 2:1). Yield= 56% (53 mg). White solid. m.p. = 90-92 °C. D.r. and e.e. were determined by HPLC analysis on a Daicel Chiralpak IC column: hexane/*i*-PrOH 98/2, flow rate 1 mL/min, 25 °C, λ = 220 nm: t_1= 18 min, t_2= 20 min, t_3= 48 min, t_4= 54 min. d.r.> 20:1 and e.e. (major diastereoisomer) > 99%. Peaks 3 and 4: minor diastereoisomer; peaks 1 and 2: major diastereoisomer.

HRMS-ESI-ORBITRAP (+): calculated for $[C_{26}H_{34}N_2NaO_5]^+$ 493.2309, found 493.2304 $[M+Na]^+$.

^{1}H NMR (400 MHz, $CDCl_3$) δ 7.60 – 7.07 (m, 10H), 5.18 – 4.27 (m, 2H), 1.74 – 1.11 (m, 21H).

^{13}C NMR (101 MHz, $CDCl_3$) δ 172.59, 171.98, 160.28, 158.92, 155.22, 153.56, 152.86, 140.06, 139.47, 136.98, 136.02, 135.76, 135.64, 130.00, 129.81, 129.46, 128.75, 128.40, 128.33, 128.22, 128.07, 127.84, 127.61, 127.46, 127.42, 127.32, 127.17, 127.12, 126.08, 125.50, 85.45, 84.95, 83.09, 71.50, 70.63, 65.83, 57.04, 56.40, 53.77, 34.21, 30.31, 29.24, 28.56, 28.24, 28.15, 27.82, 27.80, 27.64, 25.84, 24.59, 15.25.

IR (ATR) ν(max)= 3662 (br, m) 3444 (br, m) 1704 (s) 1367 (s) 1151 (s) 1127 (s) cm^{-1}.

(*S*, *R*a)-di-tert-butyl 1-benzyl-2-(1-oxo-2-(p-tolyl)propan-2-yl)hydrazine-1,2-dicarboxylate (5b)

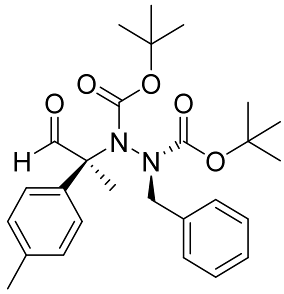

The reaction was carried out following the general procedure, at r.t. Reaction time: 32h. The crude mixture was purified by flash column chromatography (hexane:Et_2O = 8:2). Yield= 57% (53 mg). Colorless oil.

HRMS-ESI-ORBITRAP (+): calculated for $[C_{27}H_{36}N_2NaO_5]^+$ 491.2516, found 491.2515 $[M+Na]^+$.

^{1}H NMR (400 MHz, $CDCl_3$) δ 9.74 (d, *J* = 51.6 Hz, 1H), 7.35 – 6.88 (m, 9H), 5.13 – 4.01 (m, 2H), 2.47 – 2.23 (m, 3H), 1.82 – 1.13 (m, 21H).

^{13}C NMR (101 MHz, $CDCl_3$) δ 197.82, 197.28, 196.31, 196.03, 195.87, 155.52, 155.37, 155.07, 154.66, 143.85, 143.42, 138.14, 137.60, 137.43, 136.81, 136.64, 135.85, 135.74, 134.72, 130.50, 129.77, 129.22, 129.01, 128.58, 128.42, 128.24, 128.14, 128.06, 127.44, 127.34, 127.15, 127.12, 126.14, 83.32, 82.46, 82.27, 82.18, 82.05, 81.94, 72.57, 71.54, 71.29, 56.41, 54.95, 54.43, 30.31, 29.68, 28.34, 28.23, 27.85, 27.82, 27.79, 26.51, 22.12, 21.61, 20.99, 20.92, 19.92, 19.49, 18.85.

(*S*, *R*a)-di-tert-butyl 1-benzyl-2-(1-hydroxy-2-(p-tolyl)propan-2-yl)hydrazine-1,2-dicarboxylate (5br)

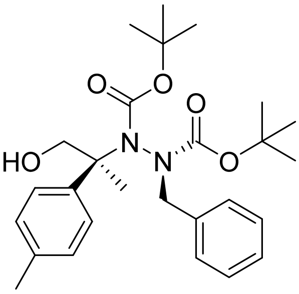

The reaction was carried out following the general reduction procedure. The dr and ee were determined by HPLC analysis on a Phenomenex Lux 5u Cellulose-2 column: hexane/*i*-PrOH 98/2, flow rate 1 mL/min, 25 °C, λ = 220 nm: t_1=17 min, t_2= 28 min, t_3= 30 min, t_4= 43 min. D.r.= 5:1 and e.e. (major diastereoisomer) >99%. Peaks 1 and 2: minor diastereoisomer; peaks 3 and 4: major diastereoisomer.

^{1}H NMR (400 MHz, $CDCl_3$) δ 7.63 – 6.90 (m, 9H), 5.36 – 3.34 (m, 4H), 2.32 (d, *J* = 7.3 Hz, 3H), 1.77 – 0.96 (m, 21H).

IR (ATR) ν(max)= 3406 (br, m) 1694 (s) 1367 (s) 1152 (s) 1067 (s) 1049 (s) cm^{-1}.

(*S*, *R*a)-di-tert-butyl 1-benzyl-2-(2-(4-(tert-butyl)phenyl)-1-oxopropan-2-yl)hydrazine-1,2-dicarboxylate (5c)

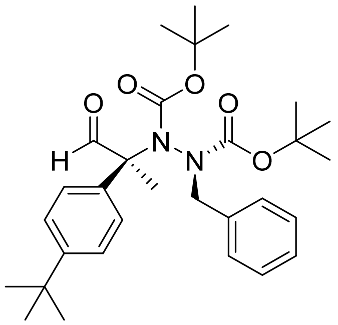

The reaction was carried out following the general procedure. Reaction time: 5 days. The crude mixture was purified by flash column chromatography (hexane:Et_2O = 8:2). Yield= 67% (104 mg). Colorless oil.

HRMS-ESI-ORBITRAP (+): calculated for $[C_{30}H_{42}N_2NaO_5]^+$ 533.2986, found 533.2984 $[M+Na]^+$.

^{1}H NMR (400 MHz, $CDCl_3$) δ 10.03 – 9.51 (m, 1H), 7.43 – 6.84 (m, 9H), 5.25 – 4.03 (m, 2H), 1.95 – 0.96 (m, 30H).

^{13}C NMR (101 MHz, $CDCl_3$) δ 197.15, 196.02, 195.63, 156.79, 155.52, 155.36, 155.15, 154.77, 153.18, 151.15, 150.69, 150.50, 136.85, 136.58, 135.66, 129.81, 129.13, 129.02, 128.96, 128.28, 128.09, 128.03, 127.43, 127.37, 127.31, 127.05, 125.98, 125.49, 125.22, 125.16, 124.99, 83.24, 82.28, 82.18, 81.98, 81.91, 81.40, 72.52, 71.52, 71.29, 71.18, 56.68, 56.43, 54.22, 34.45, 34.41, 31.30, 31.08, 29.69, 28.35, 28.23, 27.92, 27.81, 21.86, 19.68, 19.27, 18.56.

IR (ATR) ν(max)= 1697 (s) 1366 (s) 1150 (s) 1124 (s) cm^{-1}.

(*S*, *R*a)-di-tert-butyl 1-benzyl-2-(2-(4-(tert-butyl)phenyl)-1-hydroxypropan-2-yl)hydrazine-1,2-dicarboxylate (5cr)

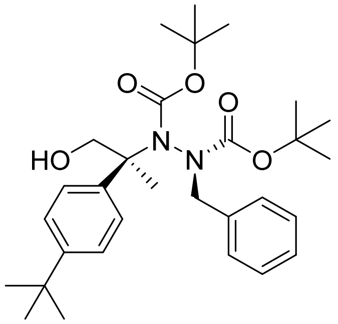

The reaction was carried out following the general reduction procedure. The d.r. and e.e. were determined by HPLC analysis on a Phenomenex Lux 5u Cellulose-2 column: hexane/*i*-PrOH 95/5, flow rate 1 mL/min, 25 °C, λ = 220 nm: t_1= 7 min, t_2= 9 min, t_3= 13 min, t_4= 15 min. D.r.= 10:1 and e.e. (major diastereoisomer) >99%. Peaks 1 and 3: minor diastereoisomer; peaks 2 and 4: major diastereoisomer.

^{1}H NMR (400 MHz, $CDCl_3$) δ 7.74 – 7.02 (m, 9H), 5.65 – 3.24 (m, 4H), 1.70 – 1.03 (m, 30H).

(*S*, *R*a)-di-tert-butyl 1-benzyl-2-(1-oxo-2-(5,6,7,8-tetrahydronaphthalen-2-yl)propan-2-yl)hydrazine-1,2-dicarboxylate (5d)

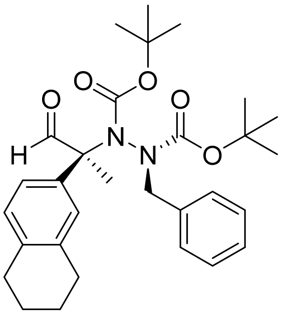

The reaction was carried out following the general procedure. Reaction time: 48h. The crude mixture was purified by flash column chromatography (hexane:Et_2O = 8:2).

Yield= 60% (92 mg). Colorless oil.

HRMS-ESI-ORBITRAP (+): calculated for $[C_{30}H_{40}N_2NaO_5]^+$ 531.2829, found 531.2827 $[M+Na]^+$.

^{1}H NMR (400 MHz, $CDCl_3$) δ 9.73 (d, *J* = 48.1 Hz, 1H), 7.46 – 6.80 (m, 8H), 5.15 – 4.03 (m, 2H), 2.93 – 2.50 (m, 4H), 1.78 (dh, *J* = 10.6, 4.6 Hz, 4H), 1.66 – 1.09 (m, 21H).

^{13}C NMR (101 MHz, $CDCl_3$) δ 196.28, 195.95, 156.78, 155.57, 155.45, 155.12, 154.68, 137.39, 136.90, 136.86, 136.72, 135.57, 129.70, 129.09, 128.80, 128.40, 128.08, 127.95, 127.78, 127.37, 127.21, 126.91, 124.29, 83.25, 82.20, 82.12, 82.01, 81.86, 71.64, 71.37, 56.57, 54.47, 29.62, 29.58, 28.98, 28.91, 28.34, 28.22, 27.84, 27.79, 23.15, 23.12, 19.94, 19.38.

(*S*, *R*a)-di-tert-butyl 1-benzyl-2-(1-hydroxy-2-(5,6,7,8-tetrahydronaphthalen-2-yl)propan-2-yl)hydrazine-1,2-dicarboxylate (5dr)

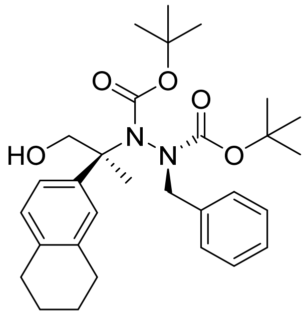

The reaction was carried out following the general reduction procedure.

The d.r. and e.e. were determined by HPLC analysis on a Daicel Chiralpak IC column.: hexane/*i*-PrOH 98/2, flow rate 1 mL/min, 25 °C, λ = 220 nm: t_1= 19 min, t_2= 21 min, t_3= 50 min, t_4= 55 min. D.r.= 13:1 and e.e. (major diastereoisomer) >99%. Peaks 1 and 2: minor diastereoisomer; peaks 3 and 4: major diastereoisomer.

^{1}H NMR (400 MHz, $CDCl_3$) δ 7.68 – 6.69 (m, 8H), 5.46 – 3.21 (m, 4H), 2.74 (q, *J* = 5.3 Hz, 4H), 1.80 (dh, *J* = 7.8, 4.3, 3.9 Hz, 4H), 1.70 – 1.15 (m, 21H). **IR** (ATR) ν(max)= 3407 (br, m) 1693 (s) 1367 (s) 1153 (s) 1058 (s) 1049 (s) cm^{-1}.

(*S*, *R*a)-di-tert-butyl 1-(2-([1,1'-biphenyl]-4-yl)-1-oxopropan-2-yl)-2-benzylhydrazine-1,2-dicarboxylate (5e)

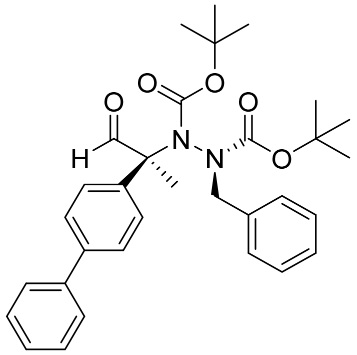

The reaction was carried out following the general procedure, at r.t. Reaction time: 32h. The crude mixture was purified by flash column chromatography (hexane:Et_2O = 6:1). Yield= 63% (100 mg). White solid.

HRMS-ESI-ORBITRAP (+): calculated for $[C_{32}H_{38}N_2NaO_5]^+$ 553.2673, found 553.2669 $[M+Na]^+$.

^{1}H NMR (400 MHz, $CDCl_3$) δ 10.08 – 9.60 (m, 1H), 7.66 – 7.11 (m, 14H), 5.61 – 3.98 (m, 2H), 1.93 – 1.11 (m, 21H).

^{13}C NMR (101 MHz, $CDCl_3$) δ 197.73, 197.45, 195.60, 155.42, 154.75, 145.77, 145.49, 140.64, 139.87, 139.59, 137.78, 136.58, 135.85, 135.57, 129.70, 129.42, 128.94, 128.90, 128.76, 128.66, 128.62, 128.48, 128.21, 128.19, 127.93, 127.65, 127.37, 127.26, 127.21, 127.11, 126.98, 126.96, 126.93, 126.83, 126.68, 82.53, 82.20, 71.31, 54.60, 29.68, 28.36, 28.25, 28.08, 27.85, 27.58, 26.65, 26.37, 19.99. **IR** (ATR) ν(max)= 1699 (s) 1679 (s) 1367 (s) 1151 (s) cm^{-1}.

(*S*, *R*a)-di-tert-butyl 1-(2-([1,1'-biphenyl]-4-yl)-1-hydroxypropan-2-yl)-2-benzylhydrazine-1,2-dicarboxylate (5er)

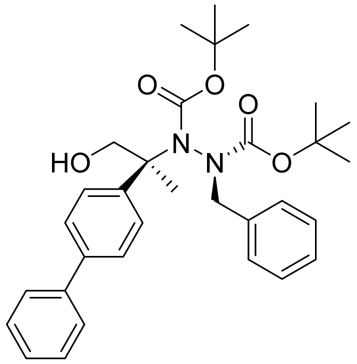

The reaction was carried out following the general reduction procedure. The d.r. and e.e. were determined by HPLC analysis on a Daicel Chiralpak IC column.: hexane/*i*-PrOH 98/2, flow rate 1 mL/min, 25 °C, λ = 220 nm: t_1= 19 min, t_2= 21 min, t_3= 38 min, t_4= 42 min. D.r.= 5:1 and e.e.(major diastereoisomer)= 99%. Peaks 1 and 2: minor diastereoisomer; peaks 3 and 4: major diastereoisomer.
^{1}H NMR (400 MHz, $CDCl_3$) δ 8.02 – 6.92 (m, 14H), 5.50 – 3.31 (m, 4H), 1.93 – 0.99 (m, 21H).

(*S*, *R*a)-di-tert-butyl 1-benzyl-2-(2-(naphthalen-2-yl)-1-oxopropan-2-yl)hydrazine-1,2-dicarboxylate (5f)

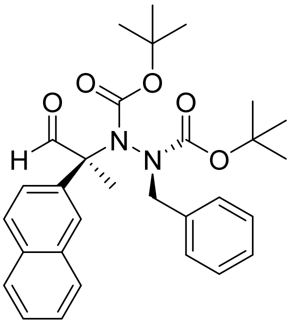

The reaction was carried out following the general procedure. Reaction time: 5 days. The crude mixture was purified by flash column chromatography (hexane:Et_2O = 7:3). Yield= 72% (109 mg). Colorless oil.
HRMS-ESI-ORBITRAP (+): calculated for $[C_{30}H_{36}N_2NaO_5]^+$ 527.2516, found 527.2513 $[M+Na]^+$.
^{1}H NMR (400 MHz, $CDCl_3$) δ 10.11 – 9.64 (m, 1H), 8.18 – 6.92 (m, 12H), 5.16 – 4.09 (m, 2H), 2.00 – 0.99 (m, 21H).
^{13}C NMR (101 MHz, $CDCl_3$) δ 198.09, 197.52, 196.38, 195.72, 155.58, 155.47, 155.16, 154.79, 136.58, 136.39, 135.59, 134.50, 132.91, 132.81, 132.52, 132.39, 130.19, 129.70, 129.54, 129.30, 129.06, 128.82, 128.46, 128.42, 128.38, 128.13, 128.05, 127.95, 127.78, 127.66, 127.50, 127.41, 127.38, 126.77, 126.31, 126.28, 126.19, 126.07, 125.96, 125.29, 125.05, 124.30, 123.90, 83.48, 82.52, 82.38, 82.21, 82.09, 72.85, 71.77, 71.54, 56.36, 54.62, 29.69, 28.37, 28.27, 27.92, 27.86, 27.75, 26.68, 22.56, 20.05, 19.82.
IR (ATR) ν(max)= 1734 (s) 1710 (s) 1694 (s) 1367 (s) 1150 (s) cm^{-1}.

(*S*, *R*a)-di-tert-butyl 1-benzyl-2-(1-hydroxy-2-(naphthalen-2-yl)propan-2-yl)hydrazine-1,2-dicarboxylate (5fr)

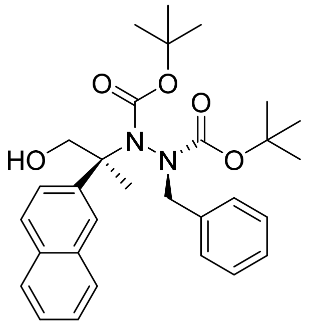

The reaction was carried out following the general reduction procedure. The dr and ee were determined by HPLC analysis on a Daicel Chiralpak IC column.: hexane/*i*-PrOH 98/2, flow rate 1 mL/min, 25 °C, λ = 220 nm: t_1= 17 min, t_2= 18 min, t_3= 37 min, t_4= 43 min. D.r.= 11:1 and e.e.(major diastereoisomer)= >99%. Peaks 1 and 2: minor diastereoisomer; peaks 3 and 4: major diastereoisomer.

^{1}H NMR (400 MHz, $CDCl_3$) δ 8.18 – 6.99 (m, 12H), 5.51 – 3.41 (m, 4H), 1.88 – 0.63 (m, 21H).

(*S*, *R*a)-di-tert-butyl 1-benzyl-2-(2-(4-methoxyphenyl)-1-oxopropan-2-yl)hydrazine-1,2-dicarboxylate (5g)

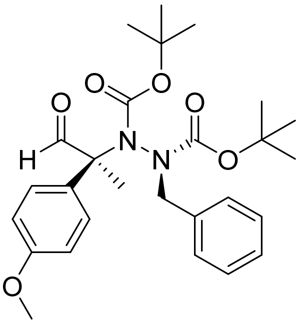

The reaction was carried out following the general procedure. Reaction time: 72h. The crude mixture was purified by flash column chromatography (hexane:Et_2O = 8:2). Yield= 65% (94 mg). Colorless oil.

HRMS-ESI-ORBITRAP (+): calculated for $[C_{27}H_{36}N_2NaO_6]^+$ 507.2466, found 507.2467 $[M+Na]^+$.

^{1}H NMR (400 MHz, $CDCl_3$) δ 9.71 (d, *J* = 51.0 Hz, 1H), 7.41 – 6.66 (m, 9H), 5.12 – 4.08 (m, 2H), 3.88 – 3.69 (m, 3H), 1.86 – 1.03 (m, 21H).

^{13}C NMR (101 MHz, $CDCl_3$) δ 197.07, 196.73, 196.19, 195.70, 163.47, 159.47, 159.14, 159.05, 158.80, 155.48, 155.36, 155.05, 154.68, 136.76, 136.58, 131.47, 130.77, 130.57, 130.33, 129.72, 129.14, 128.99, 128.66, 128.46, 128.13, 128.07, 127.63, 127.44, 127.35, 113.66, 113.64, 113.35, 83.37, 82.42, 82.28, 82.16, 82.00, 81.92, 81.55, 72.25, 71.21, 71.08, 71.00, 70.89, 56.36, 55.44, 55.24, 54.39, 30.30, 29.67, 28.33, 28.22, 28.15, 27.87, 27.82, 27.80, 26.31, 21.89, 19.61, 19.24.

(*S*, *R*a)-di-tert-butyl 1-benzyl-2-(1-hydroxy-2-(4-methoxyphenyl)propan-2-yl)hydrazine-1,2-dicarboxylate (5gr)

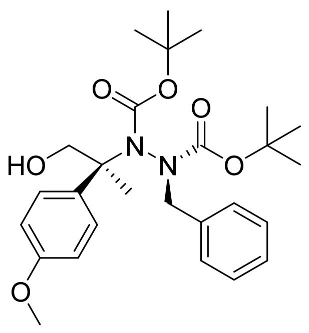

The reaction was carried out following the general reduction procedure. The d.r. and e.e. were determined by HPLC analysis on a Daicel Chiralpak IC column.: hexane/*i*-PrOH 98/2, flow rate 1 mL/min, 25 °C, λ = 220 nm: t_1= 27 min, t_2= 30 min, t_3= 51 min, t_4= 62 min. D.r.= 11:1 and e.e.(major diastereoisomer)= >99%. Peaks 1 and 2: minor diastereoisomer; peaks 3 and 4: major diastereoisomer.

^{1}H NMR (400 MHz, $CDCl_3$) δ 7.82 – 6.64 (m, 9H), 5.36 – 3.30 (m, 7H), 1.89 – 0.99 (m, 21H).

IR (ATR) ν(max)= 3406 (br, m) 1694 (s) 1367 (s) 1249 (s) 1152 (s) 1067 (s) 1032 (s) cm^{-1}.

(*S*, *R*a)-di-tert-butyl 1-benzyl-2-(2-(3-(benzyloxy)phenyl)-1-oxopropan-2-yl)hydrazine-1,2-dicarboxylate (5h)

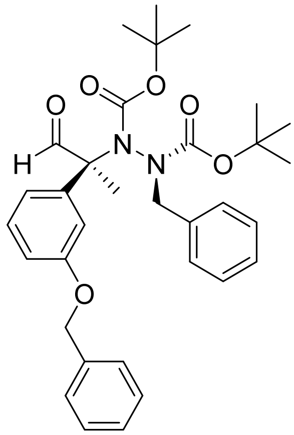

The reaction was carried out following the general procedure. Reaction time: 4 days. The crude mixture was purified by flash column chromatography (hexane:Et_2O = 7:3). Yield= 50% (84 mg). Colorless oil.

HRMS-ESI-ORBITRAP (+): calculated for $[C_{27}H_{36}N_2NaO_5]^+$ 583.2779, found 583.2772 $[M+Na]^+$.

^{1}H NMR (400 MHz, $CDCl_3$) δ 9.74 (d, *J* = 51.7 Hz, 1H), 7.66 – 6.77 (m, 14H), 5.01 (d, *J* = 4.5 Hz, 2H), 4.67 – 4.06 (m, 2H), 1.80 – 1.21 (m, 21H).

^{13}C NMR (101 MHz, $CDCl_3$) δ 197.16, 195.81, 195.30, 158.76, 156.71, 155.52, 155.34, 155.11, 154.77, 140.59, 137.00, 136.97, 136.75, 136.57, 129.75, 129.63, 129.28, 128.97, 128.56, 128.15,

128.08, 127.95, 127.47, 127.38, 121.31, 120.27, 119.86, 118.75, 114.45, 114.34, 114.15, 113.70, 113.58, 113.12, 83.41, 82.43, 82.32, 82.11, 82.05, 72.67, 71.63, 71.32, 70.18, 69.95, 56.42, 54.38, 29.69, 28.34, 28.24, 27.82, 26.71, 22.39, 19.84, 19.51.

IR (ATR) ν(max)= 1696 (s) 1367 (s) 1149 (s) 1056 (s) 1027 (s) cm^{-1}.

(*S*, *R*a)-di-tert-butyl 1-benzyl-2-(2-(3-(benzyloxy)phenyl)-1-hydroxypropan-2-yl)hydrazine-1,2-dicarboxylate (5hr)

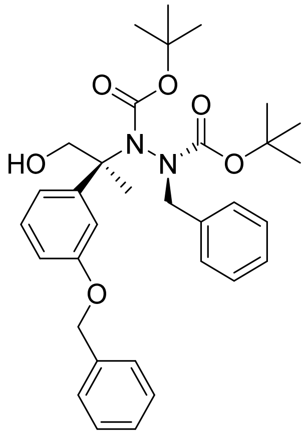

The reaction was carried out following the general reduction procedure. The d.r. and e.e. were determined by HPLC analysis on a Daicel Chiralpak IC column.: hexane/*i*-PrOH 98/2, flow rate 1 mL/min, 25 °C, λ = 220 nm: t_1= 20 min, t_2= 23 min, t_3= 46 min, t_4= 58 min. D.r.= 12:1 and e.e.(major diastereoisomer)= >99%. Peaks 1 and 2: minor diastereoisomer; peaks 3 and 4: major diastereoisomer.

^{1}H NMR (400 MHz, $CDCl_3$) δ 7.56 – 6.72 (m, 14H), 5.02 (d, *J* = 6.5 Hz, 2H), 4.82 – 3.78 (m, 4H), 1.81 – 0.71 (m, 21H).

(*S*, *R*a)-di-tert-butyl 1-benzyl-2-(1-oxo-2-(thiophen-2-yl)propan-2-yl)hydrazine-1,2-dicarboxylate (5i)

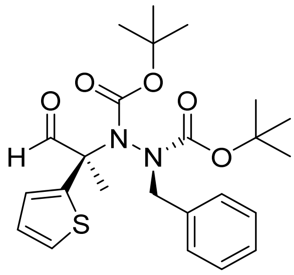

The reaction was carried out following the general procedure. Reaction time: 72h. The crude mixture was purified by flash column chromatography (hexane:Et_2O = 7:3). Yield= 33% (46 mg). Colorless oil.

HRMS-ESI-ORBITRAP (+): calculated for $[C_{24}H_{32}N_2NaO_5S]^+$ 483.1924, found 483.1924 $[M+Na]^+$

^{1}H NMR (400 MHz, $CDCl_3$) δ 9.89 – 9.47 (m, 1H), 7.39 – 6.85 (m, 8H), 5.03 – 4.06 (m, 2H), 1.77 – 1.12 (m, 21H).

^{13}C NMR (101 MHz, $CDCl_3$) δ 195.54, 194.87, 194.43, 193.94, 192.24, 155.19, 154.87, 154.54, 154.22, 153.94, 142.63, 140.27, 140.15, 136.64, 136.57, 136.36, 136.09, 132.34, 132.32, 129.79, 129.22, 129.15, 128.59, 128.46, 128.28, 128.18, 127.50, 127.41, 127.32, 127.25, 126.96, 126.94, 126.73, 126.62, 126.38, 126.36, 125.56, 125.44, 125.32, 124.78, 124.58, 123.60, 123.42, 83.81, 82.38, 82.24, 81.91, 81.67, 69.73, 69.44, 69.07, 68.61, 56.35, 56.04, 55.20, 54.08, 29.67, 28.34, 28.26, 28.16, 28.01, 27.89, 27.76, 27.53, 27.51, 19.33, 19.10, 19.00.

IR (ATR) ν(max)= 1734 (s) 1692 (s) 1367 (s) 1150 (s) cm^{-1}.

(*S*, *R*a)-di-tert-butyl 1-benzyl-2-(1-hydroxy-2-(thiophen-2-yl)propan-2-yl)hydrazine-1,2-dicarboxylate (5ir)

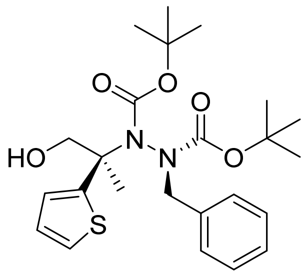

The reaction was carried out following the general reduction procedure. The d.r. and e.e. were determined by HPLC analysis on a Daicel Chiralpak IC: hexane/*i*-PrOH 98/2, flow rate 0.5 mL/min, 25 °C, λ = 220 nm: t_1= 41 min, t_2= 43 min, t_3= 48 min, t_4= 66 min. D.r.= 1.3:1 and e.e.(major diastereoisomer)= 92%. Peaks 1 and 2: minor diastereoisomer; peaks 3 and 4: major diastereoisomer.

^{1}H NMR (400 MHz, $CDCl_3$) δ 8.13 – 6.85 (m, 7H), 5.13 – 3.30 (m, 4H), 1.82 – 1.10 (m, 21H).

(*S*, *R*a)-di-tert-butyl 1-benzyl-2-(1-oxo-2-phenylbutan-2-yl)hydrazine-1,2-dicarboxylate (5j)

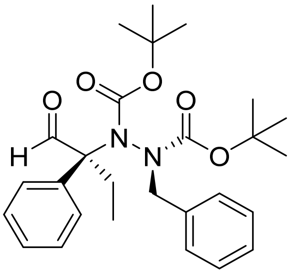

The reaction was carried out following the general procedure. (with 1.5 eq of **4a**). Reaction time: 7 days. The crude mixture was purified by flash column chromatography (hexane:Et_2O = 8:2).
Yield= 34% (32 mg). Colorless oil.
HRMS-ESI-ORBITRAP (+): calculated for $[C_{27}H_{36}N_2NaO_5]^+$ 491.2516, found 491.2516 $[M+Na]^+$.
^{1}H NMR (400 MHz, $CDCl_3$) δ 10.30 – 9.49 (m, 1H), 7.73 – 6.89 (m, 10H), 5.46 – 4.10 (m, 2H), 2.75 – 0.52 (m, 23H).
^{13}C NMR (101 MHz, $CDCl_3$) δ 198.20, 197.40, 196.25, 195.15, 156.26, 155.65, 155.39, 154.92, 154.74, 154.40, 154.13, 152.95, 150.07, 138.07, 137.93, 137.36, 136.95, 136.79, 136.73, 136.45, 136.28, 135.59, 130.50, 130.29, 130.14, 129.93, 129.74, 129.65, 129.58, 129.39, 128.97, 128.77, 128.57, 128.41, 128.36, 128.34, 128.28, 128.26, 128.24, 128.21, 128.16, 128.12, 127.90, 127.60, 127.50, 127.43, 127.36, 127.32, 127.24, 126.95, 83.12, 82.84, 82.11, 82.00, 81.62, 81.34, 81.24, 80.93, 80.83, 80.72, 80.50, 75.14, 74.63, 74.50, 73.65, 73.50, 70.48, 57.01, 56.42, 54.55, 52.56, 28.40, 28.35, 28.33, 28.22, 28.18, 28.13, 27.98, 27.92, 27.81, 27.74, 27.71, 27.39, 26.93, 23.98, 23.64, 23.10, 10.81, 9.24, 9.00, 8.75, 8.58, 8.40.
IR (ATR) ν(max)= 1699 (s) 1367 (s) 1152 (s) cm^{-1}.

(*S*, *R*a)-di-tert-butyl 1-benzyl-2-(1-hydroxy-2-phenylbutan-2-yl)hydrazine-1,2-dicarboxylate (5jr)

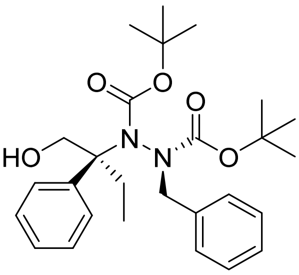

The reaction was carried out following the general reduction procedure, using 1.5 eq of **4a**. The d.r. and e.e. were determined by HPLC analysis on a Phenomenex Lux 5u Cellulose-2 column: hexane/*i*-PrOH 95/5, flow rate 1 mL/min, 25 °C, λ = 220 nm: t_1= 8 min, t_2= 18, t_3= 20, t_4= 27 min. D.r.= 2:1

and e.e.(major diastereoisomer)= >99%. Peaks 1 and 4: minor diastereoisomer; peaks 2 and 3: major diastereoisomer.

^{1}H NMR (400 MHz, $CDCl_3$) δ 7.80 – 6.66 (m, 10H), 5.47 – 3.64 (m, 4H), 2.65 – 0.42 (m, 23H).

(*S*, *R*$_a$)-di-tert-butyl (S)-1-(4-methoxybenzyl)-2-(1-oxo-2-phenylpropan-2-yl)hydrazine-1,2-dicarboxylate (6b)

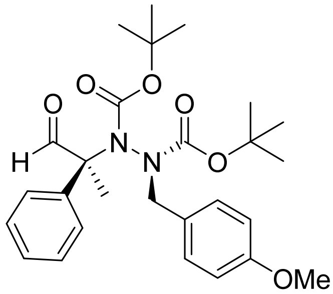

The reaction was carried out following the general procedure. Reaction time: 48h. The crude mixture was purified by flash column chromatography (hexane:Et_2O = 3:1). Yield=76% (110 mg). Colorless oil. The d.r. and e.e. were determined by HPLC analysis on a Daicel Chiralpak IC column: hexane/*i*-PrOH 98/2, flow rate 1 mL/min, 25 °C, λ = 220 nm: t_1 = 21 min, t_2 = 23 min, t_3 = 25 min, t_4 = 37 min. d.r.= 9.6:1 and e.e.(major diastereoisomer)= >99%. Peaks 1 and 3: minor diastereoisomer; peaks 2 and 4: major diastereoisomer.

HRMS-ESI-ORBITRAP (+): calculated for $[C_{27}H_{36}N_2NaO_6]^+$ 507.2466, found 507.2457 $[M+Na]^+$.

^{1}H NMR (400 MHz, $CDCl_3$) δ 9.95-955 (m 1H), 7.49 – 6.63 (m, 9H), 5.13 – 4.08 (m, 2H), 3.82 – 3.68 (m, 3H), 1.58 – 1.12 (m, 21H).

^{13}C NMR (400 MHz, $CDCl_3$) δ 197.52, 196.46, 195.79, 159.04, 156.63, 155.52, 155.34, 155.13, 154.74, 153.15, 139.14, 138.90, 131.03, 130.70, 130.47, 130.19, 129.00, 128.78, 128.24, 128.20, 128.01, 127.83, 127.65, 127.44, 127.24, 126.71, 126.07, 113.52, 83.28, 82.48, 82.34, 82.20, 82.04, 81.95, 72.80, 71.64, 71.54, 71.42, 55.57, 55.28, 55.24, 55.18, 53.85, 29.26, 28.35, 28.31, 28.25, 27.90, 27.87, 27.75, 20.06, 19.84.

IR (ATR) ν(max)= 1695 (s) 1367 (s) 1248 (s) 1150 (s) 1125 (s) cm^{-1}.

(*S*, *R*a)-di-tert-butyl (S)-1-(3-methoxybenzyl)-2-(1-oxo-2-phenylpropan-2-yl)hydrazine-1,2-dicarboxylate (6c)

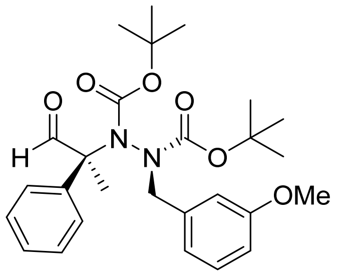

The reaction was carried out following the general procedure. Reaction time: 48h. The crude mixture was purified by flash column chromatography (hexane:Et_2O = 3:1). Yield=70% (101 mg). Colorless oil.

HRMS-ESI-ORBITRAP (+): calculated for $[C_{27}H_{36}N_2NaO_6]^+$ 507.2466, found 507.2451 $[M+Na]^+$.

^{1}H NMR (400 MHz, $CDCl_3$) δ 9.92-9.62 (m, 1H), 7.43 – 6.46 (m, 9H), 5.17 – 4.09 (m, 2H), 3.68 (m, 3H), 1.62 – 1.10 (m, 21H).

^{13}C NMR (400 MHz, $CDCl_3$) δ 210.82, 206.91, 197.36, 196.24, 195.65, 159.40, 156.73, 155.49, 155.35, 155.06, 154.70, 138.97, 138.78, 138.47, 138.16, 138.09, 129.17, 128.26, 128.21, 128.03, 127.85, 127.68, 127.28, 126.13, 122.06, 121.44, 114.82, 114.50, 114.32, 113.59, 113.27, 83.41, 82.40, 82.27, 82.14, 82.06, 71.63, 71.41, 69.49, 56.43, 56.28, 55.07, 54.99, 54.45, 53.79, 31.72, 30.91, 29.26, 28.33, 28.29, 28.22, 27.89, 27.82, 20.00, 19.69.

IR (ATR) ν(max)= 1696 (s) 1367 (s) 1260 (s) 1150 (s) 1125 (s) cm^{-1}.

(*S*, *R*a)-di-tert-butyl 1-(1-hydroxy-2-phenylpropan-2-yl)-2-(3-methoxybenzyl)hydrazine-1,2-dicarboxylate (6cr)

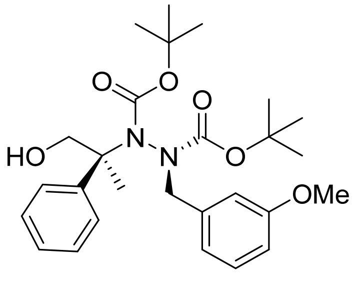

The reaction was carried out following the general reduction procedure. The d.r. and e.e. were determined by HPLC analysis on a Daicel Chiralpak IC column: hexane/*i*-PrOH 92/8, flow rate 1 mL/min, 25 °C, λ = 220 nm: t_1 = 9 min, t_2 = 11 min, t_3 = 15 min, t_4 = 18 min. D.r.= 7:1 and e.e.(major diastereoisomer)= 99%. Peaks 1 and 2: minor diastereoisomer; peaks 3 and 4: major diastereoisomer.

^{1}H NMR (400 MHz, $CDCl_3$) δ 7.82 – 6.72 (m, 9H), 5.32 – 3.38 (m, 7H), 1.78 – 1.02 (m, 21H).

(*S*, *R*a)-di-tert-butyl (S)-1-(2-methoxybenzyl)-2-(1-oxo-2-phenylpropan-2-yl)hydrazine-1,2-dicarboxylate (6d)

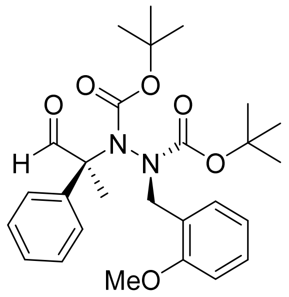

The reaction was carried out following the general procedure, at -5 °C. Reaction time: 5 days. The crude mixture was purified by flash column chromatography (hexane:Et_2O = 3:1). Yield=48% (70 mg). Colorless oil.

HRMS-ESI-ORBITRAP (+): calculated for $[C_{27}H_{36}N_2NaO_6]^+$ 507.2466, found 507.2452 $[M+Na]^+$.

^{1}H NMR (400 MHz, $CDCl_3$) δ 9.99 – 9.68 (m, 1H), 7.54 – 6.55 (m, 9H), 5.16 – 4.07 (m, 2H), 3.85 – 3.56 (m, 3H), 1.84 – 1.06 (m, 21H).

^{13}C NMR (400 MHz, $CDCl_3$) δ 198.25, 197.56, 197.46, 196.66, 196.45, 157.50, 157.45, 157.32, 157.24, 156.82, 156.06, 155.53, 155.42, 155.34, 155.24, 154.65, 154.36, 153.37, 152.84, 140.46, 139.25, 139.04, 136.75, 136.37, 132.16, 131.68, 131.12, 130.53, 128.74, 128.52, 128.21, 128.17, 128.05, 128.01, 127.89, 127.52, 127.41, 127.32, 127.01, 126.84, 126.59, 125.88, 125.21, 125.14, 124.41, 120.46, 120.29, 120.02, 119.93, 109.93, 109.88, 109.73, 82.99, 82.92, 82.40, 82.04, 81.87, 81.81, 81.78, 81.76, 81.70, 81.61, 81.26, 73.10, 72.05, 71.65, 69.48, 55.02, 54.85, 54.76, 53.80, 51.21, 50.66, 50.27, 49.17, 48.94, 31.72, 31.58, 30.32, 29.69, 29.27, 28.32, 28.29, 28.26, 28.19, 27.78, 22.69, 22.64, 20.80, 20.18, 18.38, 17.88, 14.12.

IR (ATR) ν(max)= 1695 (s) 1367 (s) 1245 (s) 1152 (s) 1131 (s) cm^{-1}.

(*S*, *R*a)-di-tert-butyl 1-(1-hydroxy-2-phenylpropan-2-yl)-2-(2-methoxybenzyl)hydrazine-1,2-dicarboxylate (6dr)

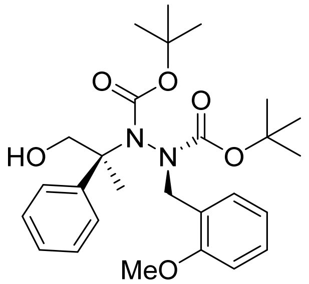

The reaction was carried out following the general reduction procedure. The d.r. and e.e. were determined by HPLC analysis on a Daicel Chiralpak IC column: hexane/*i*-PrOH 90/10, flow rate 1

mL/min, 25 °C, λ = 220 nm: t_1 = 8 min, t_2 = 9 min, t_3 = 16 min, t_4 = 23 min. D.r.= 2.4:1 and e.e.(major diastereoisomer)= 97%. Peaks 1 and 2: minor diastereoisomer; peaks 3 and 4: major diastereoisomer.
^{1}H NMR (600 MHz, $CDCl_3$) δ 7.96 – 6.68 (m, 9H), 5.26 – 3.43 (m, 7H), 1.90 – 0.96 (m, 21H).

(*S*, *R*a)-di-tert-butyl (S)-1-(4-methylbenzyl)-2-(1-oxo-2-phenylpropan-2-yl)hydrazine-1,2-dicarboxylate (6e)

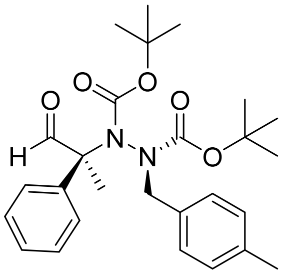

The reaction was carried out following the general procedure. Reaction time: 48h. The crude mixture was purified by flash column chromatography (hexane:Et_2O = 3:1). Yield=70% (98 mg). Colorless oil. The d.r. and e.e. were determined by HPLC analysis on a Daicel Chiralpak IC column: hexane/*i*-PrOH 98/2, flow rate 1 mL/min, 25 °C, λ = 220 nm: t_1 = 17 min, t_2 = 19 min, t_3 = 21, t_4 = 33 min. d.r.= 7:1 and e.e.(major diastereoisomer)= >99%. Peaks 1 and 3: minor diastereoisomer; peaks 2 and 4: major diastereoisomer.
HRMS-ESI-ORBITRAP (+): calculated for $[C_{27}H_{36}N_2NaO_5]^+$ 491.2516, found 491.2512 $[M+Na]^+$.
^{1}H NMR (400 MHz, $CDCl_3$) δ 9.92 – 9.63 (m, 1H), 7.41 – 6.76 (m, 9H), 5.13 – 4.08 (m, 2H), 2.30 (m, 3H), 1.79 – 1.09 (m, 21H).
^{13}C NMR (400 MHz, $CDCl_3$) δ 198.09, 197.53, 196.47, 196.18, 196.02, 195.82, 156.96, 156.71, 155.59, 155.37, 155.11, 154.72, 154.17, 153.18, 140.02, 139.12, 138.89, 137.10, 136.32, 135.97, 133.82, 133.58, 133.50, 133.09, 129.73, 129.31, 129.13, 128.90, 128.81, 128.78, 128.56, 128.51, 128.29, 128.23, 128.19, 128.02, 127.80, 127.61, 127.48, 127.21, 126.74, 126.02, 83.24, 82.50, 82.34, 82.21, 82.05, 81.95, 81.85, 81.49, 72.80, 71.67, 71.55, 71.45, 56.88, 56.04, 54.92, 54.23, 28.34, 28.27, 28.23, 28.16, 27.86, 27.82, 27.74, 26.58, 22.65, 22.18, 21.10, 21.08, 20.12, 19.90, 19.04, 18.46.
IR (ATR) ν(max)= 1697 (s) 1367 (s) 1150 (s) 1127 (s) cm^{-1}.

(*S*, *R*a)-di-tert-butyl (S)-1-(2-methylbenzyl)-2-(1-oxo-2-phenylpropan-2-yl)hydrazine-1,2-dicarboxylate (6f)

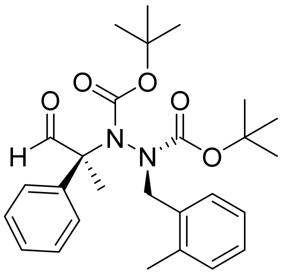

The reaction was carried out following the general procedure. Reaction time: 48h. The crude mixture was purified by flash column chromatography (hexane:Et_2O = 3:1). Yield=69% (97 mg). Colorless oil.

HRMS-ESI-ORBITRAP (+): calculated for $[C_{27}H_{36}N_2NaO_5]^+$ 491.2516, found 491.2514 $[M+Na]^+$.

^{1}H NMR (400 MHz, $CDCl_3$) δ 9.97-9.74 (m, 1H), 7.43 – 6.58 (m, 9H), 5.18 – 4.02 (m, 2H), 2.33 – 1.87 (m, 3H), 1.68 – 1.04 (m, 21H).

^{13}C NMR (400 MHz, $CDCl_3$) δ 197.85, 196.83, 195.93, 155.89, 155.28, 154.71, 138.85, 137.13, 136.89, 136.35, 134.47, 134.14, 133.09, 130.60, 130.18, 129.77, 128.56, 128.29, 128.24, 128.17, 128.00, 127.84, 127.68, 127.64, 127.39, 126.01, 125.53, 83.54, 83.31, 82.36, 82.15, 72.79, 71.66, 71.25, 65.83, 53.38, 51.20, 28.31, 28.10, 27.79, 19.70, 19.07, 18.38, 15.27.

IR (ATR) ν(max)= 1696 (s) 1367 (s) 1150 (s) cm^{-1}.

(*S*, *R*a)-di-tert-butyl 1-(1-hydroxy-2-phenylpropan-2-yl)-2-(2-methylbenzyl)hydrazine-1,2-dicarboxylate (6fr)

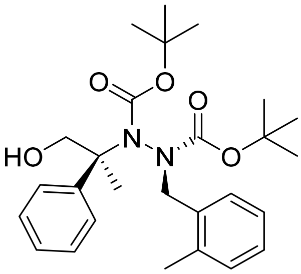

The reaction was carried out following the general reduction procedure. The d.r. and e.e. were determined by HPLC analysis on a Daicel Chiralpak IC column: hexane/*i*-PrOH 95/5, flow rate 1 mL/min, 25 °C, λ = 220 nm: t_1 = 10 min, t_2 = 10.5 min, t_3 = 17 min, t_4 = 22 min. D.r.= 5:1 and e.e.(major diastereoisomer)= 98%. Peaks 1 and 2: minor diastereoisomer; peaks 3 and 4: major diastereoisomer.

^{1}H NMR (600 MHz, $CDCl_3$): δ 7.72 – 6.80 (m, 9H), 5.35 – 3.74 (m, 4H), 2.47 – 2.08 (m, 3H), 1.75 – 0.91 (m, 21H).

(*S*, *R*a)-di-tert-butyl (S)-1-(4-(tert-butyl)benzyl)-2-(1-oxo-2-phenylpropan-2-yl)hydrazine-1,2-dicarboxylate (6g)

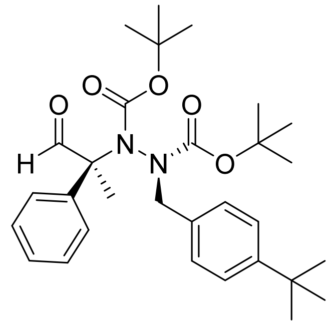

The reaction was carried out following the general procedure. Reaction time: 48h. The crude mixture was purified by flash column chromatography (hexane:Et_2O = 3:1). Yield=67% (104 mg). Colorless oil. The d.r.and e.e. were determined by HPLC analysis on a Daicel Chiralpak IC column: hexane/*i*-PrOH 98/2, flow rate 1 mL/min, 25 °C, λ = 220 nm: t_1 = 13 min, t_2 = 14 min, t_3 = 15 min, t_4 = 18 min. d.r.= 10:1 and e.e.(major diastereoisomer)= 98.6 %. Peaks 1 and 3: minor diastereoisomer; peaks 2 and 4: major diastereoisomer.

HRMS-ESI-ORBITRAP (+): calculated for $[C_{30}H_{42}N_2NaO_5]^+$ 533.2986, found 533.2980 $[M+Na]^+$.

^{1}H NMR (400 MHz, $CDCl_3$) δ 9.96-9.61 (m, 1H), 7.48 – 6.98 (m, 9H), 5.09 – 3.91 (m, 2H), 1.64 – 1.07 (m, 30H).

^{13}C NMR (400 MHz, $CDCl_3$) δ 198.07, 197.51, 196.36, 196.17, 195.69, 155.34, 155.16, 155.12, 154.77, 153.20, 150.47, 150.40, 150.27, 140.03, 139.19, 138.94, 137.13, 133.60, 133.54, 133.08, 129.66, 129.12, 128.61, 128.56, 128.29, 128.26, 128.21, 128.04, 127.88, 127.69, 127.31, 126.05, 125.09, 125.06, 83.21, 82.47, 82.24, 82.08, 81.96, 81.89, 81.77, 81.46, 72.84, 71.60, 71.48, 71.42, 55.90, 55.42, 54.27, 53.56, 34.46, 31.57, 31.34, 28.35, 28.30, 28.23, 27.79, 27.73, 26.90, 26.58, 22.64, 22.55, 19.98, 19.74, 19.14, 18.62, 14.12.

IR (ATR) ν(max)= 1695 (s) 1366 (s) 1152 (s) 1129 (s) cm^{-1}.

(*S*, *R*a)-di-tert-butyl (S)-1-(4-isopropylbenzyl)-2-(1-oxo-2-phenylpropan-2-yl)hydrazine-1,2-dicarboxylate (6h)

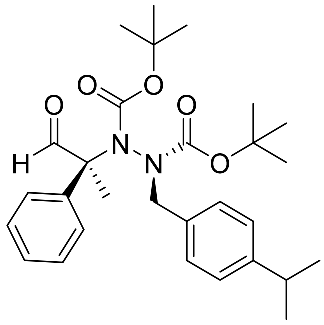

The reaction was carried out following the general procedure. Reaction time: 48h. The crude mixture was purified by flash column chromatography (hexane:Et_2O = 3:1). Yield=70 % (104 mg). Colorless oil. The d.r.and e.e. were determined by HPLC analysis on a Daicel Chiralpak IC column: hexane/*i*-PrOH 98/2, flow rate 1 mL/min, 25 °C, λ = 220 nm: t_1 = 15 min, t_2 = 17 min, t_3 = 18 min, t_4 = 22 min. d.r.= 10:1 and e.e.(major diastereoisomer)= 98.5 %. Peaks 1 and 3: minor diastereoisomer; peaks 2 and 4: major diastereoisomer.

HRMS-ESI-ORBITRAP (+): calculated for $[C_{29}H_{40}N_2NaO_5]^+$ 519.2829, found 519.2823 $[M+Na]^+$.

^{1}H NMR (400 MHz, $CDCl_3$) δ 9.75 (m, 1H), 7.42 – 6.98 (m, 9H), 5.08 – 3.96 (m, 2H), 2.86 (m, 1H), 1.84 – 1.10 (m, 27H).

^{13}C NMR (400 MHz, $CDCl_3$) δ 198.09, 197.54, 196.39, 195.72, 156.68, 155.38, 155.20, 155.11, 154.77, 153.18, 148.23, 148.20, 148.07, 140.01, 139.16, 138.93, 137.13, 133.98, 133.90, 133.08, 129.92, 129.36, 128.56, 128.29, 128.26, 128.21, 128.03, 127.87, 127.68, 127.29, 126.75, 126.23, 126.20, 126.05, 82.48, 82.26, 82.11, 81.98, 81.90, 81.79, 81.46, 72.83, 71.61, 71.49, 71.42, 56.86, 55.94, 55.61, 54.44, 53.73, 33.84, 33.75, 29.68, 28.35, 28.29, 28.23, 27.80, 27.74, 26.59, 24.06, 24.00, 23.97, 22.56, 19.99, 19.75.

IR (ATR) ν(max)= 1695 (s) 1367 (s) 1152 (s) 1131 (s) cm^{-1}.

(*S*, *R*a)-di-tert-butyl (S)-1-(naphthalen-1-ylmethyl)-2-(1-oxo-2-phenylpropan-2-yl)hydrazine-1,2-dicarboxylate (6i)

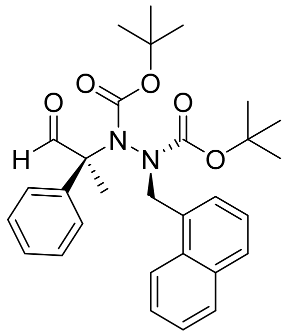

The reaction was carried out following the general procedure. Reaction time: 48h. The crude mixture was purified by flash column chromatography (hexane:Et_2O = 3:1). Yield=68% (103 mg). Colorless oil.

HRMS-ESI-ORBITRAP (+): calculated for $[C_{30}H_{36}N_2NaO_5]^+$ 527.2516, found 527.2508 $[M+Na]^+$.

^{1}H NMR (400 MHz, $CDCl_3$) δ 10.04 – 9.43 (m, 1H), 8.20 – 6.65 (m, 12H), 5.38 – 4.28 (m, 2H), 1.71 – 0.76 (m, 21H).

^{13}C NMR (400 MHz, $CDCl_3$) δ 198.13, 196.79, 196.61, 195.71, 155.37, 155.17, 154.88, 154.74, 154.13, 139.06, 138.92, 137.13, 136.35, 133.63, 133.09, 132.66, 132.41, 132.28, 131.88, 129.34, 128.56, 128.38, 128.34, 128.30, 128.19, 128.06, 127.66, 127.47, 126.97, 126.62, 126.33, 125.95, 125.70, 125.59, 125.04, 124.96, 124.84, 124.34, 123.96, 123.40, 83.57, 82.26, 82.18, 82.15, 81.92, 72.85, 71.65, 71.56, 71.07, 69.48, 65.84, 53.78, 52.55, 52.26, 50.18, 29.27, 28.33, 28.27, 27.98, 27.81, 27.65, 27.46, 27.23, 26.60, 19.87, 19.09, 18.90, 18.68, 18.36, 15.28.

IR (ATR) ν(max)= 1736 (s) 1690 (s) 1367 (s) 1151 (s) cm^{-1}.

(*S*, *R*a)-di-tert-butyl 1-(1-hydroxy-2-phenylpropan-2-yl)-2-(naphthalen-1-ylmethyl)hydrazine-1,2-dicarboxylate (6ir)

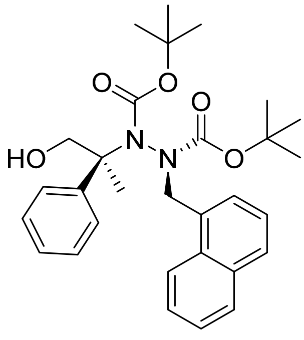

The reaction was carried out following the general reduction procedure. The d.r. and e.e. were determined by HPLC analysis on a Daicel Chiralpak IC column: hexane/*i*-PrOH 90/10, flow rate 1

mL/min, 25 °C, λ = 220 nm: t_1 = 7 min, t_2 = 8 min, t_3 = 11 min, t_4 = 12 min. D.r.= 3:1 and e.e.(major diastereoisomer)= 96%. Peaks 1 and 2: minor diastereoisomer; peaks 3 and 4: major diastereoisomer.
^{1}H NMR of alcohol (600 MHz, Chloroform-*d*) δ 7.98 – 6.85 (m, 12H), 5.39 – 3.74 (m, 4H), 1.71 – 0.74 (m, 21H).

(*S*, *R*a)-di-tert-butyl (S)-1-(naphthalen-2-ylmethyl)-2-(1-oxo-2-phenylpropan-2-yl)hydrazine-1,2-dicarboxylate (6j)

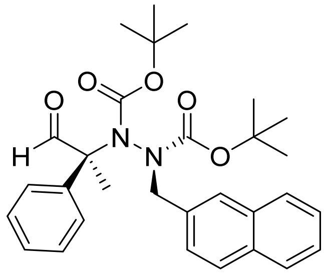

The reaction was carried out following the general procedure. Reaction time: 48h. The crude mixture was purified by flash column chromatography (hexane:Et_2O=3:1). Yield=66% (100 mg). Colorless oil. The d.r. and e.e. were determined by HPLC analysis on a Daicel Chiralpak OD-H column: hexane/*i*-PrOH 98/2, flow rate 0.5 mL/min, 25 °C, λ = 220 nm: t_1 = 13 min, t_2 = 14 min, t_3 = 18 min, t_4 = 21 min. D.r.= 6:1 and e.e.(major diastereoisomer)= >99%. Peaks 2 and 4: minor diastereoisomer; peaks 1 and 3: major diastereoisomer.
HRMS-ESI-ORBITRAP (+): calculated for $[C_{30}H_{36}N_2NaO_5]^+$ 527.2516, found 527.2509 $[M+Na]^+$.
^{1}H NMR (400 MHz, $CDCl_3$) δ 9.93 – 9.62 (m, 1H), 7.86 – 7.12 (m, 12H), 5.35 – 4.22 (m, 2H), 1.84 – 1.12 (m, 21H).
^{13}C NMR (400 MHz, $CDCl_3$) δ 197.32, 196.08, 195.61, 195.46, 156.81, 155.60, 155.45, 155.30, 155.17, 154.76, 154.51, 154.25, 153.18, 139.79, 138.96, 136.04, 135.78, 134.38, 134.17, 134.00, 133.87, 133.02, 132.76, 132.70, 128.61, 128.33, 128.30, 128.03, 127.96, 127.88, 127.78, 127.49, 127.44, 127.40, 126.99, 126.84, 126.13, 126.06, 125.94, 125.81, 83.46, 82.65, 82.44, 82.35, 82.19, 82.14, 82.06, 81.69, 72.76, 71.67, 71.34, 65.84, 57.41, 57.06, 56.64, 56.37, 55.84, 54.60, 53.43, 30.33, 29.70, 29.28, 28.36, 28.29, 28.26, 28.14, 27.84, 27.79, 22.44, 19.73, 19.47, 18.71, 18.16.
IR (ATR) ν(max)= 1695 (s) 1367 (s) 1150 (s) cm^{-1}.

(*S*, *R*a)-di-tert-butyl (S)-1-(4-bromobenzyl)-2-(1-oxo-2-phenylpropan-2-yl)hydrazine-1,2-dicarboxylate (6k)

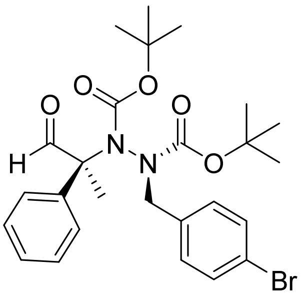

The reaction was carried out following the general procedure. Reaction time: 48h. The crude mixture was purified by flash column chromatography (hexane:Et_2O = 3:1). Yield=70% (112 mg). Colorless oil.

HRMS-ESI-ORBITRAP (+): calculated for $[C_{26}H_{36}BrN_2NaO_5]^+$ 555.1465, found 555.1458 $[M+Na]^+$.

^{1}H NMR (400 MHz, $CDCl_3$) δ 9.86 – 9.52 (m, 1H), 7.45 – 6.60 (m, 9H), 5.01 – 3.94 (m, 2H), 2.10 (s, 3H), 1.65 – 1.06 (m, 18H).

^{13}C NMR (400 MHz, $CDCl_3$) δ 206.76, 196.69, 195.64, 195.10, 156.61, 155.40, 155.31, 155.03, 154.57, 154.30, 154.04, 153.38, 153.04, 139.94, 139.52, 138.69, 136.27, 135.96, 135.82, 135.77, 135.59, 131.30, 131.23, 131.20, 131.13, 130.80, 130.47, 130.18, 129.77, 128.33, 128.15, 127.97, 127.86, 127.41, 127.26, 127.23, 126.10, 121.44, 121.23, 121.13, 120.98, 83.52, 83.33, 82.71, 82.57, 82.48, 82.30, 82.23, 81.84, 72.92, 72.67, 71.66, 71.51, 71.29, 69.43, 56.20, 56.03, 55.77, 55.08, 54.03, 53.80, 30.83, 29.23, 28.26, 28.16, 27.90, 27.84, 27.72, 22.14, 21.62, 19.67, 19.21, 18.76, 18.27.

IR (ATR) ν(max)= 1697 (s) 1367 (s) 1149 (s) 1127 (s) cm^{-1}.

(*S*, *R*a)-di-tert-butyl 1-(4-bromobenzyl)-2-(1-hydroxy-2-phenylpropan-2-yl)hydrazine-1,2-dicarboxylate (6kr)

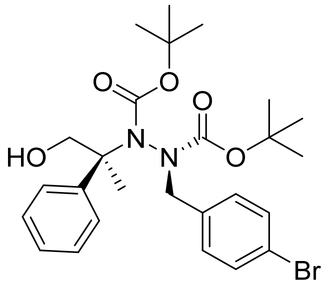

The reaction was carried out following the general reduction procedure. The d.r. and e.e. were determined by HPLC analysis on Phenomenex Lux 5u Cellulose-2 column: hexane/*i*-PrOH 90/10, flow rate 1 mL/min, 25 °C, λ = 220 nm: t_1 = 6 min, t_2 = 7 min, t_3 = 9 min, t_4 = 22 min. D.r.= 8.5:1 and e.e.(major diastereoisomer)= 99%. Peaks 1 and 2: minor diastereoisomer; peaks 3 and 4: major

diastereoisomer. The reported HPLC chromatogram is referred to the large-scale reaction (d.r.= 7:1, e.e.= 95.5%).

^{1}H NMR: (600 MHz, $CDCl_3$) δ 7.73 – 6.91 (m, 9H), 5.29 – 3.72 (m, 4H), 1.76 – 0.95 (m, 21H).

(*S*, *R*a)-di-tert-butyl (S)-1-(4-cyanobenzyl)-2-(1-oxo-2-phenylpropan-2-yl)hydrazine-1,2-dicarboxylate (6l)

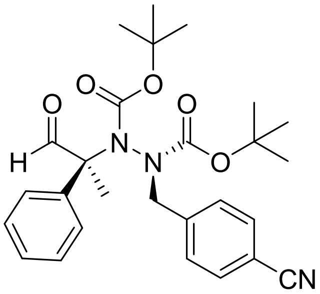

The reaction was carried out following the general procedure, at r.t. Reaction time: 48h. The crude mixture was purified by flash column chromatography (hexane:Et_2O = 2:1). Yield=42% (60 mg). Colorless oil. The d.r. and e.e. were determined by HPLC analysis on a Daicel Chiralpak IC column: hexane/*i*-PrOH 90/10, flow rate 1 mL/min, 25 °C, λ = 220 nm: t_1 = 24 min, t_2 = 28 min, t_3 = 31 min, t_4 = 36 min. D.r.= 4:1 and e.e.(major diastereoisomer)= 98%. Peaks 2 and 4: minor diastereoisomer; peaks 1 and 3: major diastereoisomer.

HRMS-ESI-ORBITRAP (+): calculated for $[C_{27}H_{33}N_3NaO_5]^+$ 502.2312, found 502.2305 $[M+Na]^+$.

^{1}H NMR (400 MHz, $CDCl_3$) δ 9.87 – 9.54 (m, 1H), 7.55 – 6.72 (m, 9H), 5.02 – 4.02 (m, 2H), 1.75 – 1.09 (m, 21H).

^{13}C NMR (400 MHz, $CDCl_3$) δ 196.39, 196.02, 195.17, 194.89, 194.61, 156.61, 155.51, 154.98, 154.45, 153.03, 142.47, 142.03, 139.04, 138.38, 138.09, 131.94, 131.90, 131.77, 129.85, 129.26, 128.90, 128.68, 128.50, 128.33, 128.18, 127.76, 127.68, 127.43, 127.39, 127.22, 126.25, 118.66, 111.17, 110.92, 83.90, 83.08, 82.86, 82.75, 82.64, 82.60, 82.27, 72.69, 71.90, 71.65, 71.35, 69.47, 65.81, 57.06, 56.37, 55.99, 54.88, 53.78, 31.71, 30.30, 29.25, 28.26, 28.17, 28.07, 28.01, 27.95, 27.75, 21.58, 19.39, 18.59, 15.26.

IR (ATR) ν(max)= 2229 (s) 1697 (s) 1368 (s) 1149 (s) cm^{-1}.

(S, R_a)-di-tert-butyl (S)-1-(3-nitrobenzyl)-2-(1-oxo-2-phenylpropan-2-yl)hydrazine-1,2-dicarboxylate (6m)

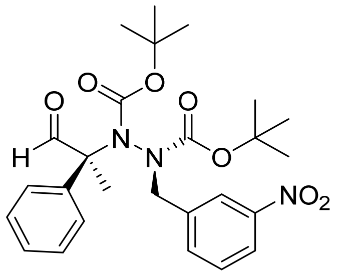

The reaction was carried out following the general procedure. Reaction time: 48h. The crude mixture was purified by flash column chromatography (hexane:Et_2O = 2:1). Yield=65% (98 mg). Colorless oil. The d.r. and e.e. were determined by HPLC analysis on a Phenomenex Lux 5u Cellulose-2 column: hexane/*i*-PrOH 90/10, flow rate 1 mL/min, 25 °C, λ = 220 nm: t_1 = 10 min, t_2 = 11 min, t_3 = 12 min, t_4 = 15 min. D.r.= 6:1 and e.e.(major diastereoisomer)= 98%. Peaks 2 and 3: minor diastereoisomer; peaks 1 and 4: major diastereoisomer.

HRMS-ESI-ORBITRAP (+): calculated for $[C_{26}H_{33}N_3NaO_7]^+$ 522.2211, found 522.2202 $[M+Na]^+$.

^{1}H NMR (400 MHz, $CDCl_3$) δ 9.92 – 9.56 (m, 1H), 8.11 – 7.11 (m, 9H), 5.02 – 4.06 (m, 2H), 1.77 – 1.15 (m, 21H).

^{13}C NMR (400 MHz, $CDCl_3$) δ 195.99, 195.53, 194.90, 194.41, 156.55, 156.24, 155.72, 155.51, 155.33, 155.03, 154.86, 154.52, 154.23, 154.02, 153.02, 148.20, 148.00, 139.12, 139.02, 138.89, 138.78, 138.31, 138.13, 135.43, 135.26, 135.05, 134.15, 133.71, 129.08, 128.93, 128.52, 128.41, 128.36, 128.23, 128.04, 127.79, 127.34, 127.19, 126.85, 126.31, 125.33, 123.93, 123.52, 123.28, 123.01, 122.34, 122.13, 122.08, 83.97, 83.07, 82.86, 82.78, 82.52, 72.92, 72.64, 71.80, 71.59, 71.29, 56.45, 55.73, 54.48, 31.54, 30.29, 29.24, 28.24, 28.15, 28.09, 27.93, 27.76, 25.21, 22.61, 21.49, 21.13, 19.21, 18.58, 18.42, 18.22, 14.09.

IR (ATR) ν(max)= 1698 (s) 1528 (s) 1368 (s) 1346 (s) 1148 (s) cm^{-1}.

(*S*, *R*a)-diisopropyl 1-benzyl-2-(1-oxo-2-phenylpropan-2-yl)hydrazine-1,2-dicarboxylate (7)

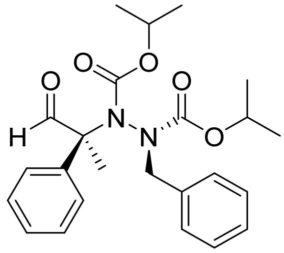

The reaction was carried out following the general procedure. Reaction time: 48h. The crude mixture was purified by flash column chromatography (hexane:Et_2O = 8:2). Yield= 56% (48 mg). Colorless oil.

HRMS-ESI-ORBITRAP (+): calculated for $[C_{24}H_{30}N_2NaO_5]^+$ 449.2047, found 449.2047 $[M+Na]^+$.
The d.r. and e.e. were determined by HPLC analysis on a Daicel Chiralpak IC: hexane/*i*-PrOH 90/10, flow rate 1 mL/min, 25 °C, λ = 220 nm: t_1= 16 min, t_2= 16 min, t_3= 23 min, t_4= 40 min. D.r.= 6.4:1 and e.e.(major)= >99%. Peaks 1 and 2: minor diastereoisomer; peaks 3 and 4: major diastereoisomer.

^{1}H NMR (400 MHz, $CDCl_3$) δ 10.00 – 9.50 (m, 1H), 7.59 – 6.93 (m, 10H), 5.21 – 4.23 (m, 4H), 1.81 – 0.74 (m, 15H).

^{13}C NMR (101 MHz, $CDCl_3$) δ 197.13, 196.21, 195.79, 157.22, 156.58, 156.45, 156.27, 155.60, 155.34, 154.89, 145.91, 141.01, 138.77, 138.63, 136.60, 136.35, 136.29, 129.56, 129.15, 128.49, 128.44, 128.32, 128.29, 128.24, 128.16, 127.91, 127.80, 127.65, 127.52, 127.38, 127.21, 127.09, 126.98, 126.93, 126.17, 126.03, 125.36, 72.89, 72.05, 71.99, 71.87, 71.32, 71.01, 70.86, 70.75, 70.29, 65.23, 56.20, 56.02, 55.21, 29.68, 25.20, 22.20, 22.07, 21.92, 21.82, 21.46, 21.31, 20.27, 19.86, 19.61.

IR (ATR) ν(max)= 1699 (s) 1374 (s) 1105 (s) cm^{-1}.

(*S*, *R*a)-di-tert-butyl (S)-1-allyl-2-(1-oxo-2-phenylpropan-2-yl)hydrazine-1,2-dicarboxylate (8)

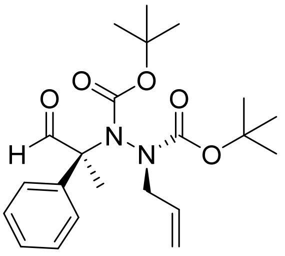

The reaction was carried out following the general procedure, at r.t. Reaction time: 6 days. The crude mixture was purified by flash column chromatography (hexane:Et_2O = 3:1). Yield=40% (50 mg). Colorless oil.

HRMS-ESI-ORBITRAP (+): calculated for $[C_{22}H_{32}N_2NaO_5]^+$ 427.2203, found 427.2202 $[M+Na]^+$.

^{1}H NMR (400 MHz, $CDCl_3$) δ 9.93 – 9.61 (m, 1H), 7.60 – 7.18 (m, 5H), 6.07 – 5.65 (m, 1H), 5.20 – 4.90 (m, 2H), 4.27 – 3.64 (m, 2H), 1.69 – 1.37 (m, 18H), 1.27 – 1.11 (m, 3H).

^{13}C NMR (400 MHz, $CDCl_3$) δ 197.07, 196.38, 195.87, 155.36, 154.97, 154.86, 154.60, 153.20, 138.94, 138.66, 133.74, 133.58, 133.23, 128.22, 128.09, 127.78, 127.63, 127.41, 126.99, 126.90, 126.12, 118.11, 117.84, 117.57, 83.22, 82.58, 82.38, 82.09, 81.73, 72.73, 71.61, 71.47, 56.17, 55.01, 54.20, 30.30, 29.67, 28.32, 28.21, 28.10, 28.07, 27.70, 22.39, 20.50, 20.07.

(*S*, *R*a)-di-tert-butyl 1-allyl-2-(1-hydroxy-2-phenylpropan-2-yl)hydrazine-1,2-dicarboxylate (8r)

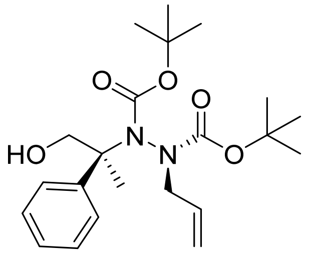

The reaction was carried out following the general reduction procedure. The d.r. and e.e. were determined by HPLC analysis on Daicel Chiralpak IC column: hexane/*i*-PrOH 97.5/2.5, flow rate 1 mL/min, 25 °C, λ = 220 nm: t_1 = 12 min, t_2 = 13 min, t_3 = 22 min, t_4 = 46 min. D.r.= 6:1 and e.e.(major diastereoisomer)= 96%. Peaks 1 and 2: minor diastereoisomer; peaks 3 and 4: major diastereoisomer. The reported HPLC chromatogram is referred to the reaction with the second step at -5°C (d.r.= 15:1, e.e.>99%).

^{1}H NMR (400 MHz, $CDCl_3$) δ 7.93 – 6.97 (m, 6H), 6.18 – 3.43 (m, 8H), 1.92 – 0.86 (m, 21H).

IR (ATR) ν(max)= 3406 (br,m) 1715 (s) 1367 (s) 1144 (s) 1069 (s) cm^{-1}.

(*S*, *R*$_a$)-di-tert-butyl (S)-1-(2-(tert-butoxycarbonyl)allyl)-2-(1-oxo-2-phenylpropan-2-yl)hydrazine-1,2-dicarboxylate (9)

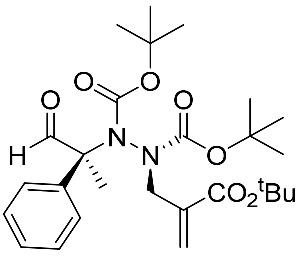

The reaction was carried out following the general procedure. Reaction time: 72h. The crude mixture was purified by flash column chromatography (hexane:Et_2O = 3:1). Yield=40% (60 mg). Colorless oil. The d.r. and e.e. were determined by HPLC analysis on a Daicel Chiralpak IC column: hexane/*i*-PrOH 98/2, flow rate 1 mL/min, 25 °C, λ = 220 nm: t_1 = 13 min, t_2 = 14 min, t_3 = 15 min, t_4 = 17 min. D.r.= 4.7:1 and e.e.(major diastereoisomer)= 90%. Peaks 1 and 4: minor diastereoisomer; peaks 2 and 3: major diastereoisomer.

HRMS-ESI-ORBITRAP (+): calculated for $[C_{27}H_{40}N_2NaO_7]^+$ 527.2728, found 527.2717 $[M+Na]^+$.

^{1}H NMR (400 MHz, $CDCl_3$) δ 9.89 – 9.59 (m, 1H), 7.60 – 7.17 (m, 5H), 6.20 – 3.56 (m, 4H), 1.84 – 1.07 (m, 30H).

^{13}C NMR (400 MHz, $CDCl_3$) δ 197.19, 195.30, 194.05, 193.72, 165.22, 155.71, 155.43, 154.69, 154.58, 154.36, 152.88, 138.22, 137.10, 136.25, 136.16, 135.61, 135.49, 133.07, 128.54, 128.51, 128.34, 128.27, 127.94, 127.37, 127.09, 126.94, 126.33, 126.01, 125.85, 125.34, 83.55, 83.15, 82.95, 82.81, 82.42, 82.07, 81.58, 81.02, 80.88, 80.81, 72.07, 71.99, 71.70, 54.38, 54.06, 53.01, 31.54, 29.65, 29.24, 28.19, 28.11, 28.02, 28.00, 27.93, 27.73, 26.57, 22.61, 19.62, 19.48, 18.71, 17.14, 17.01, 14.08.

IR (ATR) ν(max)= 1702 (s) 1367 (s) 1143 (s) cm^{-1}.

(*S*, *R*a)-di-tert-butyl 1-(2-([1,1'-biphenyl]-4-yl)-1-oxopropan-2-yl)-2-(4-bromobenzyl)hydrazine-1,2-dicarboxylate (10ek)

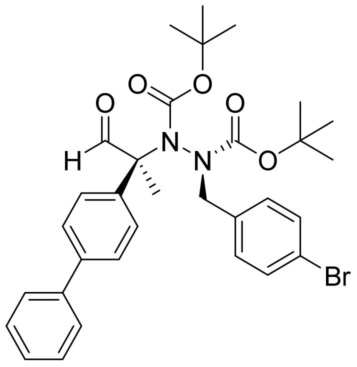

The reaction was carried out following the general procedure. Reaction time: 72h. The crude mixture was purified by flash column chromatography (hexane:Et_2O = 8:2). Yield= 64% (117 mg). White solid.

HRMS-ESI-ORBITRAP (+): calculated for $[C_{32}H_{37}BrN_2NaO_5]^+$ 631.1778, found 631.1775 $[M+Na]^+$.

^{1}H NMR (400 MHz, $CDCl_3$) δ 10.00 – 9.62 (m, 1H), 7.82 – 6.68 (m, 13H), 5.06 – 3.97 (m, 2H), 1.91 – 1.13 (m, 21H).

^{13}C NMR (101 MHz, $CDCl_3$) δ 195.78, 195.57, 195.16, 155.64, 155.45, 155.12, 154.64, 154.31, 154.07, 141.08, 140.82, 140.41, 140.29, 140.17, 137.61, 135.90, 135.61, 134.81, 134.65, 131.26, 131.21, 131.17, 130.48, 129.76, 128.89, 128.81, 127.99, 127.81, 127.68, 127.64, 127.46, 127.11, 127.08, 127.03, 126.91, 126.75, 121.51, 121.20, 121.05, 82.76, 82.66, 82.44, 82.30, 81.99, 71.43, 71.23, 56.34, 55.31, 54.29, 30.91, 29.68, 28.35, 28.15, 27.98, 27.93, 19.87, 19.70, 18.85, 18.42.

IR (ATR) ν(max)= 1698 (s) 1366 (s) 1152 (s) cm^{-1}.

(*S*, *R*a)-di-tert-butyl 1-(2-([1,1'-biphenyl]-4-yl)-1-hydroxypropan-2-yl)-2-(4-bromobenzyl)hydrazine-1,2-dicarboxylate (10ekr)

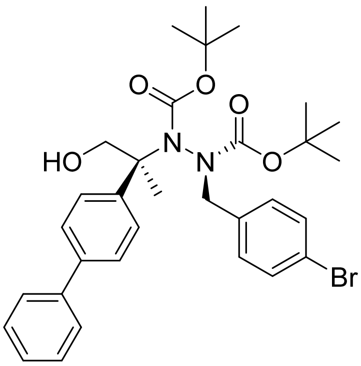

The reaction was carried out following the reported one-pot procedure. The crude mixture was purified by flash column chromatography (hexane:Et_2O = 8:2). Yield= 64% (117 mg). White solid.

The d.r. and e.e. were determined by HPLC analysis on a Phenomenex Lux 5u Cellulose-2 column: hexane/*i*-PrOH 98/2, flow rate 1 mL/min, 25 °C, λ = 220 nm: t_1= 25 min, t_2= 42 min, t_3= 78 min, t_4= 99 min. D.r.= 8:1 and e.e.(major diastereoisomer)= >99%. Peaks 1 and 2: major diastereoisomer; peaks 3 and 4: minor diastereoisomer.

^{1}H NMR (400 MHz, $CDCl_3$) δ 8.08 – 6.80 (m, 13H), 5.23 – 3.15 (m, 4H), 1.89 – 0.96 (m, 21H).

NMR traces

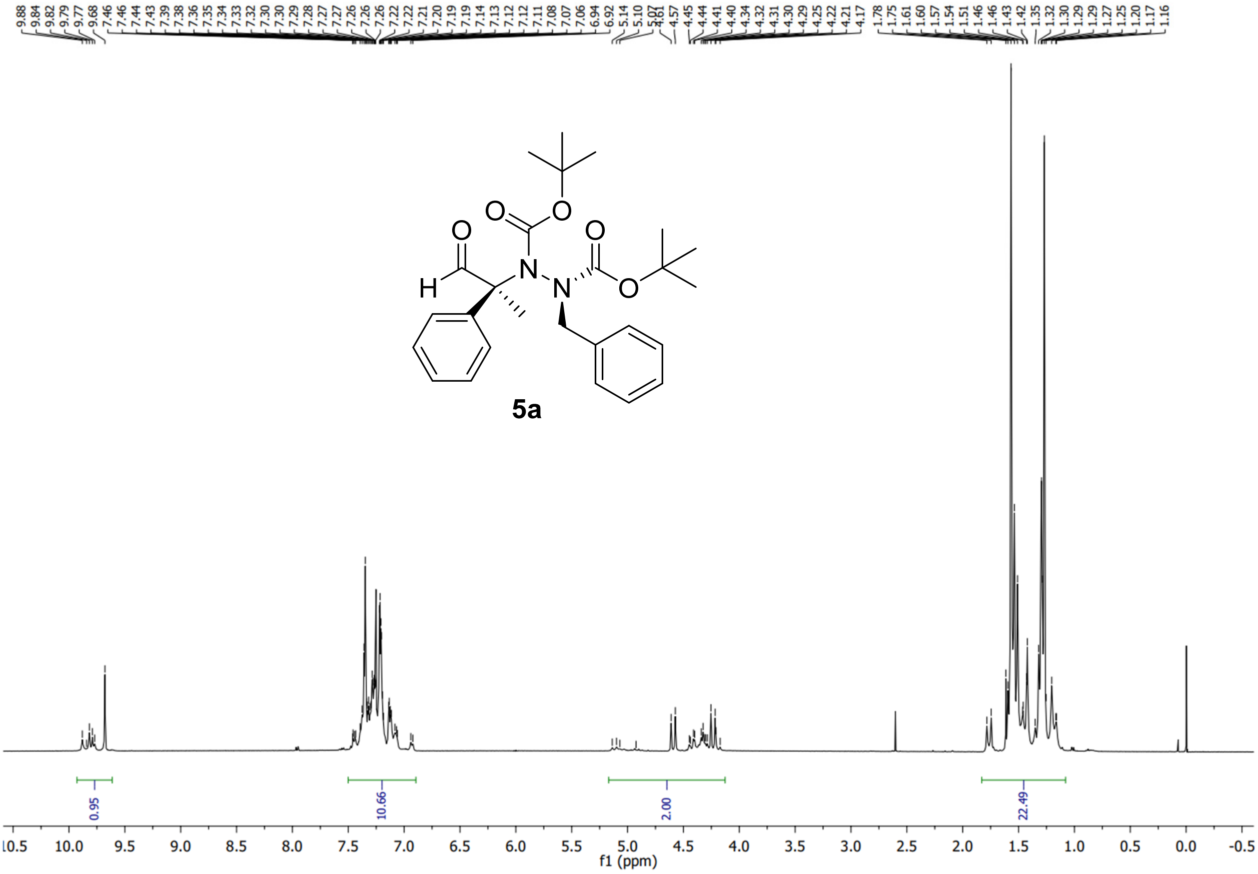

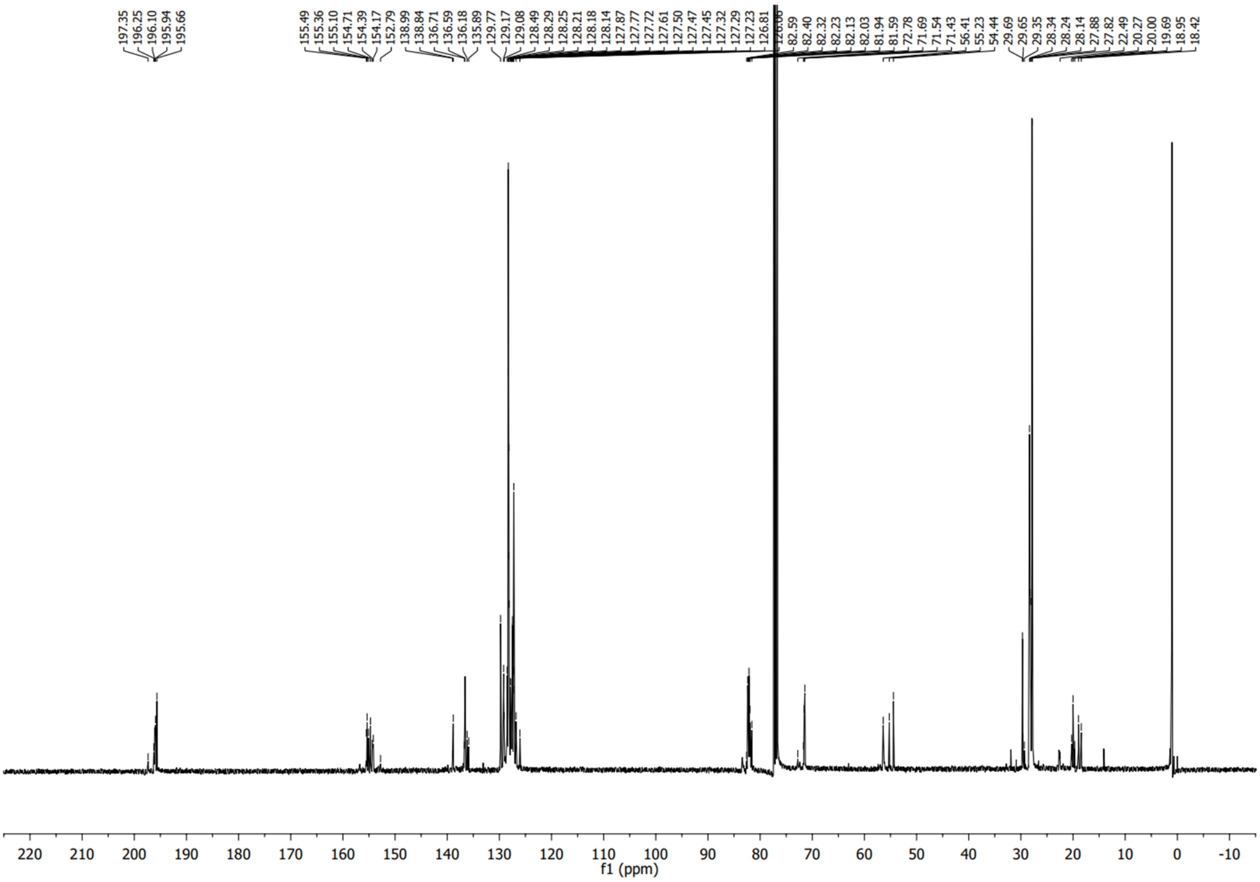

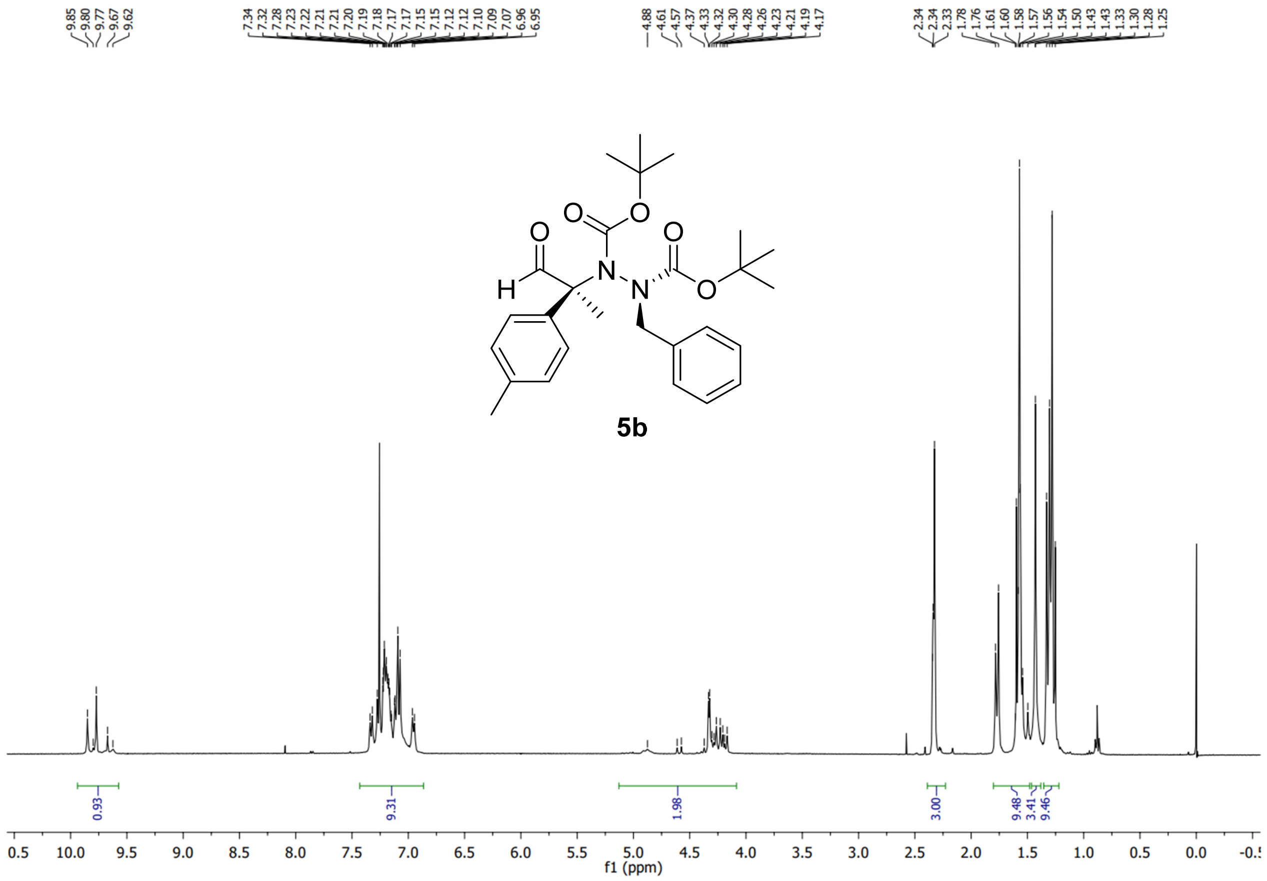

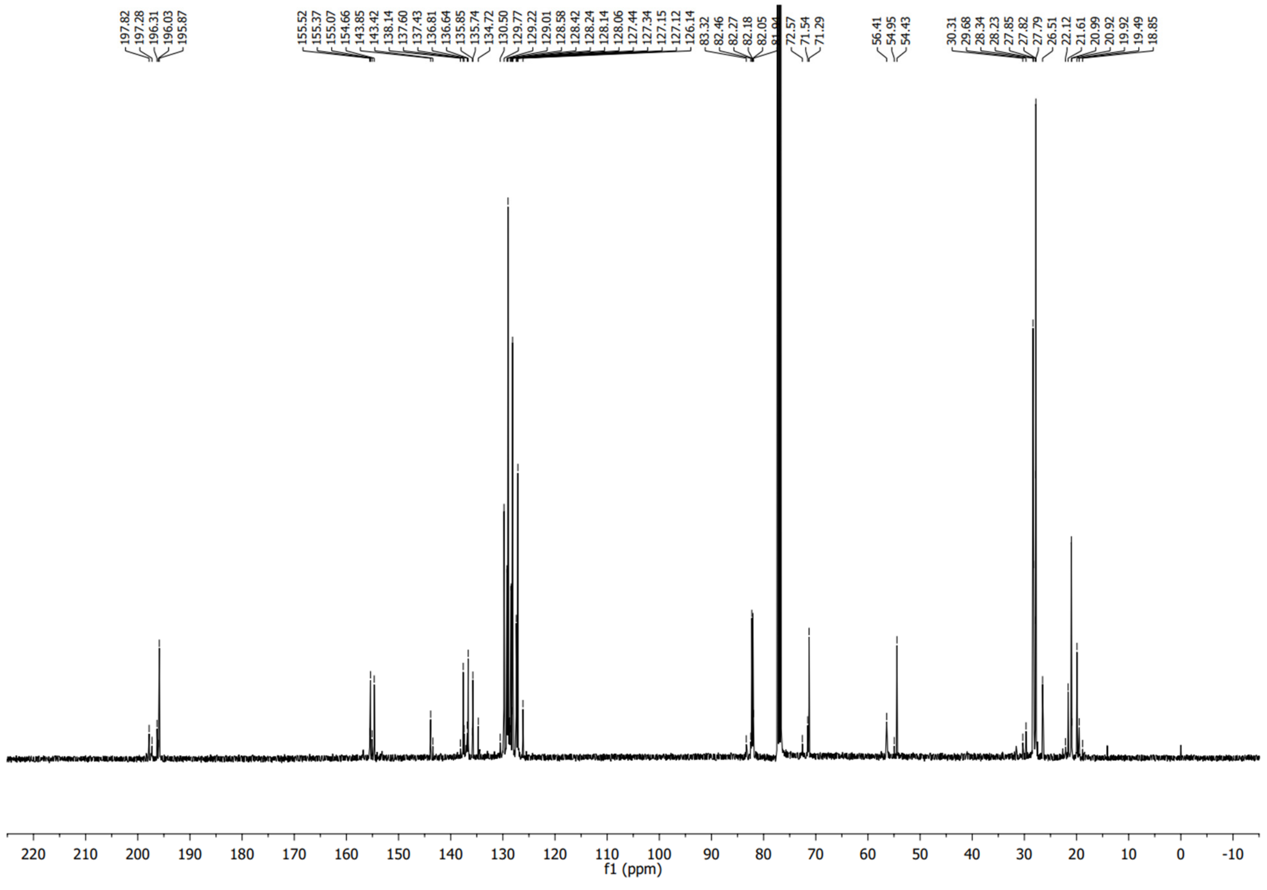

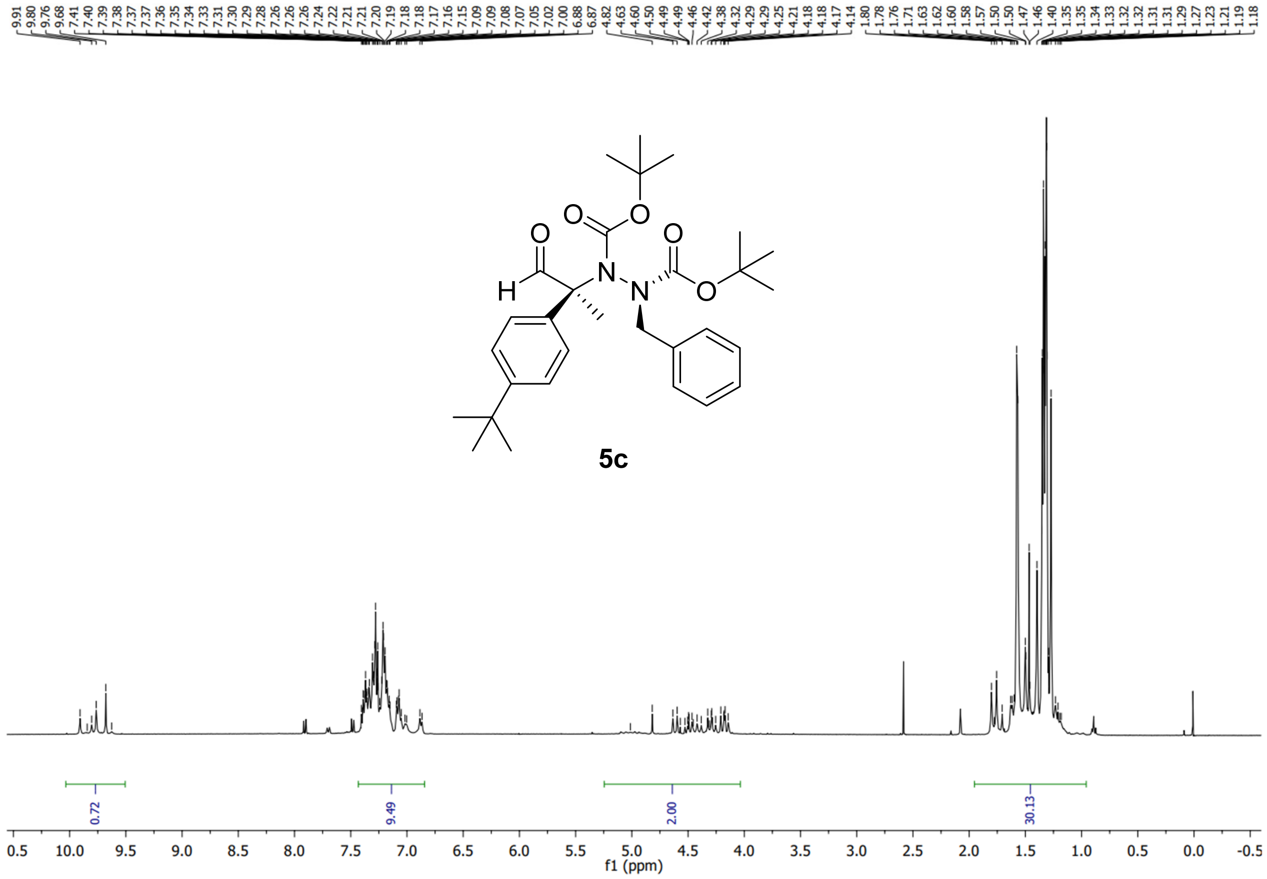
9.91 9.80 9.76 9.68 7.41 7.40 7.39 7.38 7.37 7.37 7.36 7.35 7.34 7.33 7.31 7.30 7.29 7.28 7.26 7.26 7.24 7.22 7.21 7.21 7.20 7.19 7.18 7.18 7.17 7.16 7.15 7.09 7.09 7.08 7.07 7.05 7.02 7.00 6.88 6.87 4.82 4.63 4.60 4.50 4.49 4.49 4.46 4.42 4.38 4.32 4.29 4.29 4.25 4.21 4.18 4.18 4.17 4.14 1.80 1.78 1.76 1.71 1.63 1.62 1.60 1.58 1.57 1.50 1.50 1.47 1.46 1.40 1.35 1.35 1.34 1.33 1.32 1.32 1.31 1.31 1.29 1.27 1.23 1.21 1.19 1.18
O
O
O
O
O
H
N
N
5c
0.72
9.49
2.00
30.13
0.5 10.0 9.5 9.0 8.5 8.0 7.5 7.0 6.5 6.0 5.5 5.0 4.5 4.0 3.5 3.0 2.5 2.0 1.5 1.0 0.5 0.0 -0.5
f1 (ppm)

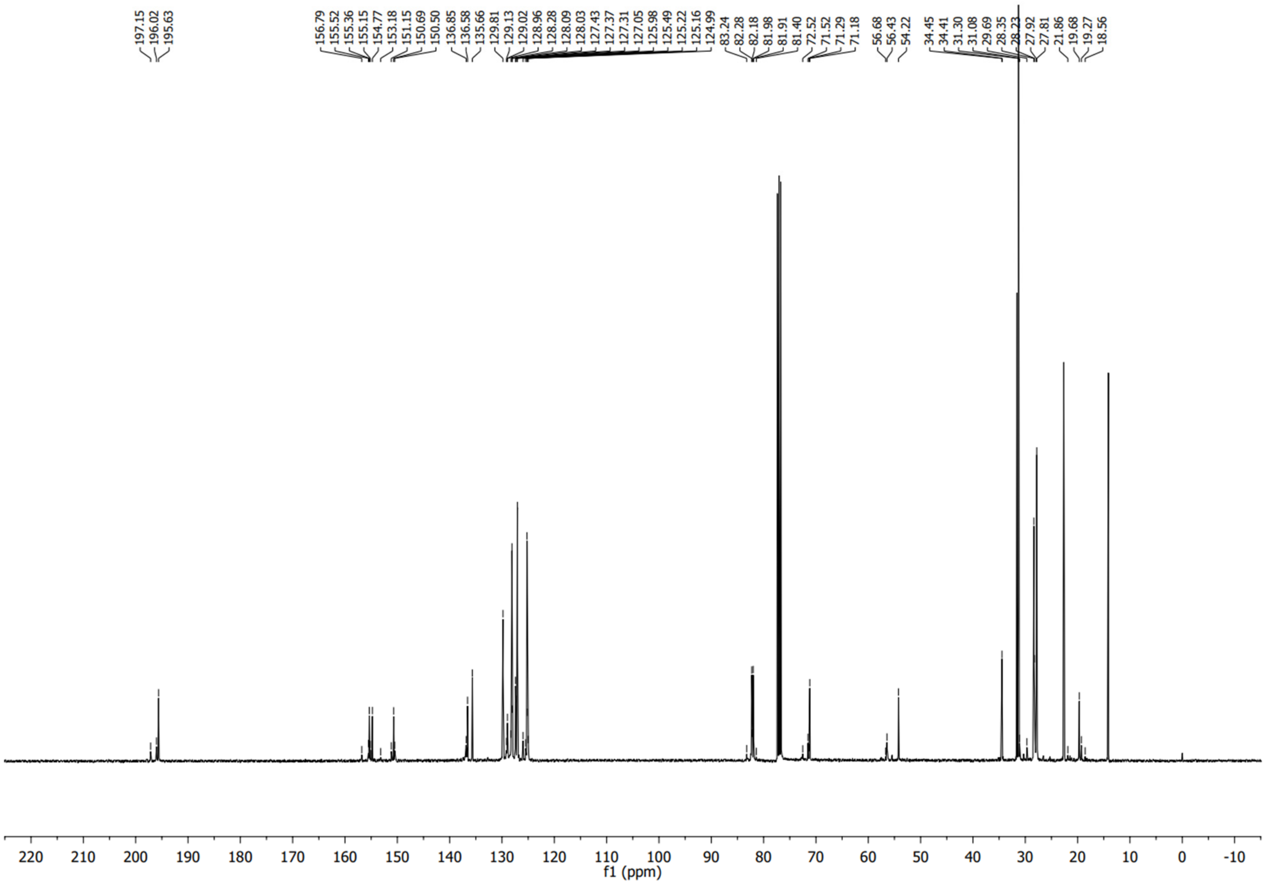
197.15 196.02 195.63
156.79 155.52 155.36 155.15 154.77 153.18 151.15 150.69 150.50 136.85 136.58 135.66 129.81 129.13 129.02 128.96 128.28 128.09 128.03 127.43 127.37 127.31 127.05 125.98 125.49 125.22 125.16 124.99 83.24 82.28 82.18 81.98 81.91 81.40 72.52 71.52 71.29 71.18
56.68 56.43 54.22
34.45 34.41 31.30 31.08 29.69 28.35 28.23 27.92 27.81 21.86 19.68 19.27 18.56
220 210 200 190 180 170 160 150 140 130 120 110 100 90 80 70 60 50 40 30 20 10 0 -10
f1 (ppm)

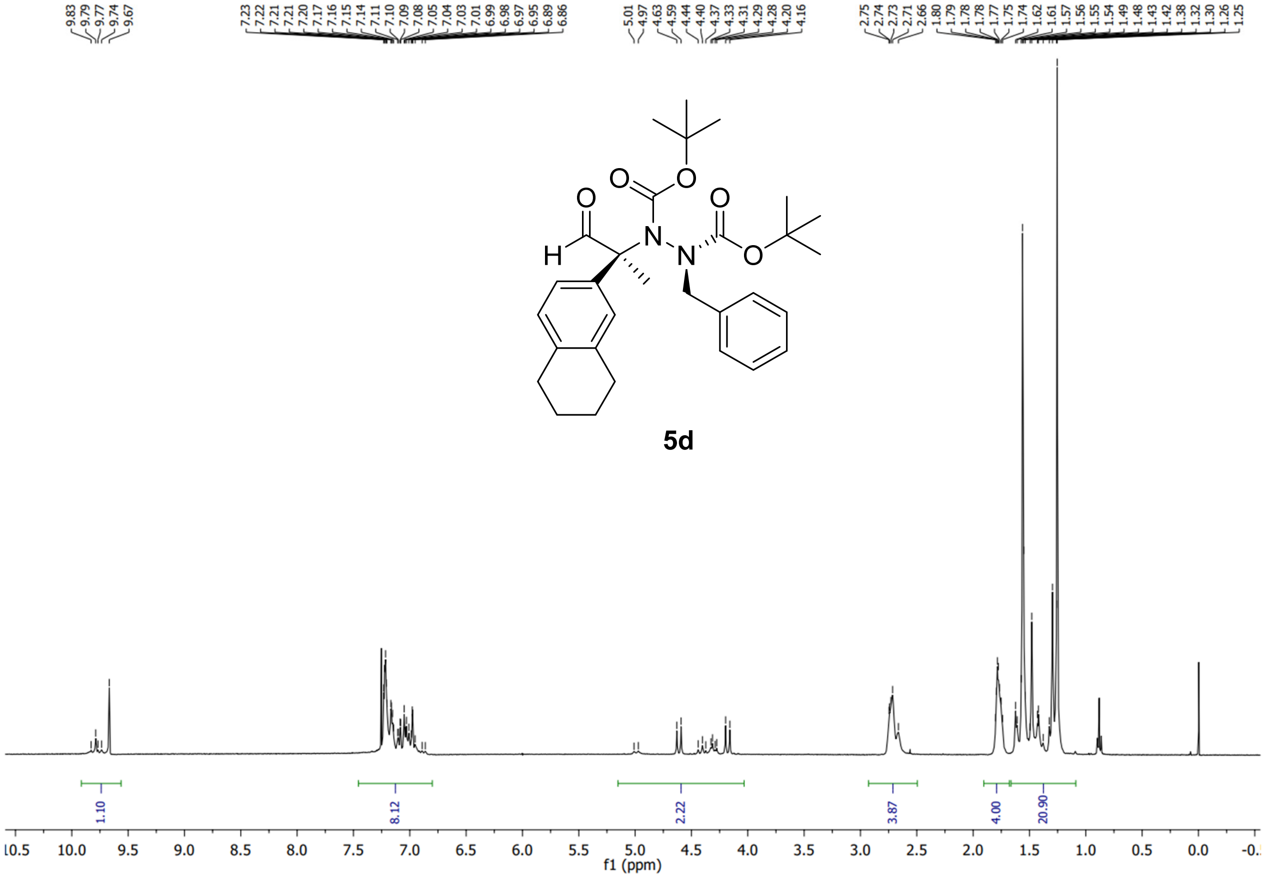
9.83 9.79 9.77 9.74 9.67
7.23 7.22 7.21 7.21 7.20 7.17 7.16 7.15 7.14 7.11 7.10 7.09 7.08 7.05 7.04 7.03 7.01 6.99 6.98 6.97 6.95 6.89 6.86
5.01 4.97 4.63 4.59 4.44 4.40 4.37 4.33 4.31 4.29 4.28 4.20 4.16
2.75 2.74 2.73 2.71 2.66 1.80 1.79 1.78 1.78 1.77 1.75 1.74 1.62 1.61 1.57 1.56 1.55 1.54 1.49 1.48 1.43 1.42 1.38 1.32 1.30 1.26 1.25
5d
1.10
8.12
2.22
3.87
4.00
20.90
10.5 10.0 9.5 9.0 8.5 8.0 7.5 7.0 6.5 6.0 5.5 5.0 4.5 4.0 3.5 3.0 2.5 2.0 1.5 1.0 0.5 0.0
f1 (ppm)

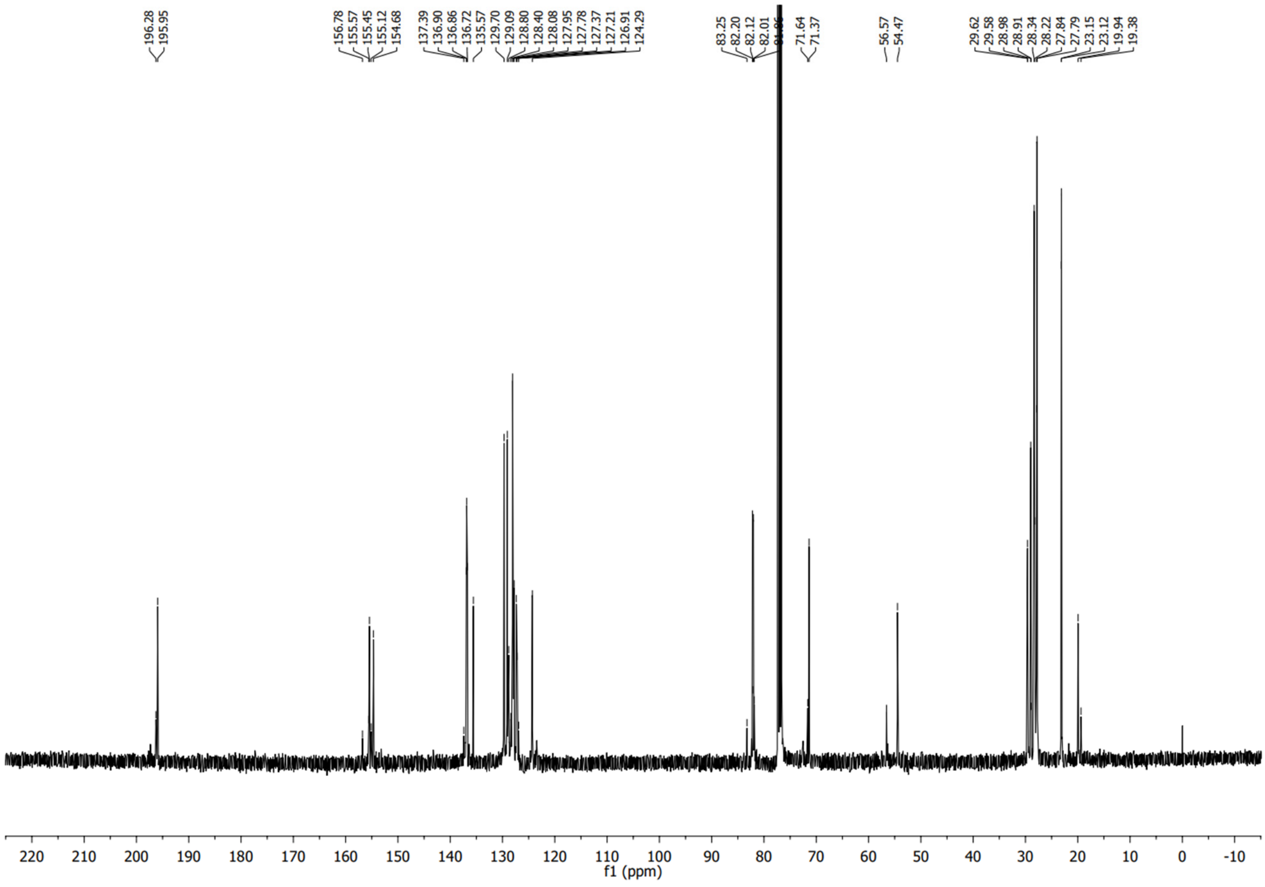
196.28 195.95
156.78 155.57 155.45 155.12 154.68
137.39 136.90 136.86 136.72 135.57 129.70 129.09 128.80 128.40 128.08 127.95 127.78 127.37 127.21 126.91 124.29
83.25 82.20 82.12 82.01 [illegible] 71.64 71.37
56.57 54.47
29.62 29.58 28.98 28.91 28.34 28.22 27.84 27.79 23.15 23.12 19.94 19.38
220 210 200 190 180 170 160 150 140 130 120 110 100 90 80 70 60 50 40 30 20 10 0 -10
f1 (ppm)

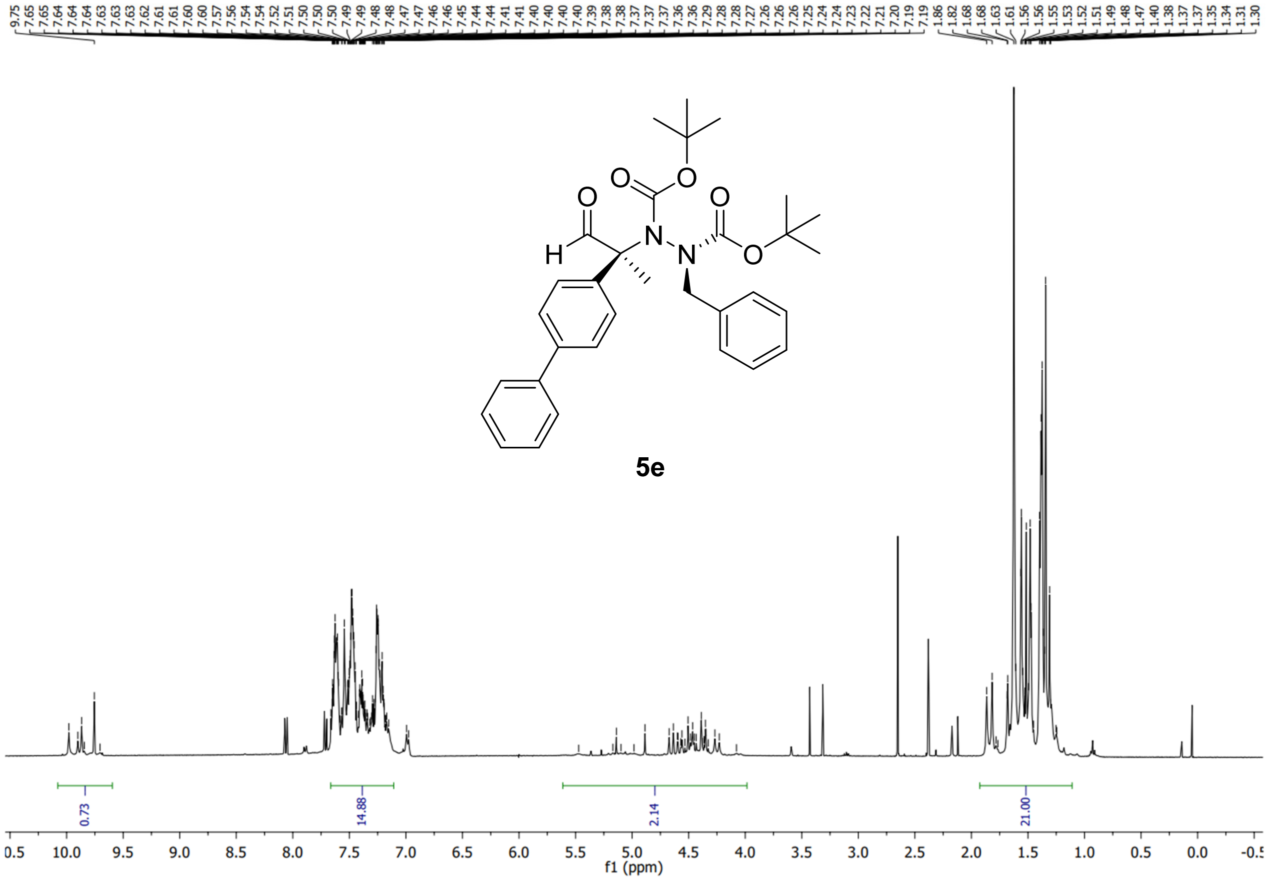
9.75 7.65 7.65 7.64 7.64 7.63 7.63 7.63 7.62 7.61 7.61 7.60 7.60 7.57 7.56 7.54 7.54 7.52 7.51 7.50 7.50 7.50 7.49 7.49 7.48 7.48 7.47 7.47 7.46 7.46 7.45 7.44 7.44 7.41 7.40 7.40 7.40 7.40 7.39 7.38 7.38 7.37 7.37 7.37 7.36 7.36 7.29 7.28 7.28 7.27 7.26 7.26 7.26 7.25 7.24 7.24 7.23 7.22 7.21 7.20 7.19 1.86 1.82 1.68 1.68 1.63 1.61 1.56 1.56 1.55 1.53 1.52 1.51 1.49 1.48 1.47 1.40 1.38 1.37 1.37 1.35 1.34 1.31 1.30
5e
0.73
14.88
2.14
21.00
f1 (ppm)

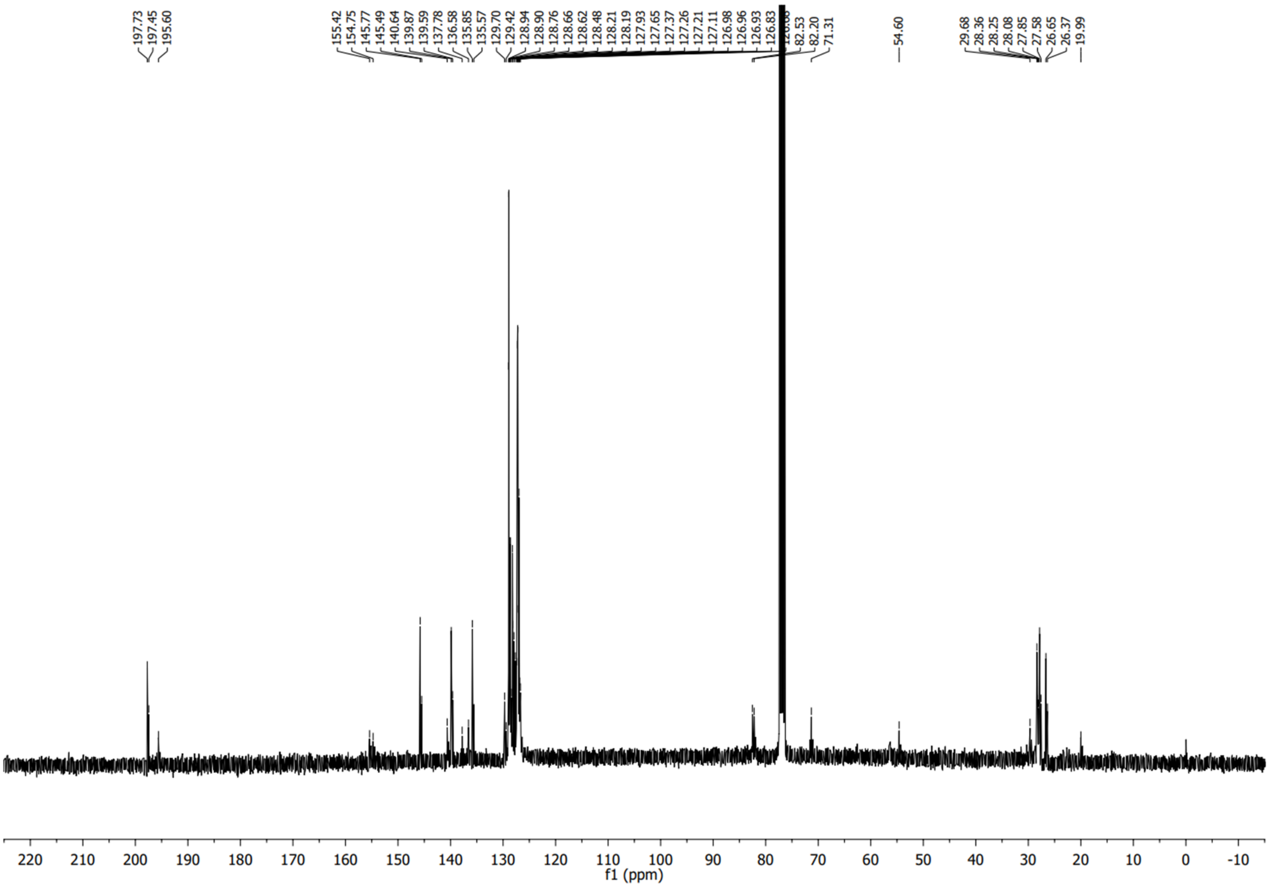
197.73 197.45 195.60
155.42 154.75 145.77 145.49 140.64 139.87 139.59 137.78 136.58 135.85 135.57 129.70 129.42 128.94 128.90 128.76 128.66 128.62 128.48 128.21 128.19 127.93 127.65 127.37 127.26 127.21 127.11 126.98 126.96 126.93 126.83 126.68 82.53 82.20 71.31
54.60
29.68 28.36 28.25 28.08 27.85 27.58 26.65 26.37 19.99
f1 (ppm)

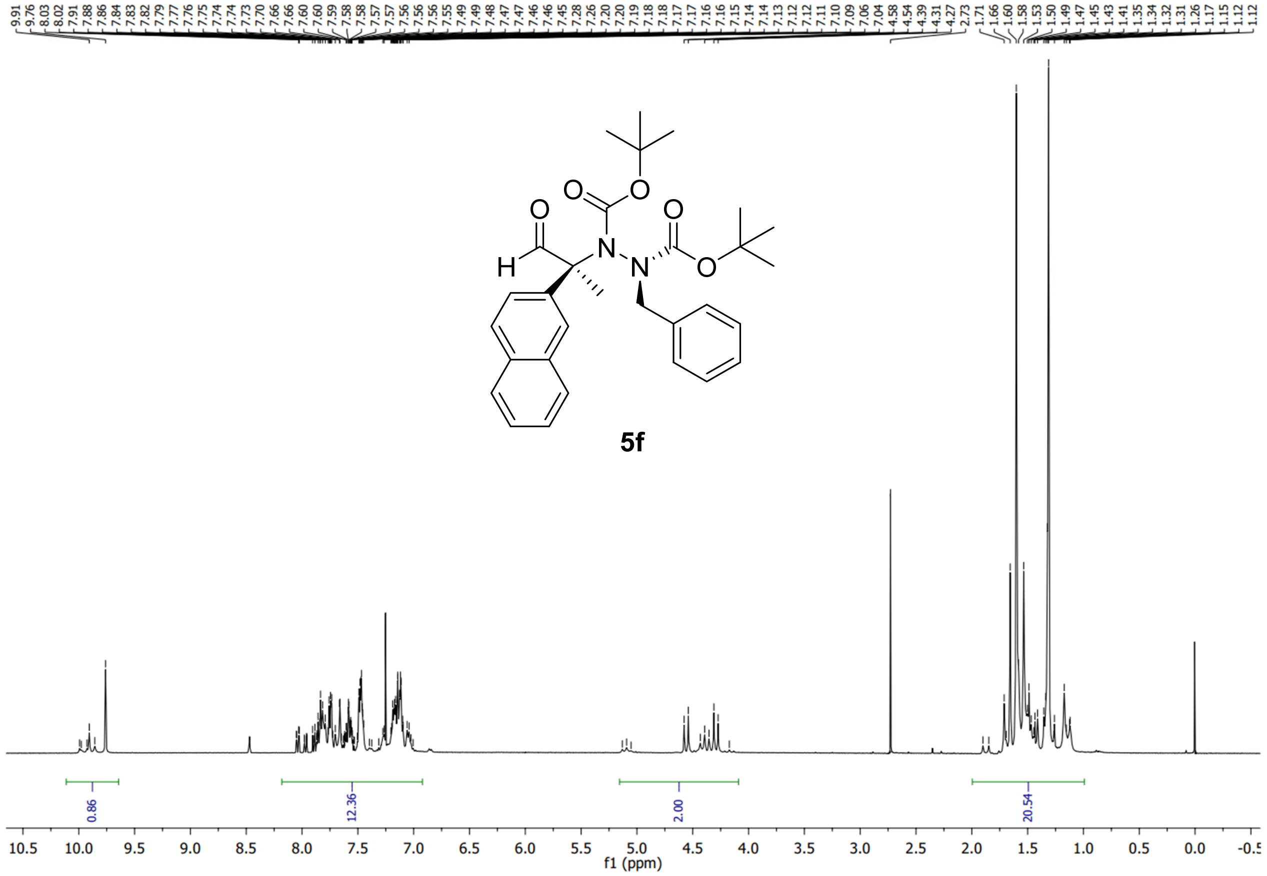
9.91 9.76 8.03 8.02 7.91 7.88 7.86 7.84 7.83 7.82 7.79 7.77 7.76 7.75 7.74 7.74 7.73 7.70 7.66 7.60 7.60 7.59 7.58 7.58 7.57 7.57 7.56 7.56 7.55 7.49 7.49 7.48 7.47 7.47 7.46 7.45 7.28 7.26 7.20 7.20 7.19 7.18 7.18 7.17 7.17 7.16 7.16 7.15 7.14 7.13 7.12 7.12 7.11 7.10 7.09 7.06 7.04 4.58 4.54 4.39 4.31 4.27 2.73 1.71 1.66 1.60 1.58 1.53 1.50 1.49 1.47 1.45 1.43 1.41 1.35 1.34 1.32 1.31 1.26 1.17 1.15 1.12 1.12
5f
0.86
12.36
2.00
20.54
10.5 10.0 9.5 9.0 8.5 8.0 7.5 7.0 6.5 6.0 5.5 5.0 4.5 4.0 3.5 3.0 2.5 2.0 1.5 1.0 0.5 0.0 -0.5
f1 (ppm)

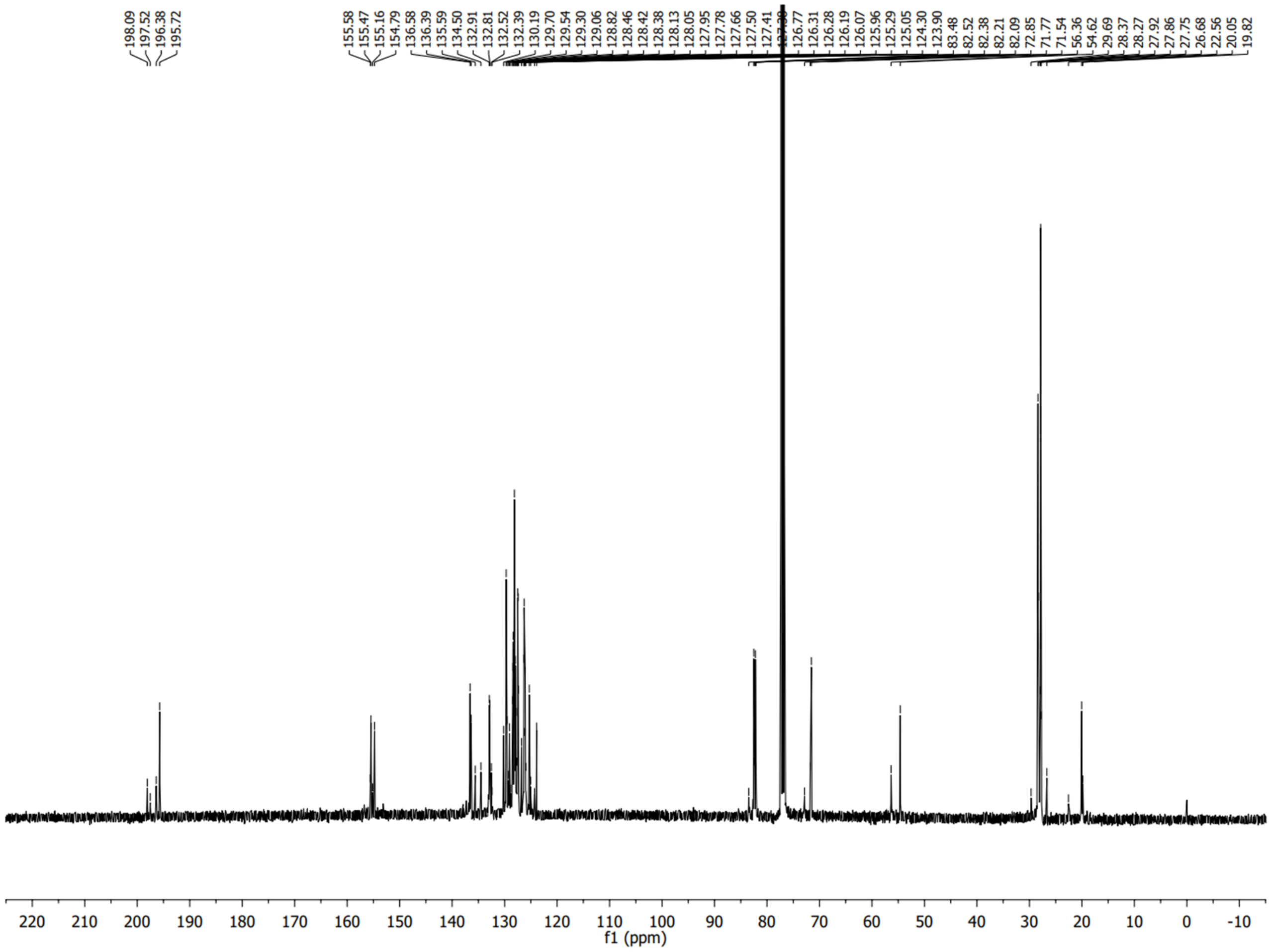
198.09 197.52 196.38 195.72
155.58 155.47 155.16 154.79 136.58 136.39 135.59 134.50 132.91 132.81 132.52 132.39 130.19 129.70 129.54 129.30 129.06 128.82 128.46 128.42 128.38 128.13 128.05 127.95 127.78 127.66 127.50 127.41 126.77 126.31 126.28 126.19 126.07 125.96 125.29 125.05 124.30 123.90 83.48 82.52 82.38 82.21 82.09 72.85 71.77 71.54 56.36 54.62 29.69 28.37 28.27 27.92 27.86 27.75 26.68 22.56 20.05 19.82
220 210 200 190 180 170 160 150 140 130 120 110 100 90 80 70 60 50 40 30 20 10 0 -10
f1 (ppm)

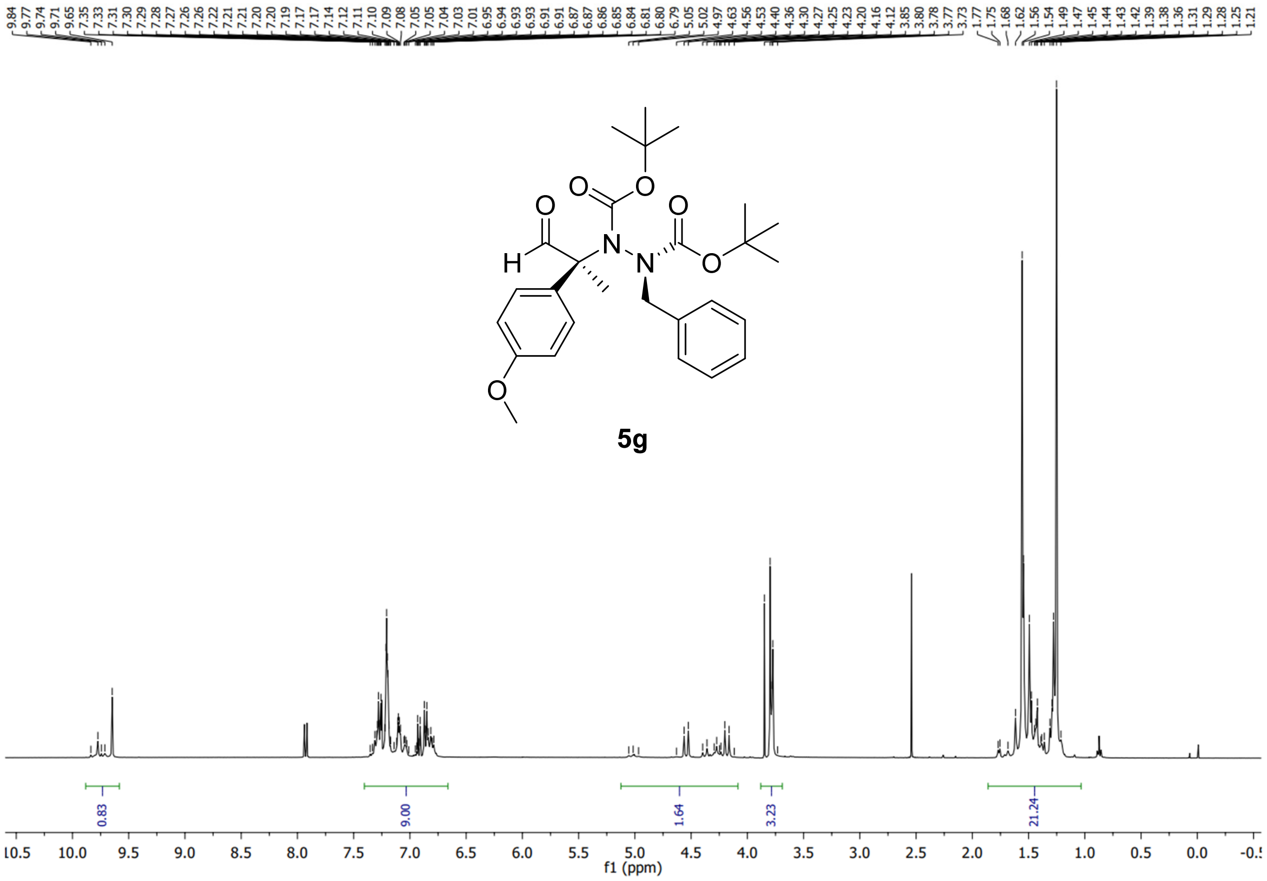
9.84 9.77 9.74 9.71 9.65 7.35 7.33 7.31 7.30 7.29 7.28 7.27 7.26 7.26 7.22 7.21 7.21 7.20 7.19 7.17 7.17 7.14 7.12 7.11 7.10 7.09 7.08 7.05 7.05 7.04 7.03 7.01 6.95 6.94 6.93 6.93 6.91 6.91 6.87 6.87 6.86 6.85 6.85 6.84 6.81 6.80 6.79 5.05 5.02 4.97 4.63 4.56 4.53 4.40 4.36 4.30 4.27 4.25 4.23 4.20 4.16 4.12 3.85 3.80 3.78 3.77 3.73 1.77 1.75 1.68 1.62 1.56 1.54 1.49 1.47 1.45 1.44 1.43 1.42 1.39 1.38 1.36 1.31 1.29 1.28 1.25 1.21
5g
0.83
9.00
1.64
3.23
21.24
10.5 10.0 9.5 9.0 8.5 8.0 7.5 7.0 6.5 6.0 5.5 5.0 4.5 4.0 3.5 3.0 2.5 2.0 1.5 1.0 0.5 0.0 -0.5
f1 (ppm)

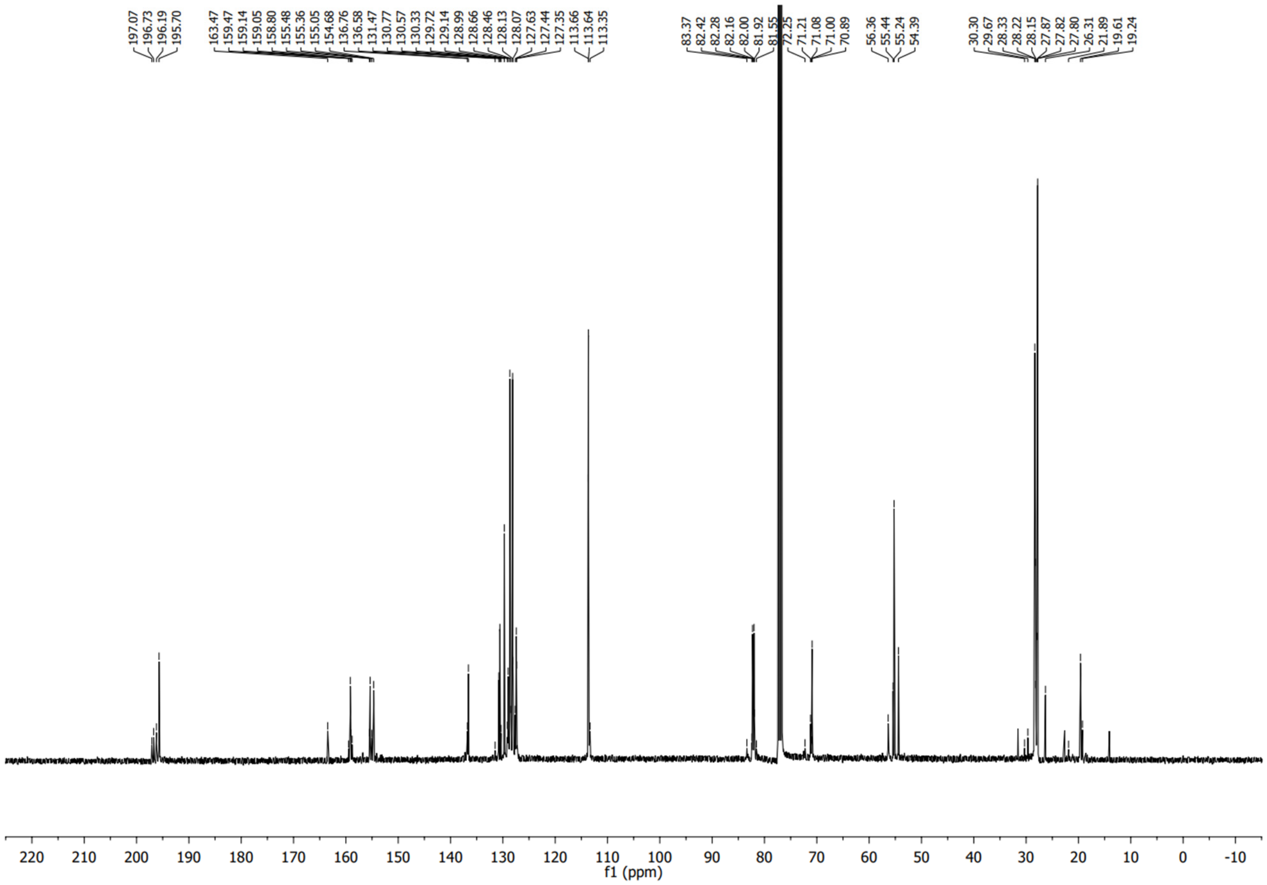
197.07 196.73 196.19 195.70 163.47 159.47 159.14 159.05 158.80 155.48 155.36 155.05 154.68 136.76 136.58 131.47 130.77 130.57 130.33 129.72 129.14 128.99 128.66 128.46 128.13 128.07 127.63 127.44 127.35 113.66 113.64 113.35 83.37 82.42 82.28 82.16 82.00 81.92 81.55 72.25 71.21 71.08 71.00 70.89 56.36 55.44 55.24 54.39 30.30 29.67 28.33 28.22 28.15 27.87 27.82 27.80 26.31 21.89 19.61 19.24
220 210 200 190 180 170 160 150 140 130 120 110 100 90 80 70 60 50 40 30 20 10 0 -10
f1 (ppm)

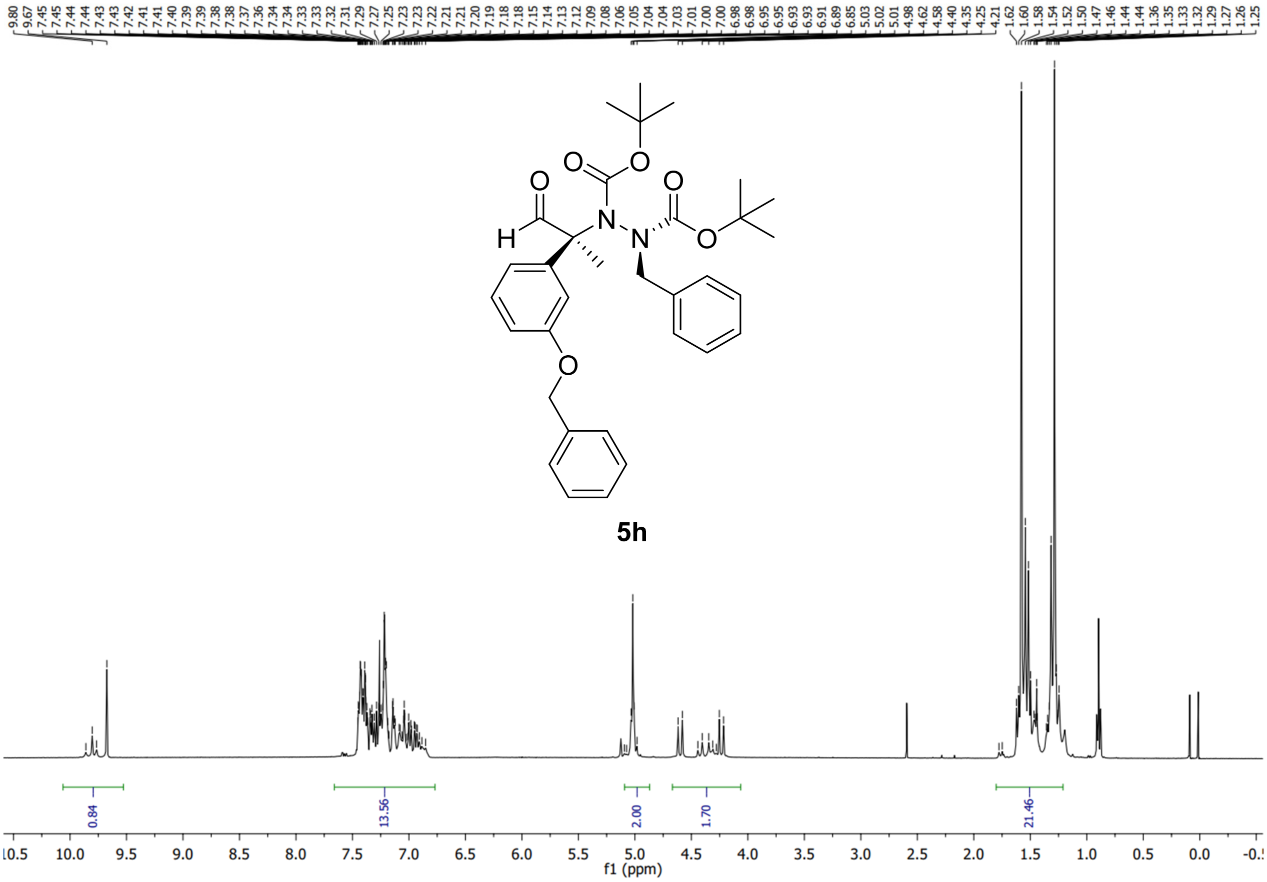
9.80 9.67 7.45 7.45 7.44 7.44 7.43 7.43 7.42 7.41 7.41 7.40 7.39 7.39 7.38 7.38 7.37 7.36 7.34 7.34 7.33 7.33 7.32 7.31 7.29 7.27 7.25 7.23 7.23 7.22 7.21 7.21 7.20 7.19 7.18 7.18 7.15 7.14 7.13 7.12 7.09 7.08 7.06 7.05 7.04 7.04 7.03 7.01 7.00 7.00 6.98 6.98 6.95 6.95 6.93 6.93 6.91 6.89 6.85 5.03 5.02 5.01 4.98 4.62 4.58 4.40 4.35 4.25 4.21 1.62 1.60 1.58 1.54 1.52 1.50 1.47 1.46 1.44 1.36 1.35 1.33 1.32 1.29 1.27 1.26 1.25
5h
0.84
13.56
2.00
1.70
21.46
f1 (ppm)

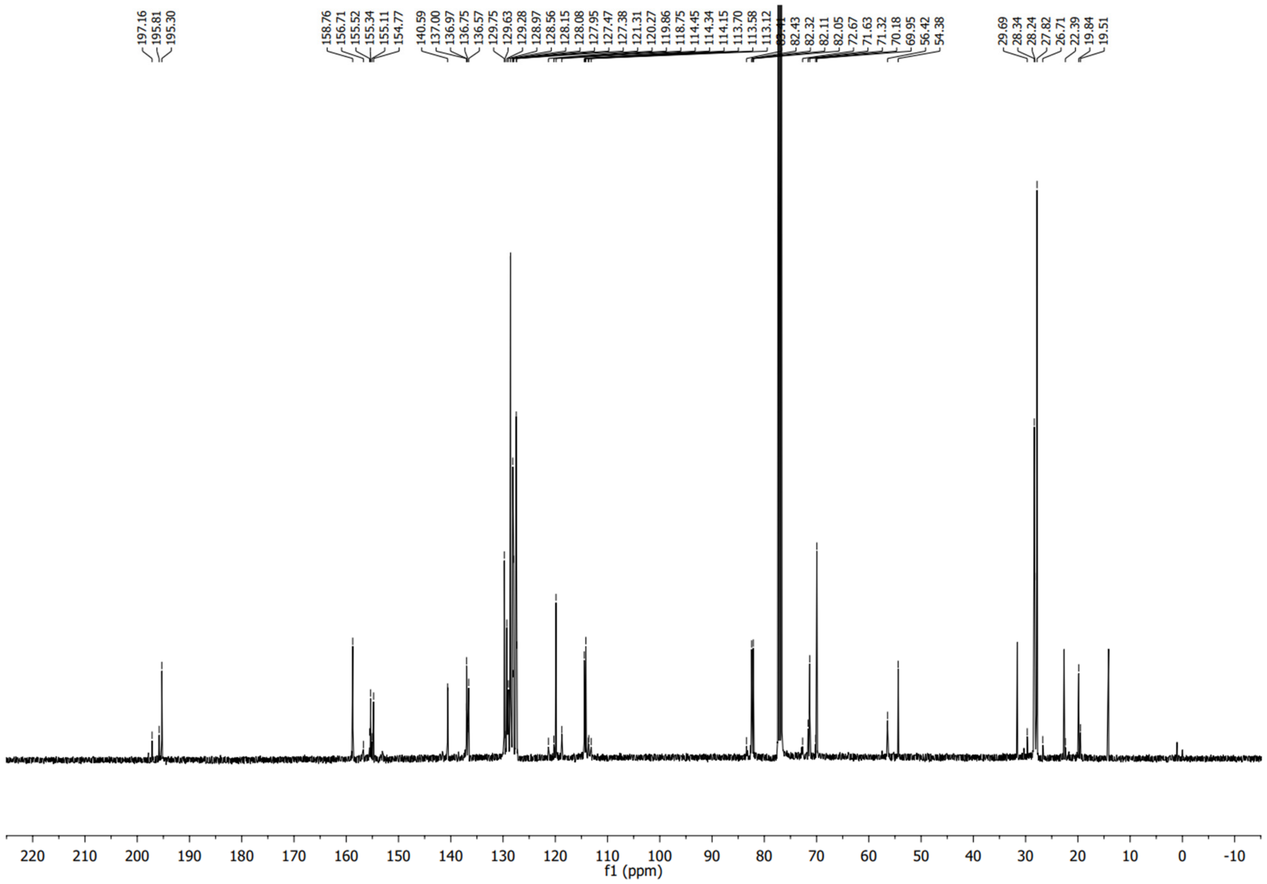
197.16 195.81 195.30 158.76 156.71 155.52 155.34 155.11 154.77 140.59 137.00 136.97 136.75 136.57 129.75 129.63 129.28 128.97 128.56 128.15 128.08 127.95 127.47 127.38 121.31 120.27 119.86 118.75 114.45 114.34 114.15 113.70 113.58 113.12 82.43 82.32 82.11 82.05 72.67 71.63 71.32 70.18 69.95 56.42 54.38 29.69 28.34 28.24 27.82 26.71 22.39 19.84 19.51
f1 (ppm)

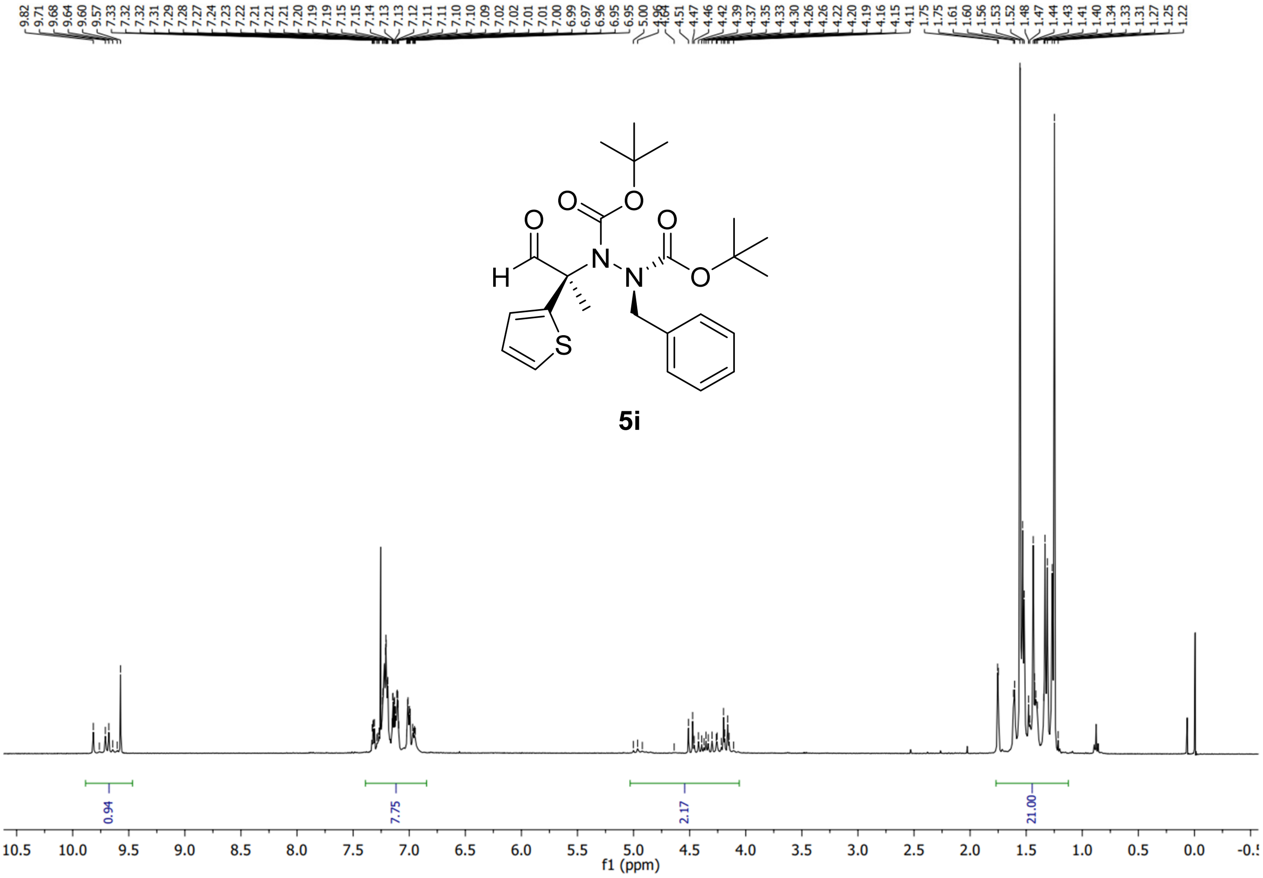
5i
f1 (ppm)

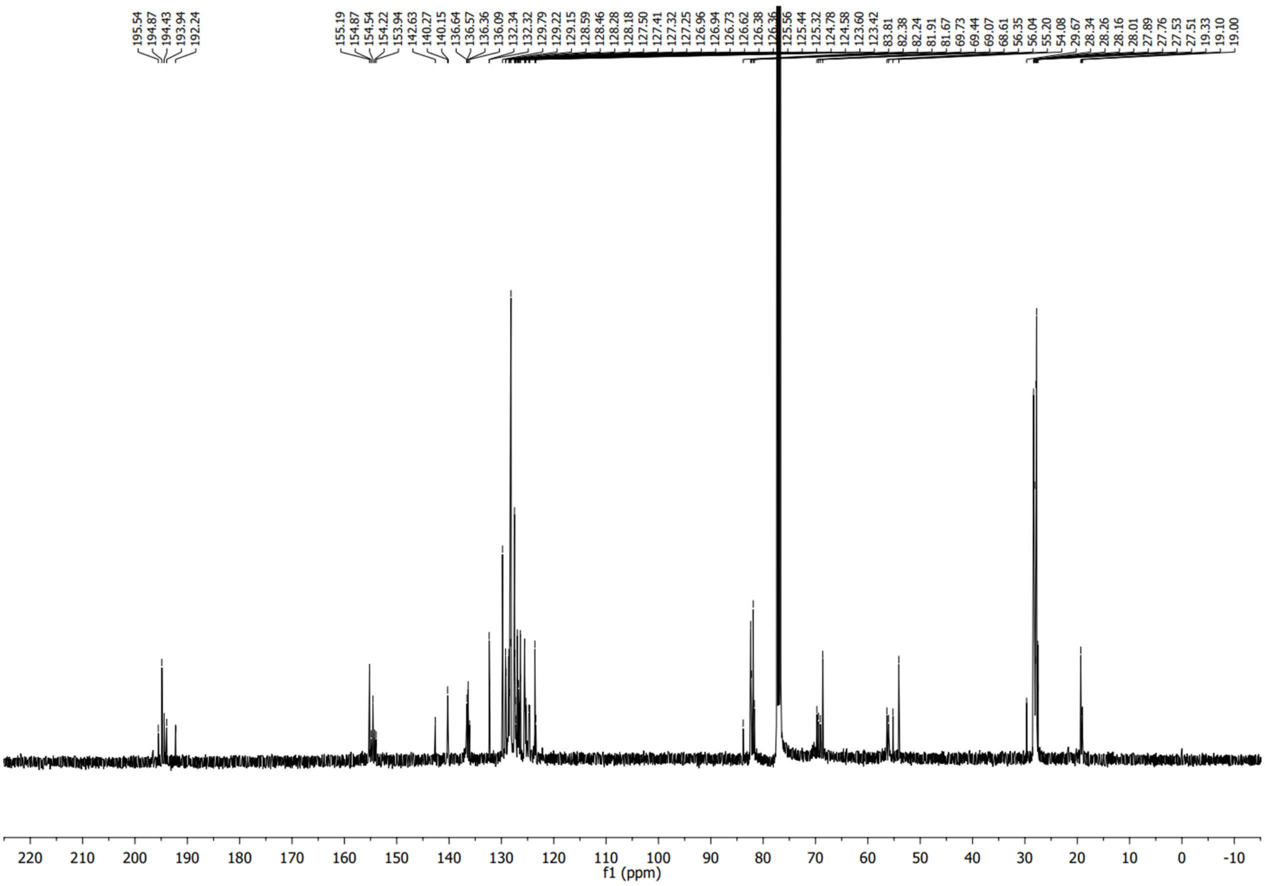
f1 (ppm)

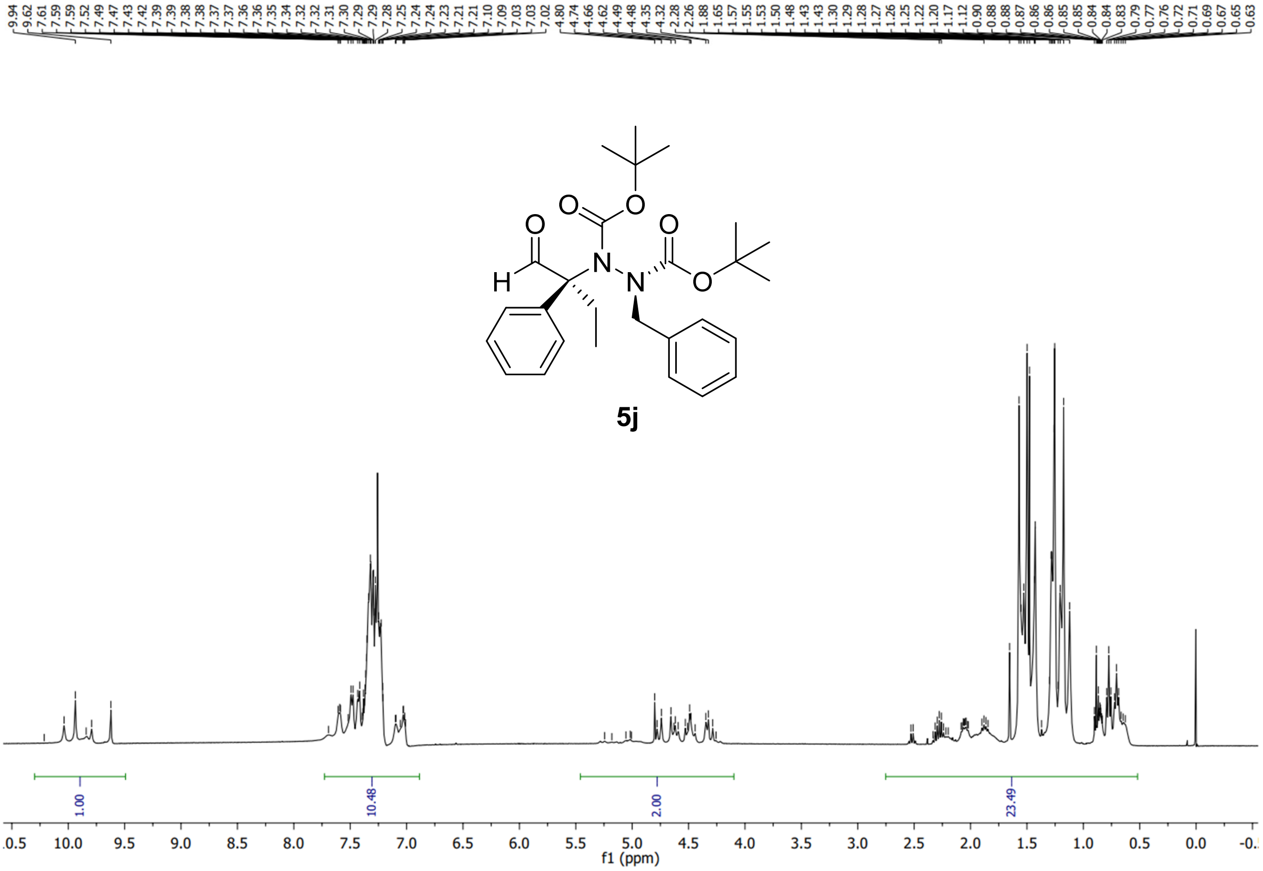
9.94 9.62 7.61 7.59 7.59 7.52 7.49 7.47 7.43 7.42 7.39 7.39 7.38 7.38 7.37 7.37 7.36 7.36 7.35 7.34 7.32 7.32 7.31 7.30 7.29 7.29 7.28 7.25 7.24 7.24 7.23 7.21 7.21 7.10 7.09 7.03 7.02
4.80 4.74 4.66 4.62 4.49 4.48 4.35 4.32 2.28 2.26 1.88 1.65 1.57 1.55 1.53 1.50 1.48 1.43 1.43 1.30 1.29 1.28 1.27 1.26 1.25 1.22 1.20 1.17 1.12 0.90 0.88 0.88 0.87 0.86 0.86 0.85 0.85 0.84 0.84 0.83 0.79 0.77 0.76 0.72 0.71 0.69 0.67 0.65 0.63
O
O
O
O
O
H
N
N
5j
1.00
10.48
2.00
23.49
f1 (ppm)

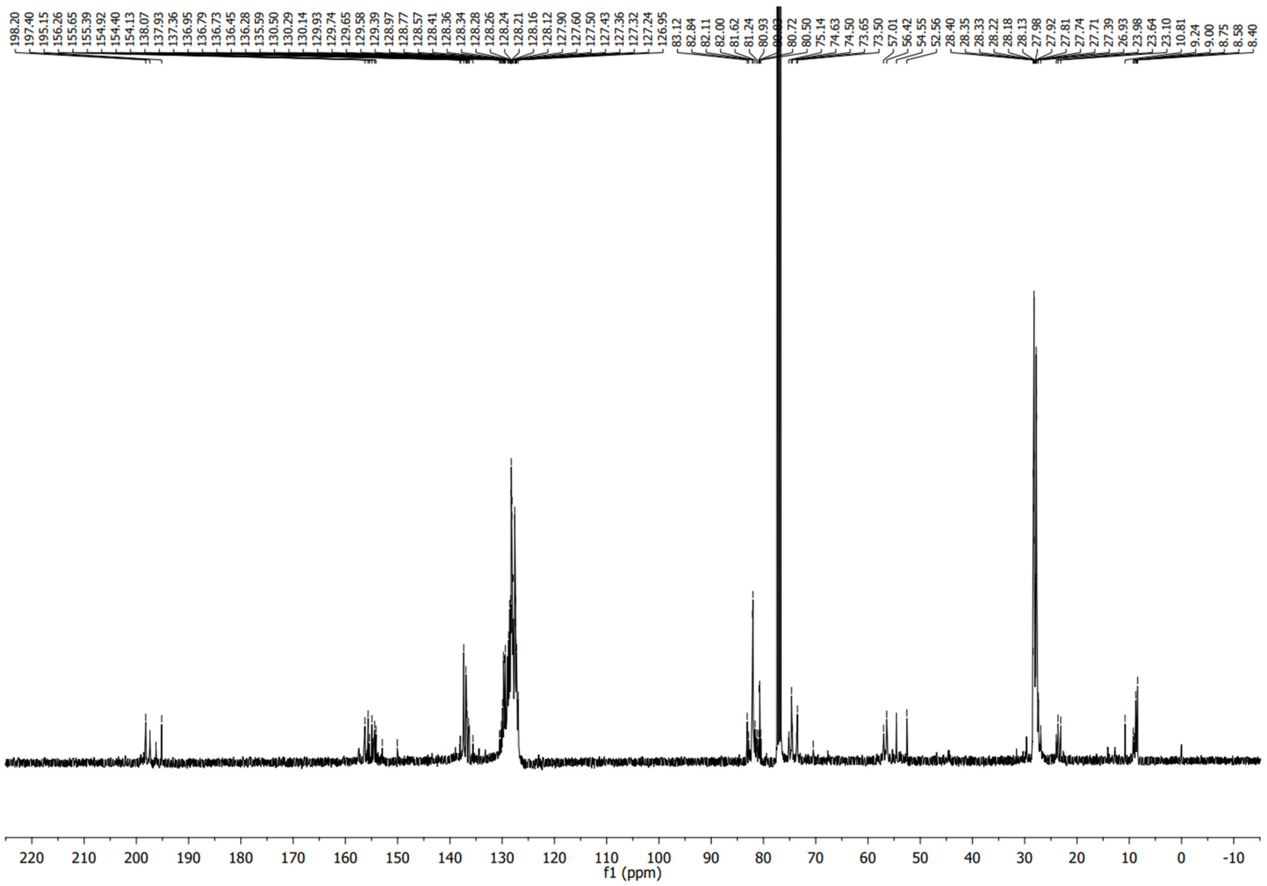
198.20 197.40 195.15 156.26 155.65 155.39 154.92 154.40 154.13 138.07 137.93 137.36 136.95 136.79 136.73 136.45 136.28 135.59 130.50 130.29 130.14 129.93 129.74 129.65 129.58 129.39 128.97 128.77 128.57 128.41 128.36 128.34 128.28 128.26 128.24 128.21 128.16 128.12 127.90 127.60 127.50 127.43 127.36 127.32 127.24 126.95
83.12 82.84 82.11 82.00 81.62 81.24 80.93 80.72 80.50 75.14 74.63 74.50 73.65 73.50 57.01 56.42 54.55 52.56 28.40 28.35 28.33 28.22 28.18 28.13 27.98 27.92 27.81 27.74 27.71 27.39 26.93 23.98 23.64 23.10 10.81 9.24 9.00 8.75 8.58 8.40
f1 (ppm)

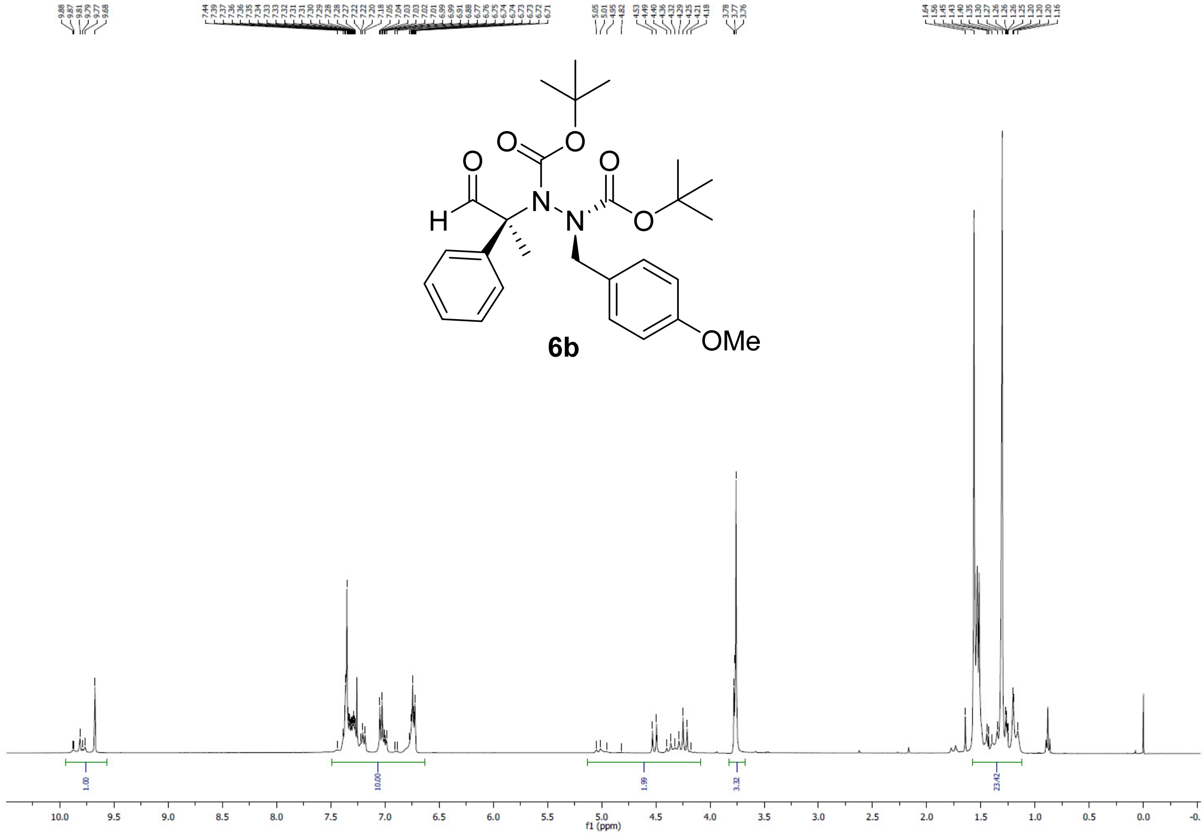
6b
OMe
f1 (ppm)

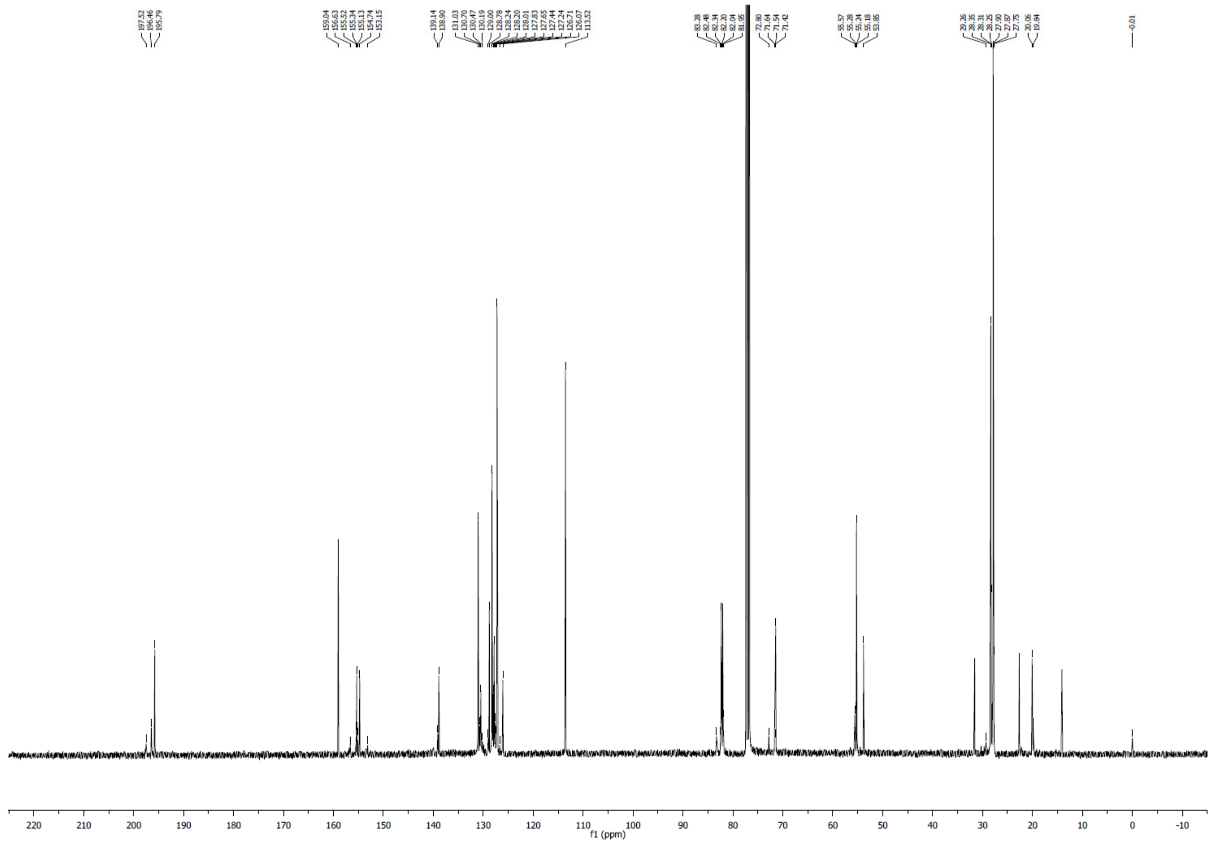
f1 (ppm)

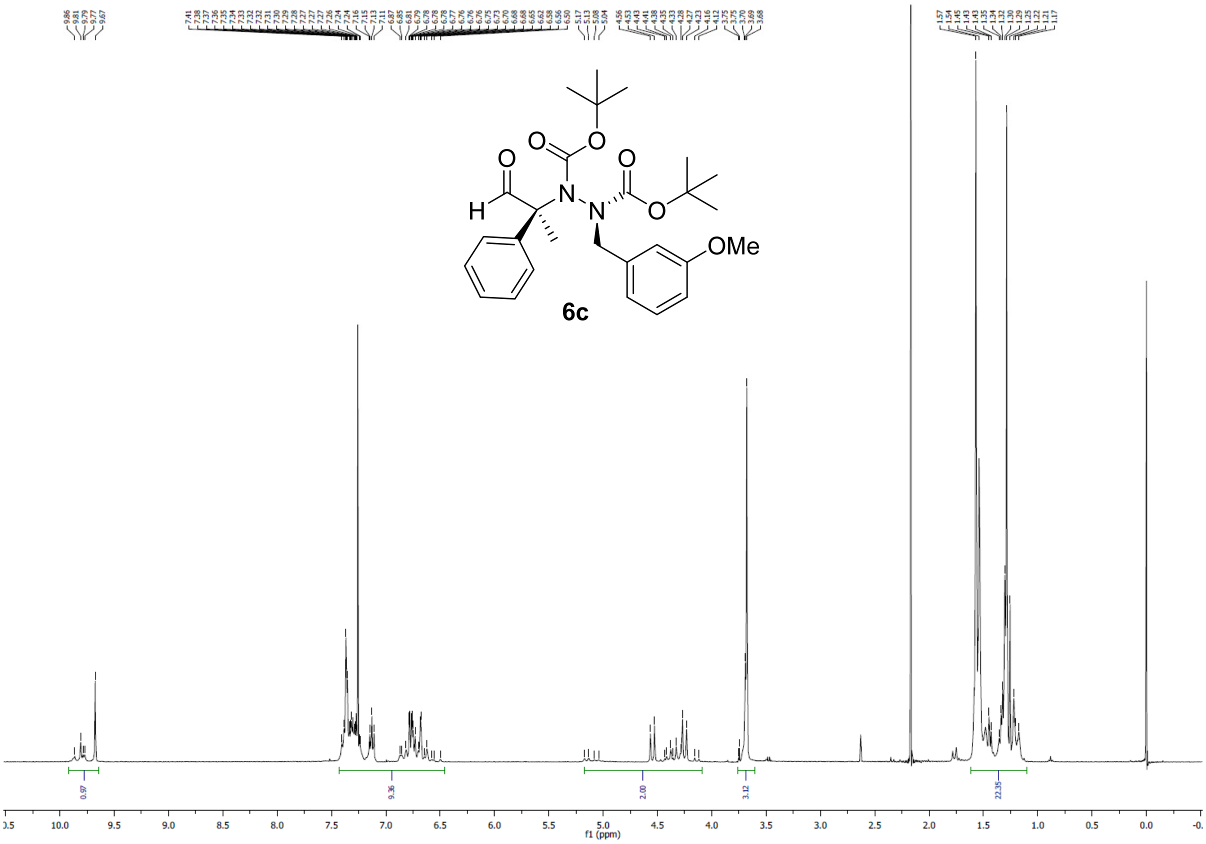

O
H
N
OMe
6c
f1 (ppm)

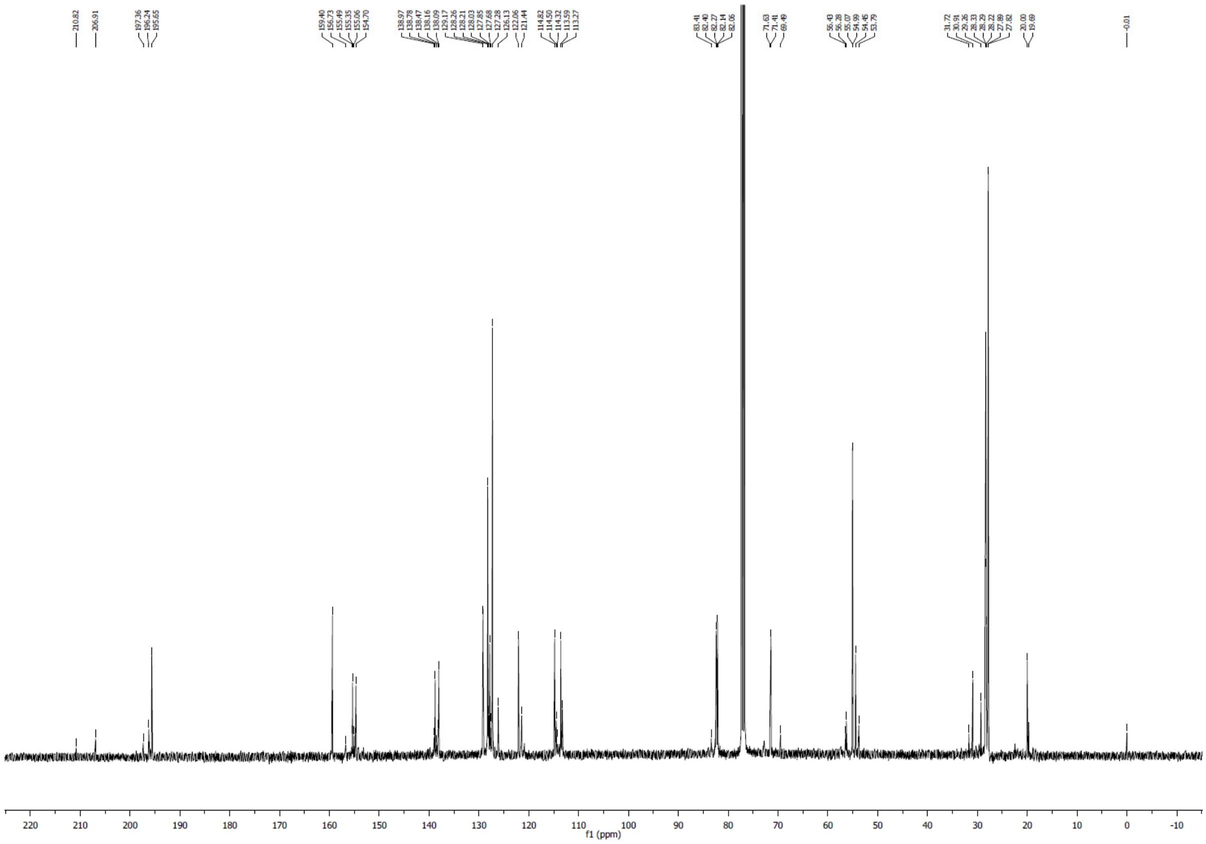

f1 (ppm)

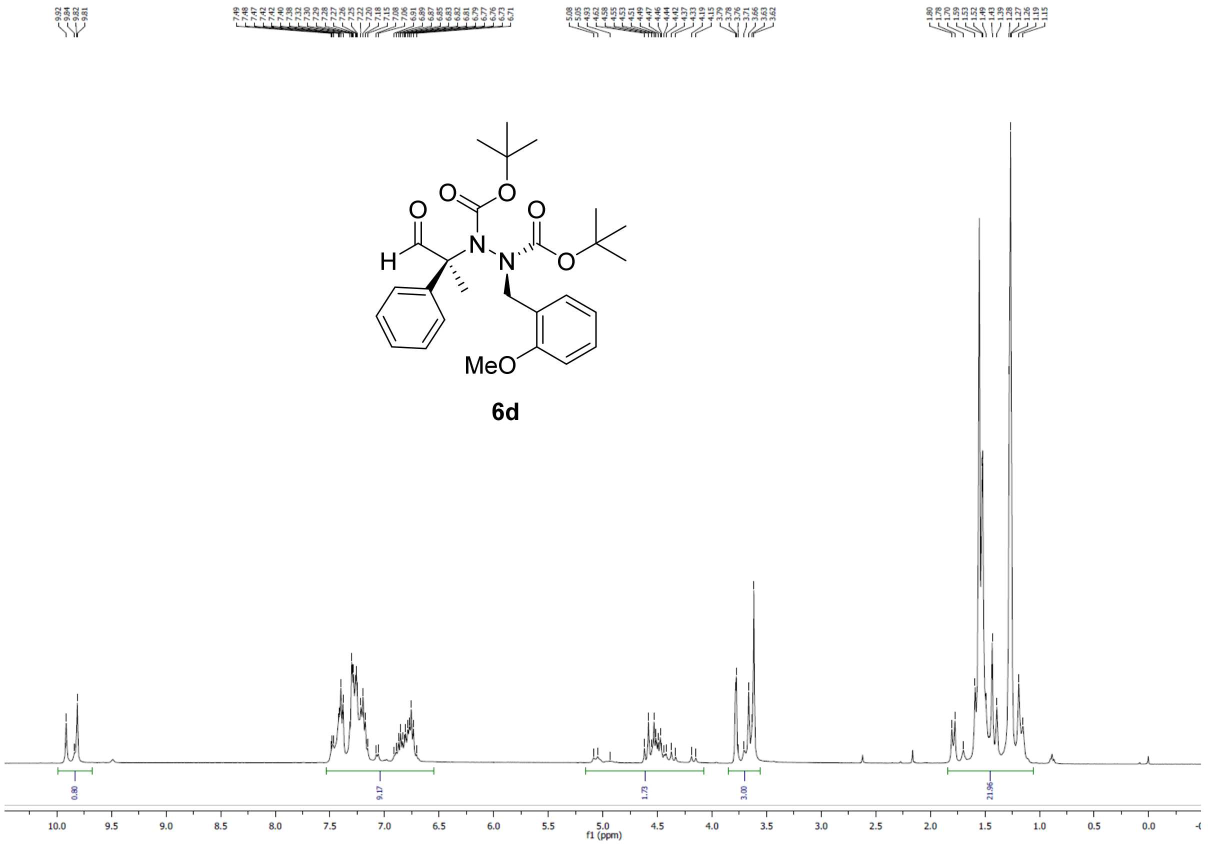
MeO
6d
f1 (ppm)

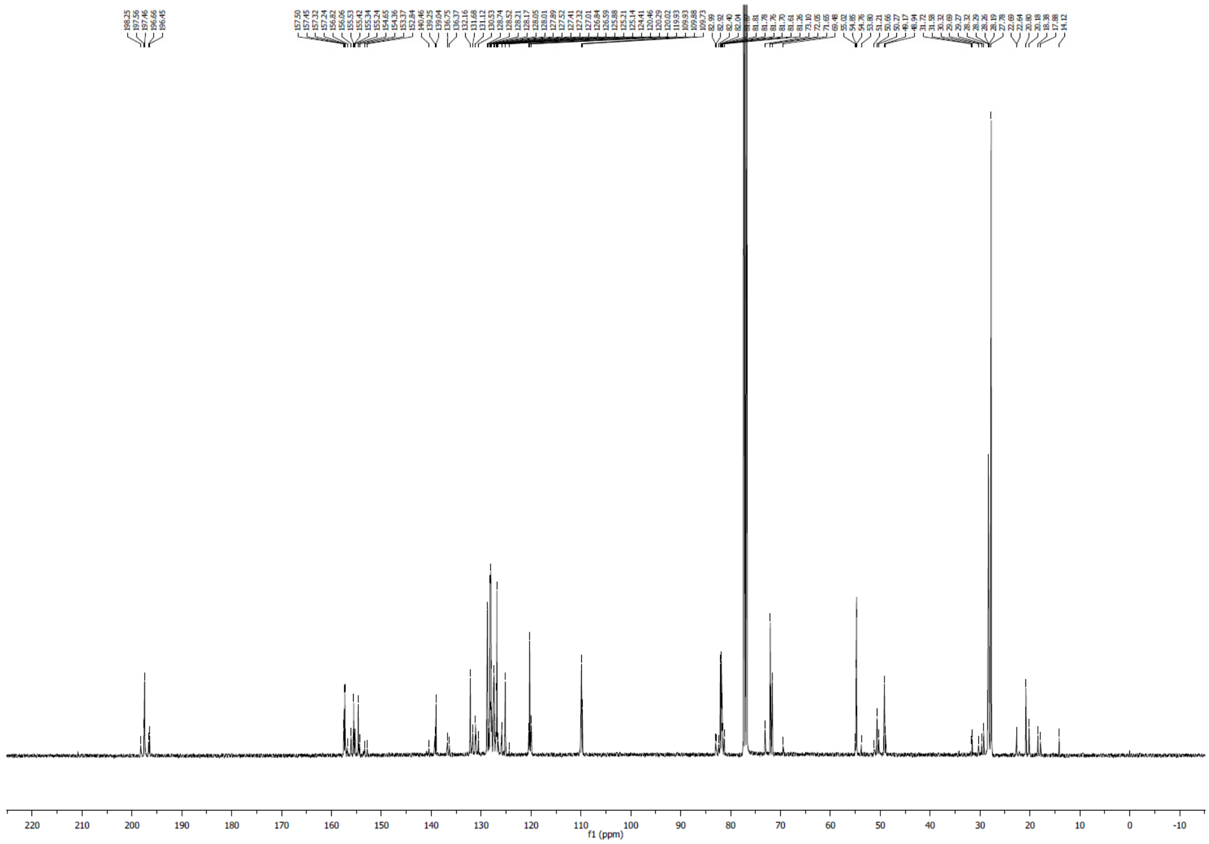
f1 (ppm)

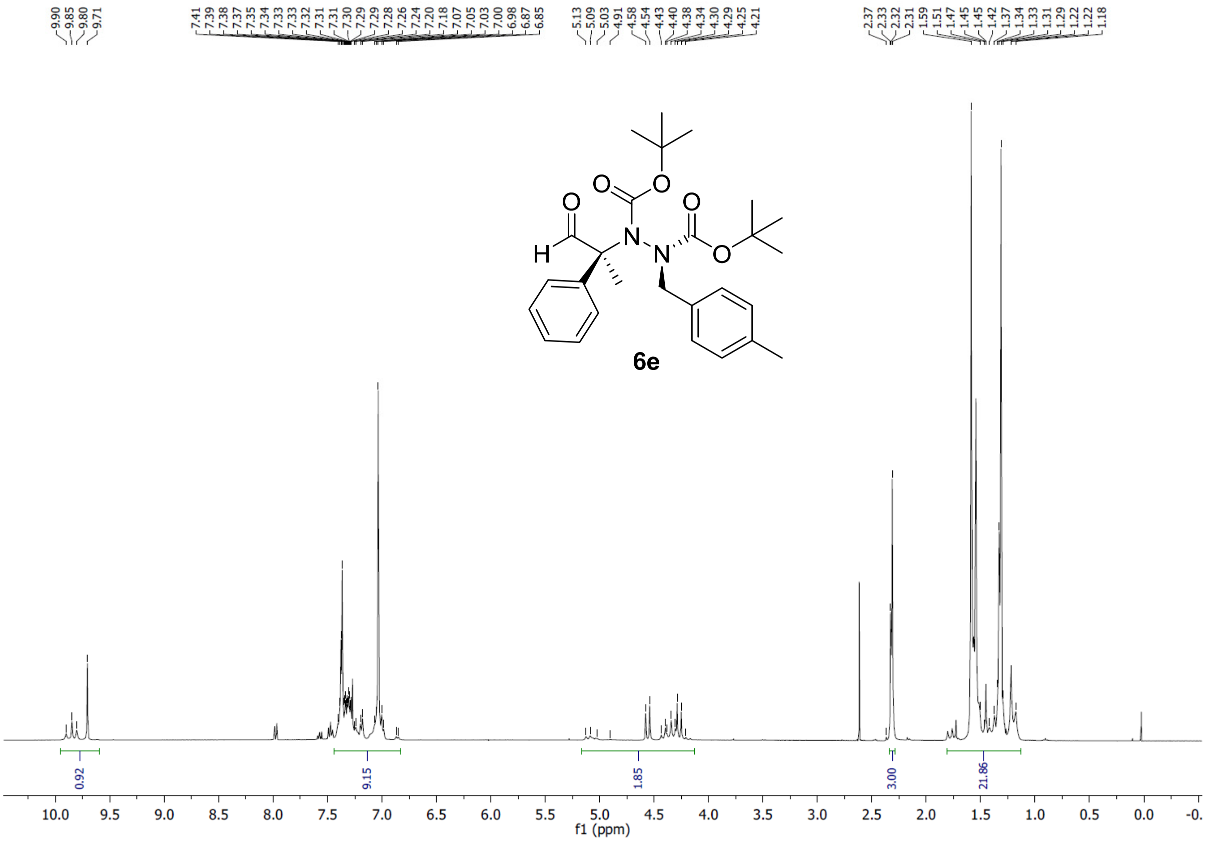
9.90
9.85
9.80
9.71
7.41 7.39 7.38 7.37 7.35 7.34 7.33 7.33 7.32 7.31 7.31 7.30 7.29 7.29 7.28 7.26 7.24 7.20 7.18 7.07 7.05 7.03 7.00 6.98 6.87 6.85
5.13 5.09 5.03 4.91 4.58 4.54 4.43 4.40 4.38 4.34 4.30 4.29 4.25 4.21
2.37 2.33 2.32 2.31 1.59 1.51 1.47 1.45 1.45 1.42 1.37 1.34 1.33 1.31 1.29 1.22 1.22 1.18
O
O
O
O
O
H
N
N
6e
0.92
9.15
1.85
3.00
21.86
10.0 9.5 9.0 8.5 8.0 7.5 7.0 6.5 6.0 5.5 5.0 4.5 4.0 3.5 3.0 2.5 2.0 1.5 1.0 0.5 0.0 -0.
f1 (ppm)

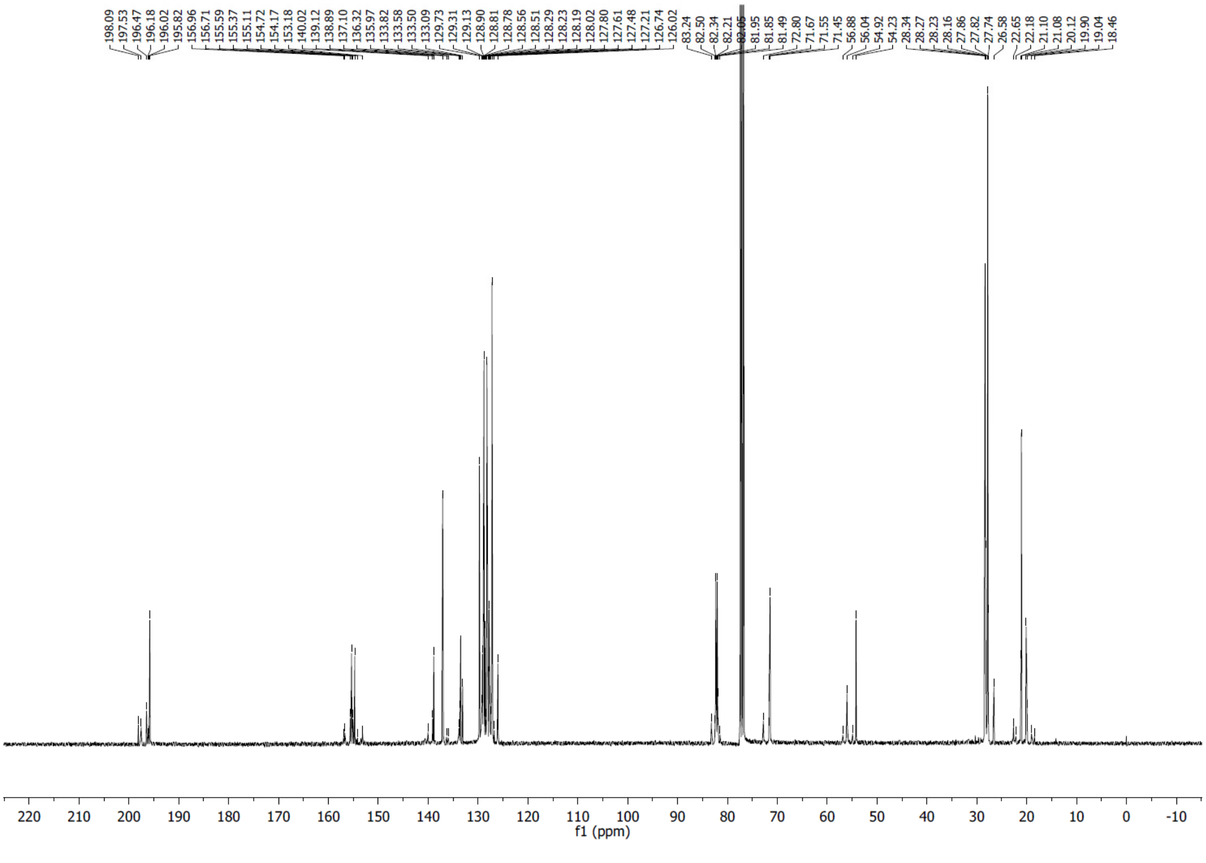
198.09 197.53 196.47 196.18 196.02 195.82
156.96 156.71 155.59 155.37 155.11 154.72 154.17 153.18 140.02 139.12 138.89 137.10 136.32 135.97 133.82 133.58 133.50 133.09 129.73 129.31 129.13 128.90 128.81 128.78 128.56 128.51 128.29 128.23 128.19 128.02 127.80 127.61 127.48 127.21 126.74 126.02
83.24 82.50 82.34 82.21 81.95 81.85 81.49 72.80 71.67 71.55 71.45
56.88 56.04 54.92 54.23
28.34 28.27 28.23 28.16 27.86 27.82 27.74 26.58 22.65 22.18 21.10 21.08 20.12 19.90 19.04 18.46
220 210 200 190 180 170 160 150 140 130 120 110 100 90 80 70 60 50 40 30 20 10 0 -10
f1 (ppm)

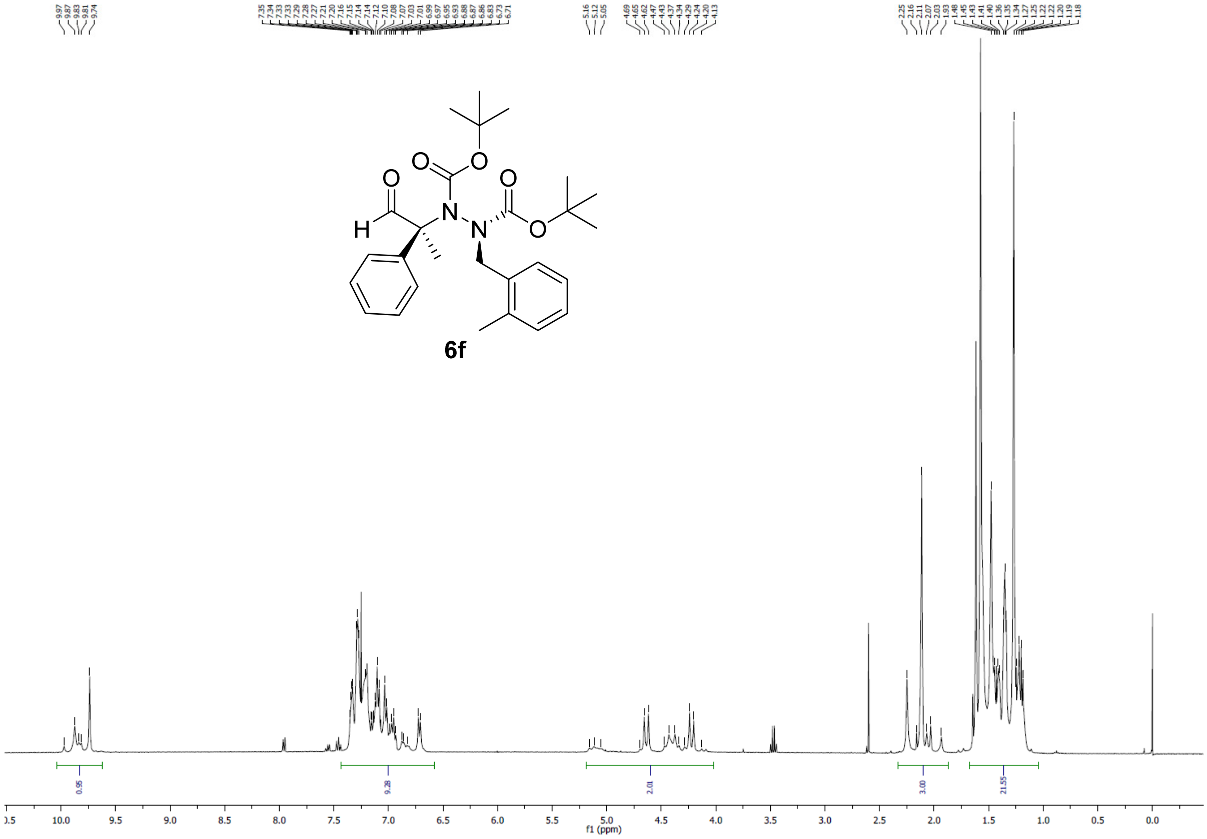

6f

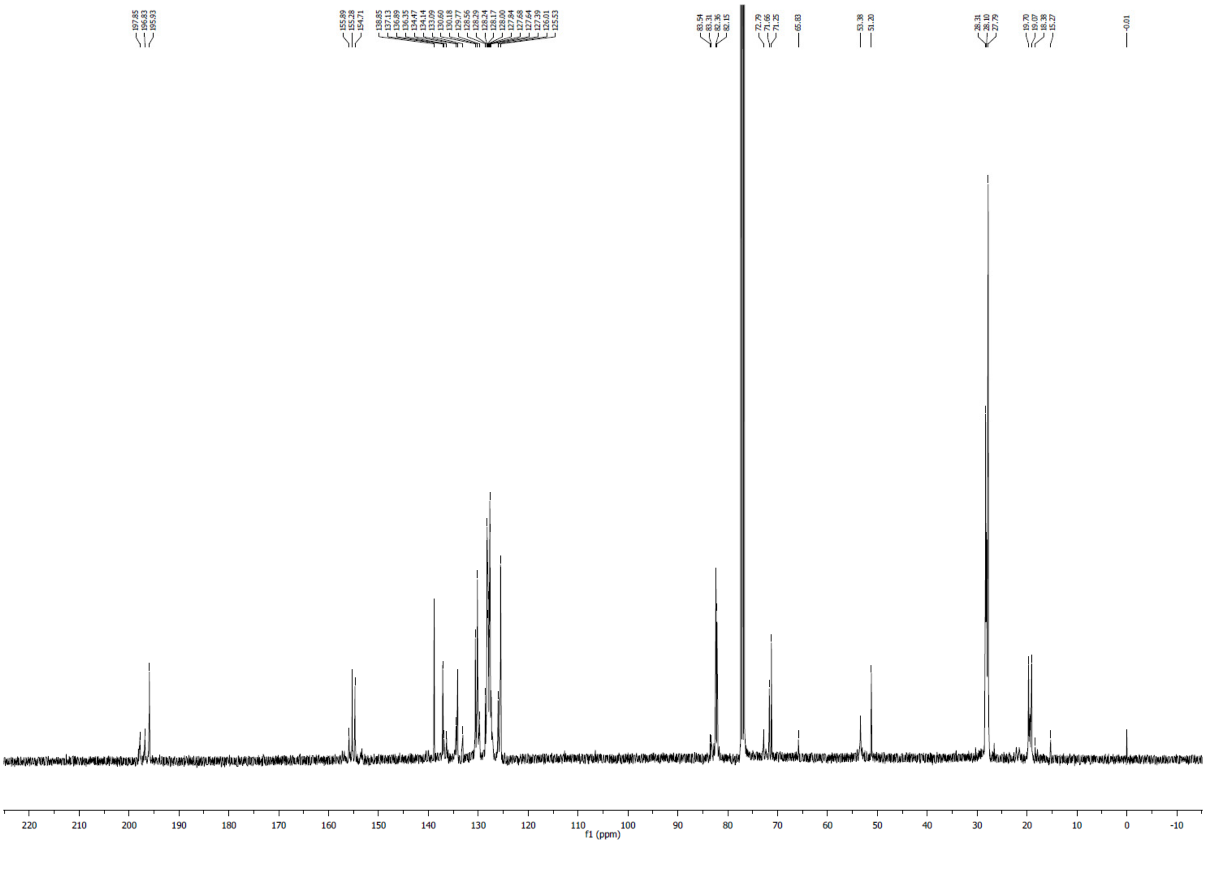

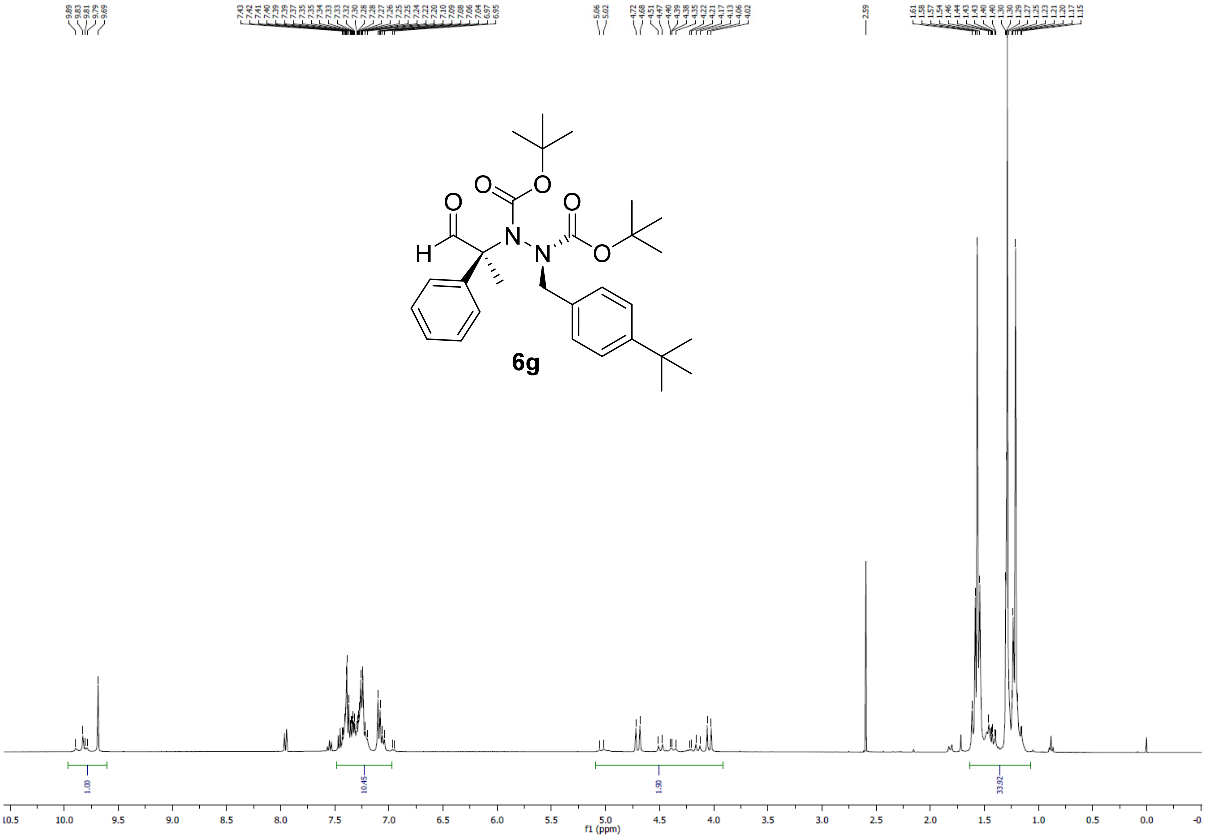

6g

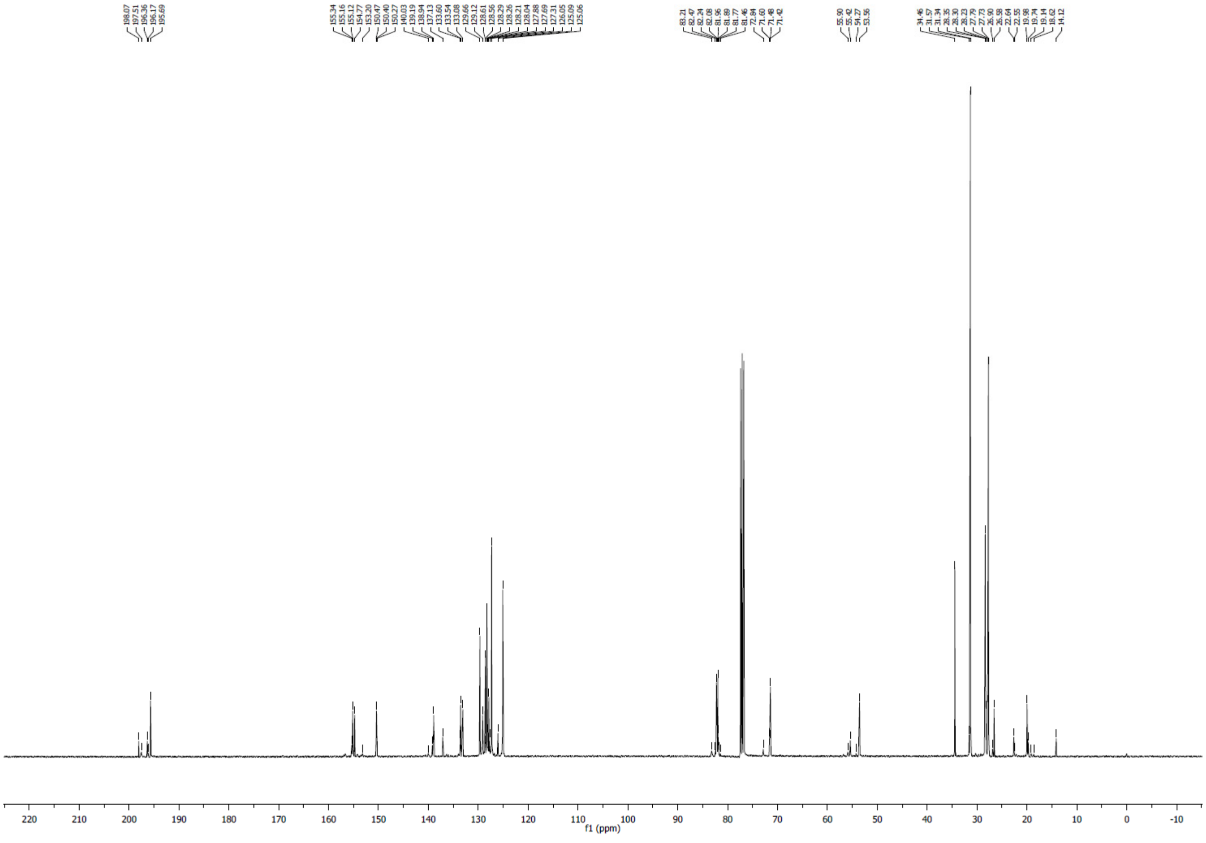

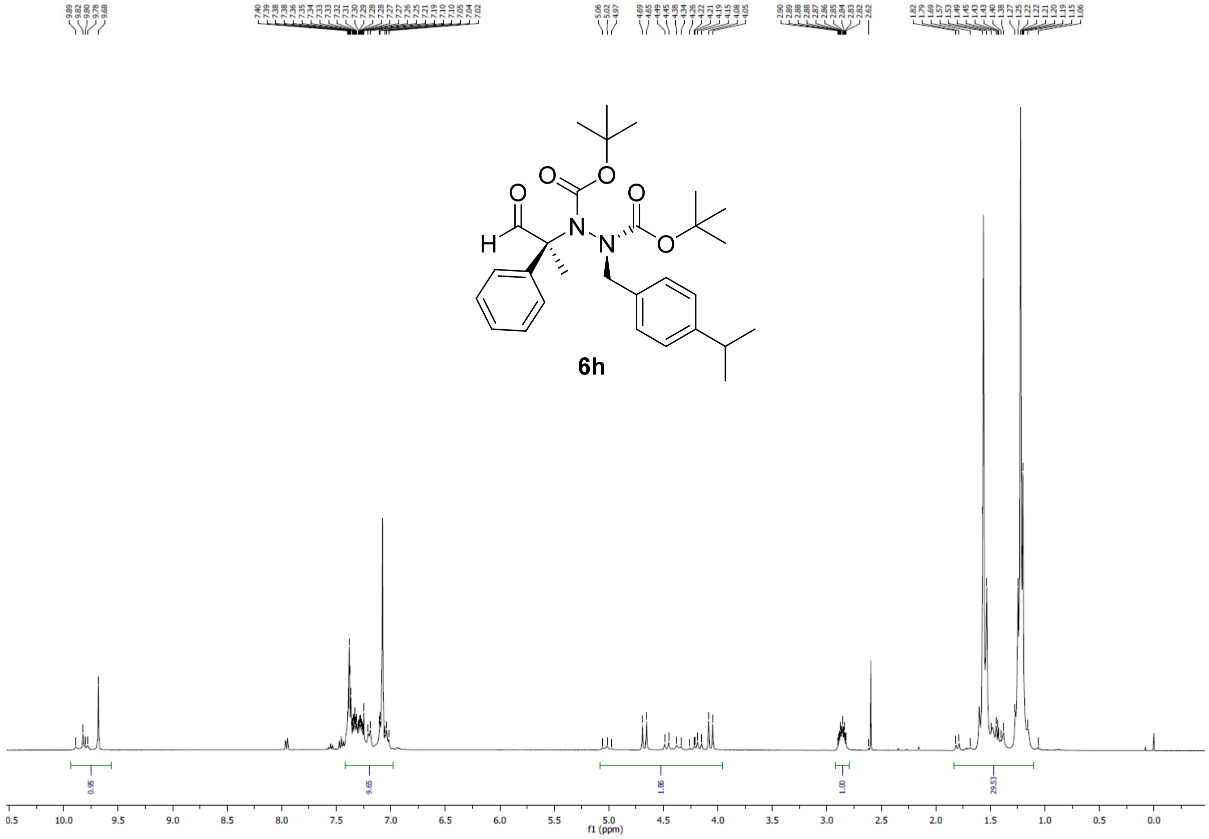
6h
f1 (ppm)

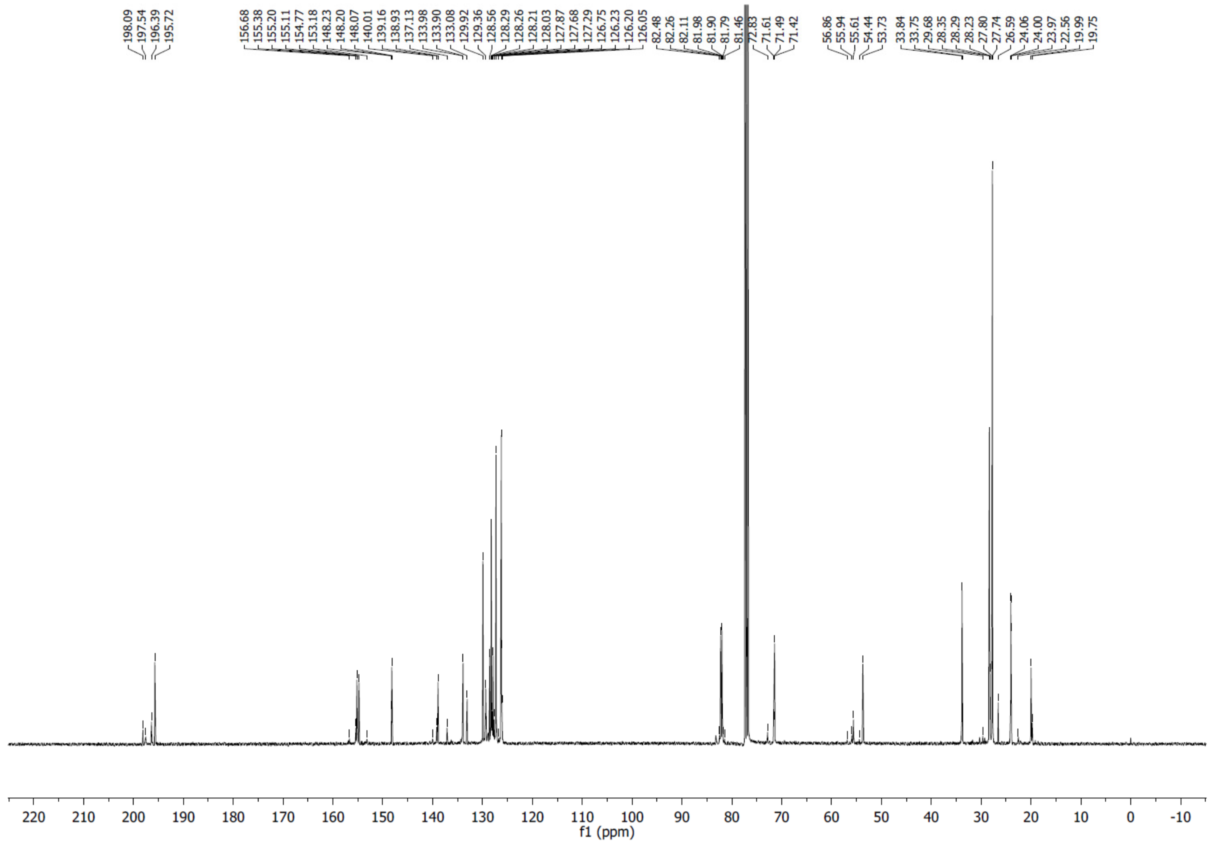
f1 (ppm)

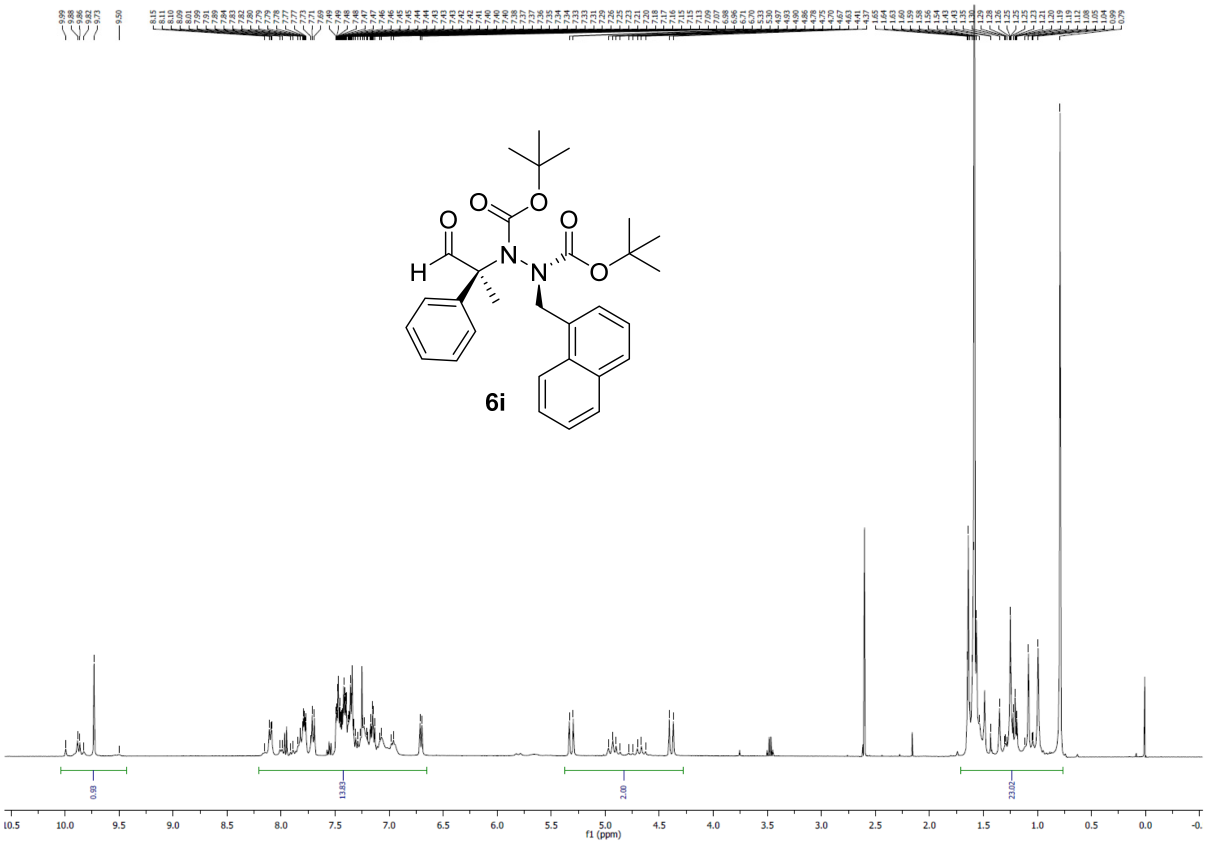
6i
f1 (ppm)

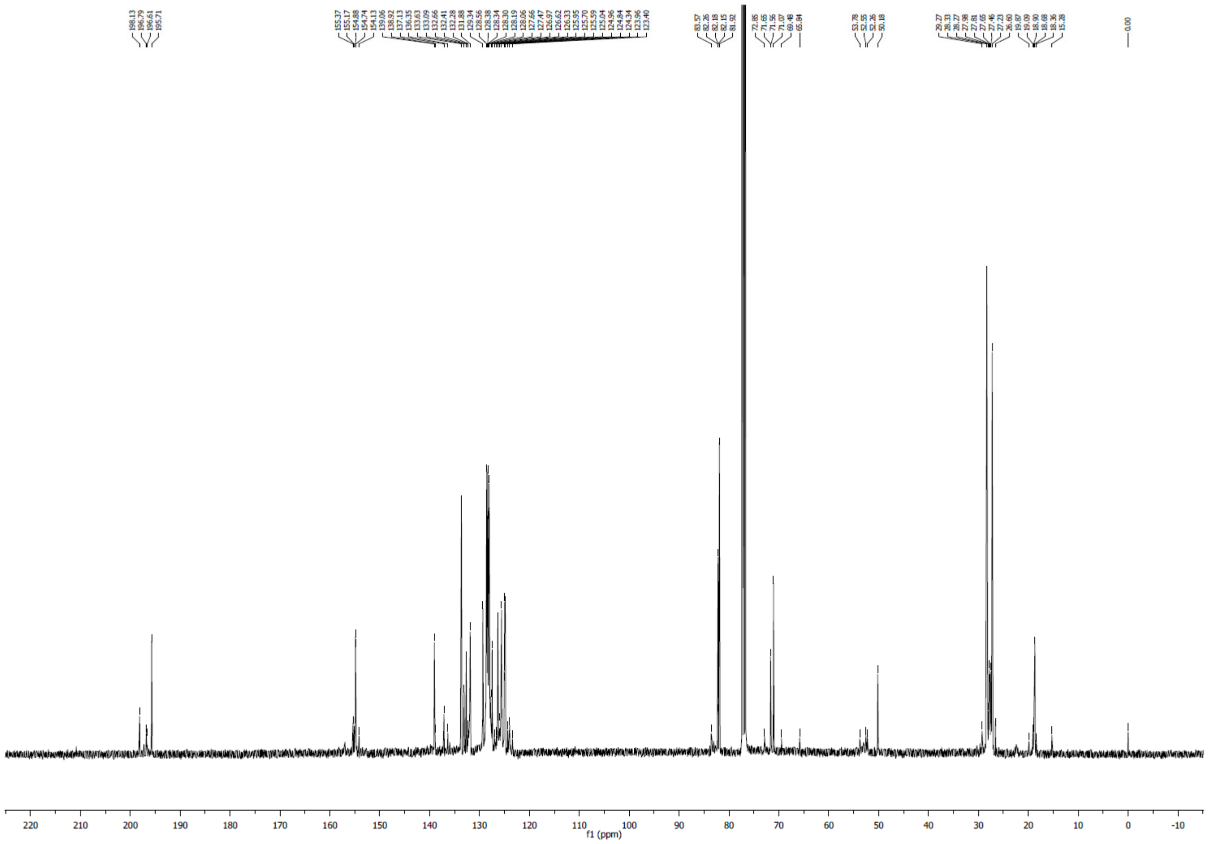
f1 (ppm)

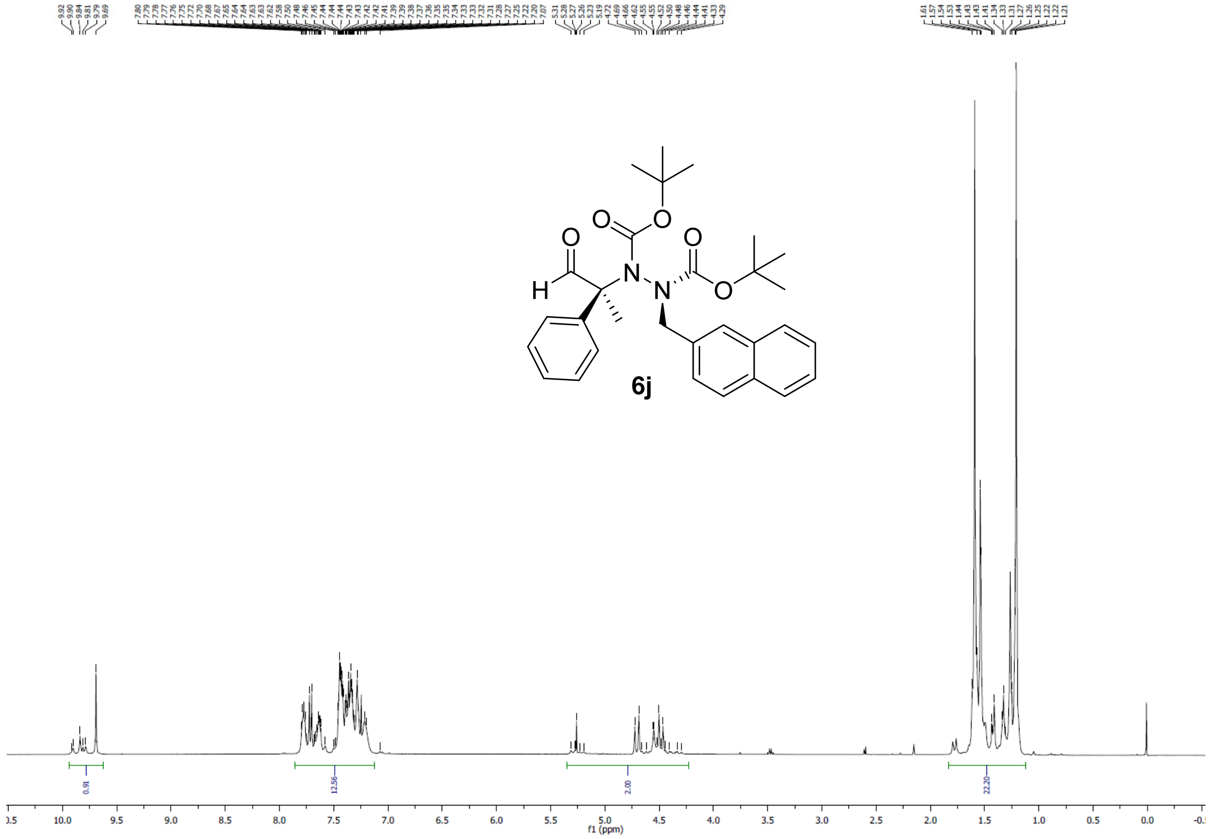
6j
f1 (ppm)

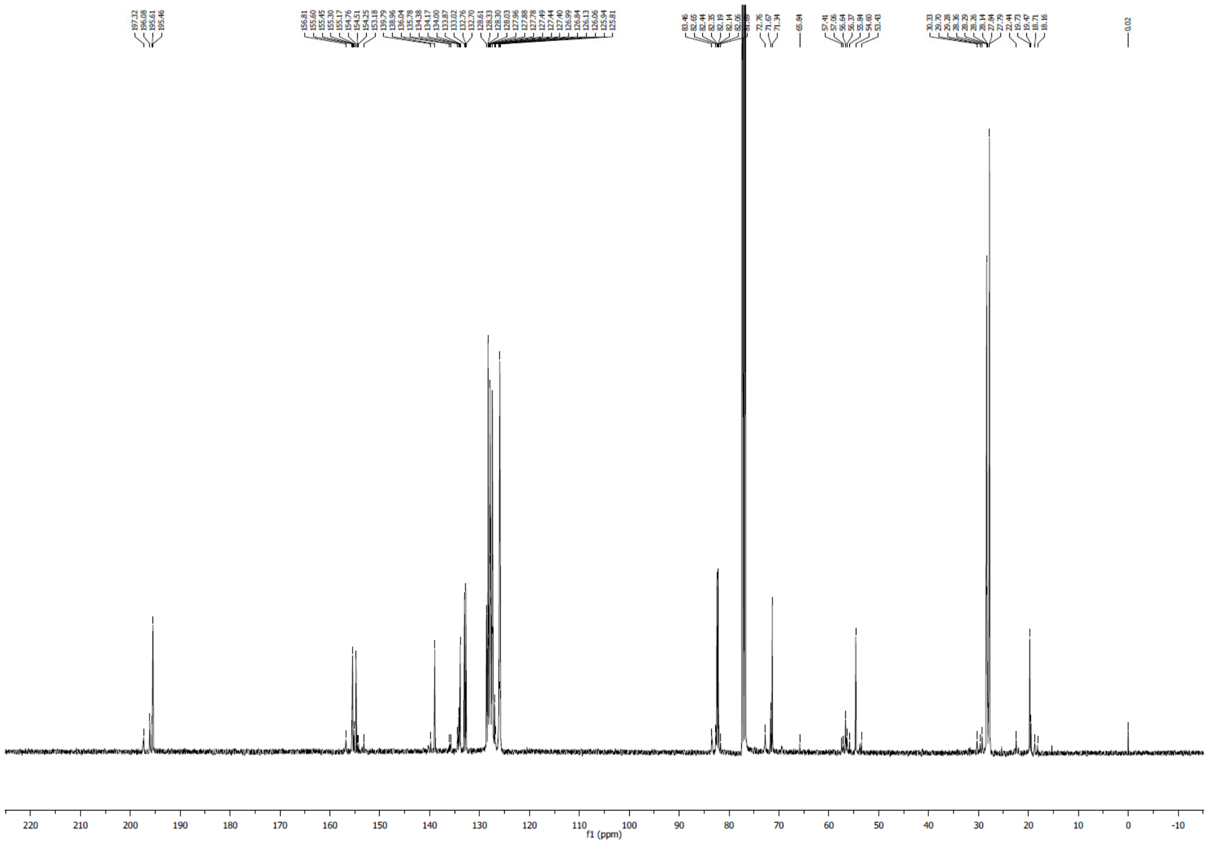
f1 (ppm)

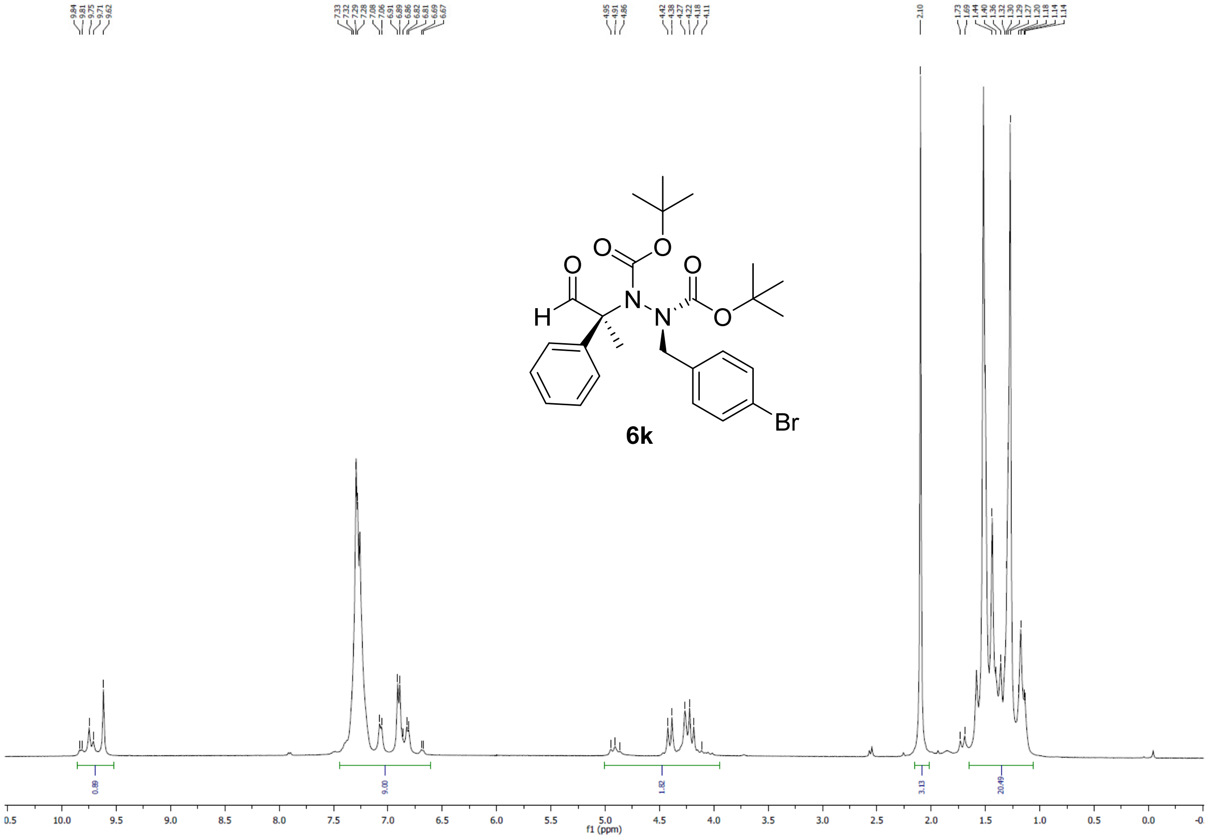
6k
Br
f1 (ppm)

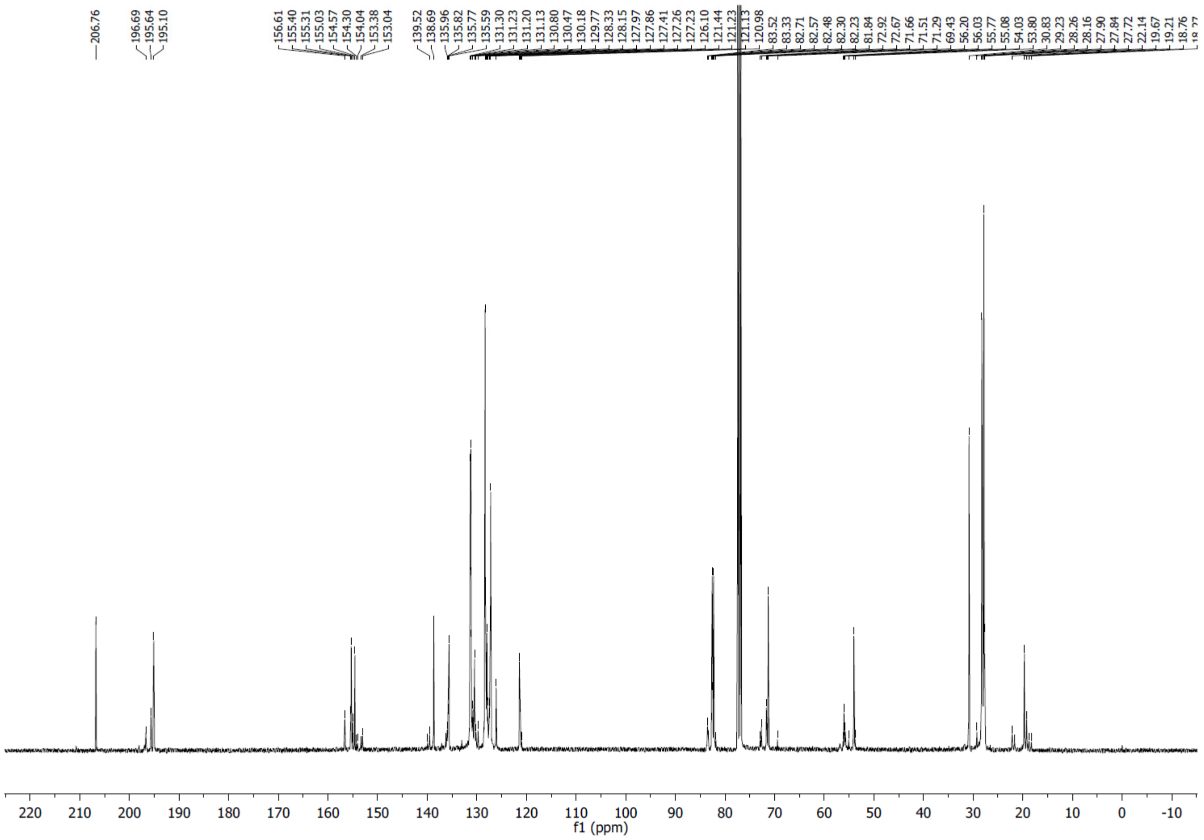
f1 (ppm)

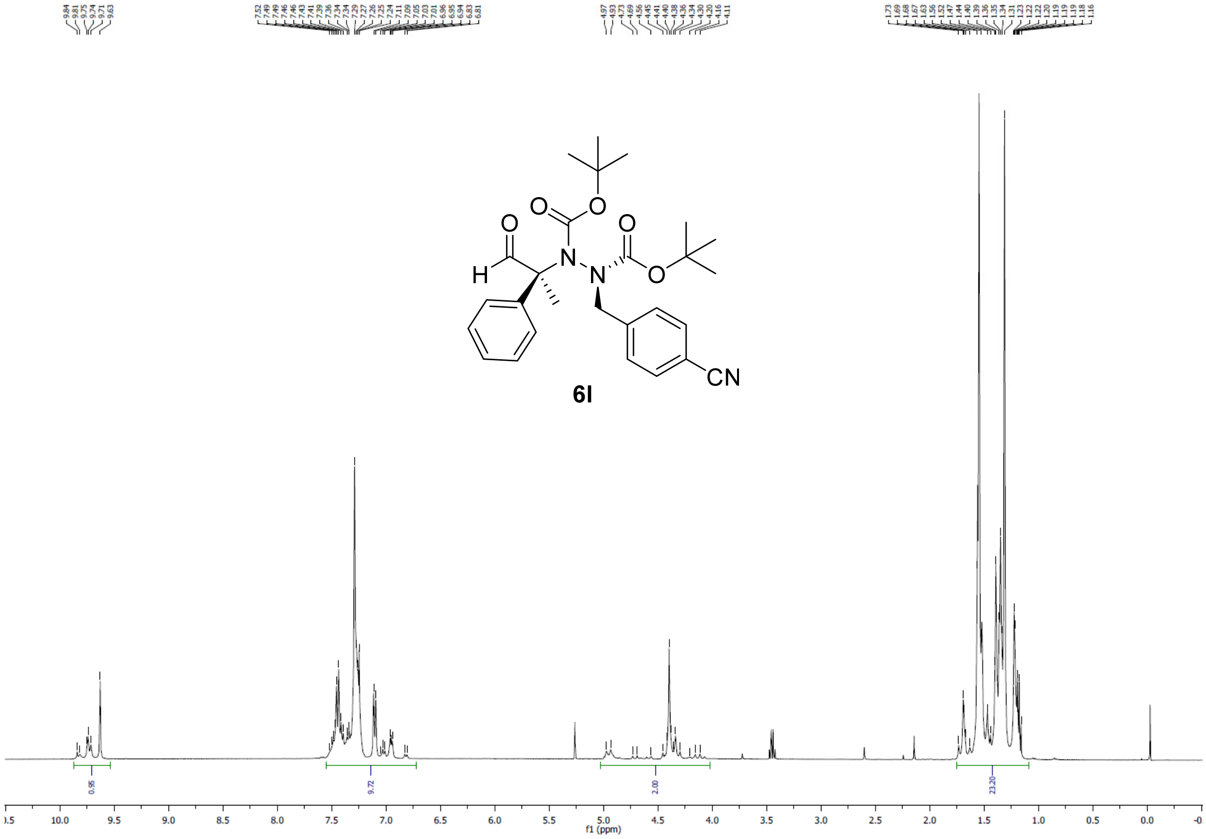
6l
f1 (ppm)

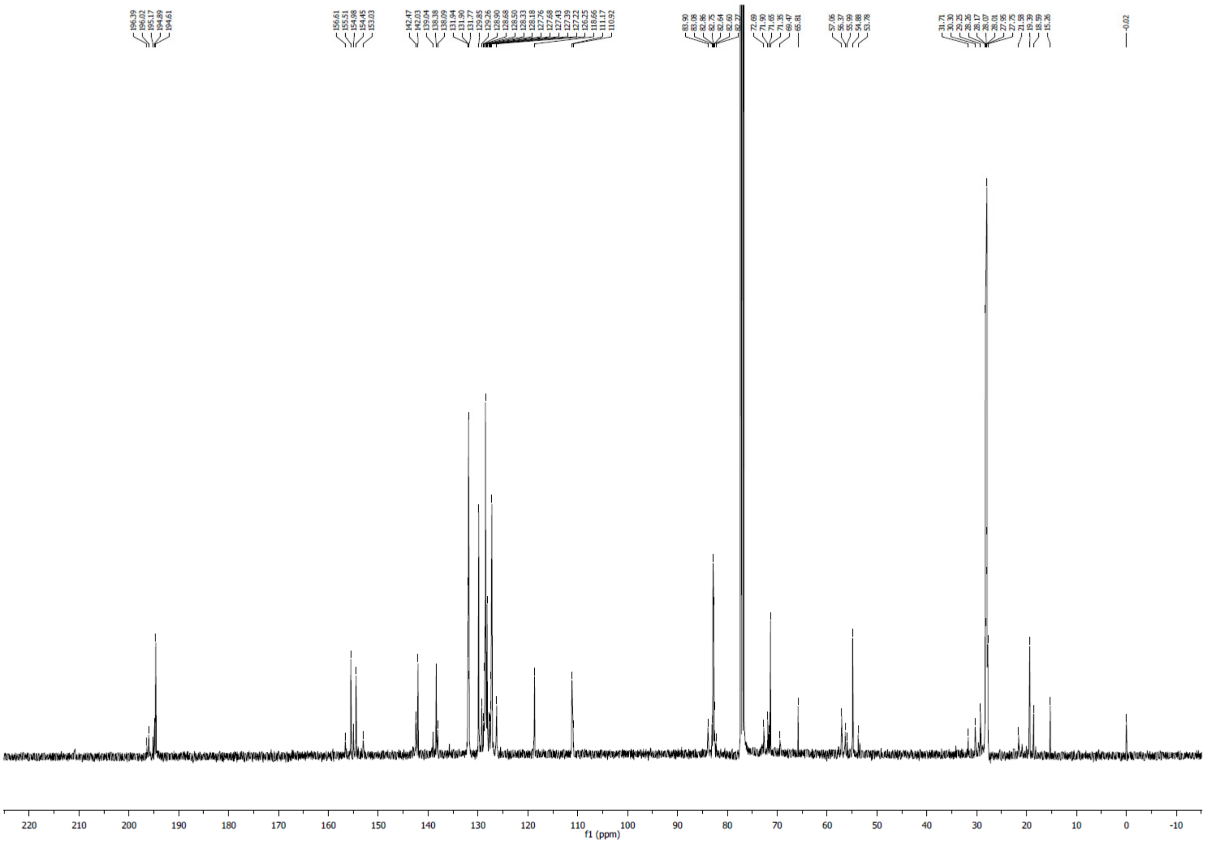
f1 (ppm)

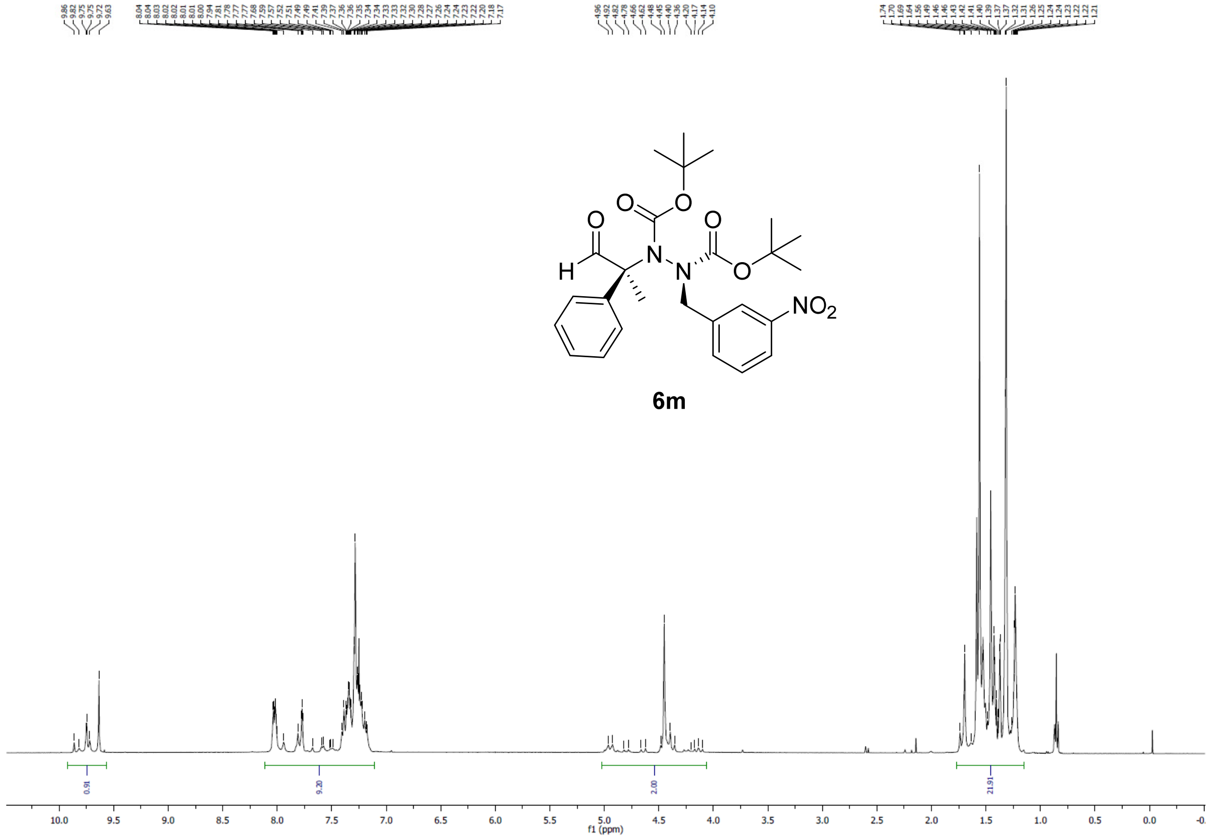

6m
f1 (ppm)

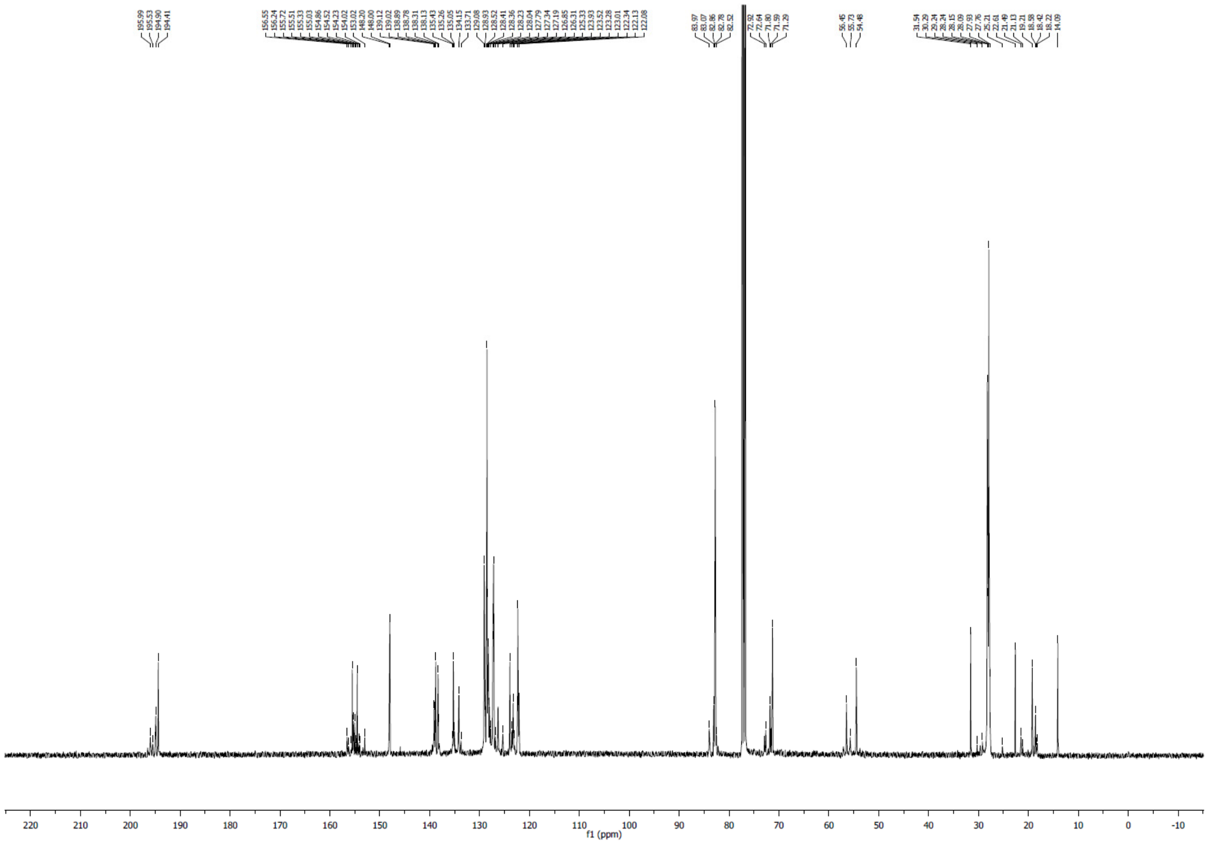

f1 (ppm)

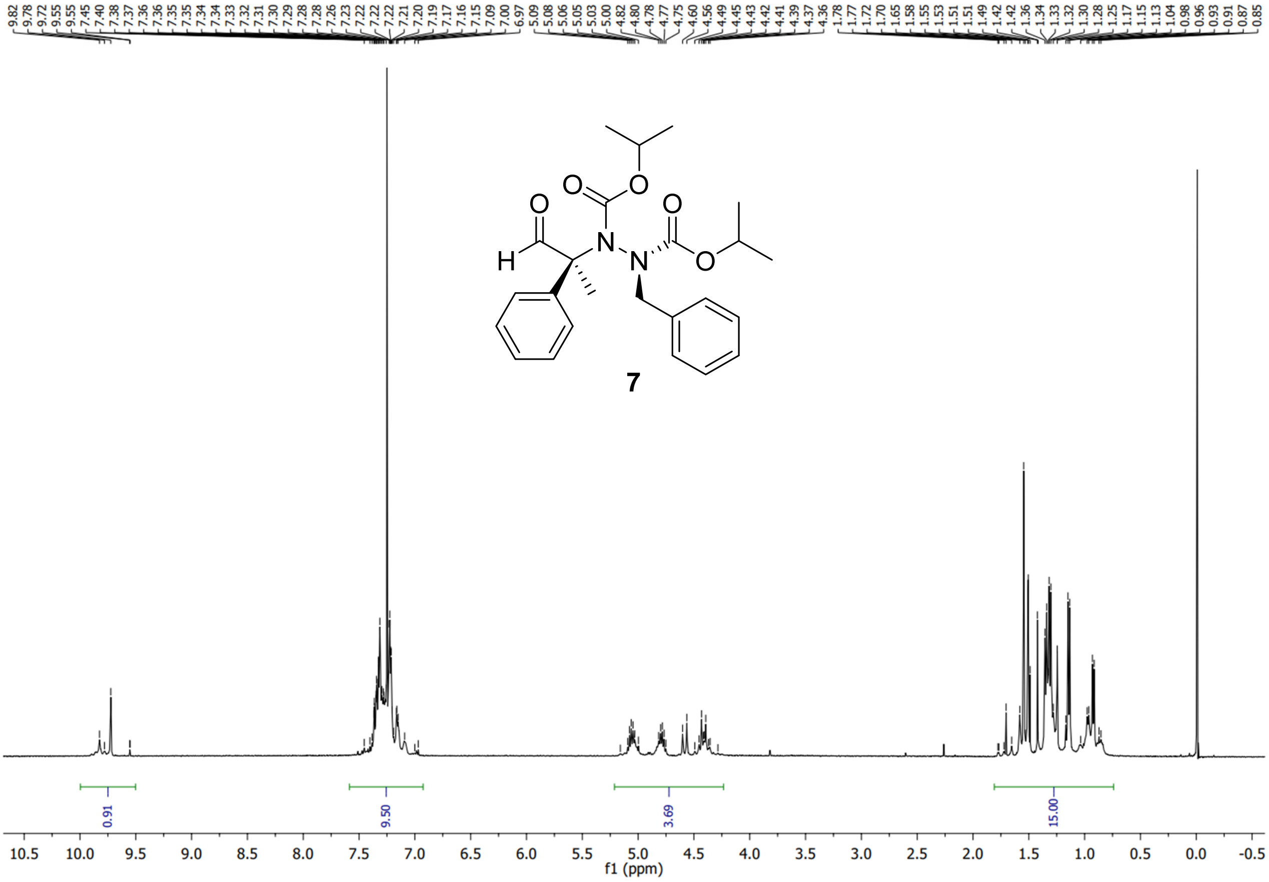
7
0.91
9.50
3.69
15.00
f1 (ppm)

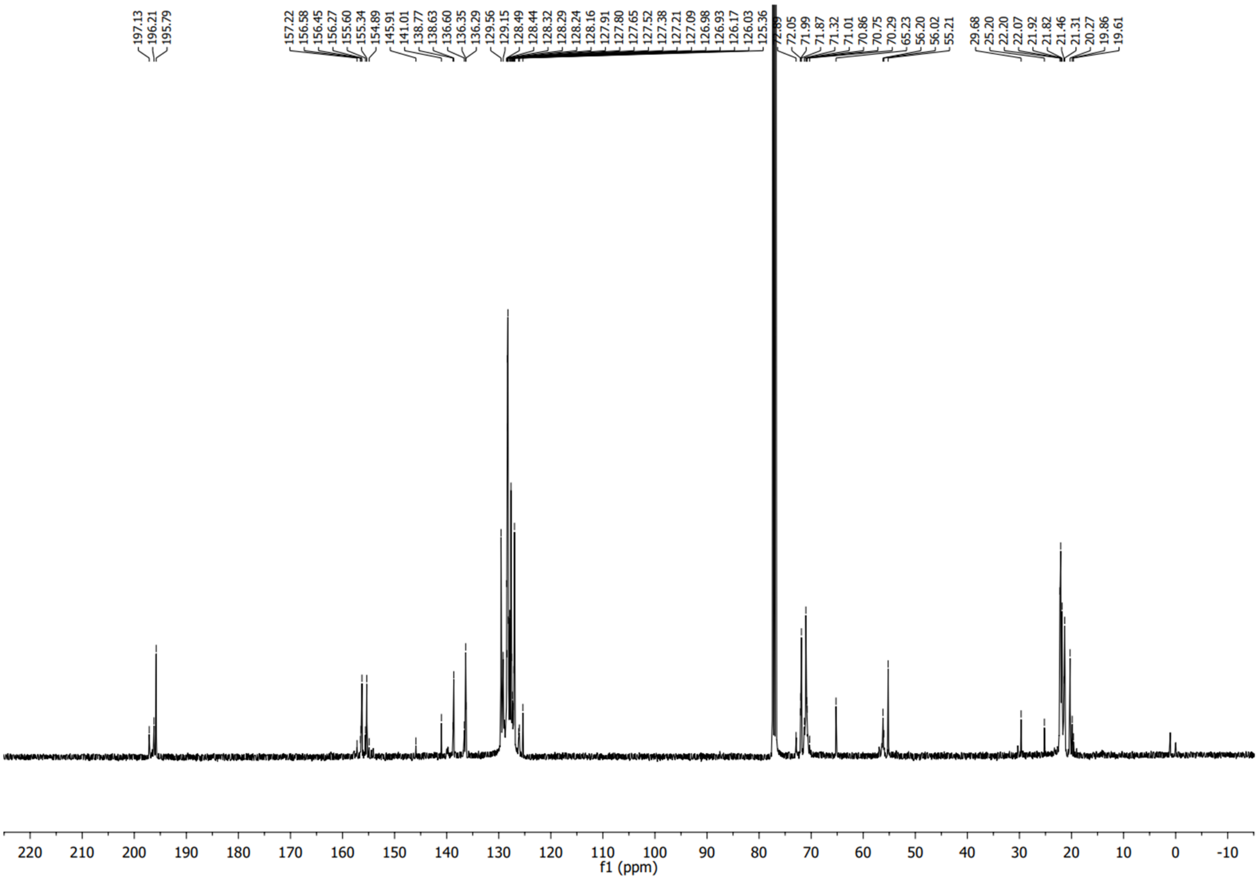
f1 (ppm)

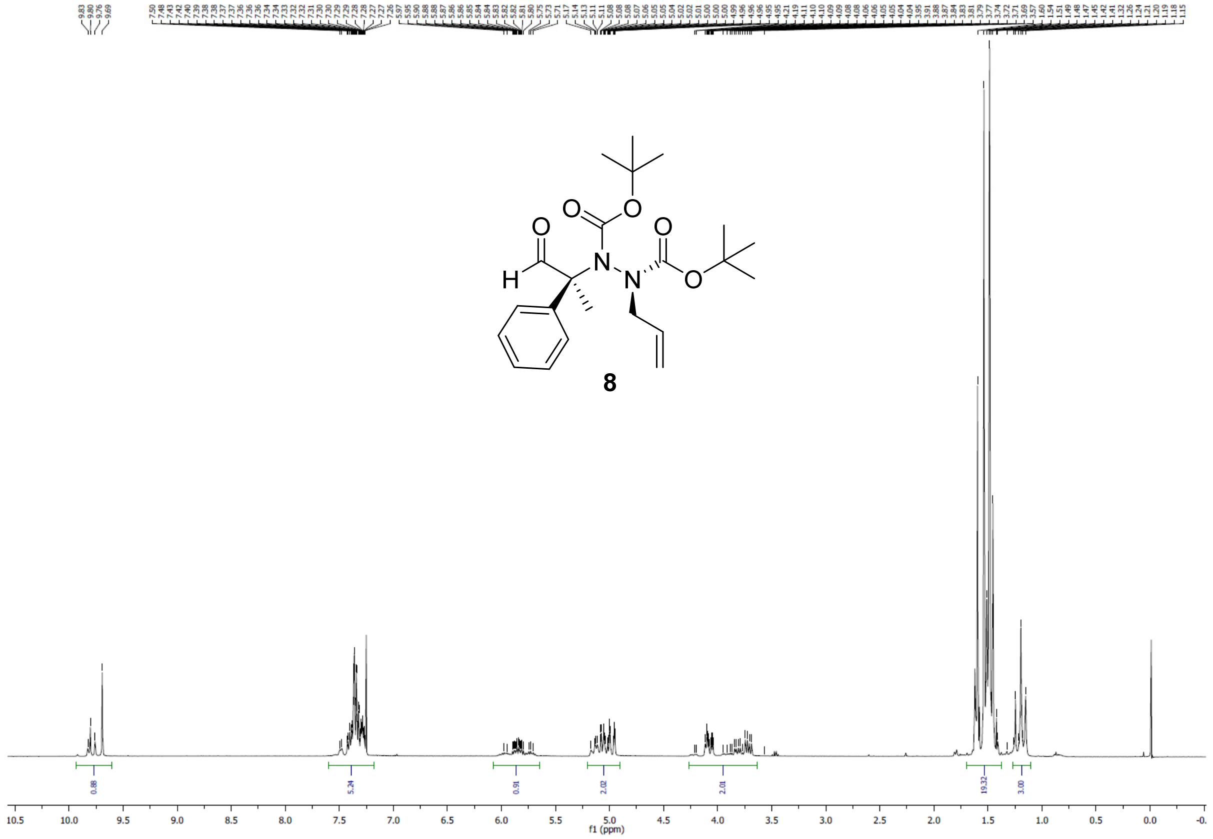
8
f1 (ppm)

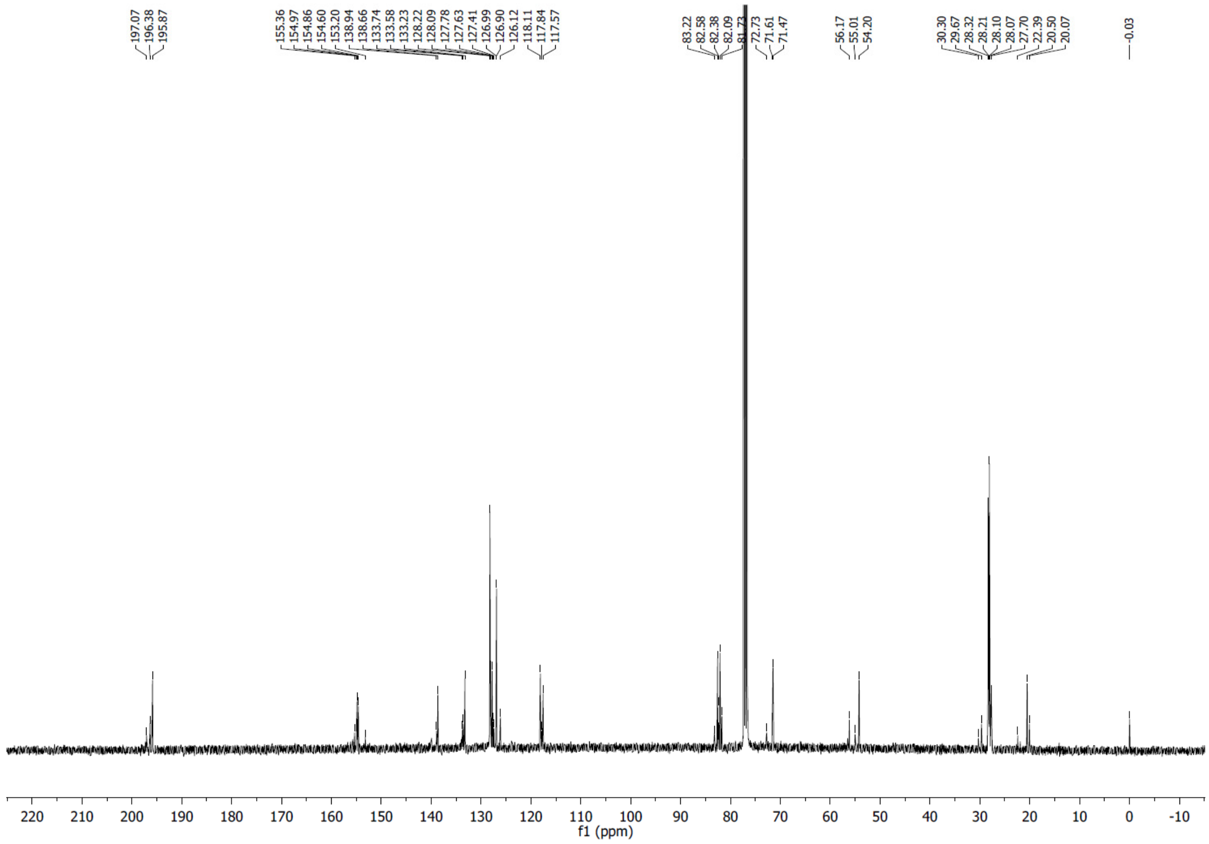
197.07
196.38
195.87
155.36
154.97
154.86
154.60
153.20
138.94
138.66
133.74
133.58
133.23
128.22
128.09
127.78
127.63
127.41
126.99
126.90
126.12
118.11
117.84
117.57
83.22
82.58
82.38
82.09
72.73
71.61
71.47
56.17
55.01
54.20
30.30
29.67
28.32
28.21
28.10
28.07
27.70
22.39
20.50
20.07
-0.03
f1 (ppm)

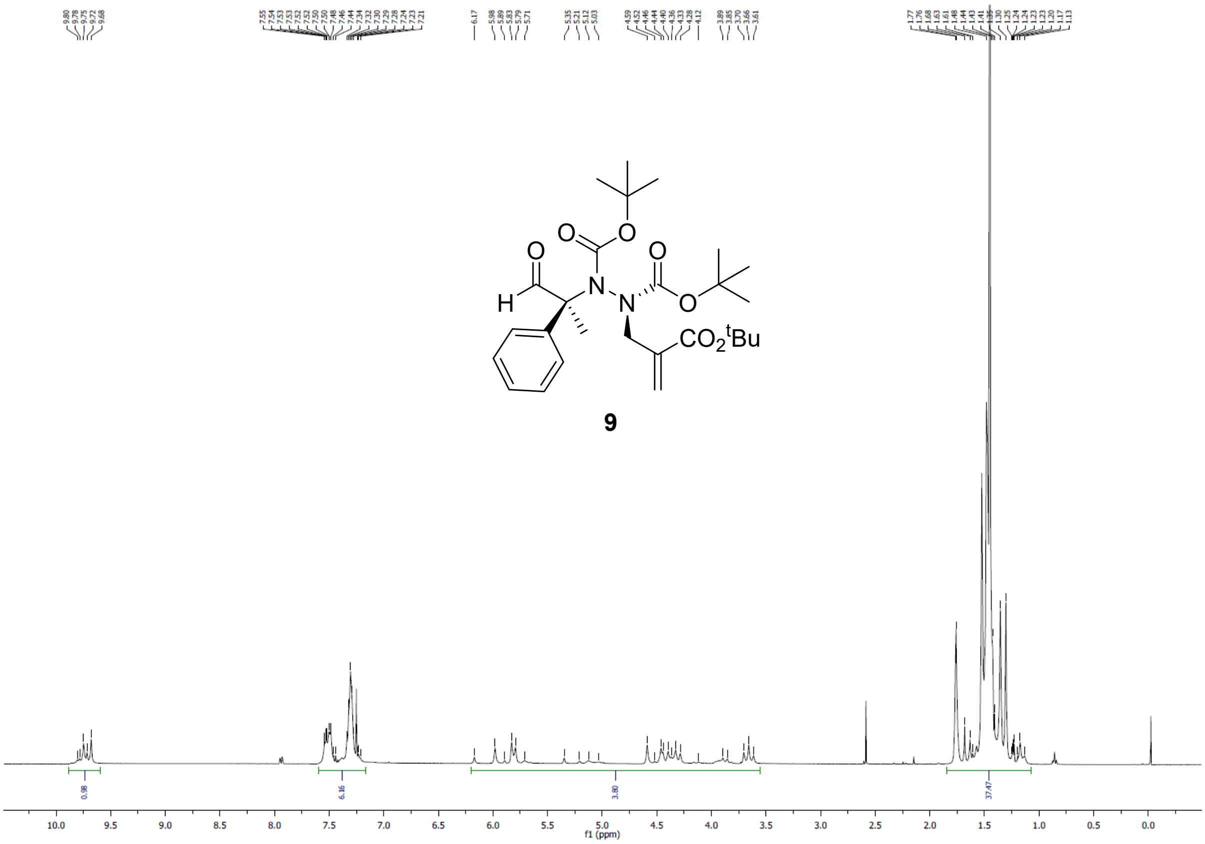

9

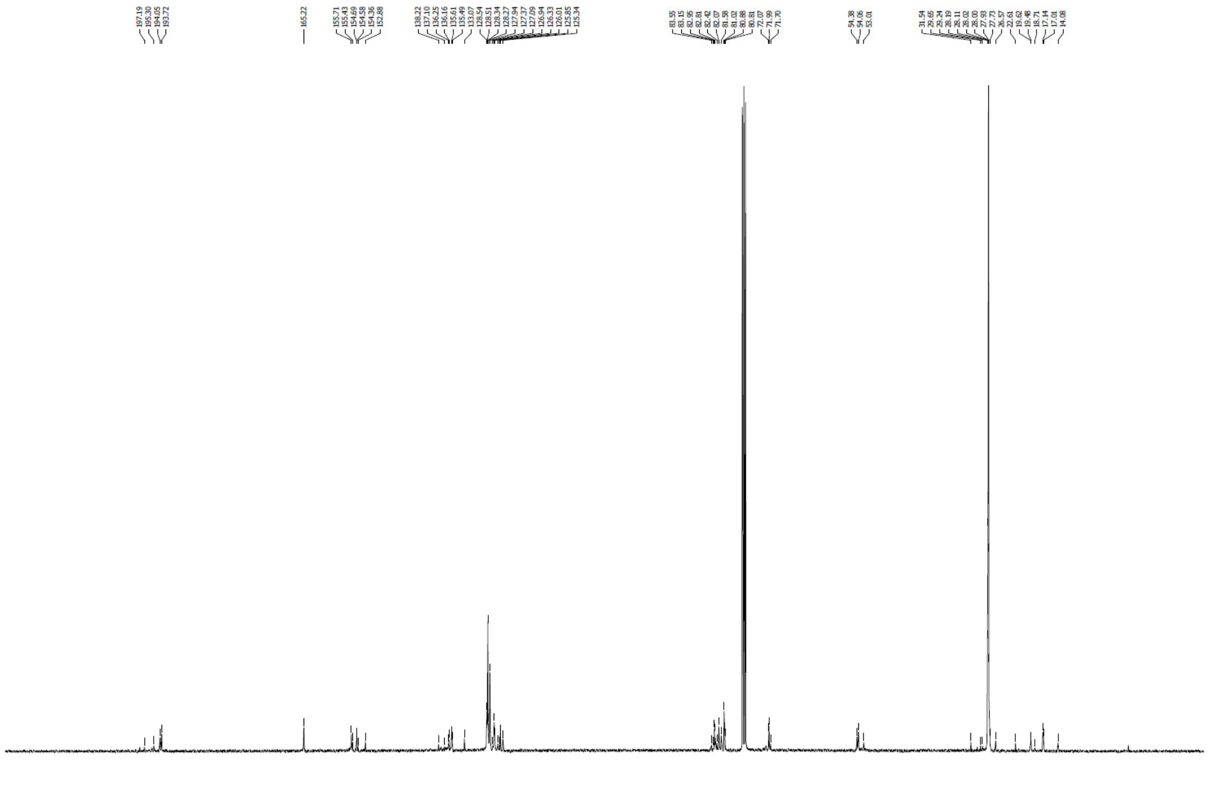

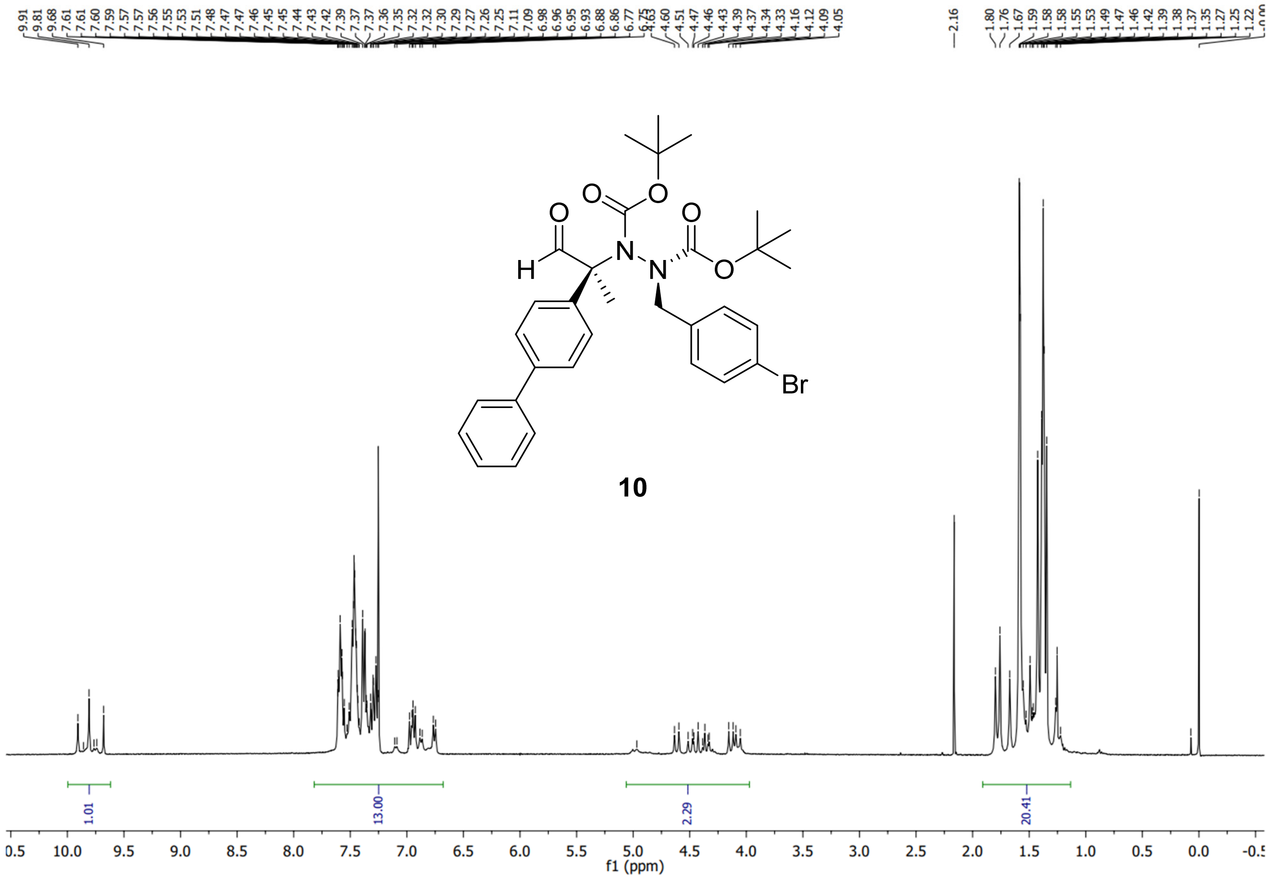

10

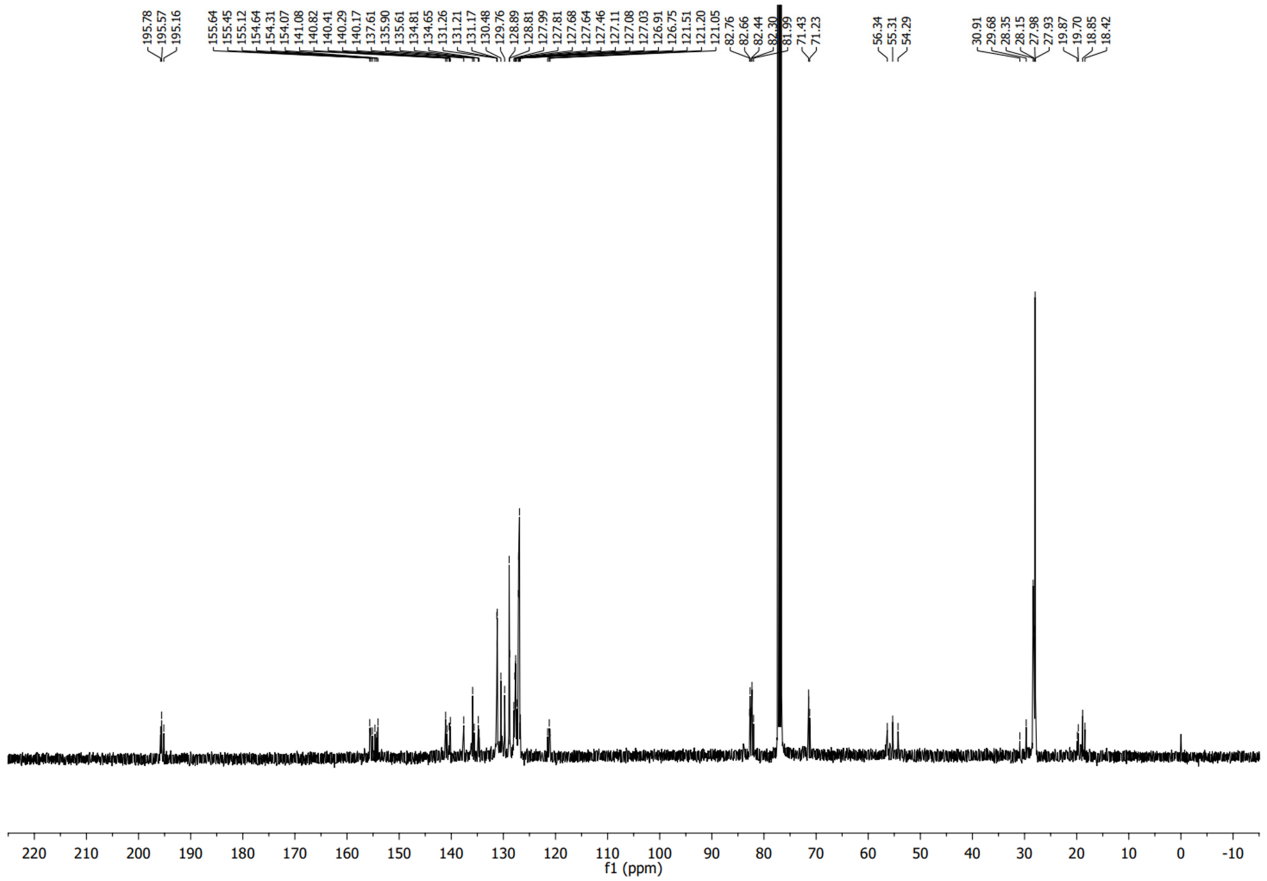

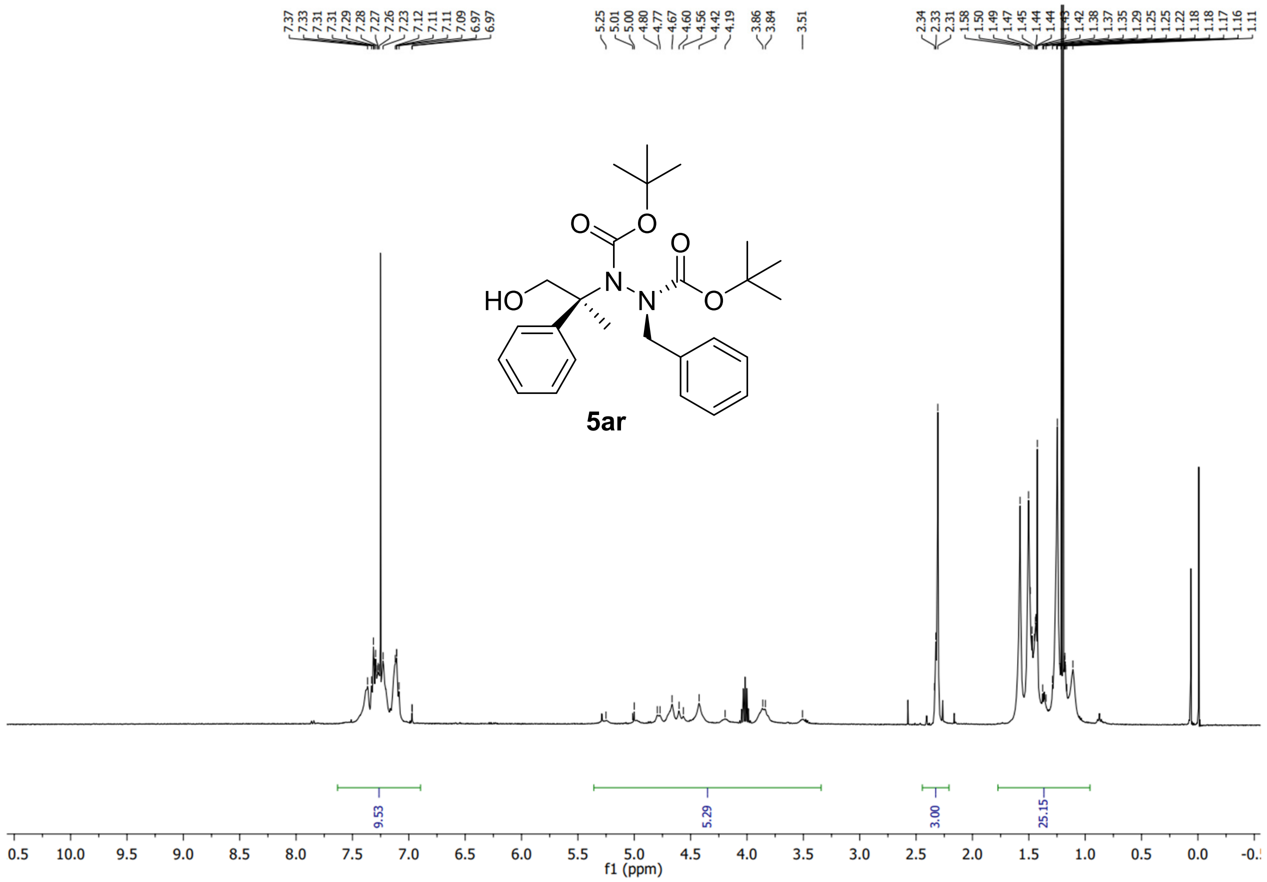
7.37
7.33
7.31
7.29
7.28
7.27
7.26
7.23
7.12
7.11
7.11
7.09
6.97
6.97
5.25
5.01
5.00
4.80
4.77
4.67
4.60
4.56
4.42
4.19
3.86
3.84
3.51
2.34
2.33
2.31
1.58
1.50
1.49
1.47
1.45
1.44
1.44
1.42
1.38
1.37
1.35
1.29
1.25
1.25
1.22
1.18
1.18
1.17
1.16
1.11
HO
O
N
5ar
9.53
5.29
3.00
25.15
f1 (ppm)

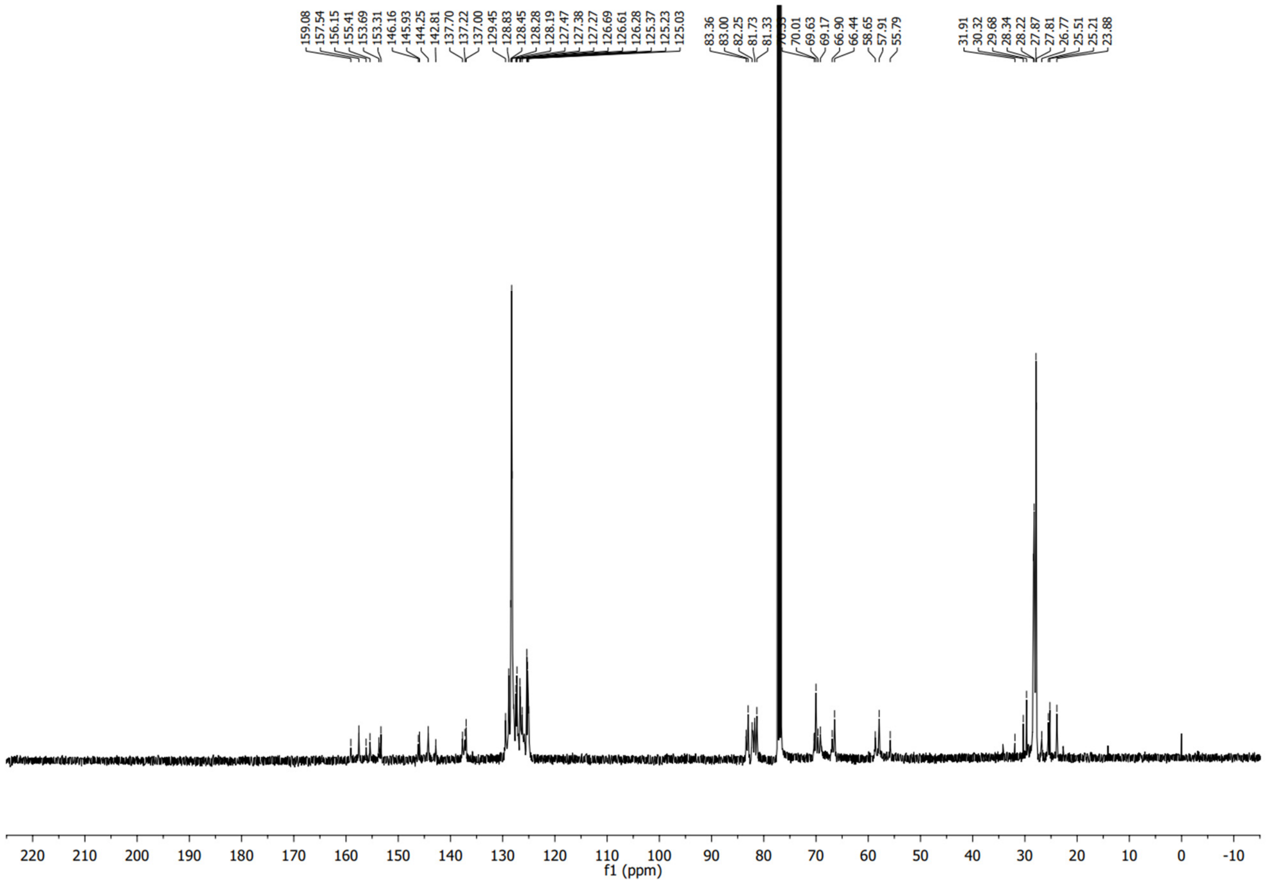
159.08
157.54
156.15
155.41
153.69
153.31
146.16
145.93
144.25
142.81
137.70
137.22
137.00
129.45
128.83
128.45
128.28
128.19
127.47
127.38
127.27
126.69
126.61
126.28
125.37
125.23
125.03
83.36
83.00
82.25
81.73
81.33
70.01
69.63
69.17
66.90
66.44
58.65
57.91
55.79
31.91
30.32
29.68
28.34
28.22
27.87
27.81
26.77
25.51
25.21
23.88
f1 (ppm)

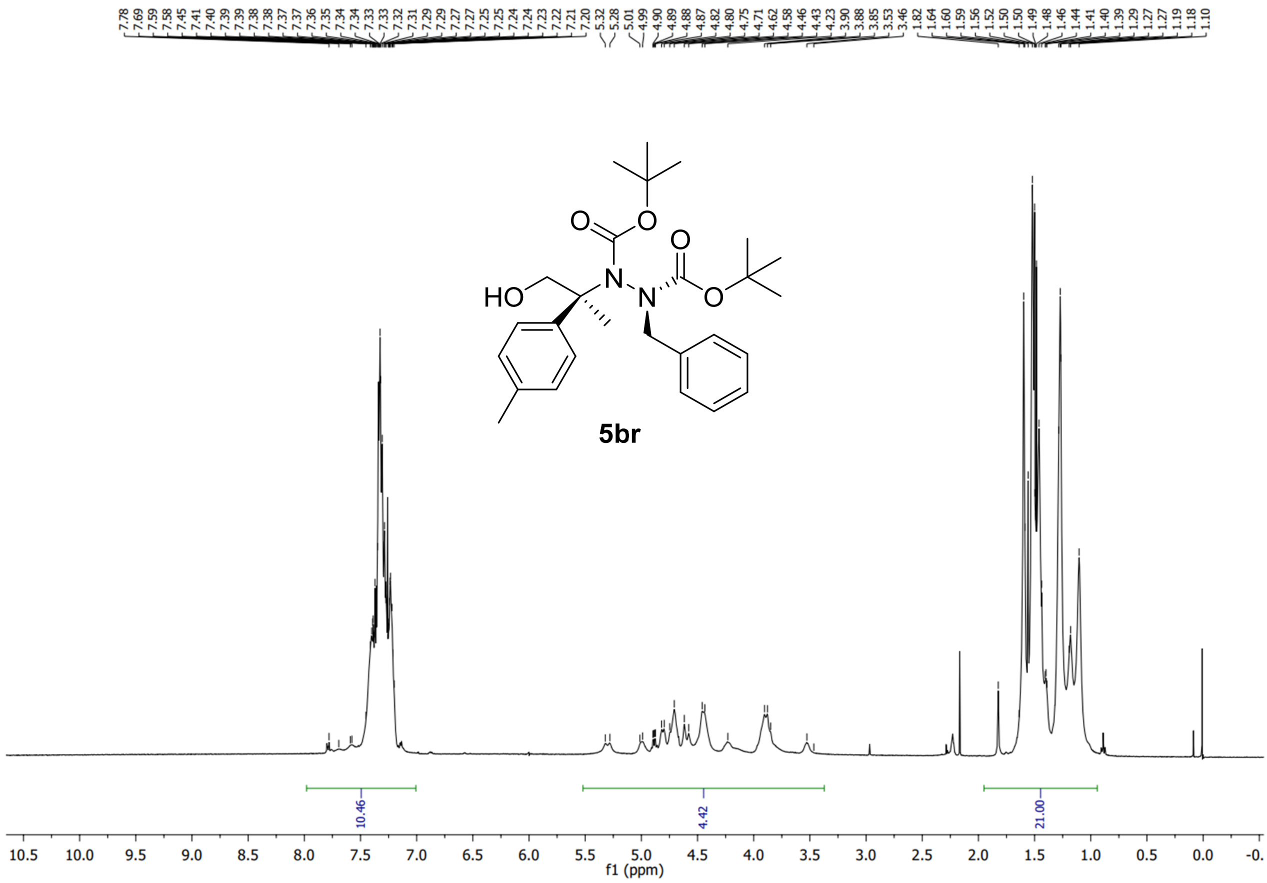
5br
f1 (ppm)

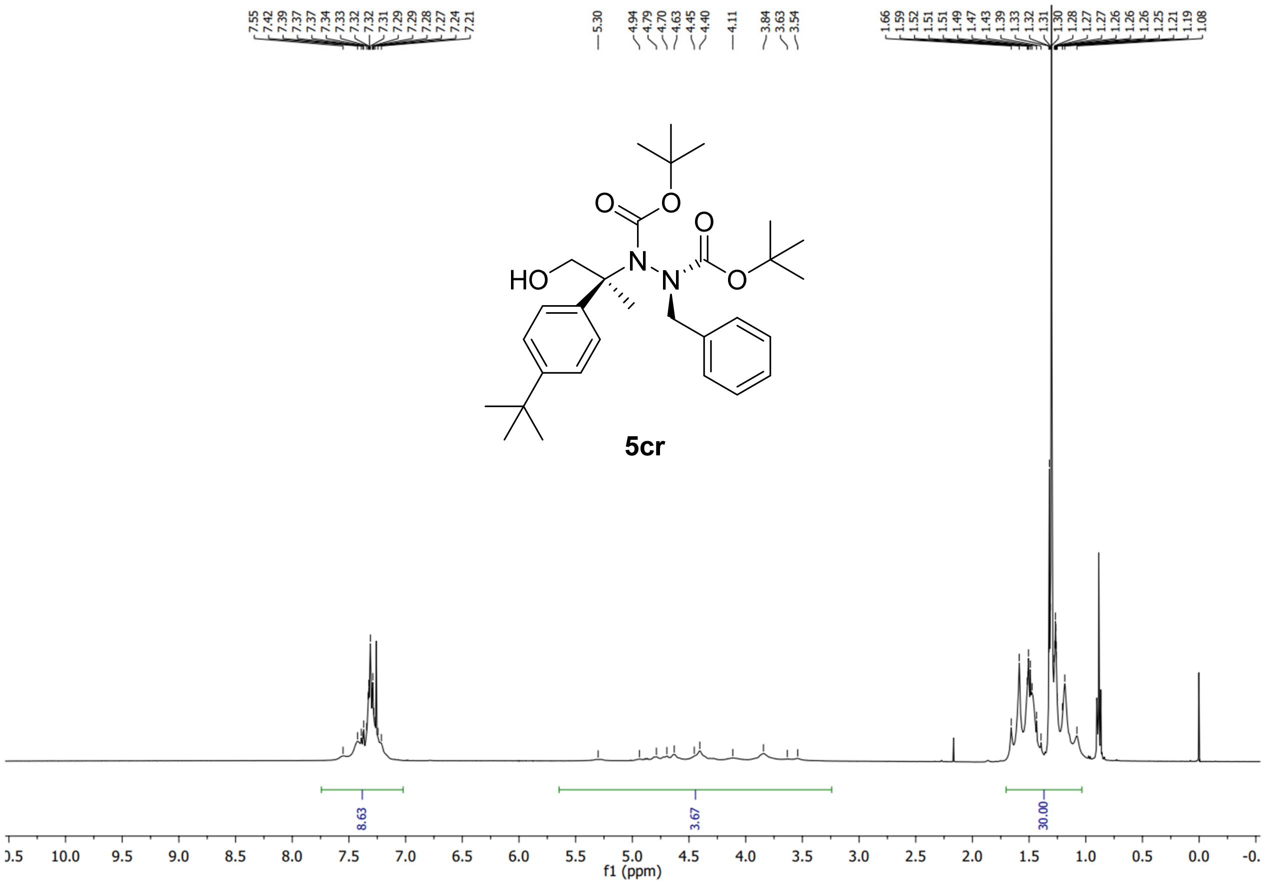
5cr
f1 (ppm)

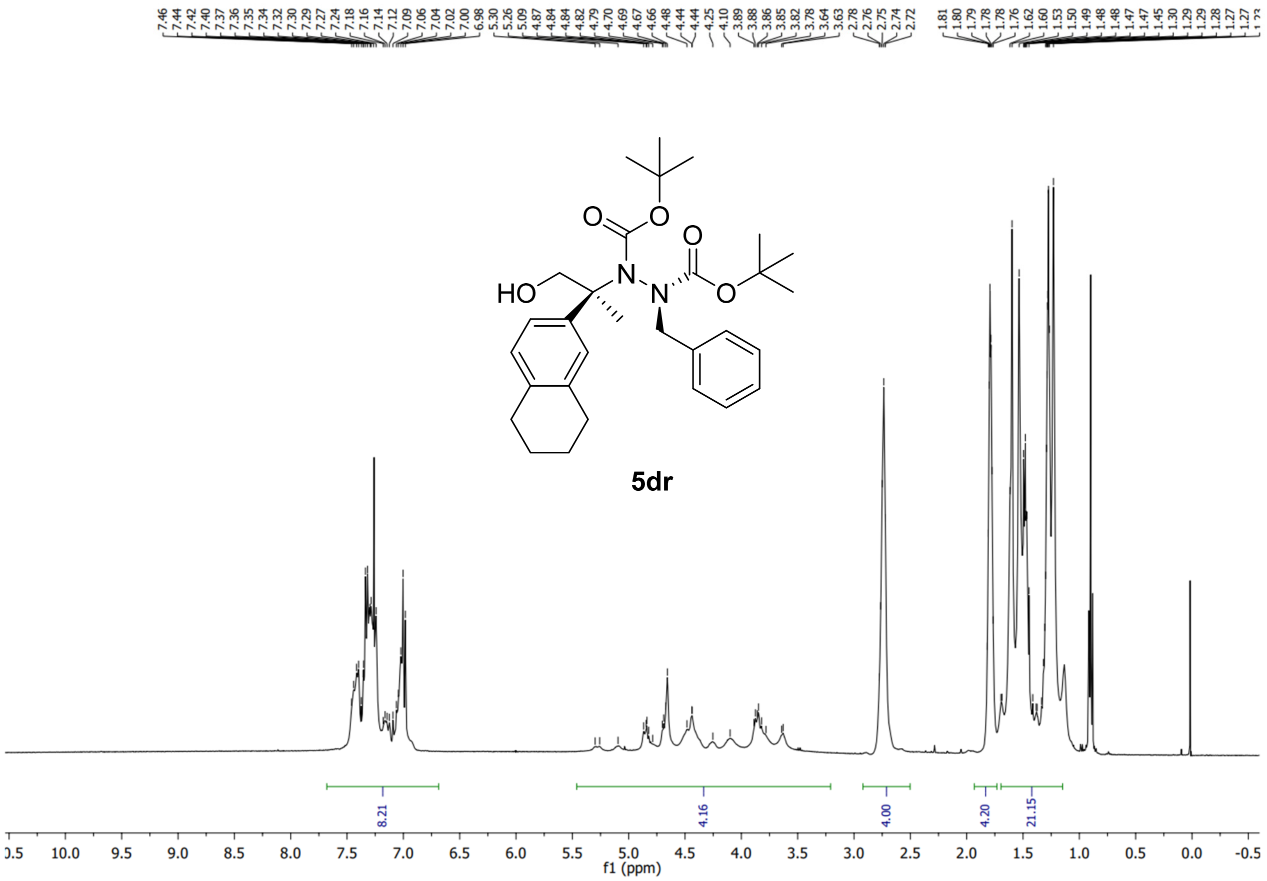

5dr

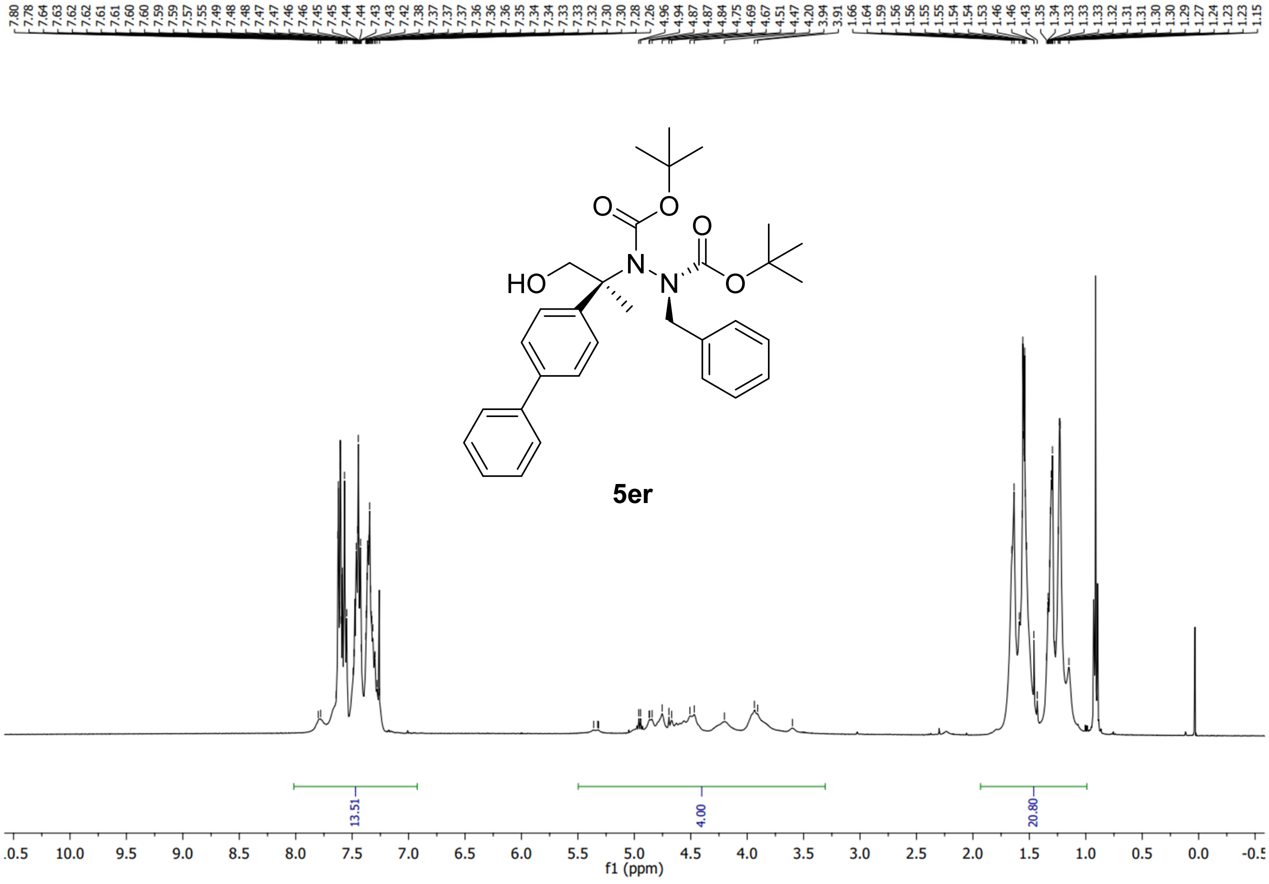

5er

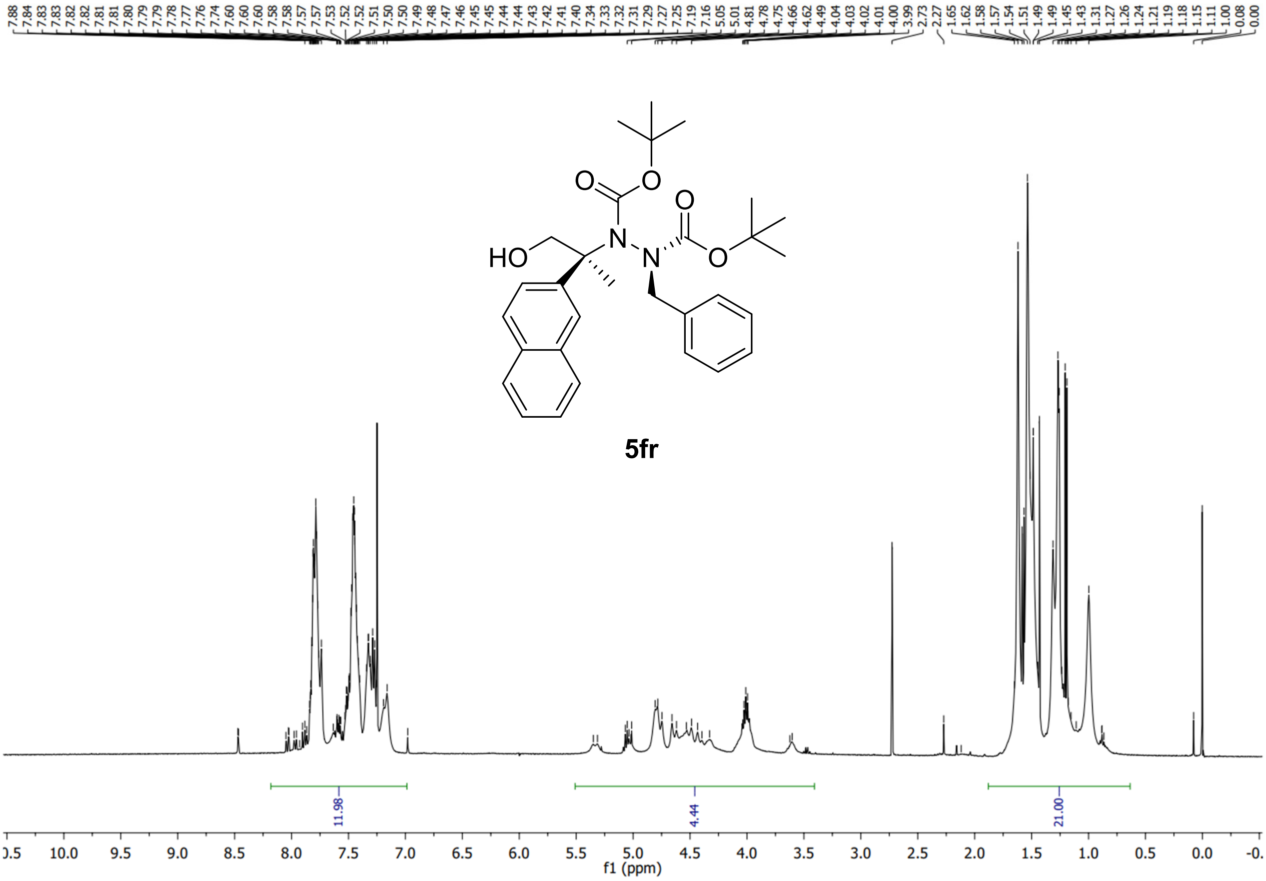
5fr
f1 (ppm)

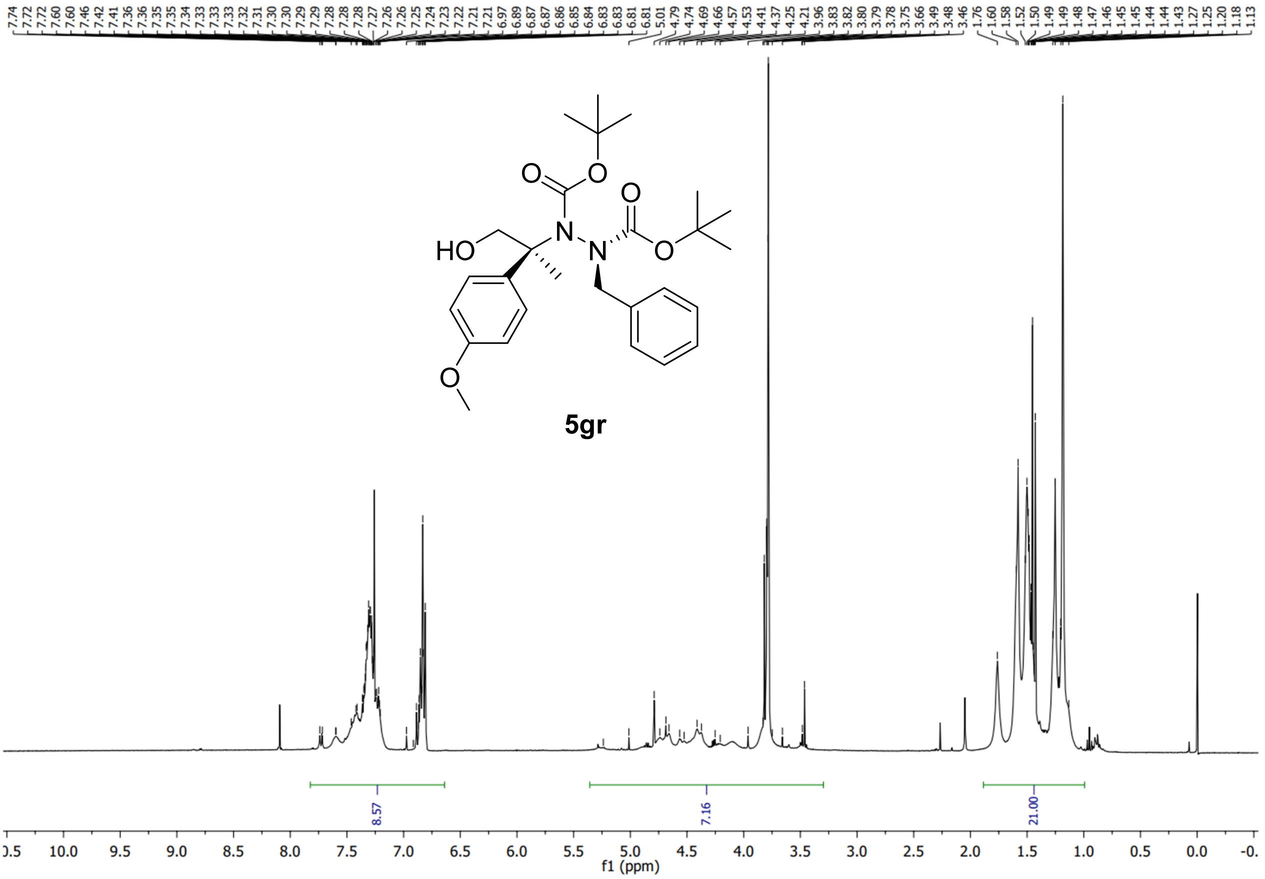
5gr
f1 (ppm)

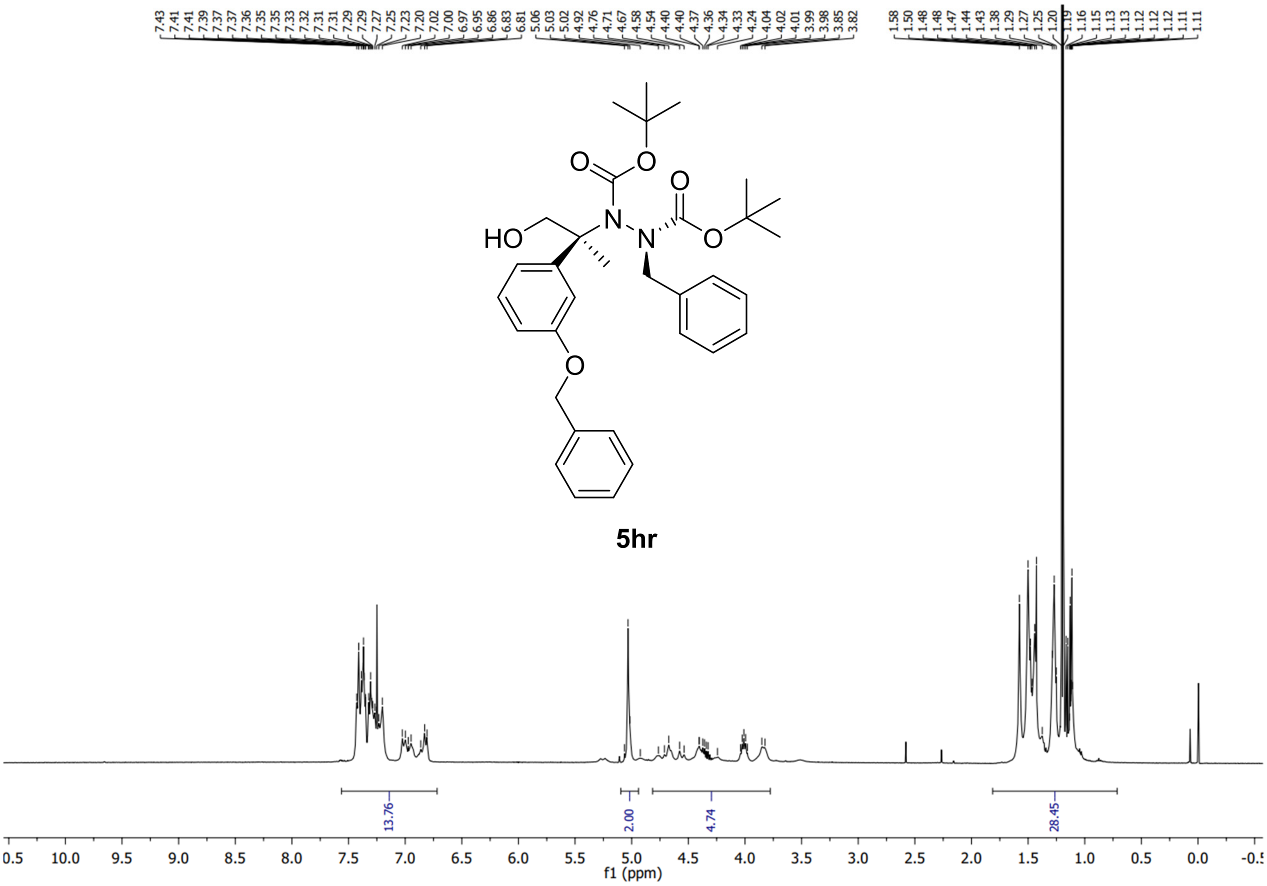
5hr
f1 (ppm)

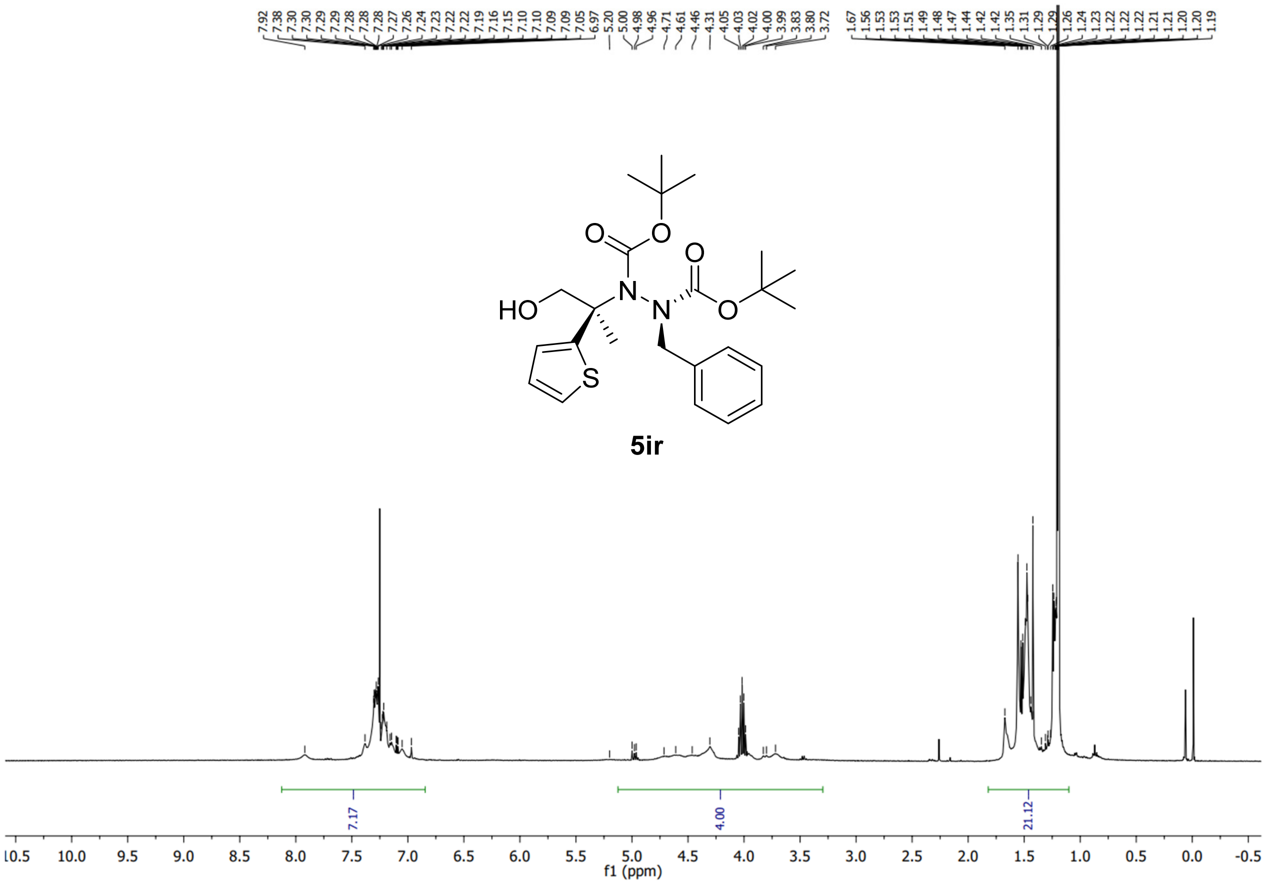
5ir
f1 (ppm)

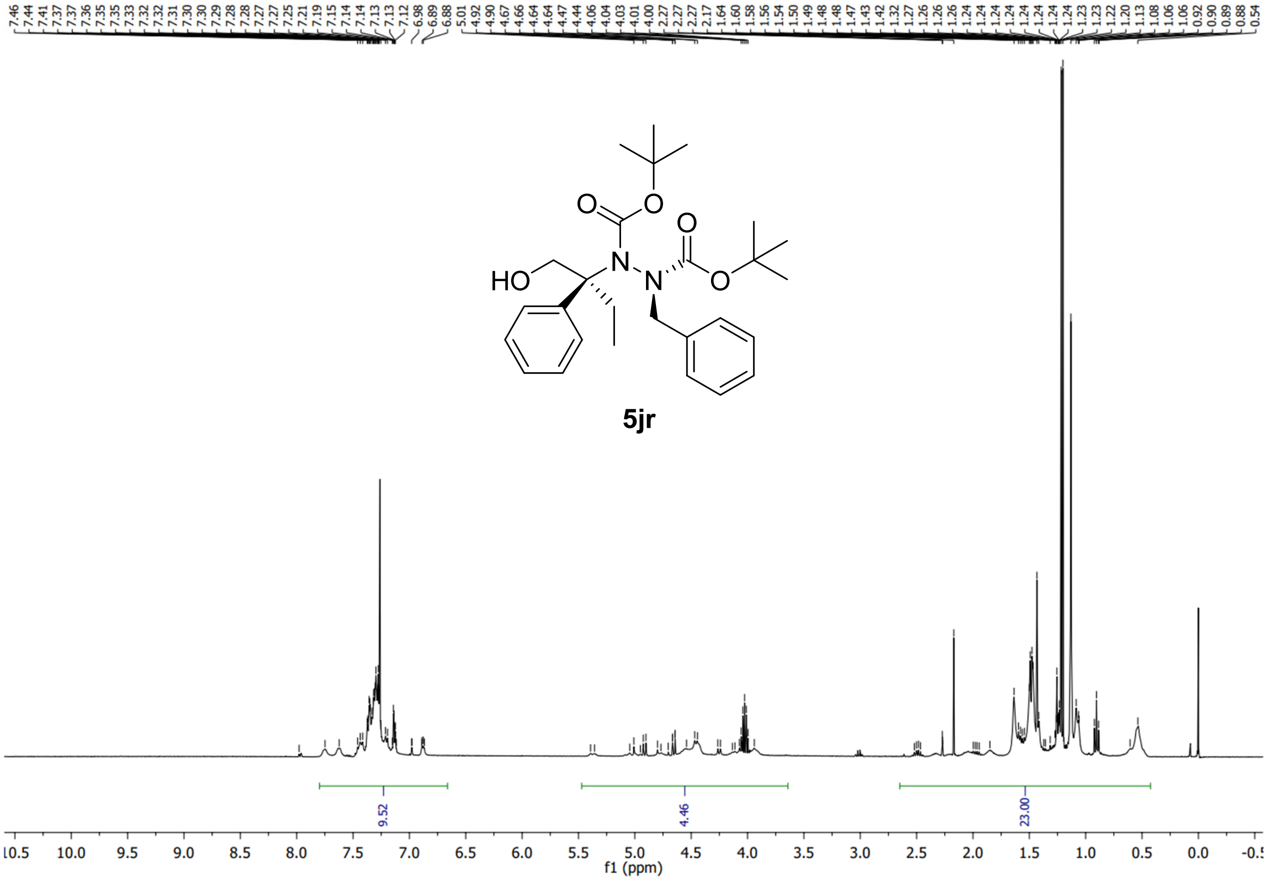
HO
O
N
5jr
f1 (ppm)

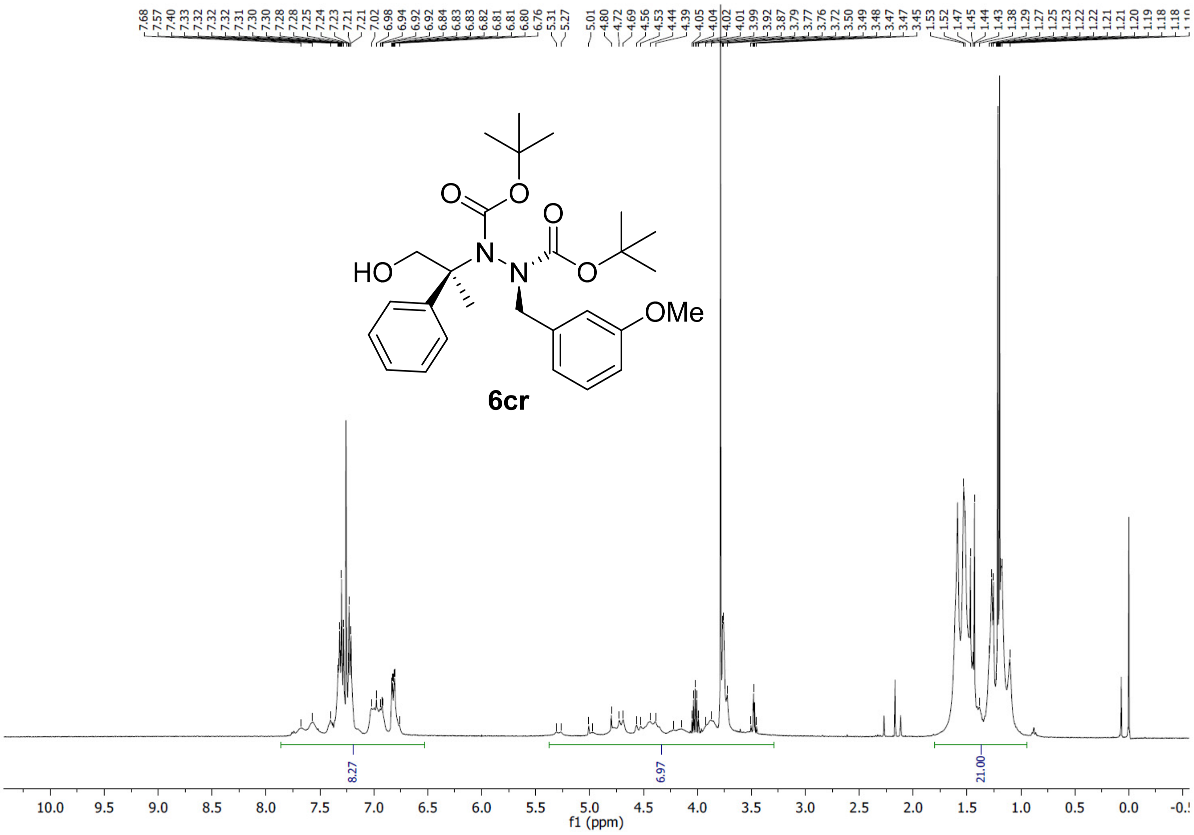
HO
O
N
OMe
6cr
f1 (ppm)

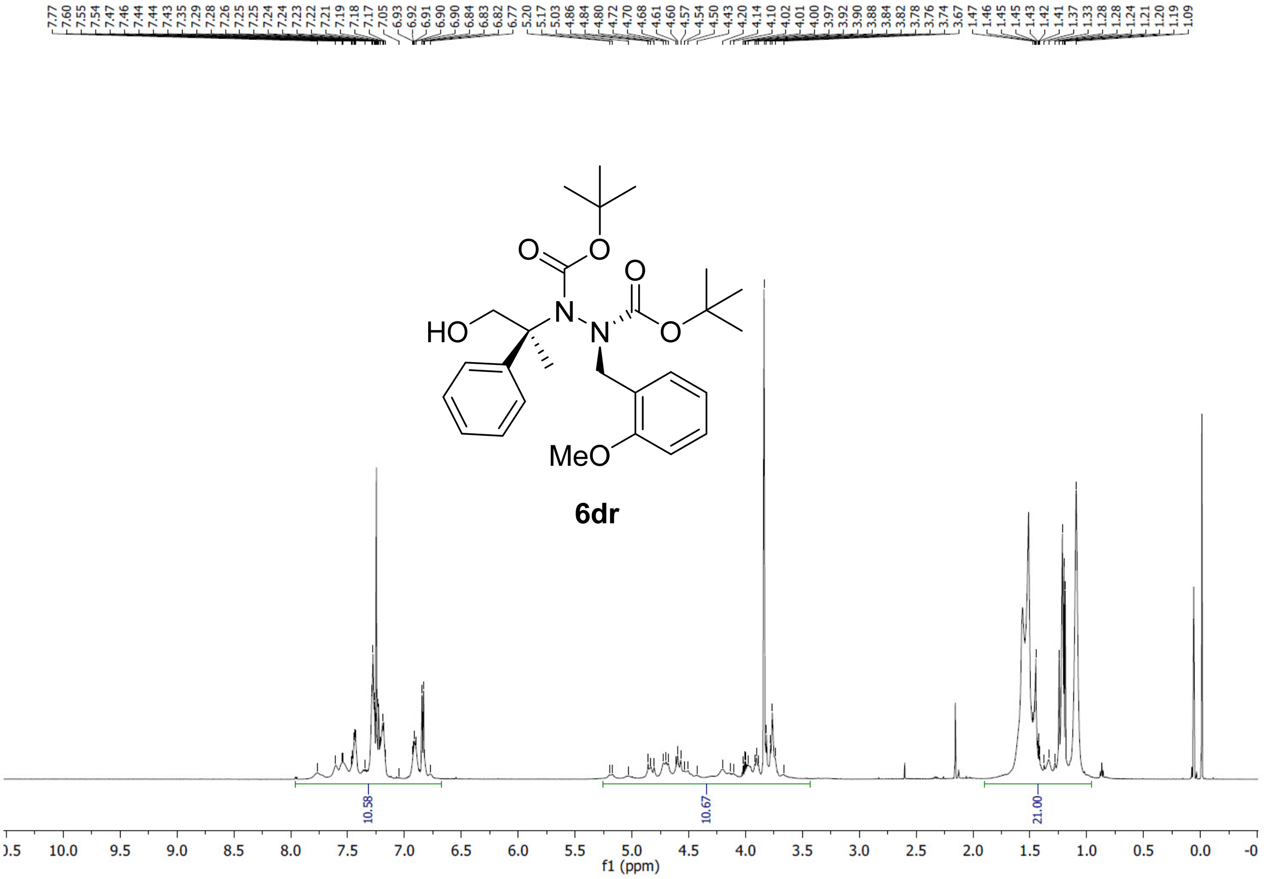

6dr
f1 (ppm)

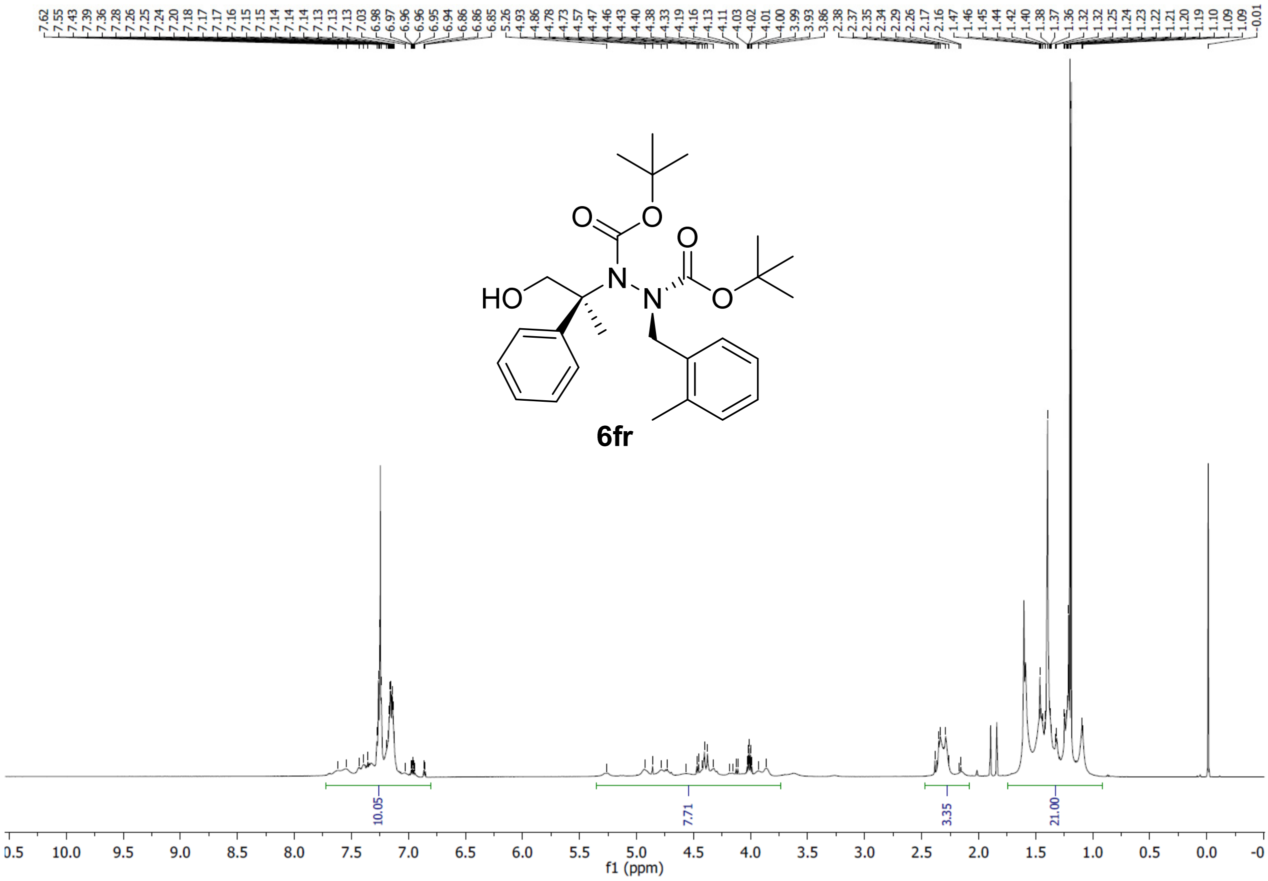

6fr
f1 (ppm)

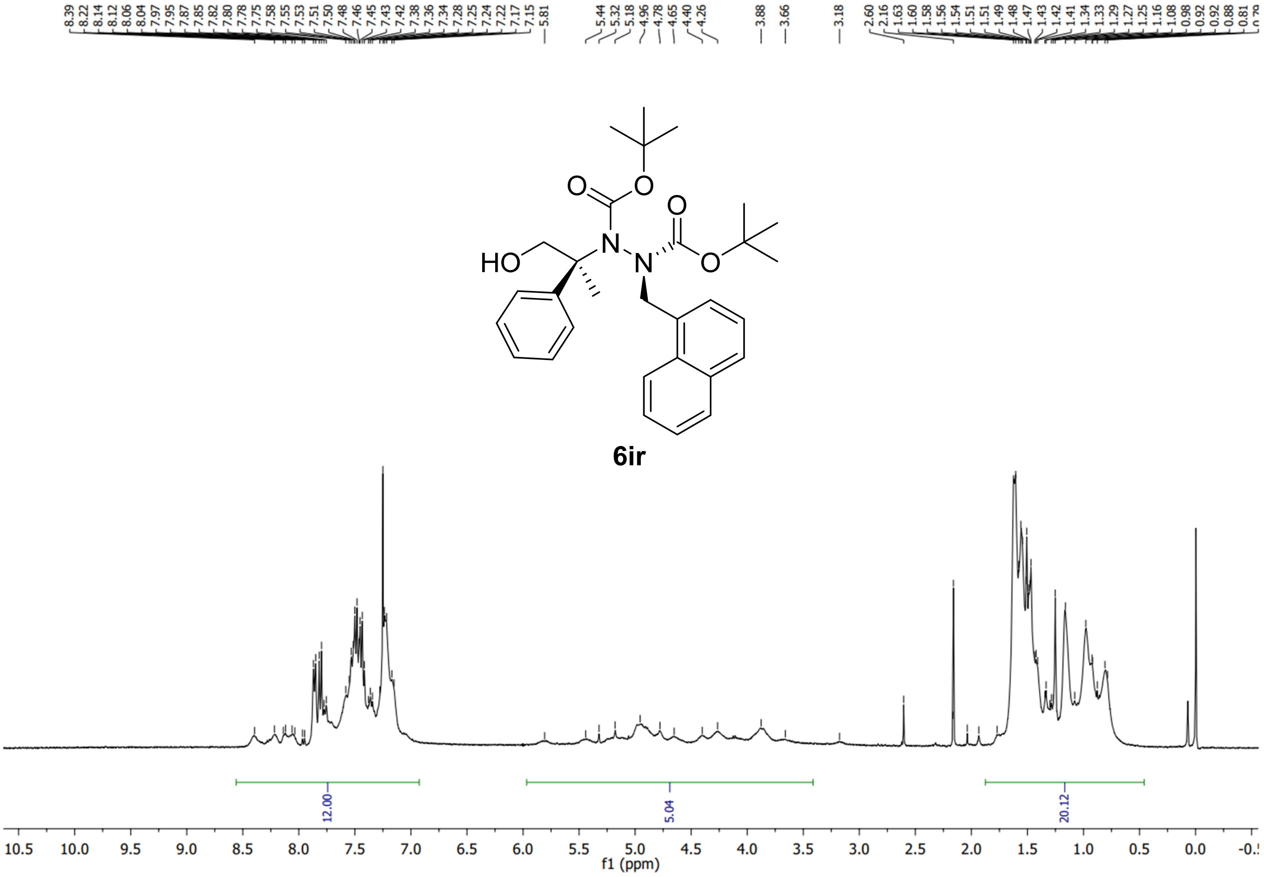
6ir
f1 (ppm)

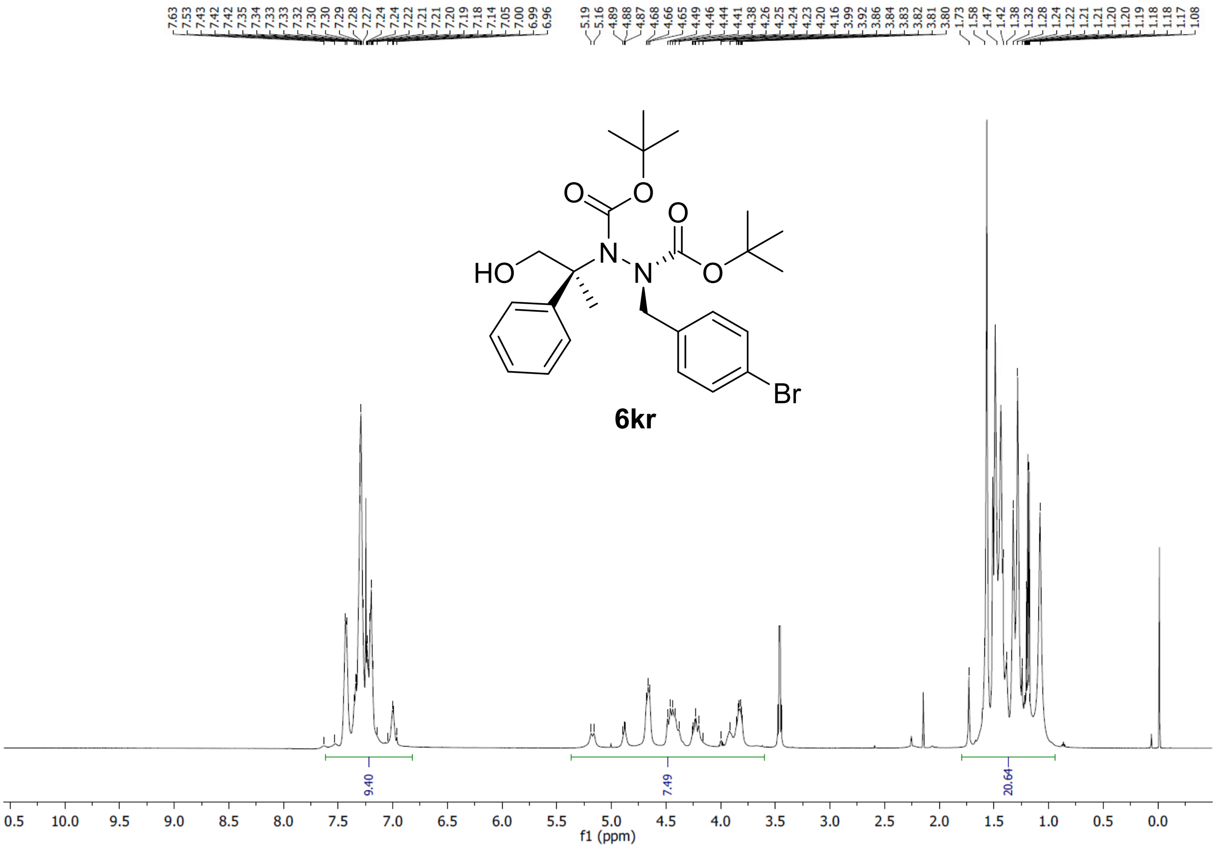
6kr
f1 (ppm)

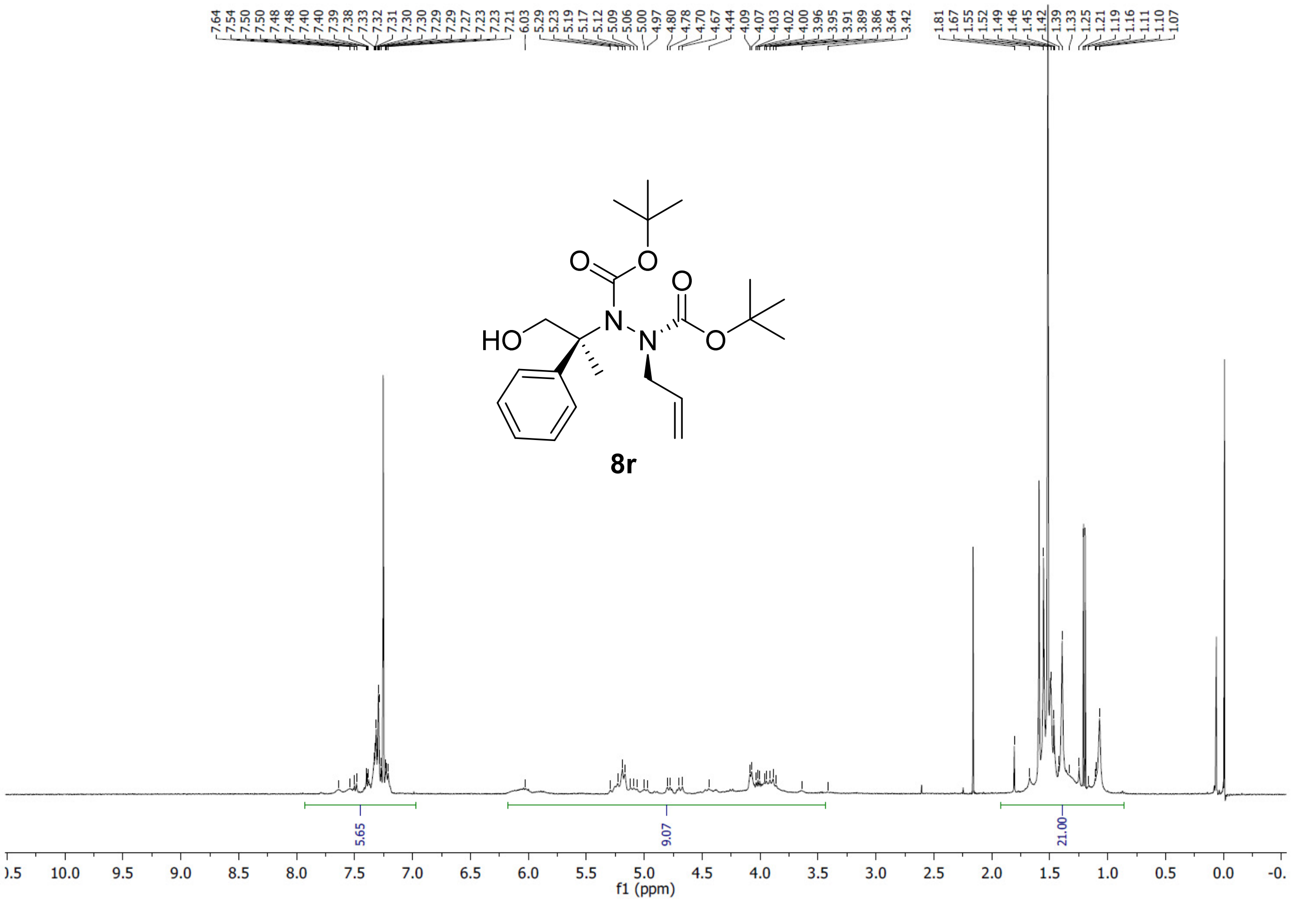
8r
f1 (ppm)

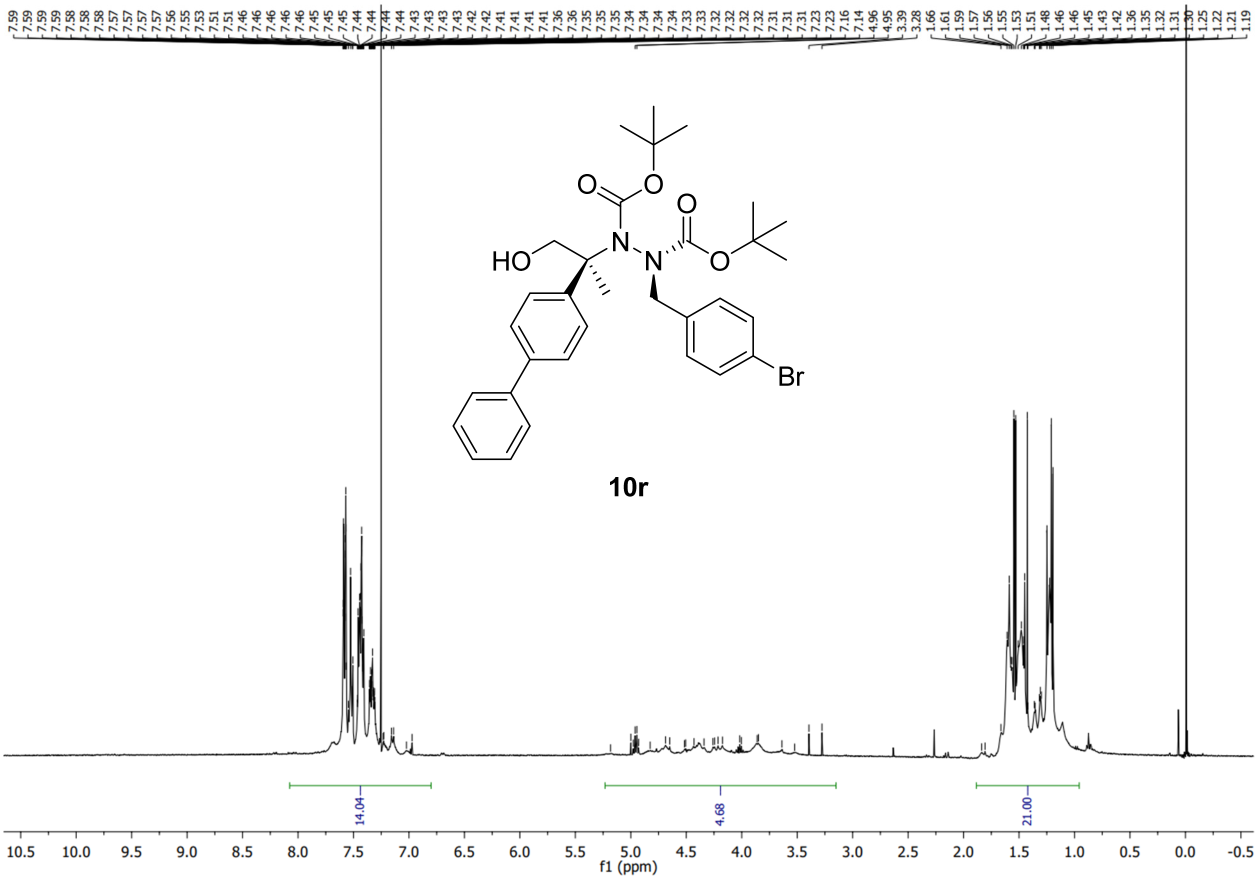
10r
f1 (ppm)

5ao

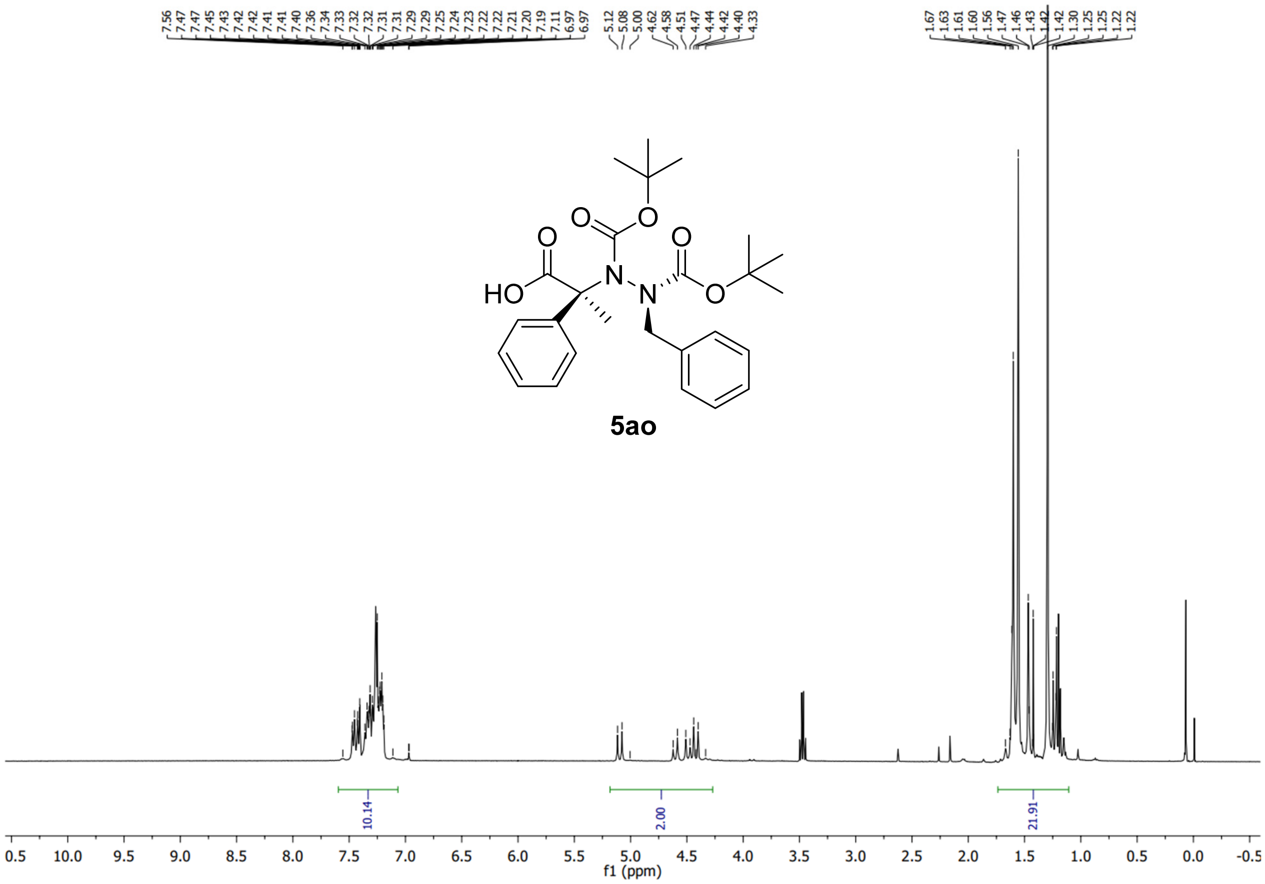
7.56 7.47 7.47 7.45 7.43 7.42 7.42 7.41 7.40 7.36 7.34 7.33 7.32 7.32 7.31 7.31 7.29 7.29 7.25 7.24 7.23 7.22 7.22 7.21 7.20 7.19 7.11 6.97 6.97
5.12 5.08 5.00 4.62 4.58 4.51 4.47 4.44 4.42 4.40 4.33
1.67 1.63 1.61 1.60 1.56 1.47 1.46 1.43 1.42 1.42 1.30 1.25 1.25 1.22 1.22
10.14
2.00
21.91
0.5 10.0 9.5 9.0 8.5 8.0 7.5 7.0 6.5 6.0 5.5 5.0 4.5 4.0 3.5 3.0 2.5 2.0 1.5 1.0 0.5 0.0 -0.5
f1 (ppm)

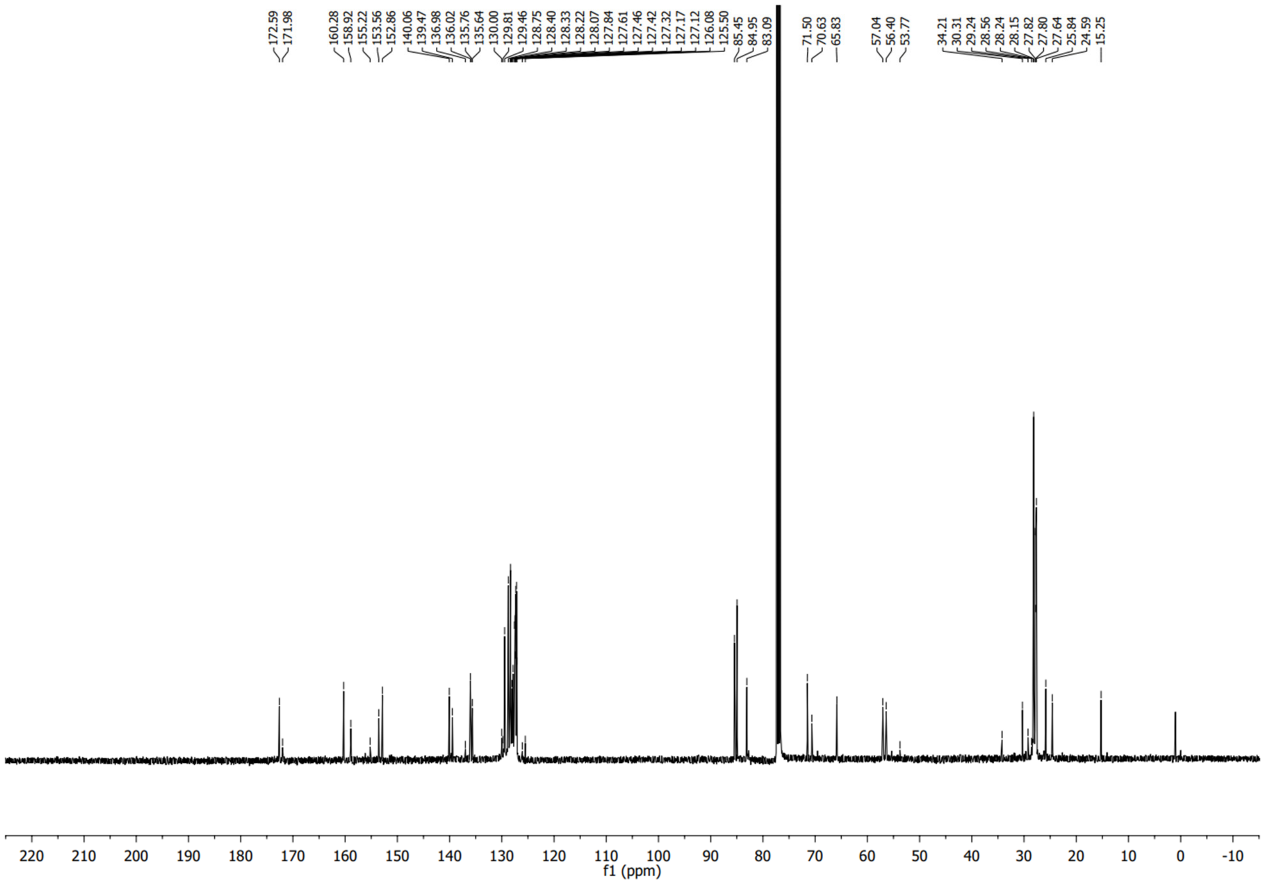
172.59 171.98
160.28 158.92 155.22 153.56 152.86 140.06 139.47 136.98 136.02 135.76 135.64 130.00 129.81 129.46 128.75 128.40 128.33 128.22 128.07 127.84 127.61 127.46 127.42 127.32 127.17 127.12 126.08 125.50
85.45 84.95 83.09
71.50 70.63 65.83
57.04 56.40 53.77
34.21 30.31 29.24 28.56 28.24 28.15 27.82 27.80 27.64 25.84 24.59 15.25
220 210 200 190 180 170 160 150 140 130 120 110 100 90 80 70 60 50 40 30 20 10 0 -10
f1 (ppm)

HPCL traces

Data File C:\CHEM32\1\DATA\CP\MC_54_2.D
Sample Name: MC_54_2

===

Acq. Operator : Chiara
Acq. Instrument : HPLC-1 Location : Vial 1
Injection Date : 23/02/2021 13:39:21
Acq. Method : C:\CHEM32\1\METHODS\DEF_LC.M
Last changed : 23/02/2021 13:31:10 by Chiara
(modified after loading)
Analysis Method : C:\CHEM32\1\METHODS\DEF_LC.M
Last changed : 24/02/2021 18:09:49 by Chiara
(modified after loading)
Sample Info : MC_54_2, 1.0 mL/min, 98:2 hex:ipr, 25°C, IC

Additional Info : Peak(s) manually integrated

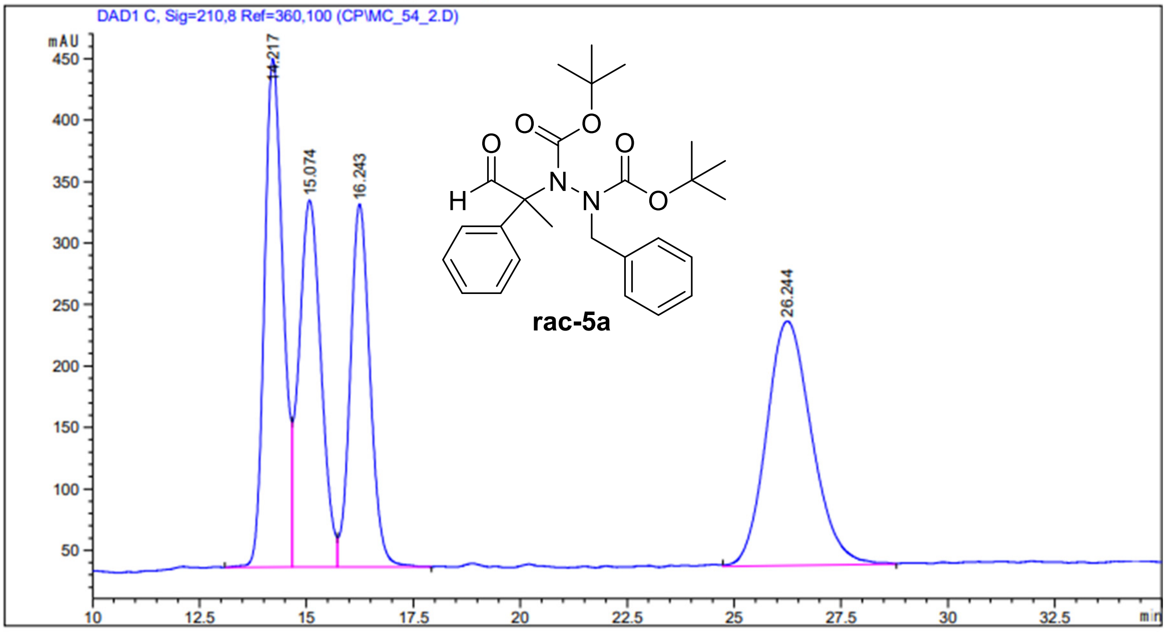

===

Area Percent Report

===

Sorted By : Signal
Multiplier: : 1.0000
Dilution: : 1.0000
Use Multiplier & Dilution Factor with ISTDs

Signal 1: DAD1 C, Sig=210,8 Ref=360,100

Peak #	RetTime [min]	Type	Width [min]	Area [mAU*s]	Height [mAU]	Area %
1	14.217	BV	0.4660	1.25196e4	412.61871	26.4103
2	15.074	VV	0.5484	1.07350e4	297.93188	22.6456
3	16.243	VB	0.5045	9504.39063	294.48352	20.0496
4	26.244	VV	1.1159	1.46453e4	198.70433	30.8945

Totals : 4.74044e4 1203.73845

Data File C:\CHEM32\1\DATA\GC\GC_334.D
Sample Name: GC_334.D

===

Acq. Operator : Chiara
Acq. Instrument : HPLC-1 Location : Vial 1
Injection Date : 10/12/2021 15:04:47
Acq. Method : C:\CHEM32\1\METHODS\DEF_LC.M
Last changed : 10/12/2021 14:42:19 by Chiara
(modified after loading)
Analysis Method : C:\CHEM32\1\METHODS\DEF_LC.M
Last changed : 18/05/2022 15:43:08 by Giovanni
(modified after loading)
Sample Info : GC_334, 1 mL/min, 98:2 hex:ipr, 25°C, IC

Additional Info : Peak(s) manually integrated

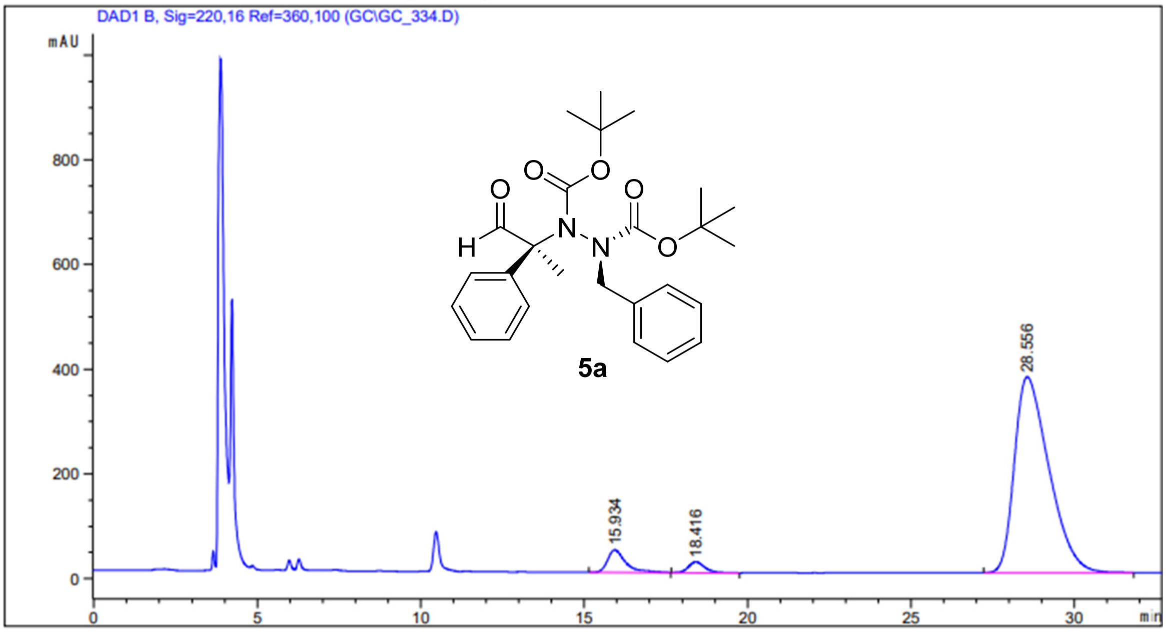

===
Area Percent Report
===

Sorted By : Signal
Multiplier: : 1.0000
Dilution: : 1.0000
Use Multiplier & Dilution Factor with ISTDs

Signal 1: DAD1 B, Sig=220,16 Ref=360,100

Peak #	RetTime [min]	Type	Width [min]	Area [mAU*s]	Height [mAU]	Area %
1	15.934	BB	0.5672	1646.84863	42.95967	5.5226
2	18.416	BB	0.5263	704.22192	20.42389	2.3616
3	28.556	BB	1.1208	2.74691e4	374.05396	92.1158

Totals : 2.98201e4 437.43751

Data File C:\CHEM32\1\DATA\CP\CP_233_R.D
Sample Name: CP_233_R

===

Acq. Operator : Chiara
Acq. Instrument : HPLC-1 Location : Vial 1
Injection Date : 25/03/2022 16:38:16
Acq. Method : C:\CHEM32\1\METHODS\DEF_LC.M
Last changed : 25/03/2022 16:36:55 by Chiara
(modified after loading)
Analysis Method : C:\CHEM32\1\METHODS\DEF_LC.M
Last changed : 18/05/2022 15:47:25 by Giovanni
(modified after loading)
Sample Info : CP_233_R, 1 mL/min, 98:2 hex:ipr, 25°C, IC

Additional Info : Peak(s) manually integrated

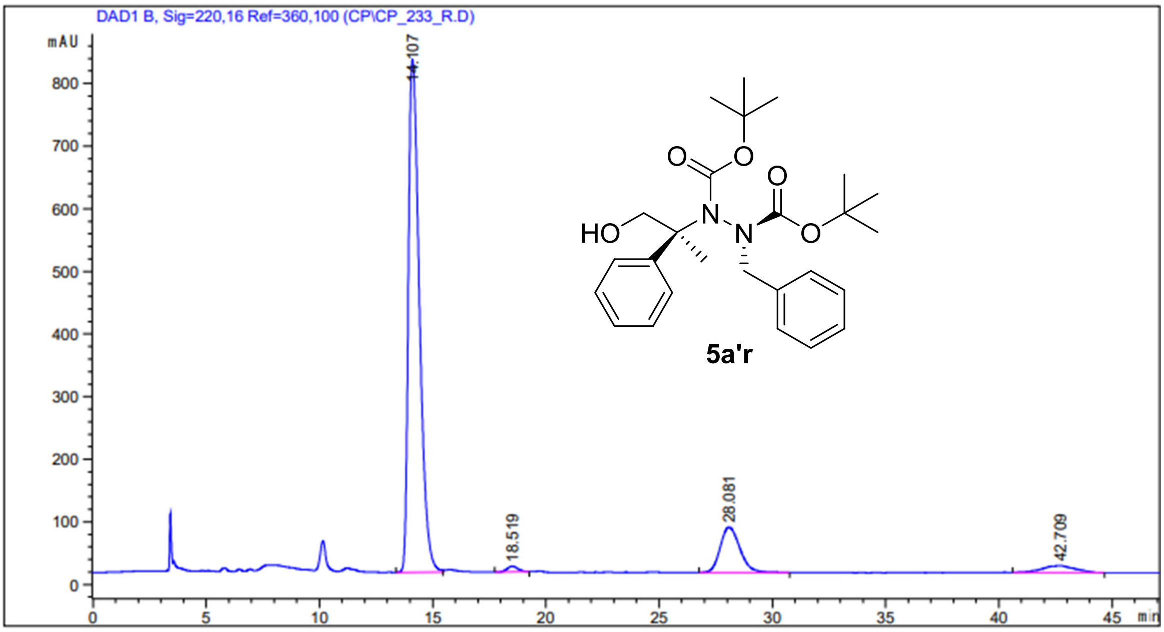

===
Area Percent Report
===

Sorted By : Signal
Multiplier: : 1.0000
Dilution: : 1.0000
Use Multiplier & Dilution Factor with ISTDs

Signal 1: DAD1 B, Sig=220,16 Ref=360,100

Peak #	RetTime [min]	Type	Width [min]	Area [mAU*s]	Height [mAU]	Area %
1	14.107	BV	0.5232	2.76049e4	819.14893	82.6333
2	18.519	BB	0.5194	301.73871	8.94777	0.9032
3	28.081	BB	0.9441	4370.65576	72.41911	13.0832
4	42.709	BB	1.2980	1129.23645	11.03340	3.3803

Totals : 3.34066e4 911.54920

ata File C:\CHEM32\1\DATA\CP\CP_234_R.D
ample Name: CP_234_R

===

Acq. Operator : Chiara
Acq. Instrument : HPLC-1 Location : Vial 1
Injection Date : 25/03/2022 14:46:52
Acq. Method : C:\CHEM32\1\METHODS\DEF_LC.M
Last changed : 25/03/2022 14:46:16 by Chiara
(modified after loading)
Analysis Method : C:\CHEM32\1\METHODS\DEF_LC.M
Last changed : 18/05/2022 15:56:19 by Giovanni
(modified after loading)
Sample Info : CP_234_R, 1 mL/min, 98:2 hex:ipr, 25°C, IC

Additional Info : Peak(s) manually integrated

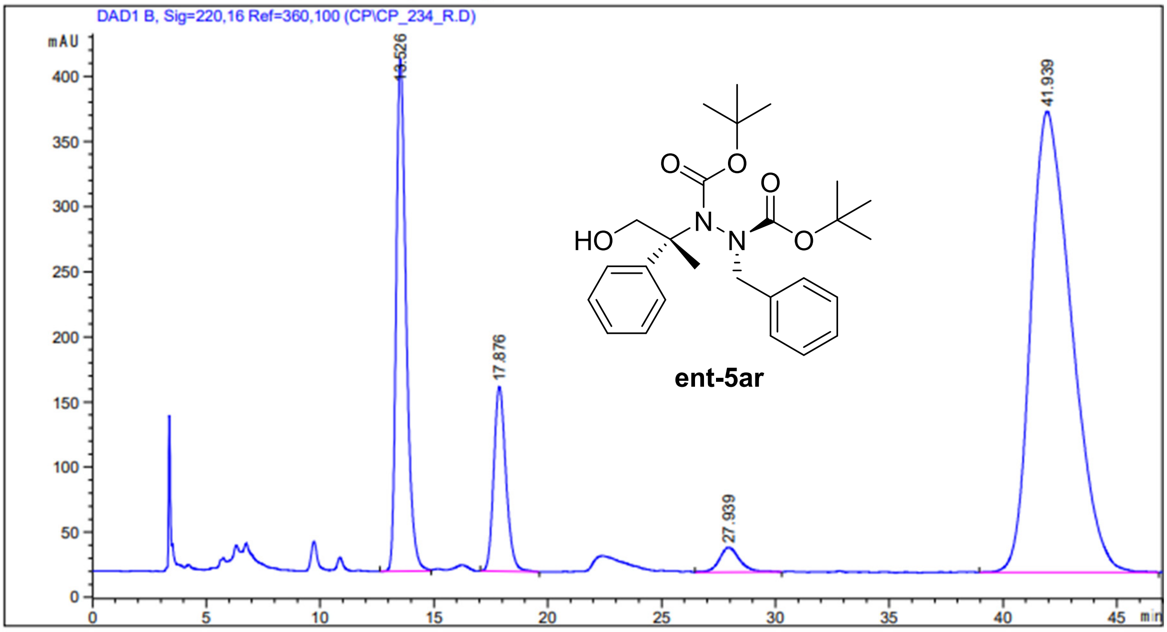

===
Area Percent Report
===

Sorted By : Signal
Multiplier: : 1.0000
Dilution: : 1.0000
Use Multiplier & Dilution Factor with ISTDs

Signal 1: DAD1 B, Sig=220,16 Ref=360,100

Peak #	RetTime [min]	Type	Width [min]	Area [mAU*s]	Height [mAU]	Area %
1	13.526	BV	0.4639	1.19908e4	393.02396	19.2553
2	17.876	BB	0.5690	5265.57910	141.88268	8.4557
3	27.939	BB	0.9515	1178.91699	18.80141	1.8932
4	41.939	VB	1.9417	4.38373e4	353.75073	70.3958

Totals : 6.22725e4 907.45877

Data File C:\CHEM32\1\DATA\CP\CP_235_R.D
Sample Name: CP_235_R

===

Acq. Operator : Chiara
Acq. Instrument : HPLC-1 Location : Vial 1
Injection Date : 28/03/2022 15:40:19
Acq. Method : C:\CHEM32\1\METHODS\DEF_LC.M
Last changed : 28/03/2022 14:41:27 by Alberto
(modified after loading)
Analysis Method : C:\CHEM32\1\METHODS\DEF_LC.M
Last changed : 18/05/2022 15:55:24 by Giovanni
(modified after loading)
Sample Info : CP_235_R, 1 mL/min, 98:2 hex:ipr, 25°C, IC

Additional Info : Peak(s) manually integrated

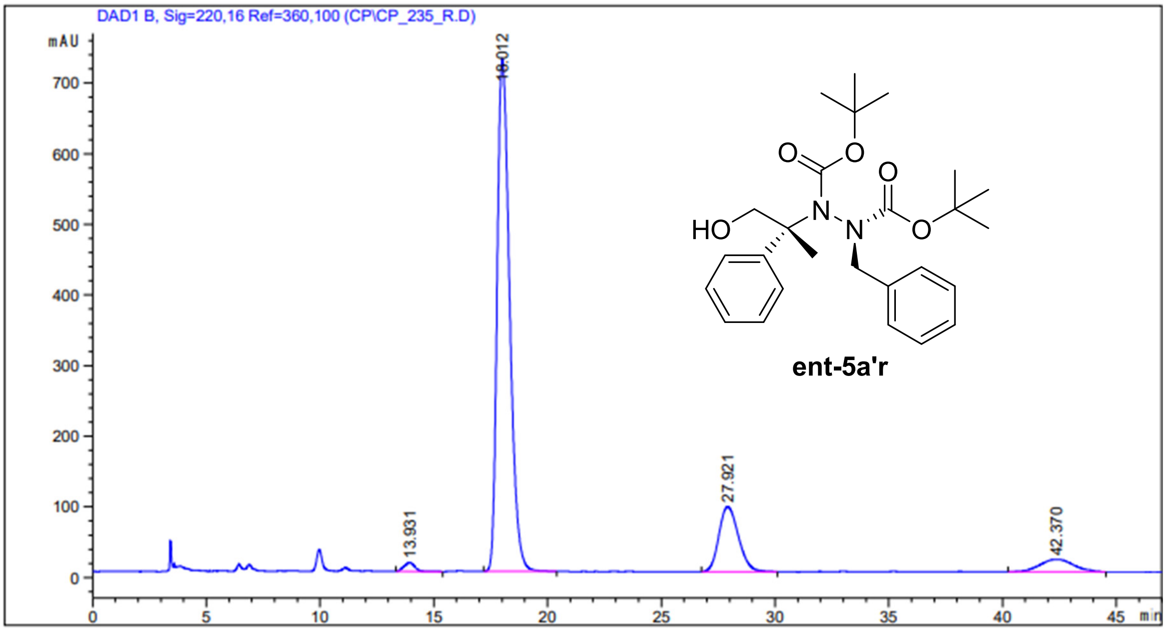

===
Area Percent Report
===

Sorted By : Signal
Multiplier: : 1.0000
Dilution: : 1.0000
Use Multiplier & Dilution Factor with ISTDs

Signal 1: DAD1 B, Sig=220,16 Ref=360,100

Peak #	RetTime [min]	Type	Width [min]	Area [mAU*s]	Height [mAU]	Area %
1	13.931	BB	0.4947	417.59653	12.47040	1.1816
2	18.012	BB	0.5999	2.76104e4	725.60986	78.1230
3	27.921	BB	0.9296	5496.96240	91.92726	15.5535
4	42.370	BB	1.2393	1817.24609	17.44482	5.1419

Totals : 3.53422e4 847.45235

Data File H:\HPLC\1\DATA\GC\GC_435.D
Sample Name: GC_435

===

Acq. Operator : Chiara
Acq. Instrument : HPLC-1 Location : Vial 1
Injection Date : 09/06/2022 16:45:51
Acq. Method : C:\CHEM32\1\METHODS\DEF_LC.M
Last changed : 09/06/2022 16:45:02 by Chiara
(modified after loading)
Analysis Method : H:\HPLC\2\METHODS\DEF_LC.M
Last changed : 21/06/2022 22:53:21
(modified after loading)
Sample Info : GC_435, 1 mL/min, 98:2 hex:ipr, 25°C, IC

Additional Info : Peak(s) manually integrated

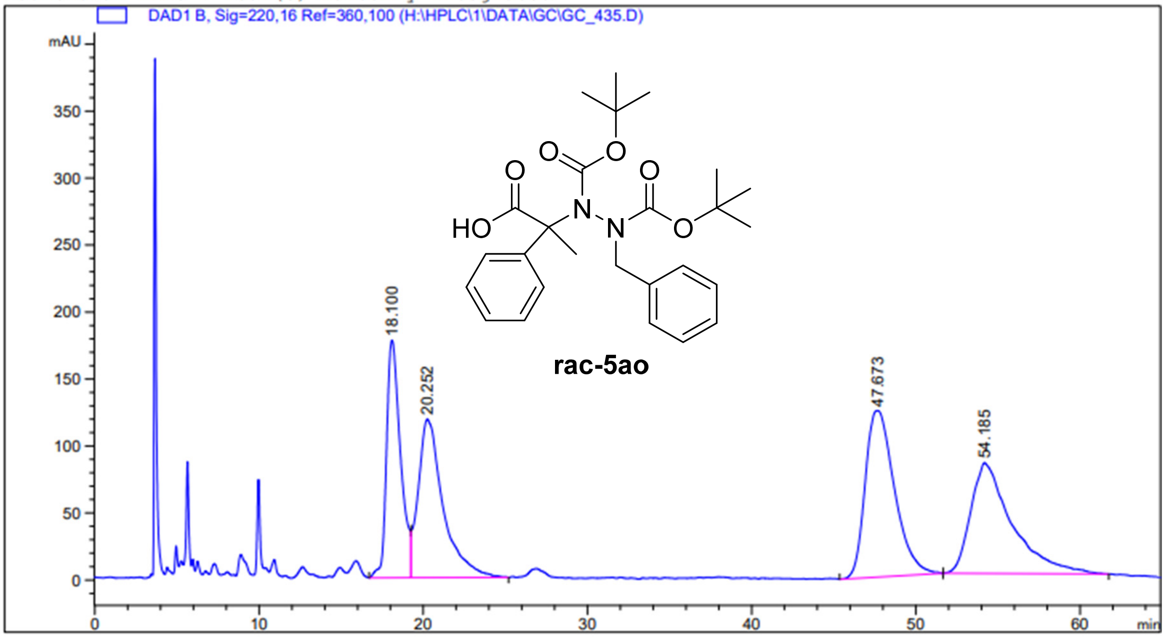

===
Area Percent Report
===

Sorted By : Signal
Multiplier: : 1.0000
Dilution: : 1.0000
Use Multiplier & Dilution Factor with ISTDs

Signal 1: DAD1 B, Sig=220,16 Ref=360,100

Peak #	RetTime [min]	Type	Width [min]	Area [mAU*s]	Height [mAU]	Area %
1	18.100	BV	0.8961	1.04589e4	177.31779	19.8196
2	20.252	VB	1.3715	1.24886e4	118.31341	23.6658
3	47.673	BB	1.8663	1.53991e4	124.21582	29.1811
4	54.185	BB	2.1390	1.44241e4	82.69429	27.3335

Totals : 5.27707e4 502.54131

Data File H:\HPLC\1\DATA\GC\GC_438.D
Sample Name: GC_438

===

Acq. Operator : Giovanni
Acq. Instrument : HPLC-1 Location : Vial 1
Injection Date : 10/06/2022 18:05:57
Acq. Method : C:\CHEM32\1\METHODS\DEF_LC.M
Last changed : 10/06/2022 18:05:30 by Giovanni
(modified after loading)
Analysis Method : H:\HPLC\2\METHODS\DEF_LC.M
Last changed : 21/06/2022 22:55:20
(modified after loading)
Sample Info : GC_438, 1 mL/min, 98:2 hex:ipr, 25°C, IC

Additional Info : Peak(s) manually integrated

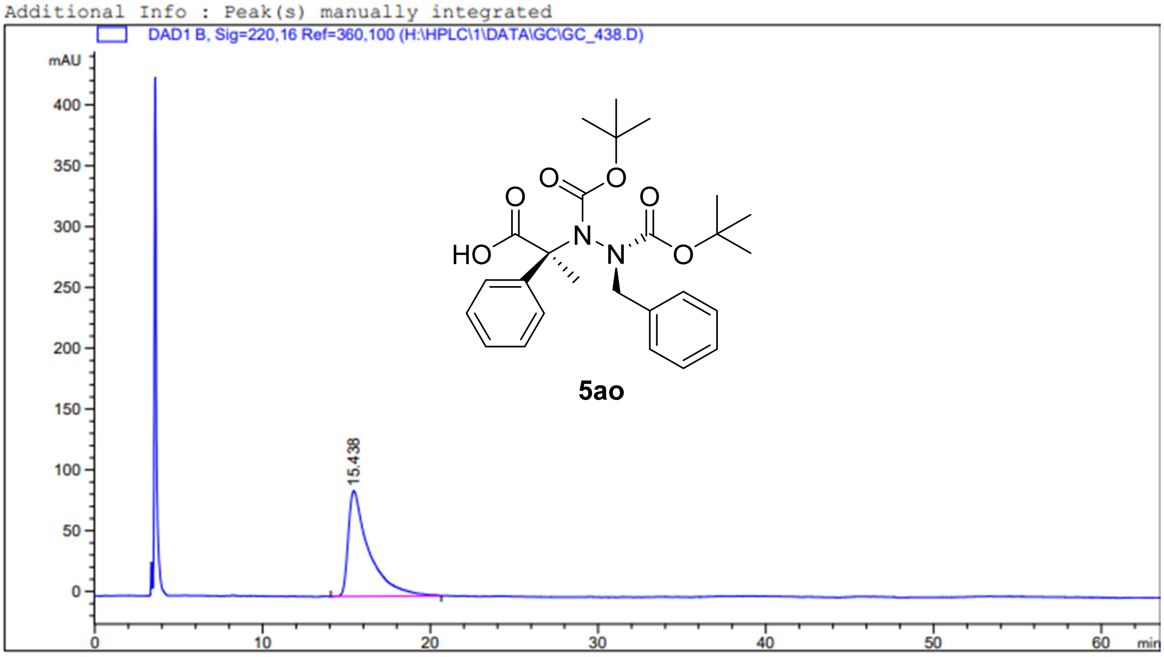

===
Area Percent Report
===

Sorted By : Signal
Multiplier: : 1.0000
Dilution: : 1.0000
Use Multiplier & Dilution Factor with ISTDs

Signal 1: DAD1 B, Sig=220,16 Ref=360,100

Peak #	RetTime [min]	Type	Width [min]	Area [mAU*s]	Height [mAU]	Area %
1	15.438	BB	1.1907	7499.94482	86.74514	100.0000

Totals : 7499.94482 86.74514

Data File C:\CHEM32\1\DATA\CP\CP_141_LUXC.D
Sample Name: CP_141_LuxC

===

Acq. Operator : Chiara
Acq. Instrument : HPLC-1 Location : Vial 1
Injection Date : 17/03/2022 14:13:25
Acq. Method : C:\CHEM32\1\METHODS\DEF_LC.M
Last changed : 17/03/2022 14:12:55 by Chiara
(modified after loading)
Analysis Method : C:\CHEM32\1\METHODS\DEF_LC.M
Last changed : 22/04/2022 17:39:59 by Chiara
(modified after loading)
Sample Info : CP_141_LuxC, 1 mL/min, 98:2 hex:ipr, 25°C, Lux 5u Cellulose-2

Additional Info : Peak(s) manually integrated

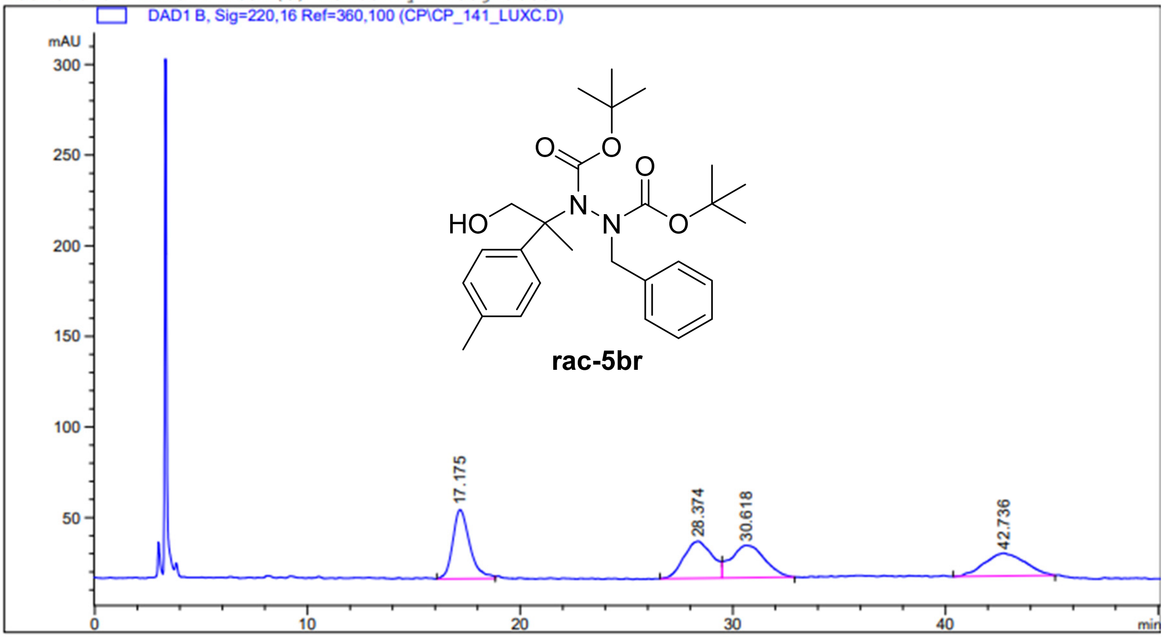

===
Area Percent Report
===

Sorted By : Signal
Multiplier: : 1.0000
Dilution: : 1.0000
Use Multiplier & Dilution Factor with ISTDs

Signal 1: DAD1 B, Sig=220,16 Ref=360,100

Peak #	RetTime [min]	Type	Width [min]	Area [mAU*s]	Height [mAU]	Area %
1	17.175	BV	0.8410	2130.24976	38.17538	27.7597
2	28.374	BV	1.1798	1904.16736	20.42281	24.8136
3	30.618	VB	1.4154	1975.45605	17.84628	25.7425
4	42.736	BB	1.6009	1664.02673	12.61544	21.6842

Totals : 7673.89990 89.05992

```
Data File C:\CHEM32\1\DATA\CP\CP_254_R.D
Sample Name: CP_254_R

    =====================================================================
    Acq. Operator   : Chiara
    Acq. Instrument : HPLC-1                          Location : Vial 1
    Injection Date  : 22/04/2022 11:31:14
    Acq. Method     : C:\CHEM32\1\METHODS\DEF_LC.M
    Last changed    : 22/04/2022 11:28:01 by Chiara
                      (modified after loading)
    Analysis Method : C:\CHEM32\1\METHODS\DEF_LC.M
    Last changed    : 22/04/2022 17:37:28 by Chiara
                      (modified after loading)
    Sample Info     : CP_254_R, 1 mL/min, 98:2 hex:ipr, 25°C, Lux 5u Cellulos
                      e

    Additional Info : Peak(s) manually integrated
```

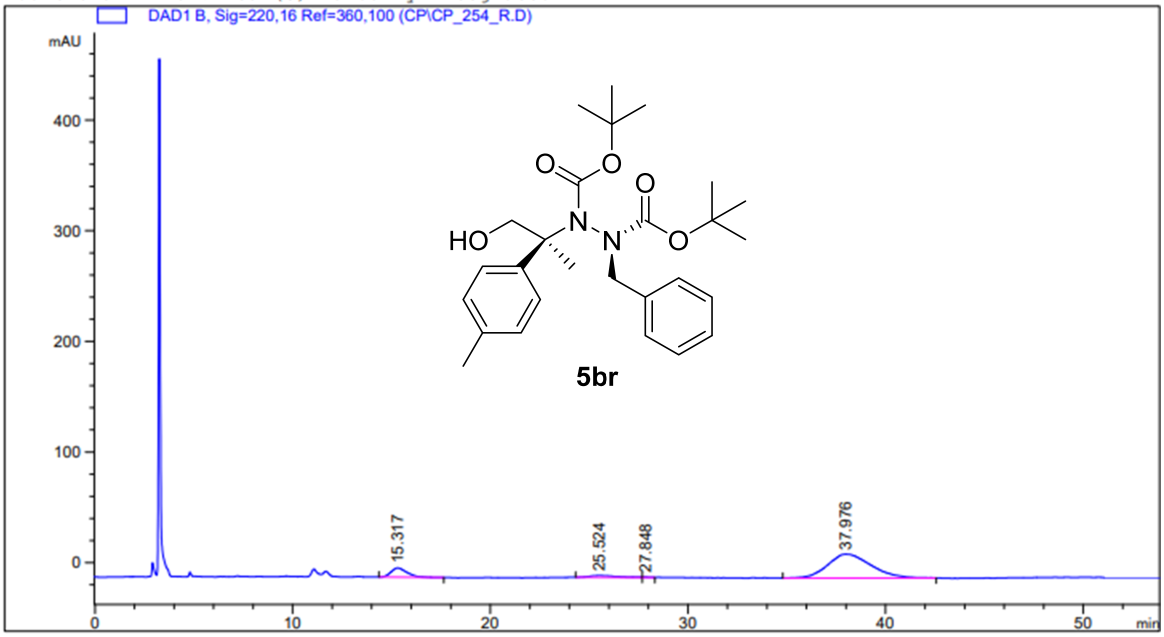


```
    =====================================================================
                              Area Percent Report
    =====================================================================

    Sorted By             :      Signal
    Multiplier:                  :      1.0000
    Dilution:                    :      1.0000
    Use Multiplier & Dilution Factor with ISTDs


    Signal 1: DAD1 B, Sig=220,16 Ref=360,100
```

Peak #	RetTime [min]	Type	Width [min]	Area [mAU*s]	Height [mAU]	Area %
1	15.317	BB	0.8531	479.38226	8.30861	12.1362
2	25.524	BV	1.1480	157.74081	1.67915	3.9934
3	27.848	VB	0.2966	6.85585	3.21790e-1	0.1736
4	37.976	BB	1.9262	3306.05005	21.54502	83.6969

Totals : 3950.02898 31.85456

Data File C:\CHEM32\1\DATA\CP\CP_208_R_LUXC.D
Sample Name: CP_208_R_LuxC

===

Acq. Operator : Chiara
Acq. Instrument : HPLC-1 Location : Vial 1
Injection Date : 09/03/2022 12:27:27
Acq. Method : C:\CHEM32\1\METHODS\DEF_LC.M
Last changed : 09/03/2022 12:25:28 by Chiara
(modified after loading)
Analysis Method : C:\CHEM32\1\METHODS\DEF_LC.M
Last changed : 22/04/2022 17:56:54 by Chiara
(modified after loading)
Sample Info : CP_208_R_LuxC, 1 mL/min, 95:5 hex:ipr, 25°C, lux 5u cellulose-2

Additional Info : Peak(s) manually integrated

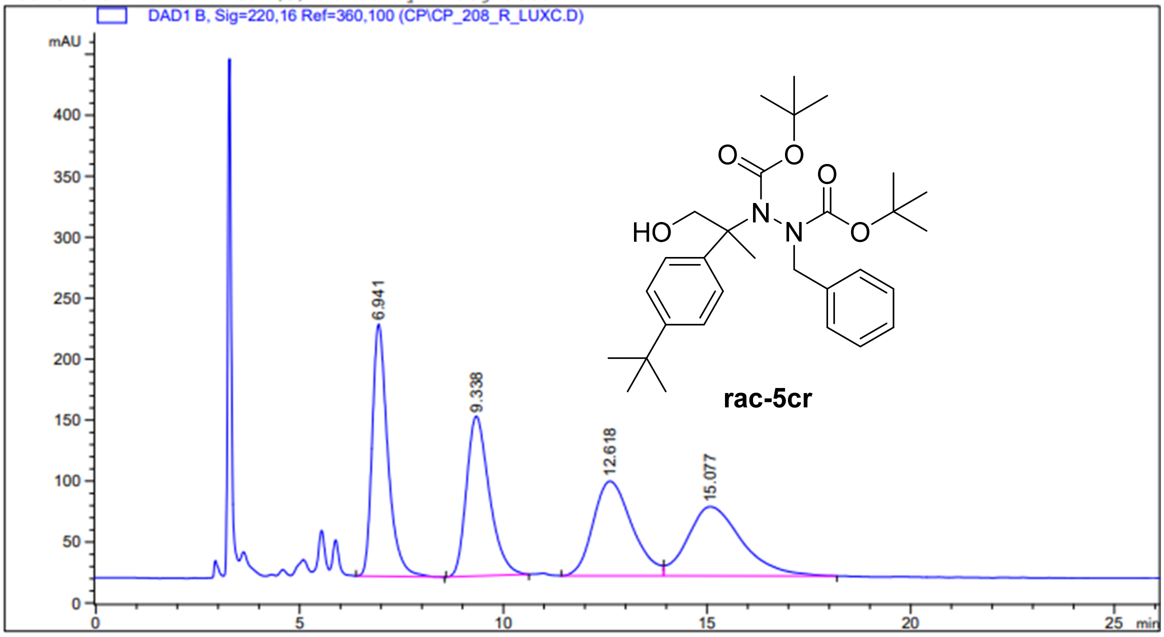

===
Area Percent Report
===

Sorted By : Signal
Multiplier: : 1.0000
Dilution: : 1.0000
Use Multiplier & Dilution Factor with ISTDs

Signal 1: DAD1 B, Sig=220,16 Ref=360,100

Peak #	RetTime [min]	Type	Width [min]	Area [mAU*s]	Height [mAU]	Area %
1	6.941	BB	0.4023	5497.83203	206.72803	26.1940
2	9.338	BB	0.6018	5122.81299	131.12161	24.4072
3	12.618	BV	1.0064	5136.26514	77.58142	24.4713
4	15.077	VB	1.3805	5232.02393	56.70445	24.9275

Totals : 2.09889e4 472.13551

Data File C:\CHEM32\1\DATA\CP\CP_215_R_LUXC.D
Sample Name: CP_215_R_LuxC

===

Acq. Operator : Chiara
Acq. Instrument : HPLC-1 Location : Vial 1
Injection Date : 10/03/2022 15:36:51
Acq. Method : C:\CHEM32\1\METHODS\DEF_LC.M
Last changed : 10/03/2022 15:22:31 by Chiara
(modified after loading)
Analysis Method : C:\CHEM32\1\METHODS\DEF_LC.M
Last changed : 22/04/2022 17:58:37 by Chiara
(modified after loading)
Sample Info : CP_215_R_LuxC, 1 mL/min, 95:5 hex:ipr, 25°C, LuxC

Additional Info : Peak(s) manually integrated

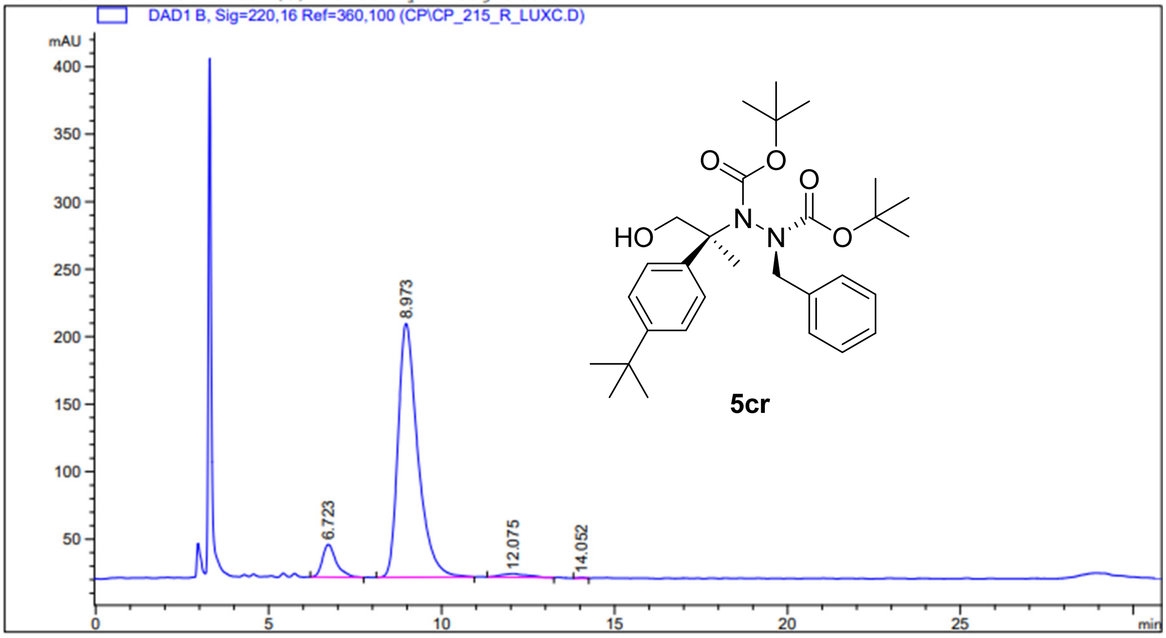

===
Area Percent Report
===

Sorted By : Signal
Multiplier: : 1.0000
Dilution: : 1.0000
Use Multiplier & Dilution Factor with ISTDs

Signal 1: DAD1 B, Sig=220,16 Ref=360,100

Peak #	RetTime [min]	Type	Width [min]	Area [mAU*s]	Height [mAU]	Area %
1	6.723	BB	0.4193	675.41315	24.22640	8.1690
2	8.973	BB	0.5939	7445.16797	188.05902	90.0482
3	12.075	BB	0.6714	140.41481	2.65319	1.6983
4	14.052	BB	0.1892	6.98399	5.85274e-1	0.0845

Totals : 8267.97991 215.52389

Data File C:\CHEM32\1\DATA\CP\CP_209_RIDOTTO.D
Sample Name: CP_209_ridotto

===

Acq. Operator : Chiara
Acq. Instrument : HPLC-1 Location : Vial 1
Injection Date : 02/02/2022 13:57:29
Acq. Method : C:\CHEM32\1\METHODS\DEF_LC.M
Last changed : 02/02/2022 13:56:41 by Giovanni
(modified after loading)
Analysis Method : C:\CHEM32\1\METHODS\DEF_LC.M
Last changed : 22/04/2022 18:00:41 by Chiara
(modified after loading)
Sample Info : CP_209_ridotto, 1 mL/min, 98:2 hex:ipr, 25°C, IC

Additional Info : Peak(s) manually integrated

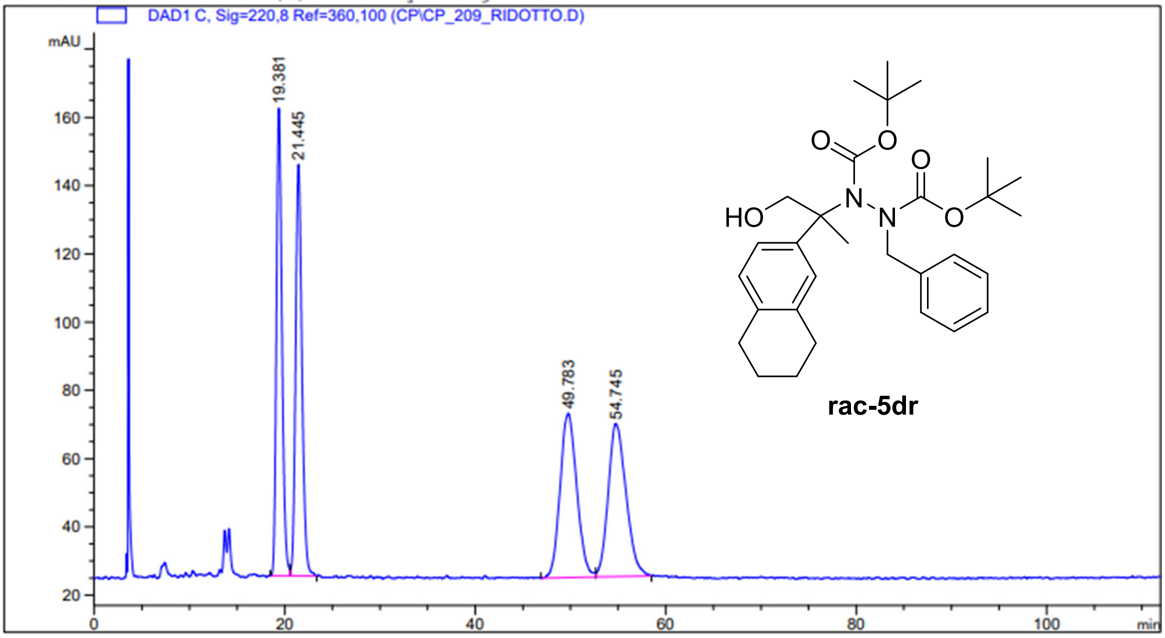

===
Area Percent Report
===

Sorted By : Signal
Multiplier: : 1.0000
Dilution: : 1.0000
Use Multiplier & Dilution Factor with ISTDs

Signal 1: DAD1 C, Sig=220,8 Ref=360,100

Peak #	RetTime [min]	Type	Width [min]	Area [mAU*s]	Height [mAU]	Area %
1	19.381	BV	0.6305	5571.86377	137.00578	24.3925
2	21.445	VB	0.7019	5559.34180	120.58350	24.3377
3	49.783	BV	1.7572	5865.04834	48.14730	25.6760
4	54.745	VB	1.6920	5846.29053	44.87307	25.5939
Totals :				2.28425e4	350.60965	

Data File H:\HPLC\1\DATA\CP\CP_219_R.D
Sample Name: CP_219_R

===

Acq. Operator : Chiara
Acq. Instrument : HPLC-1 Location : Vial 1
Injection Date : 18/02/2022 12:55:07
Acq. Method : C:\CHEM32\1\METHODS\DEF_LC.M
Last changed : 18/02/2022 12:10:40 by Chiara
(modified after loading)
Analysis Method : H:\HPLC\2\METHODS\DEF_LC.M
Last changed : 06/06/2022 10:31:47
(modified after loading)
Sample Info : CP_219_R, 1 mL/min, 98:2 hex:ipr, 25°C, IC

Additional Info : Peak(s) manually integrated

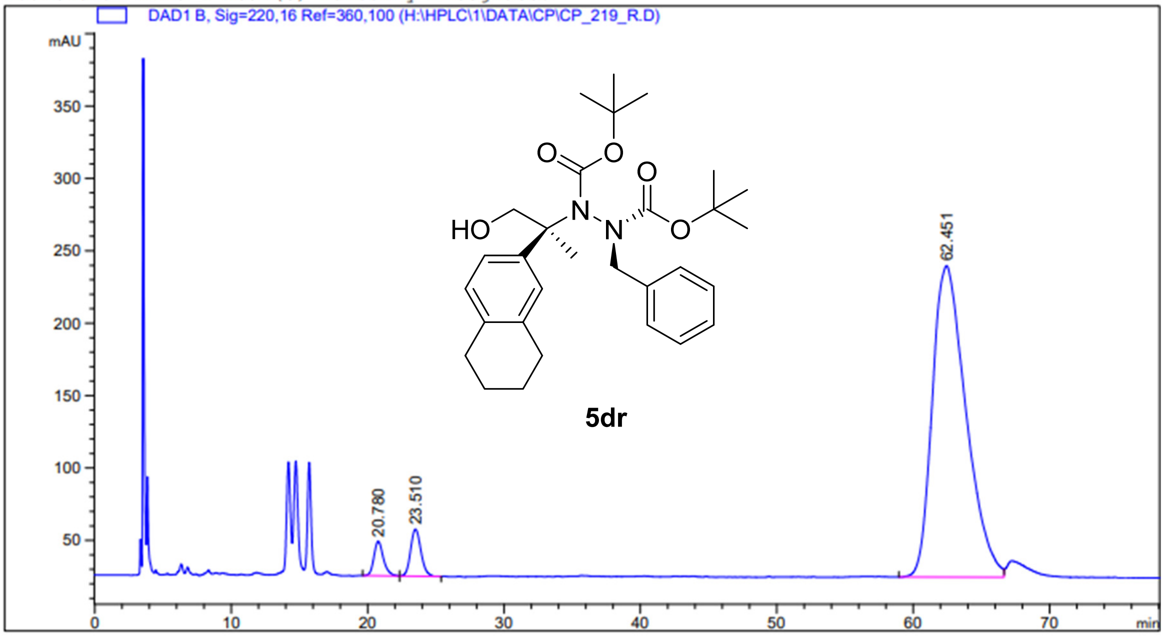

5dr

===
Area Percent Report
===

Sorted By : Signal
Multiplier: : 1.0000
Dilution: : 1.0000
Use Multiplier & Dilution Factor with ISTDs

Signal 1: DAD1 B, Sig=220,16 Ref=360,100

Peak #	RetTime [min]	Type	Width [min]	Area [mAU*s]	Height [mAU]	Area %
1	20.780	BB	0.7421	1154.79504	23.79881	2.9082
2	23.510	BB	0.8194	1711.21057	32.44208	4.3095
3	62.451	BV	2.5069	3.68421e4	215.02814	92.7823

Totals : 3.97081e4 271.26903

Data File C:\CHEM32\1\DATA\CP\CP_211_RIDOTTO.D
Sample Name: CP_211_ridotto

===

Acq. Operator : Chiara
Acq. Instrument : HPLC-1 Location : Vial 1
Injection Date : 02/02/2022 15:50:26
Acq. Method : C:\CHEM32\1\METHODS\DEF_LC.M
Last changed : 02/02/2022 15:49:49 by Chiara
(modified after loading)
Analysis Method : C:\CHEM32\1\METHODS\DEF_LC.M
Last changed : 18/05/2022 17:12:22 by Giovanni
(modified after loading)
Sample Info : CP_211_ridotto, 1 mL/min, 98:2 hex:ipr, 25°C, IC

Additional Info : Peak(s) manually integrated

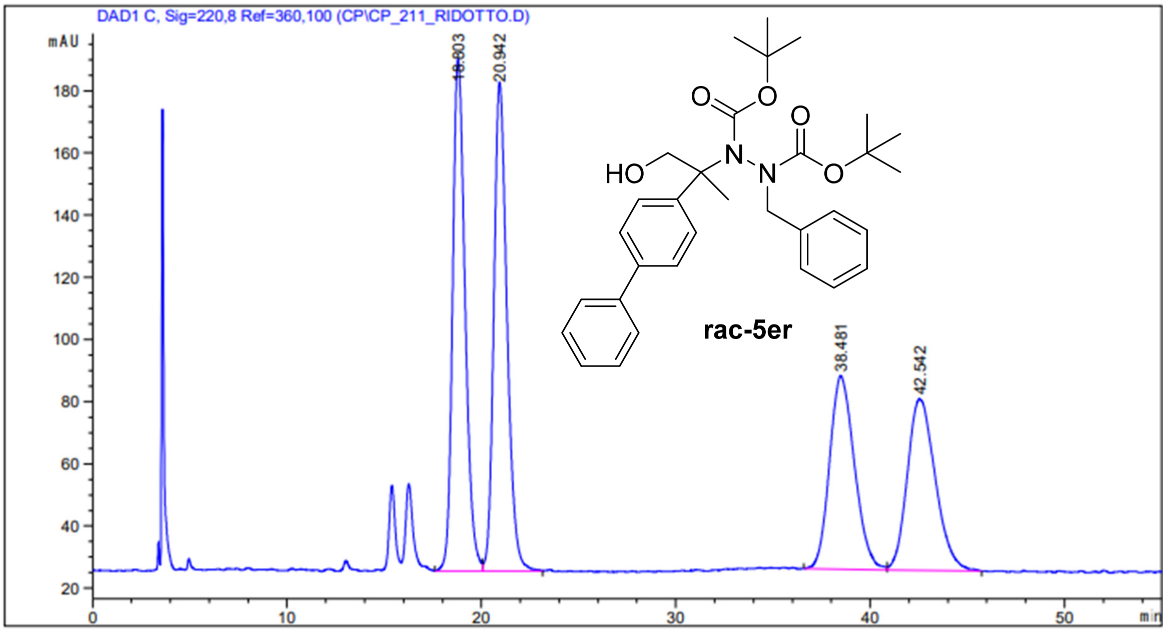

===
Area Percent Report
===

Sorted By : Signal
Multiplier: : 1.0000
Dilution: : 1.0000
Use Multiplier & Dilution Factor with ISTDs

Signal 1: DAD1 C, Sig=220,8 Ref=360,100

Peak #	RetTime [min]	Type	Width [min]	Area [mAU*s]	Height [mAU]	Area %
1	18.803	BV	0.6847	7347.02734	164.61923	28.4906
2	20.942	VB	0.7152	7375.02051	157.24989	28.5992
3	38.481	BV	1.3939	5540.93408	62.28122	21.4869
4	42.542	VB	1.4545	5524.55664	55.06638	21.4234

Totals : 2.57875e4 439.21672

)ata File H:\HPLC\1\DATA\CP\CP_255_R_2_II.D
;ample Name: CP_255_R_2_II

===
Acq. Operator : Chiara
Acq. Instrument : HPLC-1 Location : Vial 1
Injection Date : 21/04/2022 18:01:06
Acq. Method : C:\CHEM32\1\METHODS\DEF_LC.M
Last changed : 21/04/2022 17:53:32 by Chiara
(modified after loading)
Analysis Method : H:\HPLC\2\METHODS\DEF_LC.M
Last changed : 06/06/2022 10:36:40
(modified after loading)
Sample Info : CP_255_R_2_II, pulito, 1 mL/min, 98:2 hex:ipr, 25°C, IC

Additional Info : Peak(s) manually integrated

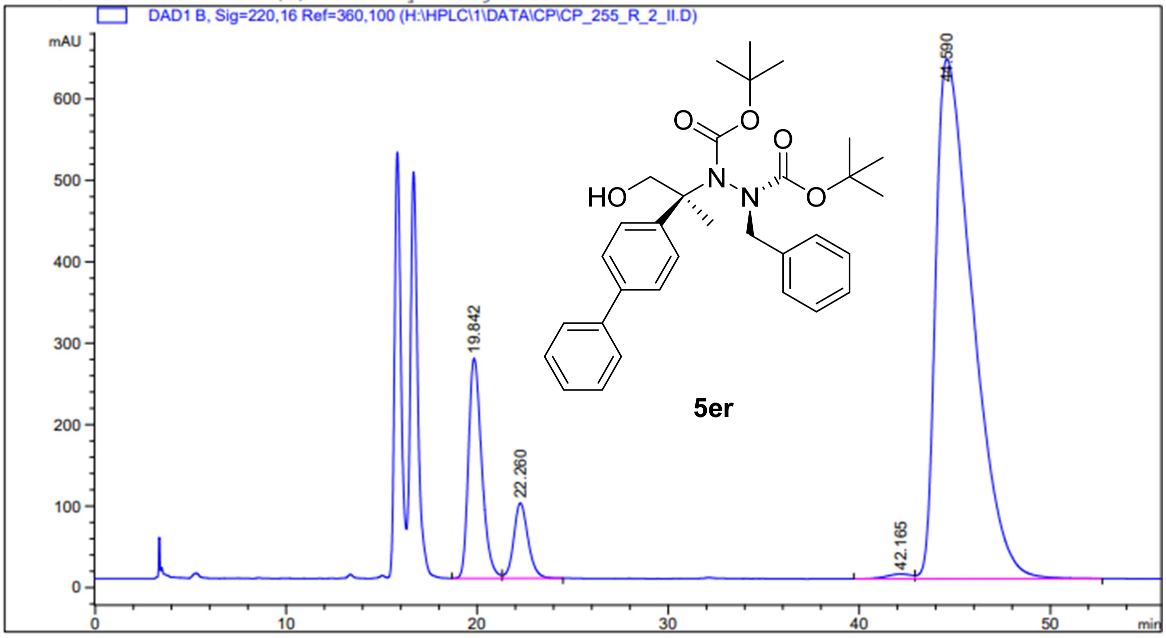

===
Area Percent Report
===

Sorted By : Signal
Multiplier: : 1.0000
Dilution: : 1.0000
Use Multiplier & Dilution Factor with ISTDs

Signal 1: DAD1 B, Sig=220,16 Ref=360,100

Peak #	RetTime [min]	Type	Width [min]	Area [mAU*s]	Height [mAU]	Area %
1	19.842	BV	0.7354	1.30479e4	270.18030	12.5910
2	22.260	VB	0.7955	4814.13281	92.45843	4.6456
3	42.165	BV	1.1949	513.15485	5.80731	0.4952
4	44.590	VB	1.9701	8.52537e4	638.37128	82.2683

Totals : 1.03629e5 1006.81731

Data File H:\HPLC\1\DATA\CP\CP_210_RIDOTTO.D
Sample Name: CP_210_ridotto

===

Acq. Operator : Chiara
Acq. Instrument : HPLC-1 Location : Vial 1
Injection Date : 03/02/2022 10:57:51
Acq. Method : C:\CHEM32\1\METHODS\DEF_LC.M
Last changed : 03/02/2022 10:17:08 by Chiara
(modified after loading)
Analysis Method : H:\HPLC\2\METHODS\DEF_LC.M
Last changed : 24/05/2022 10:41:02
(modified after loading)
Sample Info : CP_210_ridotto, 1 mL/min, 98:2 hex:ipr, 25°C, IC

Additional Info : Peak(s) manually integrated

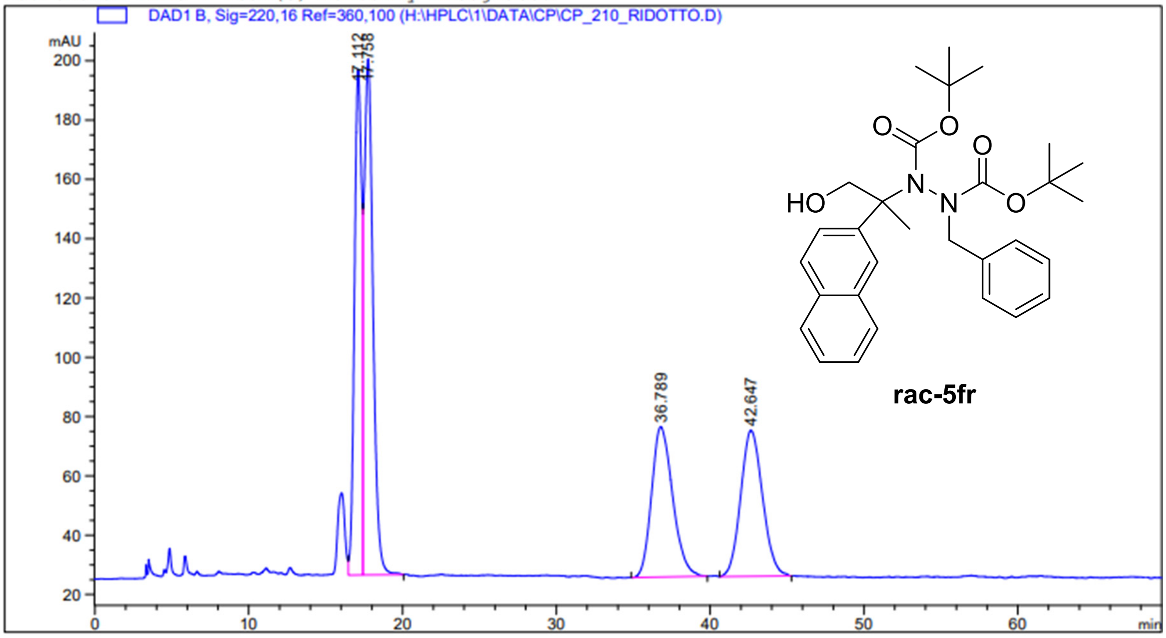

===
Area Percent Report
===

Sorted By : Signal
Multiplier: : 1.0000
Dilution: : 1.0000
Use Multiplier & Dilution Factor with ISTDs

Signal 1: DAD1 B, Sig=220,16 Ref=360,100

Peak #	RetTime [min]	Type	Width [min]	Area [mAU*s]	Height [mAU]	Area %
1	17.112	VV	0.4985	5589.28711	170.49522	25.1286
2	17.758	VB	0.5896	6943.39697	173.99034	31.2165
3	36.789	BB	1.4424	4845.10547	50.72123	21.7829
4	42.647	BB	1.5256	4864.94385	49.20243	21.8721

Totals : 2.22427e4 444.40923

Data File C:\CHEM32\1\DATA\CP\CP_220_R.D
Sample Name: CP_220_R

===

Acq. Operator : Chiara
Acq. Instrument : HPLC-1 Location : Vial 1
Injection Date : 15/02/2022 16:35:29
Acq. Method : C:\CHEM32\1\METHODS\DEF_LC.M
Last changed : 15/02/2022 16:07:22 by Giovanni
(modified after loading)
Analysis Method : C:\CHEM32\1\METHODS\DEF_LC.M
Last changed : 18/05/2022 17:20:25 by Giovanni
(modified after loading)
Sample Info : CP_220_R, 1 mL/min, 98:2 hex:ipr, 25°C, IC

Additional Info : Peak(s) manually integrated

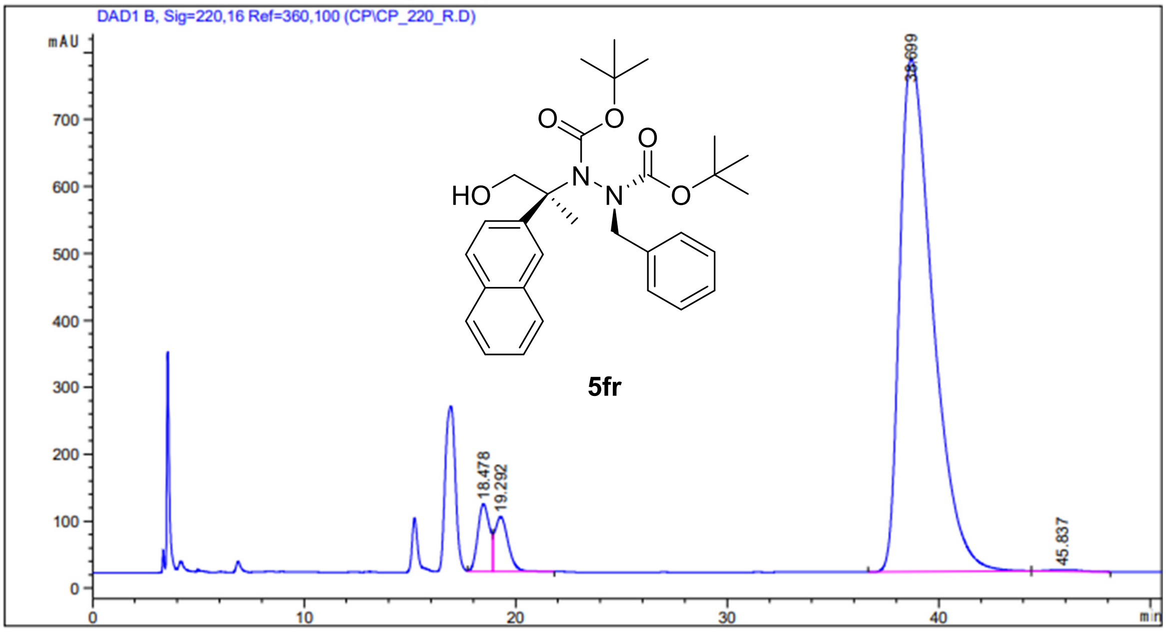

===
Area Percent Report
===

Sorted By : Signal
Multiplier: : 1.0000
Dilution: : 1.0000
Use Multiplier & Dilution Factor with ISTDs

Signal 1: DAD1 B, Sig=220,16 Ref=360,100

Peak #	RetTime [min]	Type	Width [min]	Area [mAU*s]	Height [mAU]	Area %
1	18.478	VV	0.5934	3920.14453	100.87508	4.3057
2	19.292	VB	0.6568	3568.94238	81.83964	3.9200
3	38.699	BB	1.6134	8.33232e4	765.81567	91.5181
4	45.837	BB	1.2445	233.29845	2.29790	0.2562

Totals : 9.10456e4 950.82829

Data File C:\CHEM32\1\DATA\CP\CP_135_2.D
Sample Name: CP_135_2

===

Acq. Operator : Chiara
Acq. Instrument : HPLC-1 Location : Vial 1
Injection Date : 01/03/2022 14:06:01
Acq. Method : C:\CHEM32\1\METHODS\DEF_LC.M
Last changed : 01/03/2022 13:50:56 by Chiara
(modified after loading)
Analysis Method : C:\CHEM32\1\METHODS\DEF_LC.M
Last changed : 22/04/2022 17:50:00 by Chiara
(modified after loading)
Sample Info : CP_135_2, 1 mL/min, 98:2 hex:ipr, 25°C, IC

Additional Info : Peak(s) manually integrated

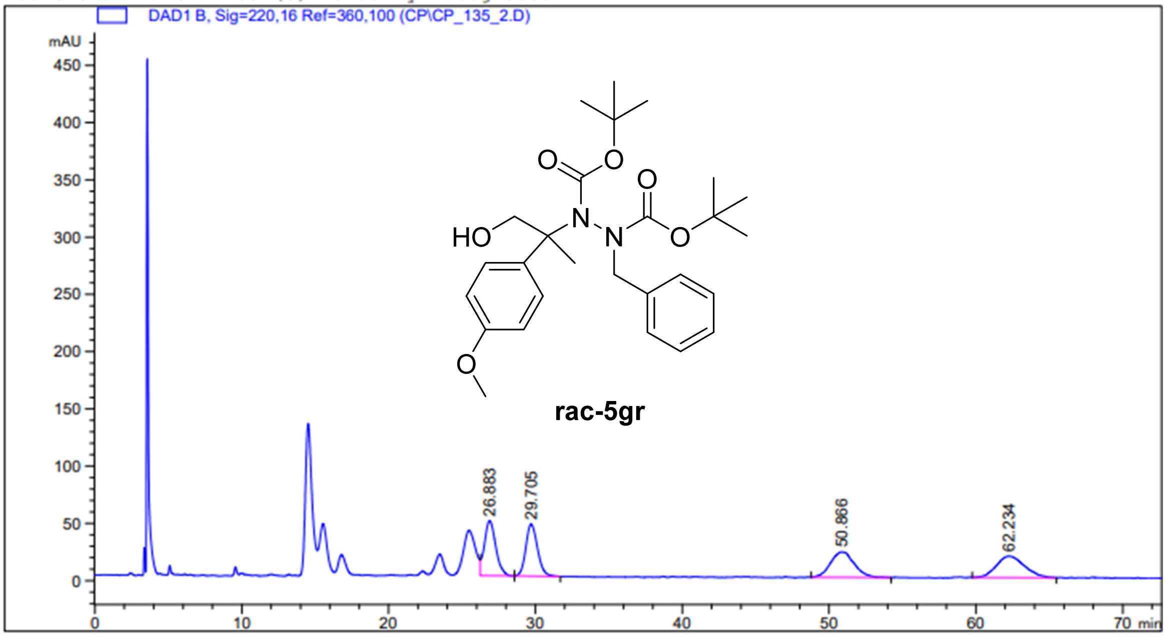

===

Area Percent Report

===

Sorted By : Signal
Multiplier: : 1.0000
Dilution: : 1.0000
Use Multiplier & Dilution Factor with ISTDs

Signal 1: DAD1 B, Sig=220,16 Ref=360,100

Peak #	RetTime [min]	Type	Width [min]	Area [mAU*s]	Height [mAU]	Area %
1	26.883	VB	0.8266	2596.38647	48.03494	25.6748
2	29.705	BB	0.8649	2526.40649	45.41959	24.9828
3	50.866	BB	1.5147	2495.89844	22.21669	24.6811
4	62.234	BB	1.8403	2493.91187	18.64765	24.6614

Totals : 1.01126e4 134.31888

```
Data File C:\CHEM32\1\DATA\CP\CP_227_R_2.D
Sample Name: CP_227_R_2

    =====================================================================
    Acq. Operator   : Chiara
    Acq. Instrument : HPLC-1                          Location : Vial 1
    Injection Date  : 02/03/2022 16:25:59
    Acq. Method     : C:\CHEM32\1\METHODS\DEF_LC.M
    Last changed    : 02/03/2022 16:23:12 by Chiara
                      (modified after loading)
    Analysis Method : C:\CHEM32\1\METHODS\DEF_LC.M
    Last changed    : 22/04/2022 17:52:27 by Chiara
                      (modified after loading)
    Sample Info     : CP_227_R_2, 1 mL/min, 98:2 hex:ipr, 25°C, IC

    Additional Info : Peak(s) manually integrated
```

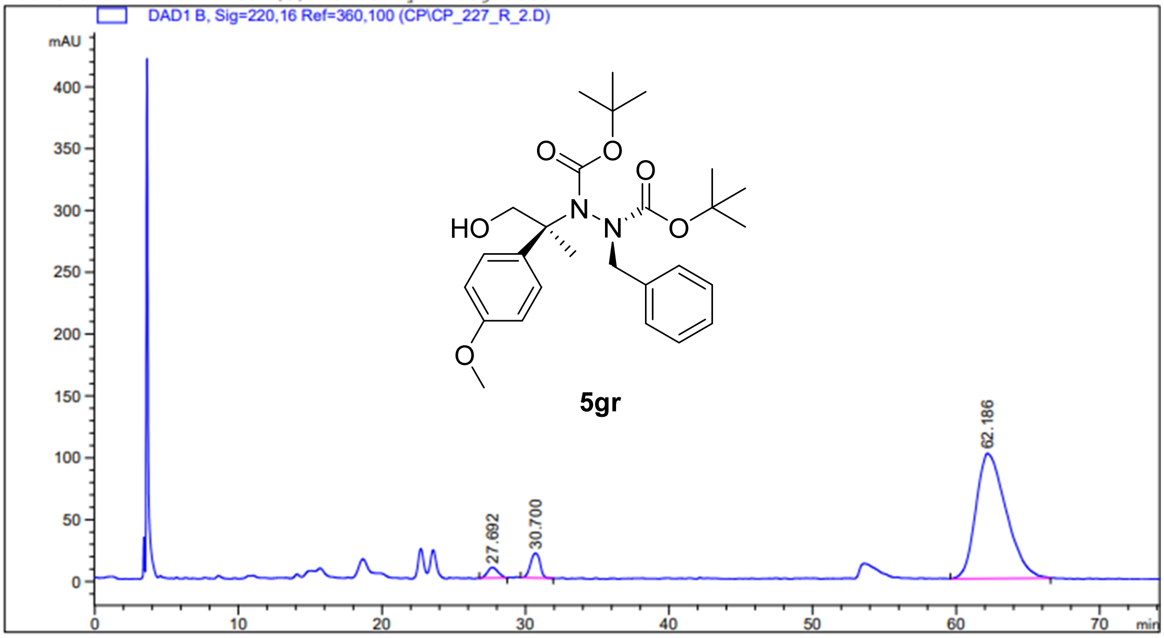


```
    =====================================================================
                           Area Percent Report
    =====================================================================

    Sorted By             :      Signal
    Multiplier:                  :      1.0000
    Dilution:                    :      1.0000
    Use Multiplier & Dilution Factor with ISTDs


    Signal 1: DAD1 B, Sig=220,16 Ref=360,100
```

Peak #	RetTime [min]	Type	Width [min]	Area [mAU*s]	Height [mAU]	Area %
1	27.692	BB	0.7806	443.52988	8.61513	2.7364
2	30.700	BB	0.7476	930.21252	20.11050	5.7391
3	62.186	BB	1.9470	1.48345e4	101.14850	91.5244

Totals : 1.62083e4   129.87413

```
Data File C:\CHEM32\1\DATA\CP\CP_212_RIDOTTO.D
Sample Name: CP_212_ridotto

=====================================================================
Acq. Operator   : Chiara
Acq. Instrument : HPLC-1                          Location : Vial 1
Injection Date  : 02/02/2022 16:51:27
Acq. Method     : C:\CHEM32\1\METHODS\DEF_LC.M
Last changed    : 02/02/2022 16:50:53 by Chiara
                  (modified after loading)
Analysis Method : C:\CHEM32\1\METHODS\DEF_LC.M
Last changed    : 26/04/2022 12:59:23 by Chiara
                  (modified after loading)
Sample Info     : CP_212_ridotto, 1 mL/min, 98:2 hex:ipr, 25°C, IC


Additional Info : Peak(s) manually integrated
```

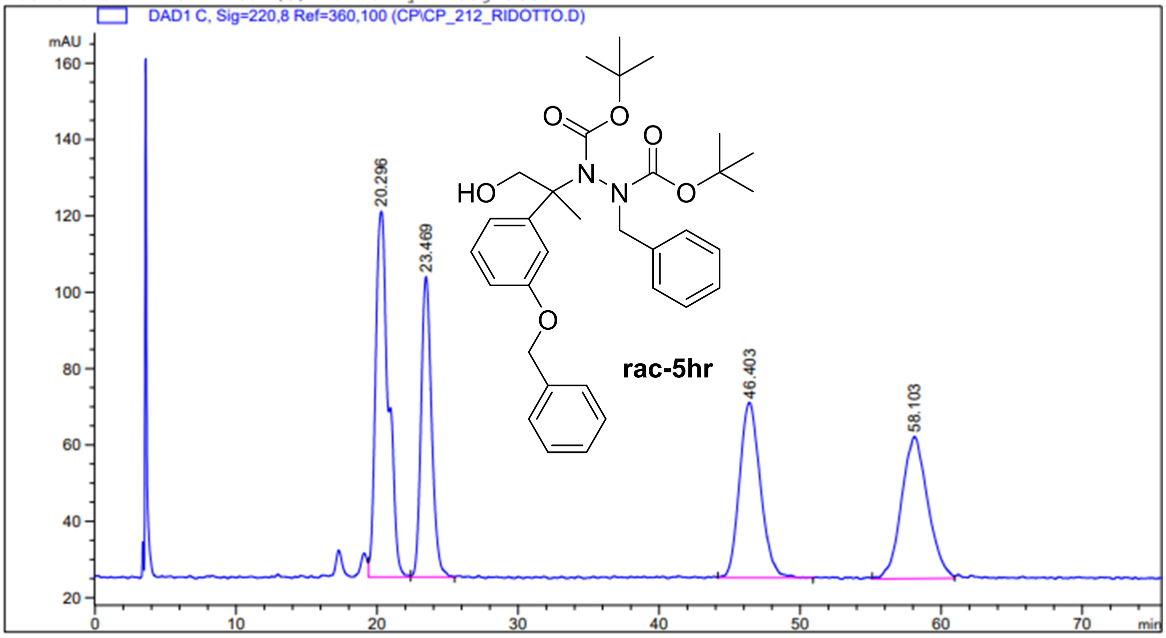


```
=====================================================================
                         Area Percent Report
=====================================================================

Sorted By             :      Signal
Multiplier:                    :       1.0000
Dilution:                      :       1.0000
Use Multiplier & Dilution Factor with ISTDs


Signal 1: DAD1 C, Sig=220,8 Ref=360,100
```

Peak #	RetTime [min]	Type	Width [min]	Area [mAU*s]	Height [mAU]	Area %
1	20.296	VV	0.9441	6043.45605	95.78014	30.7125
2	23.469	VV	0.7975	4082.83569	78.66629	20.7488
3	46.403	BB	1.5139	4733.56543	45.94631	24.0557
4	58.103	VV	1.6734	4817.63721	37.13958	24.4830

Totals : 1.96775e4 257.53232

```
Data File C:\CHEM32\1\DATA\CP\CP_222_R.D
Sample Name: CP_222_R

    =====================================================================
    Acq. Operator   : Chiara
    Acq. Instrument : HPLC-1                         Location : Vial 1
    Injection Date  : 22/02/2022 15:02:32
    Acq. Method     : C:\CHEM32\1\METHODS\DEF_LC.M
    Last changed    : 22/02/2022 13:59:28 by Chiara
                      (modified after loading)
    Analysis Method : C:\CHEM32\1\METHODS\DEF_LC.M
    Last changed    : 26/04/2022 13:00:16 by Chiara
                      (modified after loading)
    Sample Info     : CP_222_R, 1 mL/min, 90:10 hex:ipr, 25°C, IC

    Additional Info : Peak(s) manually integrated
```

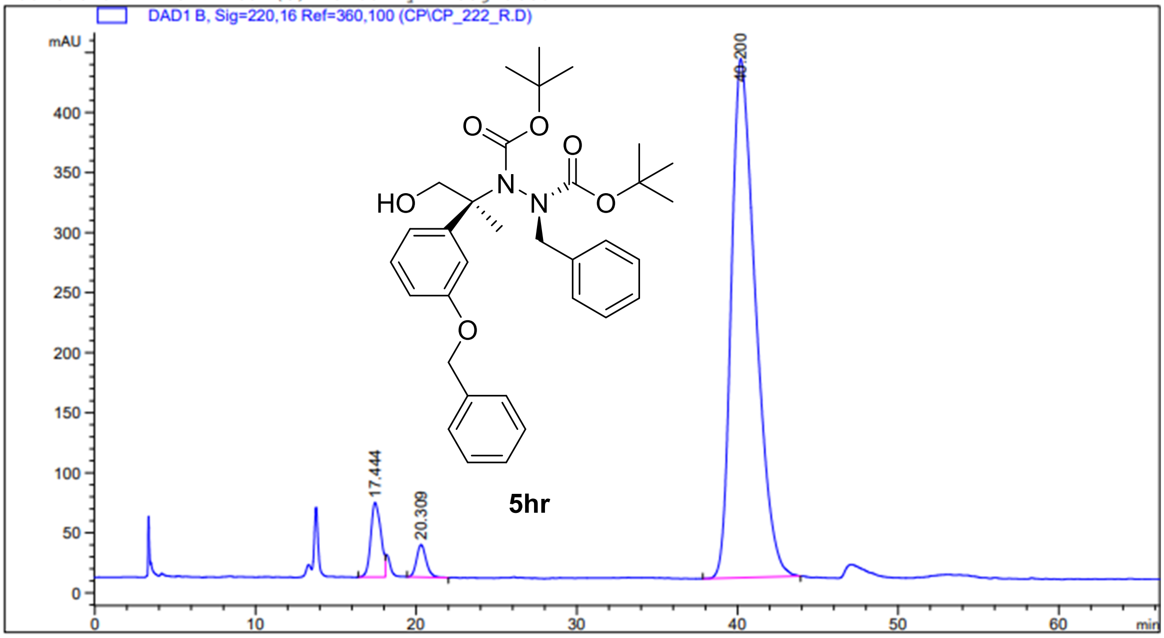


```
    =====================================================================
                             Area Percent Report
    =====================================================================

    Sorted By             :      Signal
    Multiplier:                   :      1.0000
    Dilution:                     :      1.0000
    Use Multiplier & Dilution Factor with ISTDs


    Signal 1: DAD1 B, Sig=220,16 Ref=360,100

    Peak RetTime Type  Width     Area      Height     Area
      #   [min]        [min]   [mAU*s]     [mAU]        %
    ----|-------|----|-------|----------|----------|--------|
       1  17.444 BV    0.7011 2697.91357   62.06453   5.5046
       2  20.309 BB    0.6287 1121.28235   27.10343   2.2878
       3  40.200 BB    1.5004 4.51929e4   432.02646  92.2076

    Totals :                  4.90121e4   521.19442
```

Data File C:\CHEM32\1\DATA\CP\CP_194_3.D
Sample Name: CP_194_3

===

Acq. Operator : Chiara
Acq. Instrument : HPLC-1 Location : Vial 1
Injection Date : 15/12/2021 12:33:24
Acq. Method : C:\CHEM32\1\METHODS\DEF_LC.M
Last changed : 15/12/2021 12:32:01 by Chiara
(modified after loading)
Analysis Method : C:\CHEM32\1\METHODS\DEF_LC.M
Last changed : 03/05/2022 17:55:16 by Chiara
(modified after loading)
Sample Info : CP_194_3, 0.5 mL/min, 98:2 hex:ipr, 25°C, IC

Additional Info : Peak(s) manually integrated

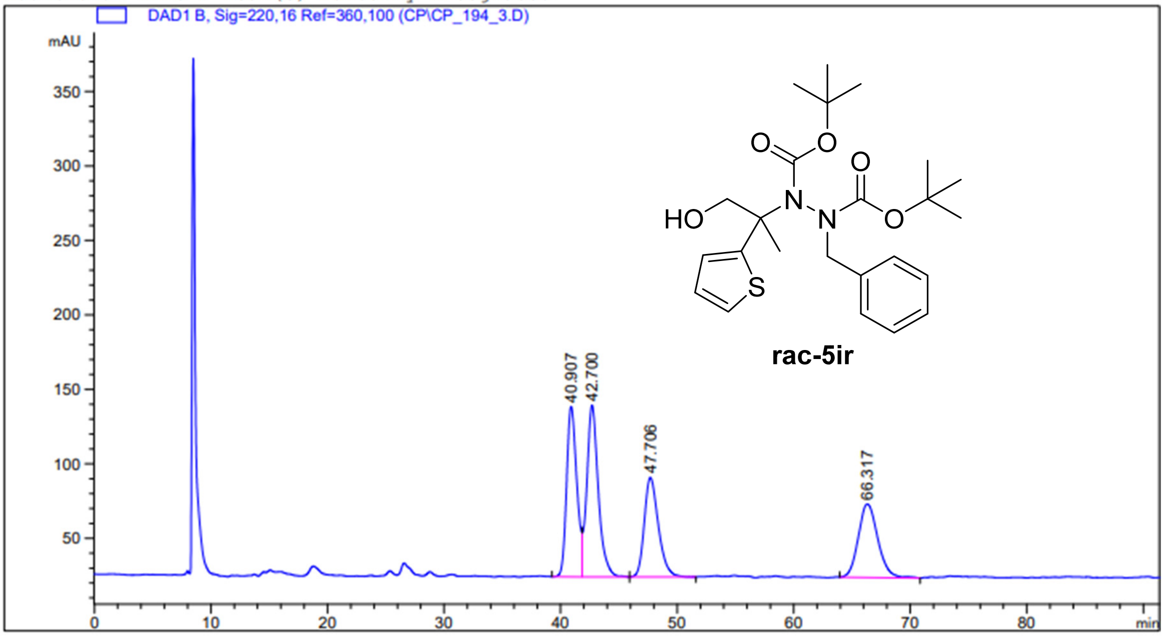

===
Area Percent Report
===

Sorted By : Signal
Multiplier: : 1.0000
Dilution: : 1.0000
Use Multiplier & Dilution Factor with ISTDs

Signal 1: DAD1 B, Sig=220,16 Ref=360,100

Peak #	RetTime [min]	Type	Width [min]	Area [mAU*s]	Height [mAU]	Area %
1	40.907	BV	0.9332	6948.21289	114.29614	26.4888
2	42.700	VB	1.0279	7934.87500	115.16032	30.2502
3	47.706	BB	1.2773	5610.95996	66.70800	21.3907
4	66.317	BB	1.7501	5736.73877	49.28159	21.8703

Totals : 2.62308e4 345.44606

Data File C:\CHEM32\1\DATA\CP\CP_223_R_2.D
Sample Name: CP_223_R_2

===

Acq. Operator : Chiara
Acq. Instrument : HPLC-1 Location : Vial 1
Injection Date : 23/02/2022 11:21:53
Acq. Method : C:\CHEM32\1\METHODS\DEF_LC.M
Last changed : 23/02/2022 11:20:57 by Chiara
(modified after loading)
Analysis Method : C:\CHEM32\1\METHODS\DEF_LC.M
Last changed : 03/05/2022 17:57:02 by Chiara
(modified after loading)
Sample Info : CP_223_R_2, 0.5 mL/min, 98:2 hex:ipr, 25°C, IC

Additional Info : Peak(s) manually integrated

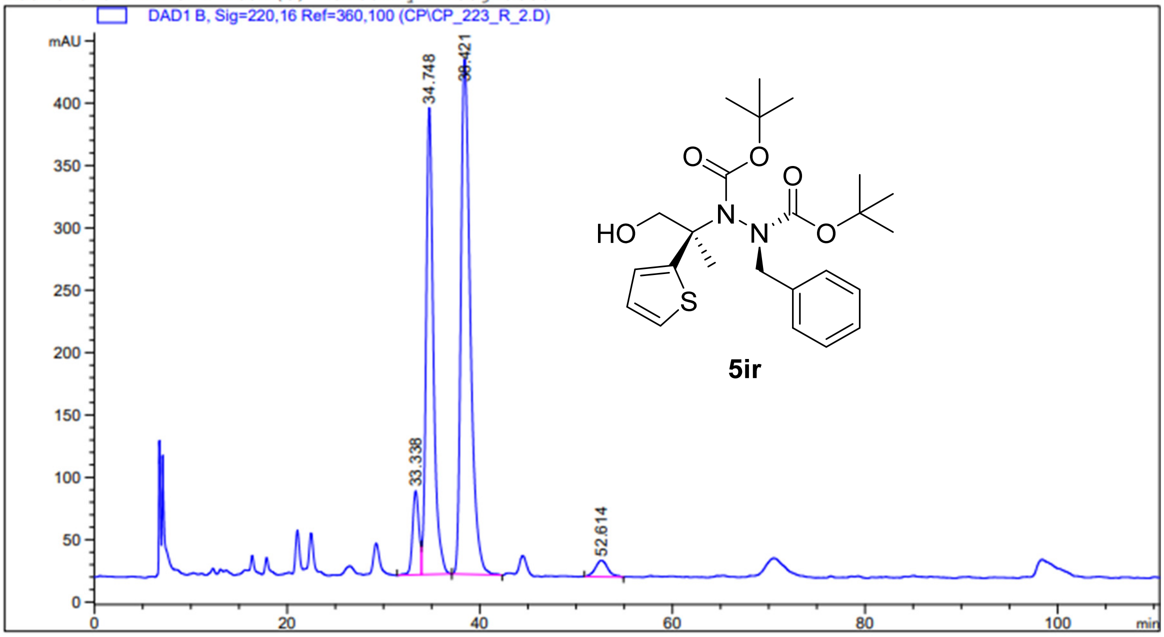

5ir

===

Area Percent Report

===

Sorted By : Signal
Multiplier: : 1.0000
Dilution: : 1.0000
Use Multiplier & Dilution Factor with ISTDs

Signal 1: DAD1 B, Sig=220,16 Ref=360,100

Peak #	RetTime [min]	Type	Width [min]	Area [mAU*s]	Height [mAU]	Area %
1	33.338	BV	0.6965	3097.83911	67.37090	5.9775
2	34.748	VB	0.7969	1.96592e4	374.21191	37.9336
3	38.421	BB	1.0200	2.78935e4	412.97580	53.8222
4	52.614	BB	1.4086	1174.72314	13.06996	2.2667

Totals : 5.18252e4 867.62857

Data File C:\CHEM32\1\DATA\CP\CP_180_R_LUXC.D
Sample Name: CP_180_R_LuxC

===

Acq. Operator : Chiara
Acq. Instrument : HPLC-1 Location : Vial 1
Injection Date : 09/03/2022 13:01:50
Acq. Method : C:\CHEM32\1\METHODS\DEF_LC.M
Last changed : 09/03/2022 12:53:56 by Chiara
(modified after loading)
Analysis Method : C:\CHEM32\1\METHODS\DEF_LC.M
Last changed : 18/05/2022 16:46:26 by Giovanni
(modified after loading)
Sample Info : CP_180_R_LuxC, 1 mL/min, 95:5 hex:ipr, 25°C, lux 5u cellulose-2

Additional Info : Peak(s) manually integrated

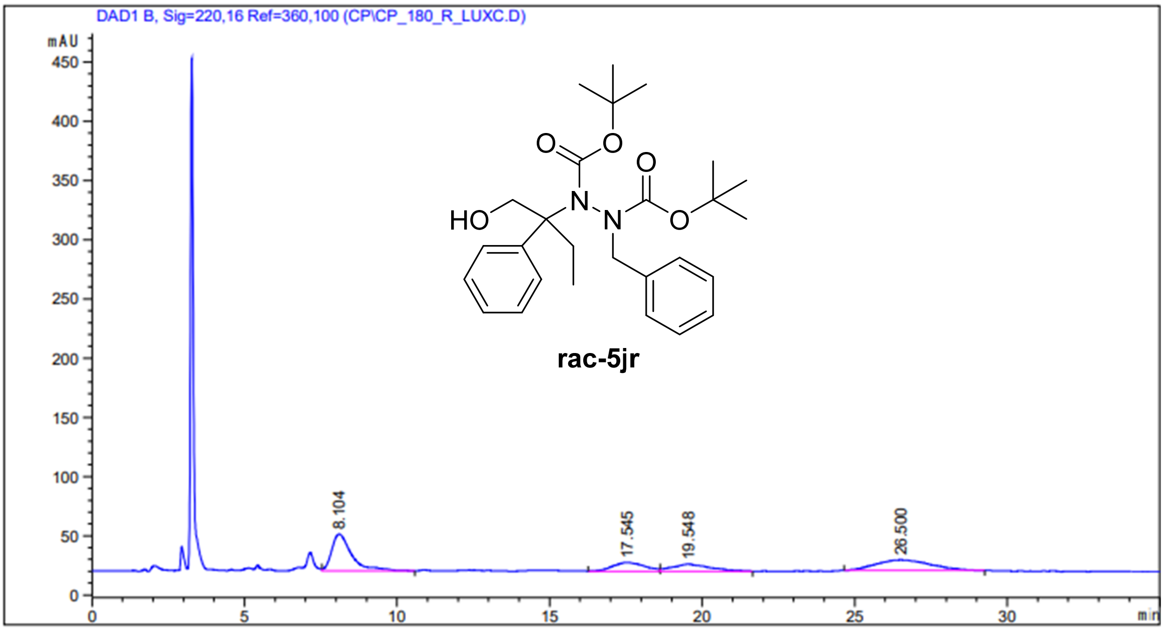

===
Area Percent Report
===

Sorted By : Signal
Multiplier: : 1.0000
Dilution: : 1.0000
Use Multiplier & Dilution Factor with ISTDs

Signal 1: DAD1 B, Sig=220,16 Ref=360,100

Peak #	RetTime [min]	Type	Width [min]	Area [mAU*s]	Height [mAU]	Area %
1	8.104	VB	0.6820	1425.58569	31.03194	39.5513
2	17.545	BV	0.9742	526.32788	7.25633	14.6024
3	19.548	VB	1.1138	504.38864	5.89765	13.9937
4	26.500	BB	1.5416	1148.09827	9.01617	31.8527

Totals : 3604.40048 53.20209

```
Data File C:\CHEM32\1\DATA\CP\CP_239R_2_LUXC.D
Sample Name: CP_239R_2_LuxC

=====================================================================
Acq. Operator   : Chiara
Acq. Instrument : HPLC-1                          Location : Vial 1
Injection Date  : 04/05/2022 15:28:50
Acq. Method     : C:\CHEM32\1\METHODS\DEF_LC.M
Last changed    : 04/05/2022 15:02:10 by Giovanni
                  (modified after loading)
Analysis Method : C:\CHEM32\1\METHODS\DEF_LC.M
Last changed    : 18/05/2022 16:53:41 by Giovanni
                  (modified after loading)
Sample Info     : CP_239R_2_LuxC, 1 mL/min, 95:5 hex:ipr, 25°C, LuxC

Additional Info : Peak(s) manually integrated
```

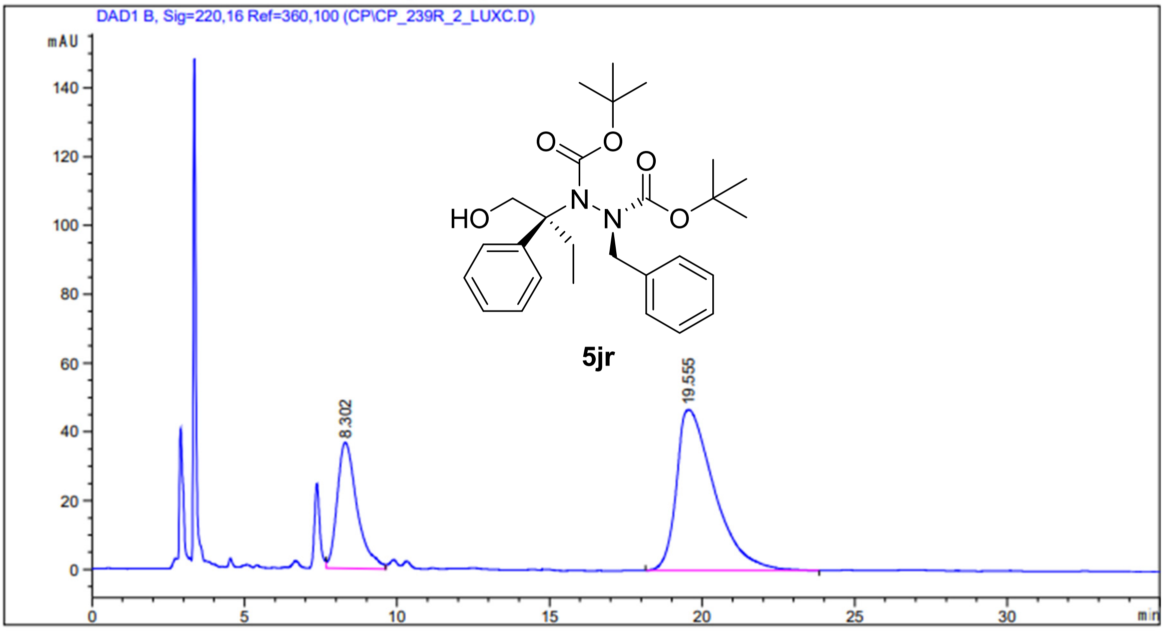


```
=====================================================================
                         Area Percent Report
=====================================================================

Sorted By             :      Signal
Multiplier:                  :      1.0000
Dilution:                    :      1.0000
Use Multiplier & Dilution Factor with ISTDs


Signal 1: DAD1 B, Sig=220,16 Ref=360,100

Peak RetTime Type  Width     Area      Height     Area
  #   [min]        [min]   [mAU*s]     [mAU]        %
----|-------|----|-------|----------|----------|--------|
   1   8.302 VV    0.6859 1657.85583   36.63976  29.0649
   2  19.555 BB    1.2258 4046.11426   46.74608  70.9351

Totals :                  5703.97009   83.38585
```

Data File H:\HPLC\1\DATA\GC\GC_366.D
Sample Name: GC_366

===

Acq. Operator : Giovanni
Acq. Instrument : HPLC-1 Location : Vial 1
Injection Date : 17/01/2022 10.41.51
Acq. Method : C:\CHEM32\1\METHODS\DEF_LC.M
Last changed : 17/01/2022 10.41.11 by Giovanni
(modified after loading)
Analysis Method : H:\HPLC\1\METHODS\DEF_LC.M
Last changed : 07/05/2022 14.10.49
(modified after loading)
Sample Info : GC_366, 1 mL/min, 98:2 hex:ipr, 25°C, IC

Additional Info : Peak(s) manually integrated

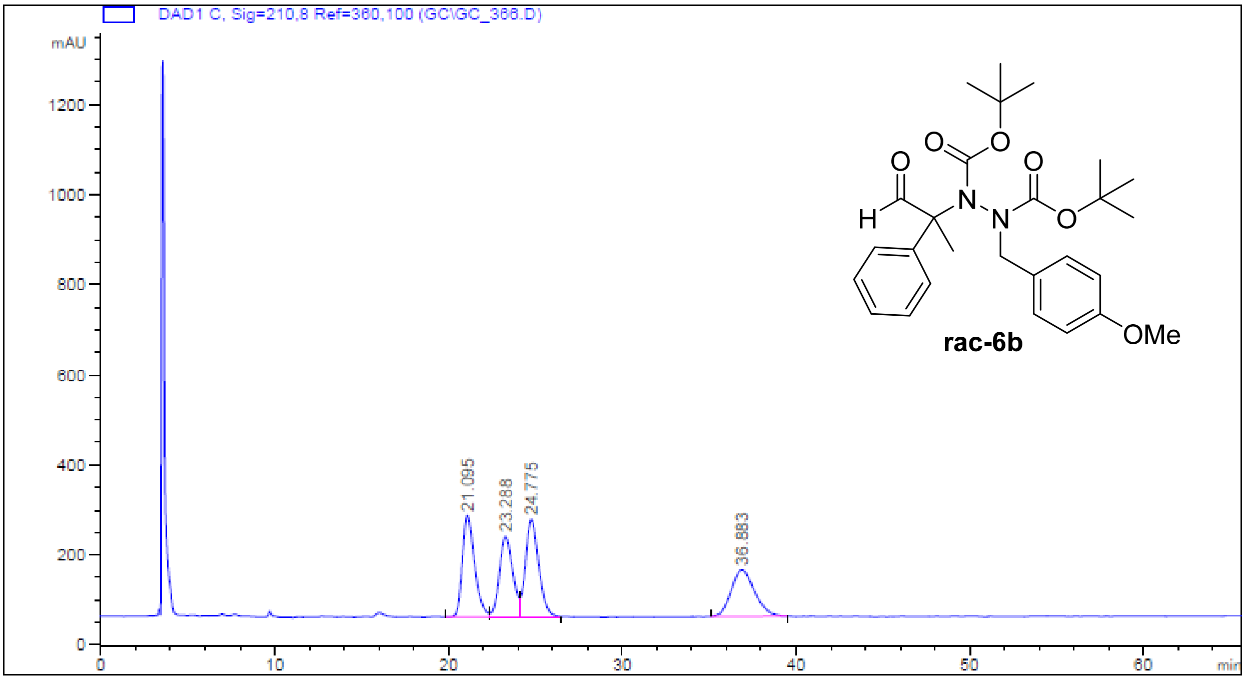

===
Area Percent Report
===

Sorted By : Signal
Multiplier: : 1.0000
Dilution: : 1.0000
Use Multiplier & Dilution Factor with ISTDs

Signal 1: DAD1 C, Sig=210,8 Ref=360,100

Peak #	RetTime [min]	Type	Width [min]	Area [mAU*s]	Height [mAU]	Area %
1	21.095	BV	0.7440	1.10458e4	225.27597	26.4823
2	23.288	VV	0.8205	9535.31738	178.14674	22.8610
3	24.775	VB	0.8049	1.14462e4	215.75931	27.4423
4	36.883	BB	1.3641	9682.70703	103.06392	23.2144

Totals : 4.17100e4 722.24594

Data File H:\HPLC\1\DATA\GC\GC_382.D
Sample Name: GC_382

===

Acq. Operator : Giovanni
Acq. Instrument : HPLC-1 Location : Vial 1
Injection Date : 18/02/2022 09.46.55
Acq. Method : C:\CHEM32\1\METHODS\DEF_LC.M
Last changed : 18/02/2022 09.29.14 by Giovanni
(modified after loading)
Analysis Method : H:\HPLC\1\METHODS\DEF_LC.M
Last changed : 07/05/2022 14.02.09
(modified after loading)
Sample Info : GC_382, 1 mL/min, 98:2 hex:ipr, 25°C, IC

Additional Info : Peak(s) manually integrated

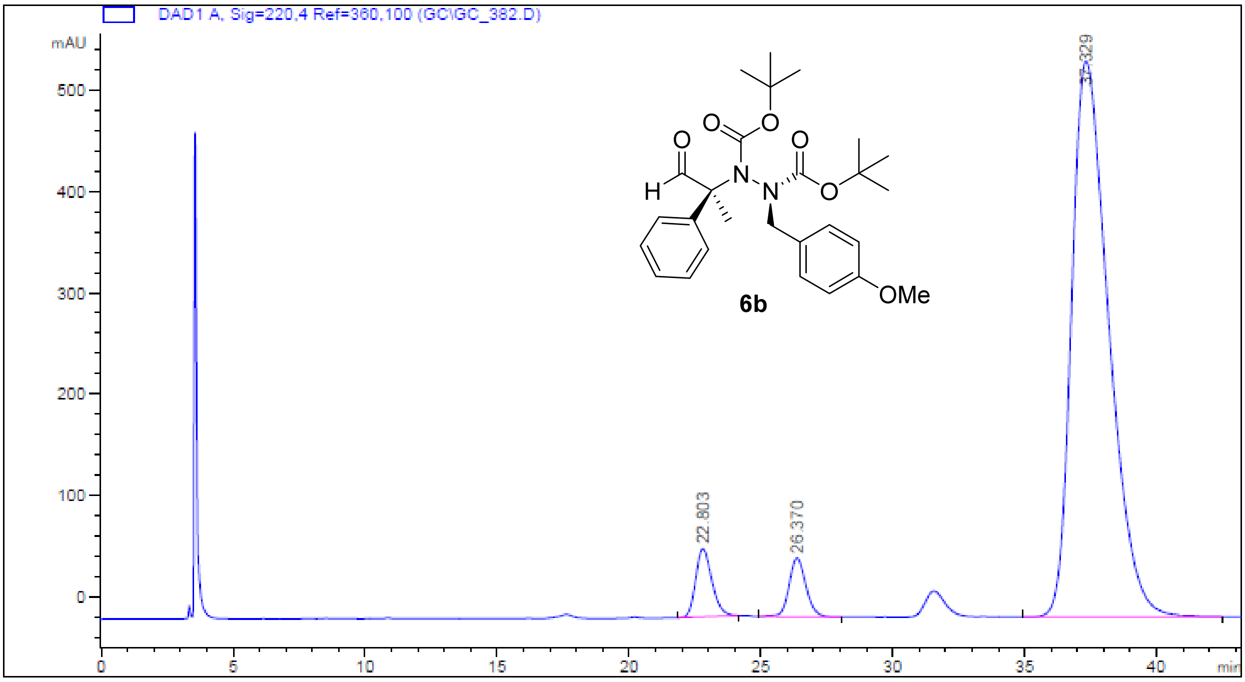

===
Area Percent Report
===

Sorted By : Signal
Multiplier: : 1.0000
Dilution: : 1.0000
Use Multiplier & Dilution Factor with ISTDs

Signal 1: DAD1 A, Sig=220,4 Ref=360,100

Peak #	RetTime [min]	Type	Width [min]	Area [mAU*s]	Height [mAU]	Area %
1	22.803	BB	0.6538	2857.00757	66.95844	4.8440
2	26.370	BB	0.6817	2584.41187	58.02159	4.3819
3	37.329	BB	1.4422	5.35383e4	548.79712	90.7741

Totals : 5.89797e4 673.77714

Data File C:\CHEM32\1\DATA\GC\GC_365_RID_1.D
Sample Name: GC_365_rid

===

Acq. Operator : Giovanni
Acq. Instrument : HPLC-1 Location : Vial 1
Injection Date : 21/02/2022 14:51:22
Acq. Method : C:\CHEM32\1\METHODS\DEF_LC.M
Last changed : 21/02/2022 14:46:01 by Giovanni
(modified after loading)
Analysis Method : C:\CHEM32\1\METHODS\DEF_LC.M
Last changed : 03/05/2022 10:40:53 by Chiara
(modified after loading)
Sample Info : GC_365_ridotto, 1 mL/min, 92:8 hex:ipr, 25°C, IC

Additional Info : Peak(s) manually integrated

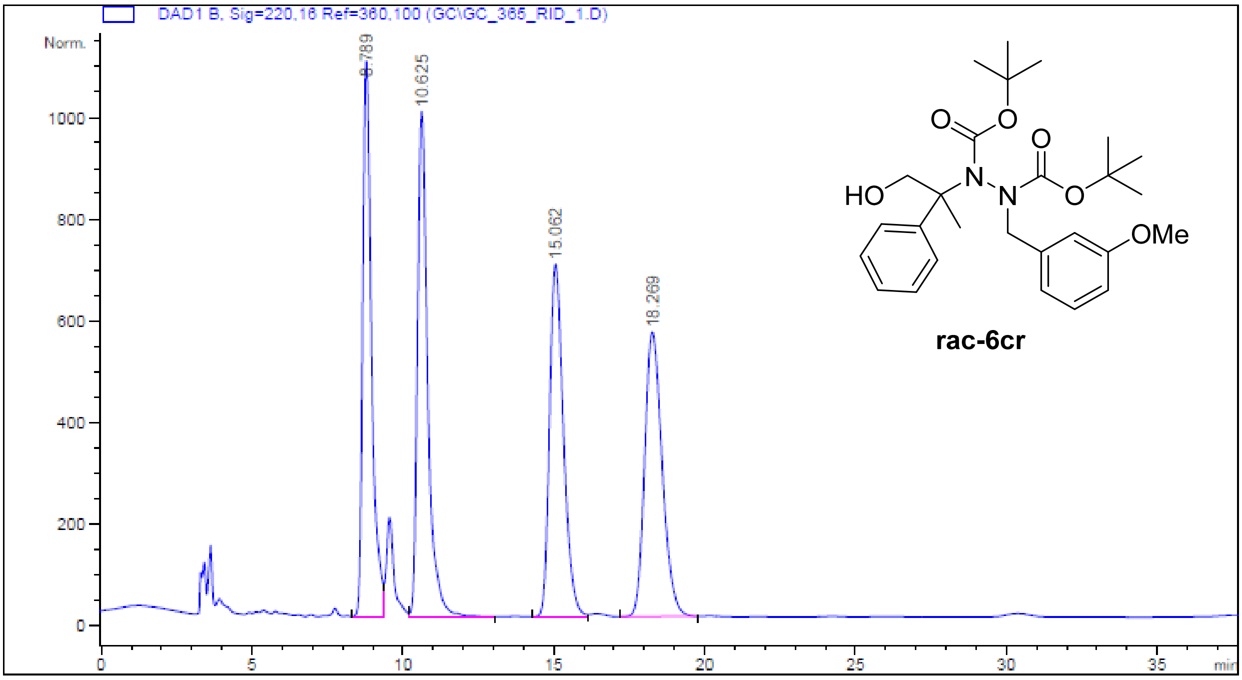

rac-6cr

===
Area Percent Report
===

Sorted By : Signal
Multiplier: : 1.0000
Dilution: : 1.0000
Use Multiplier & Dilution Factor with ISTDs

Signal 1: DAD1 B, Sig=220,16 Ref=360,100

Peak #	RetTime [min]	Type	Width [min]	Area [mAU*s]	Height [mAU]	Area %
1	8.789	BV	0.3183	2.23275e4	1093.24707	24.5720
2	10.625	VB	0.3571	2.36134e4	994.05261	25.9872
3	15.062	BV	0.4949	2.22911e4	693.72565	24.5319
4	18.269	BB	0.6238	2.26335e4	559.69019	24.9088

Totals : 9.08655e4 3340.71552

Data File C:\CHEM32\1\DATA\GC\GC_379_RID_1.D
Sample Name: GC_379_rid

===

Acq. Operator : Giovanni
Acq. Instrument : HPLC-1 Location : Vial 1
Injection Date : 21/02/2022 15:30:58
Acq. Method : C:\CHEM32\1\METHODS\DEF_LC.M
Last changed : 21/02/2022 15:29:27 by Giovanni
(modified after loading)
Analysis Method : C:\CHEM32\1\METHODS\DEF_LC.M
Last changed : 03/05/2022 10:41:39 by Chiara
(modified after loading)
Sample Info : GC_379_ridotto, 1 mL/min, 92:8 hex:ipr, 25°C, IC

Additional Info : Peak(s) manually integrated

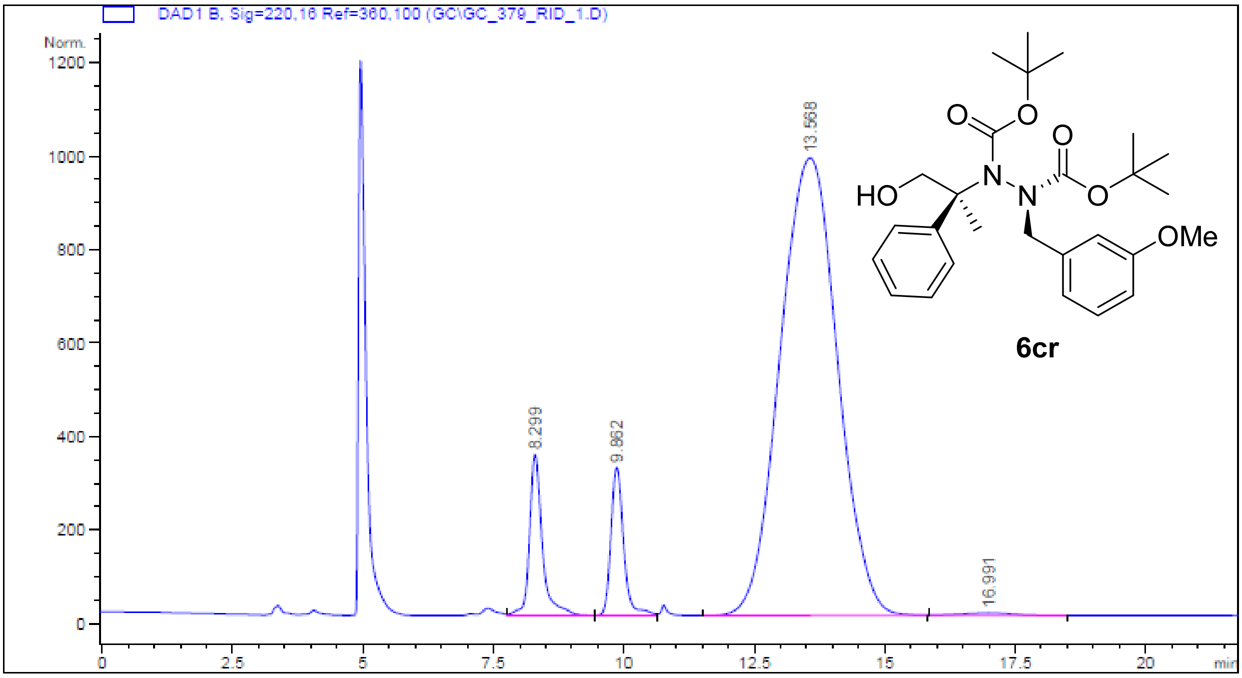

===

Area Percent Report

===

Sorted By : Signal
Multiplier: : 1.0000
Dilution: : 1.0000
Use Multiplier & Dilution Factor with ISTDs

Signal 1: DAD1 B, Sig=220,16 Ref=360,100

Peak #	RetTime [min]	Type	Width [min]	Area [mAU*s]	Height [mAU]	Area %
1	8.299	VB	0.2563	5916.02246	344.62469	6.8383
2	9.862	BV	0.2575	5356.50049	316.45337	6.1916
3	13.568	BB	1.2135	7.49478e4	978.63129	86.6318
4	16.991	BB	0.8300	292.72147	5.00762	0.3384

Totals : 8.65131e4 1644.71697

```
Data File C:\CHEM32\1\DATA\GC\GC_384_RID.D
Sample Name: GC_384_rid

    =====================================================================
    Acq. Operator   : Giovanni
    Acq. Instrument : HPLC-1                          Location : Vial 1
    Injection Date  : 18/02/2022 16:26:01
    Acq. Method     : C:\CHEM32\1\METHODS\DEF_LC.M
    Last changed    : 18/02/2022 16:15:56 by Chiara
                      (modified after loading)
    Analysis Method : C:\CHEM32\1\METHODS\DEF_LC.M
    Last changed    : 03/05/2022 10:37:18 by Chiara
                      (modified after loading)
    Sample Info     : GC_384_ridotto, 1 mL/min, 90:10 hex:ipr, 25°C, IC

    Additional Info : Peak(s) manually integrated
```

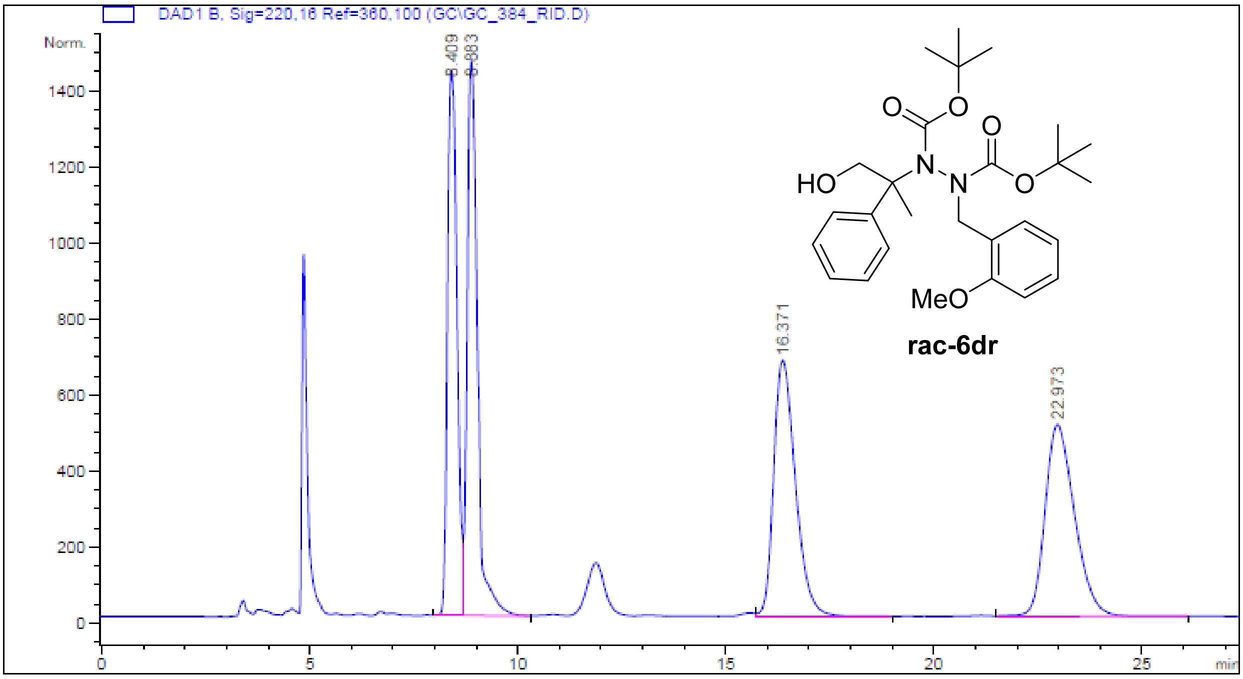


```
    =====================================================================
                             Area Percent Report
    =====================================================================

    Sorted By             :      Signal
    Multiplier:                  :      1.0000
    Dilution:                    :      1.0000
    Use Multiplier & Dilution Factor with ISTDs


    Signal 1: DAD1 B, Sig=220,16 Ref=360,100
```

Peak #	RetTime [min]	Type	Width [min]	Area [mAU*s]	Height [mAU]	Area %
1	8.409	BV	0.2541	2.28224e4	1430.49854	23.5122
2	8.883	VB	0.2632	2.48792e4	1457.47778	25.6311
3	16.371	VB	0.5598	2.44525e4	673.19305	25.1916
4	22.973	VB	0.7641	2.49122e4	504.47980	25.6651
Totals :				9.70664e4	4065.64917	

```
Data File H:\HPLC\1\DATA\GC\GC_420_RID.D
Sample Name: GC_420_RID

    =====================================================================
    Acq. Operator   : Giovanni
    Acq. Instrument : HPLC-1                          Location : Vial 1
    Injection Date  : 13/05/2022 11.28.39
    Acq. Method     : C:\CHEM32\1\METHODS\DEF_LC.M
    Last changed    : 13/05/2022 10.42.01 by Giovanni
                      (modified after loading)
    Analysis Method : H:\HPLC\1\METHODS\DEF_LC.M
    Last changed    : 21/05/2022 14.11.49
                      (modified after loading)
    Sample Info     : GC_420_RID,1 mL/min, 90:10 hex:ipr, 25°C, IC

    Additional Info : Peak(s) manually integrated
```

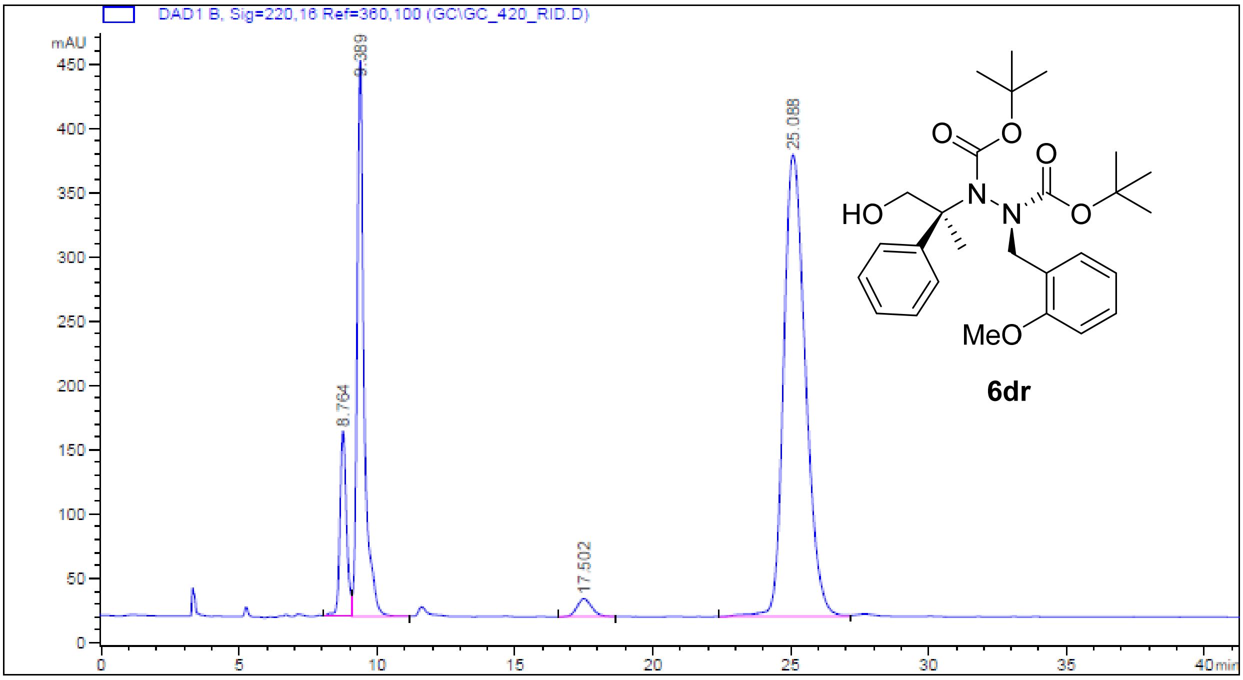

6dr

```
    =====================================================================
                             Area Percent Report
    =====================================================================

    Sorted By             :      Signal
    Multiplier:                  :      1.0000
    Dilution:                    :      1.0000
    Use Multiplier & Dilution Factor with ISTDs


    Signal 1: DAD1 B, Sig=220,16 Ref=360,100

    Peak RetTime Type  Width     Area      Height     Area
      #   [min]        [min]   [mAU*s]     [mAU]        %
    ----|-------|----|-------|----------|----------|--------|
       1   8.764 VV    0.2558 2416.99219  143.98268   8.0011
       2   9.389 VB    0.2570 7584.79053  431.60550  25.1084
       3  17.502 BB    0.5705  518.63452   13.92620   1.7169
       4  25.088 BV    0.8443 1.96877e4  358.70432  65.1736

    Totals :                  3.02082e4   948.21870
```

Data File C:\CHEM32\1\DATA\GC\GC_350.D
Sample Name: GC_350

===

Acq. Operator : Giovanni
Acq. Instrument : HPLC-1 Location : Vial 1
Injection Date : 15/12/2021 14:38:39
Acq. Method : C:\CHEM32\1\METHODS\DEF_LC.M
Last changed : 15/12/2021 14:37:53 by Giovanni
(modified after loading)
Analysis Method : C:\CHEM32\1\METHODS\DEF_LC.M
Last changed : 03/05/2022 10:54:58 by Chiara
(modified after loading)
Sample Info : GC_350, 1 mL/min, 98:2 hex:ipr, 25°C, IC

Additional Info : Peak(s) manually integrated

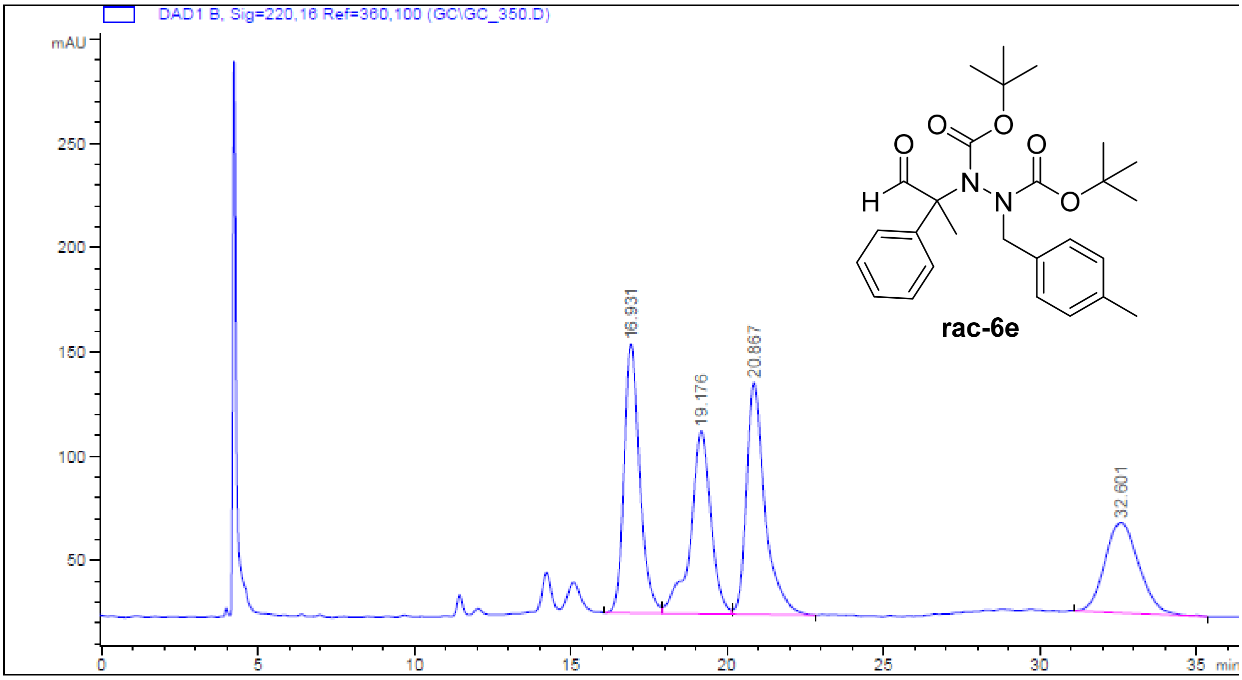

===

Area Percent Report

===

Sorted By : Signal
Multiplier: : 1.0000
Dilution: : 1.0000
Use Multiplier & Dilution Factor with ISTDs

Signal 1: DAD1 B, Sig=220,16 Ref=360,100

Peak #	RetTime [min]	Type	Width [min]	Area [mAU*s]	Height [mAU]	Area %
1	16.931	BV	0.5319	4504.48096	128.84485	27.5521
2	19.176	VV	0.6792	3986.21411	87.54908	24.3821
3	20.867	VB	0.6094	4584.28516	111.11101	28.0403
4	32.601	BB	1.1466	3273.95947	43.27497	20.0255

Totals : 1.63489e4 370.77990

Data File C:\CHEM32\1\DATA\GC\GC_393.D
Sample Name: GC_393

===

Acq. Operator : Giovanni
Acq. Instrument : HPLC-1 Location : Vial 1
Injection Date : 04/03/2022 16:29:46
Acq. Method : C:\CHEM32\1\METHODS\DEF_LC.M
Last changed : 04/03/2022 16:07:44 by Chiara
(modified after loading)
Analysis Method : C:\CHEM32\1\METHODS\DEF_LC.M
Last changed : 03/05/2022 10:55:58 by Chiara
(modified after loading)
Sample Info : GC_393, 1 mL/min, 98:2 hex:ipr, 25°C, IC

Additional Info : Peak(s) manually integrated

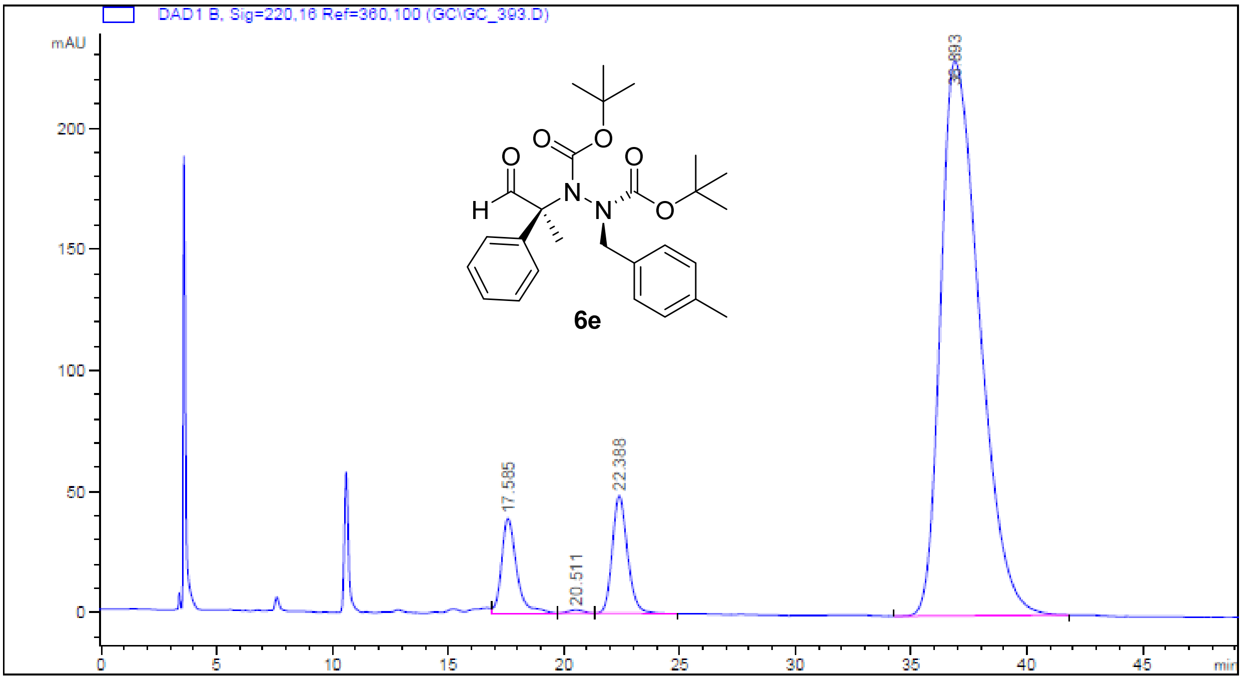

===

Area Percent Report

===

Sorted By : Signal
Multiplier: : 1.0000
Dilution: : 1.0000
Use Multiplier & Dilution Factor with ISTDs

Signal 1: DAD1 B, Sig=220,16 Ref=360,100

Peak #	RetTime [min]	Type	Width [min]	Area [mAU*s]	Height [mAU]	Area %
1	17.585	VB	0.6847	1772.01001	39.10073	5.7427
2	20.511	BB	0.5879	63.37709	1.40055	0.2054
3	22.388	BB	0.6957	2225.28589	48.64693	7.2117
4	36.893	BB	1.7723	2.67961e4	228.81345	86.8402

Totals : 3.08567e4 317.96165

Data File C:\CHEM32\1\DATA\GC\GC_370_RID_3.D
Sample Name: GC_370_RID

===

Acq. Operator : Giovanni
Acq. Instrument : HPLC-1 Location : Vial 1
Injection Date : 15/03/2022 15:06:48
Acq. Method : C:\CHEM32\1\METHODS\DEF_LC.M
Last changed : 15/03/2022 15:05:36 by Giovanni
(modified after loading)
Analysis Method : C:\CHEM32\1\METHODS\DEF_LC.M
Last changed : 03/05/2022 11:00:48 by Chiara
(modified after loading)
Sample Info : GC_370_RID, 1 mL/min, 95:5 hex:ipr, 25°C, IC

Additional Info : Peak(s) manually integrated

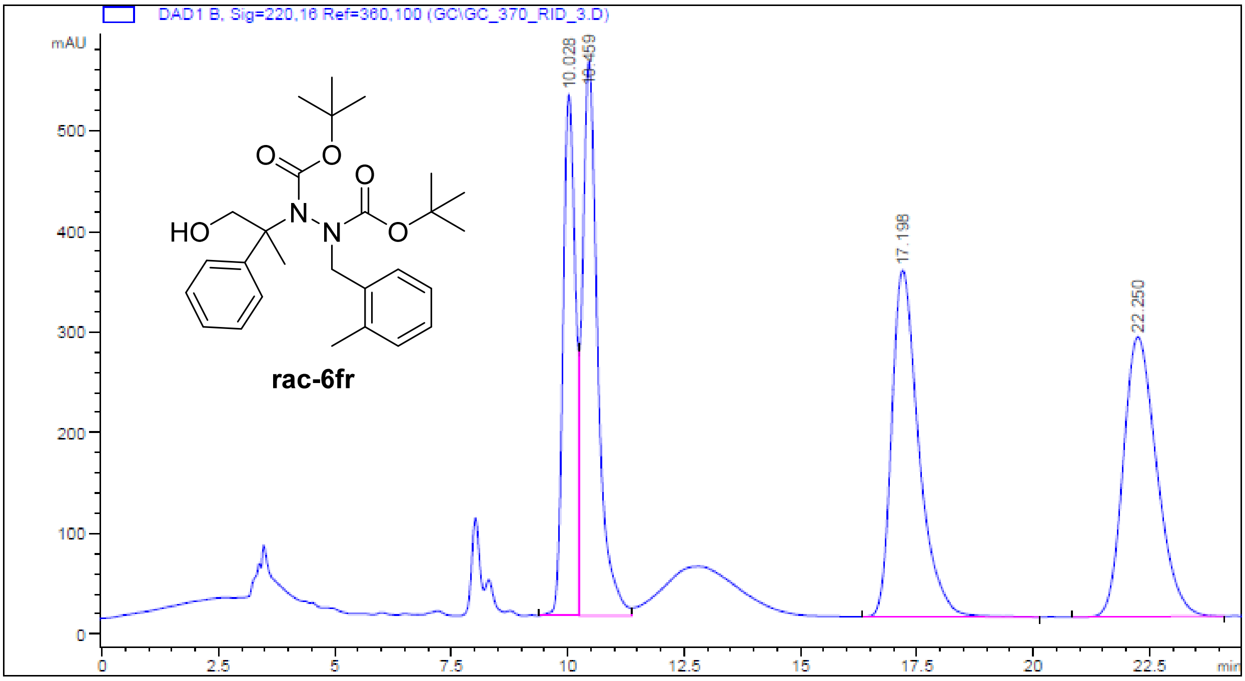

===
Area Percent Report
===

Sorted By : Signal
Multiplier: : 1.0000
Dilution: : 1.0000
Use Multiplier & Dilution Factor with ISTDs

Signal 1: DAD1 B, Sig=220,16 Ref=360,100

Peak #	RetTime [min]	Type	Width [min]	Area [mAU*s]	Height [mAU]	Area %
1	10.028	BV	0.2824	9512.76367	517.73743	19.0280
2	10.459	VV	0.3417	1.27222e4	550.32629	25.4477
3	17.198	BB	0.6110	1.38877e4	343.98843	27.7791
4	22.250	BBA	0.7709	1.38708e4	277.63614	27.7452

Totals : 4.99935e4 1689.68829

Data File C:\CHEM32\1\DATA\GC\GC_395_RID.D
Sample Name: GC_395_rid

===

Acq. Operator : Giovanni
Acq. Instrument : HPLC-1 Location : Vial 1
Injection Date : 17/03/2022 15:39:59
Acq. Method : C:\CHEM32\1\METHODS\DEF_LC.M
Last changed : 17/03/2022 15:17:43 by Chiara
(modified after loading)
Analysis Method : C:\CHEM32\1\METHODS\DEF_LC.M
Last changed : 03/05/2022 11:01:34 by Chiara
(modified after loading)
Sample Info : GC_395_rid, 1 mL/min, 95:5 hex:ipr, 25°C, IC

Additional Info : Peak(s) manually integrated

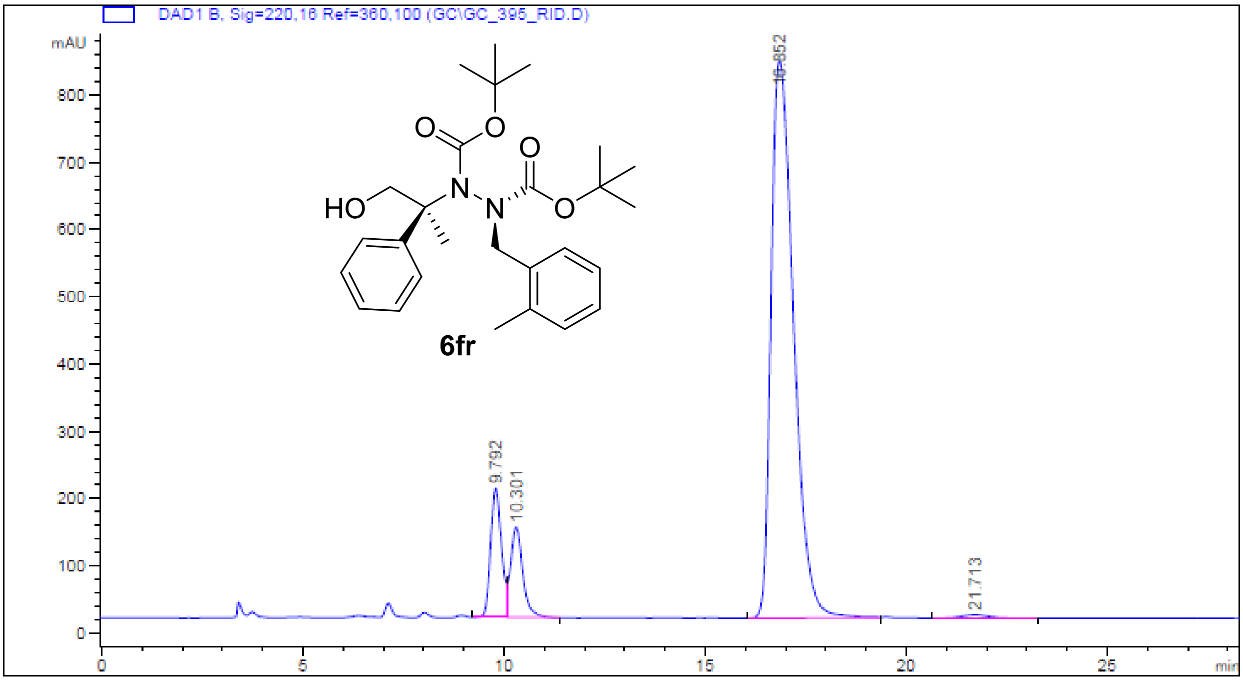

===
Area Percent Report
===

Sorted By : Signal
Multiplier: : 1.0000
Dilution: : 1.0000
Use Multiplier & Dilution Factor with ISTDs

Signal 1: DAD1 B, Sig=220,16 Ref=360,100

Peak #	RetTime [min]	Type	Width [min]	Area [mAU*s]	Height [mAU]	Area %
1	9.792	BV	0.2904	3578.63208	191.15277	9.2112
2	10.301	VB	0.3155	2844.47119	134.05757	7.3215
3	16.852	BV	0.6057	3.21656e4	827.10956	82.7926
4	21.713	BB	0.7931	262.08530	4.92326	0.6746

Totals : 3.88508e4 1157.24316

```
Data File H:\HPLC\1\DATA\GC\GC_346.D
Sample Name: GC_346

=====================================================================
Acq. Operator   : Giovanni
Acq. Instrument : HPLC-1                          Location : Vial 1
Injection Date  : 14/12/2021 12.44.25
Acq. Method     : C:\CHEM32\1\METHODS\DEF_LC.M
Last changed    : 14/12/2021 12.37.29 by Chiara
                  (modified after loading)
Analysis Method : H:\HPLC\1\METHODS\DEF_LC.M
Sample Info     : GC_346, 1 mL/min, 98:2 hex:ipr, 25°C, IC

Additional Info : Peak(s) manually integrated
```

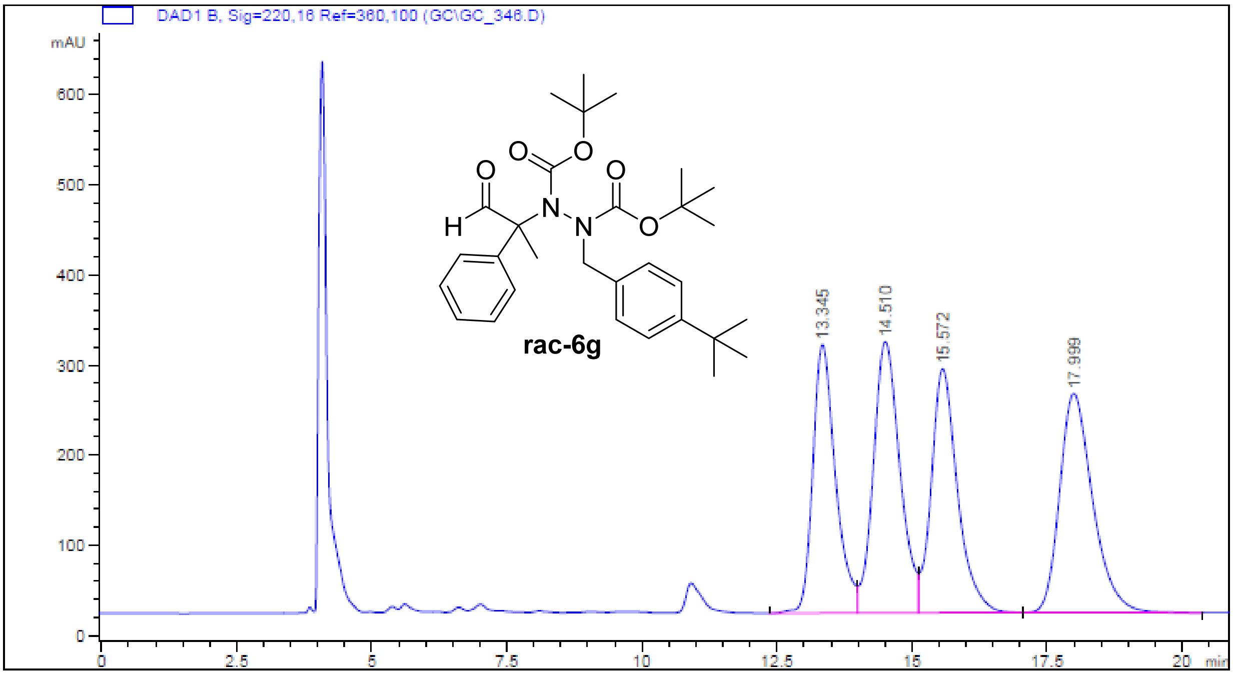


```
=====================================================================
                        Area Percent Report
=====================================================================

Sorted By             :      Signal
Multiplier:                   :      1.0000
Dilution:                     :      1.0000
Use Multiplier & Dilution Factor with ISTDs


Signal 1: DAD1 B, Sig=220,16 Ref=360,100
```

Peak #	RetTime [min]	Type	Width [min]	Area [mAU*s]	Height [mAU]	Area %
1	13.345	BV	0.4374	8722.36230	298.05161	22.6122
2	14.510	VV	0.5216	1.03088e4	300.97156	26.7248
3	15.572	VB	0.5064	9155.16895	270.81717	23.7342
4	17.999	BB	0.6502	1.03874e4	243.27058	26.9288
Totals :				3.85737e4	1113.11092	

Data File H:\HPLC\1\DATA\GC\GC_394_1.D
Sample Name: GC_394

===

```
Acq. Operator   : Giovanni
Acq. Instrument : HPLC-1                          Location : Vial 1
Injection Date  : 07/03/2022 15.13.17
Acq. Method     : C:\CHEM32\1\METHODS\DEF_LC.M
Last changed    : 07/03/2022 15.10.35 by Giovanni
                  (modified after loading)
Analysis Method : H:\HPLC\1\METHODS\DEF_LC.M
Sample Info     : GC_394, 1 mL/min, 98:2 hex:ipr, 25°C, IC

Additional Info : Peak(s) manually integrated
```

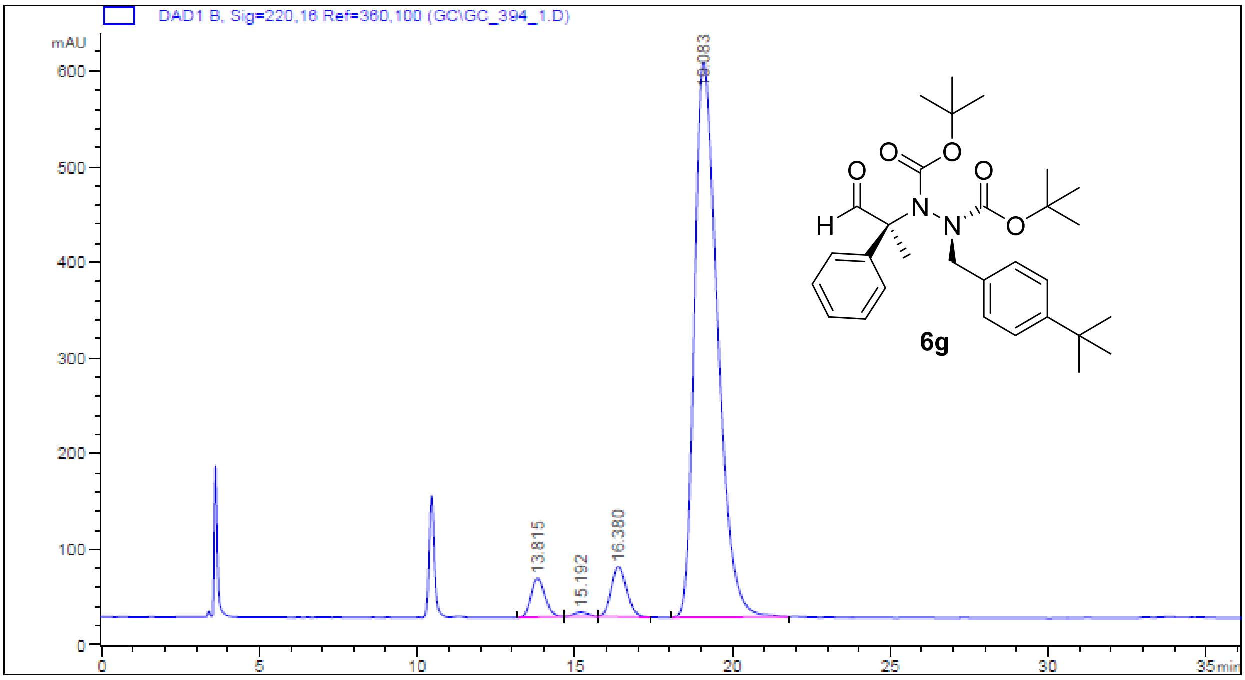

===
Area Percent Report
===

```
Sorted By             :      Signal
Multiplier:                  :      1.0000
Dilution:                    :      1.0000
Use Multiplier & Dilution Factor with ISTDs
```

Signal 1: DAD1 B, Sig=220,16 Ref=360,100

Peak #	RetTime [min]	Type	Width [min]	Area [mAU*s]	Height [mAU]	Area %
1	13.815	BB	0.4727	1235.50659	40.18773	3.7722
2	15.192	BB	0.4528	130.51820	4.31615	0.3985
3	16.380	BB	0.5252	1772.05920	52.31383	5.4104
4	19.083	BB	0.7834	2.96147e4	580.30078	90.4189

Totals : 3.27527e4 677.11849

Data File C:\CHEM32\1\DATA\GC\GC_349.D
Sample Name: GC_349

===

Acq. Operator : Giovanni
Acq. Instrument : HPLC-1 Location : Vial 1
Injection Date : 15/12/2021 14:06:37
Acq. Method : C:\CHEM32\1\METHODS\DEF_LC.M
Last changed : 15/12/2021 14:05:14 by Chiara
(modified after loading)
Analysis Method : C:\CHEM32\1\METHODS\DEF_LC.M
Last changed : 03/05/2022 10:57:34 by Chiara
(modified after loading)
Sample Info : GC_349, 1 mL/min, 98:2 hex:ipr, 25°C, IC

Additional Info : Peak(s) manually integrated

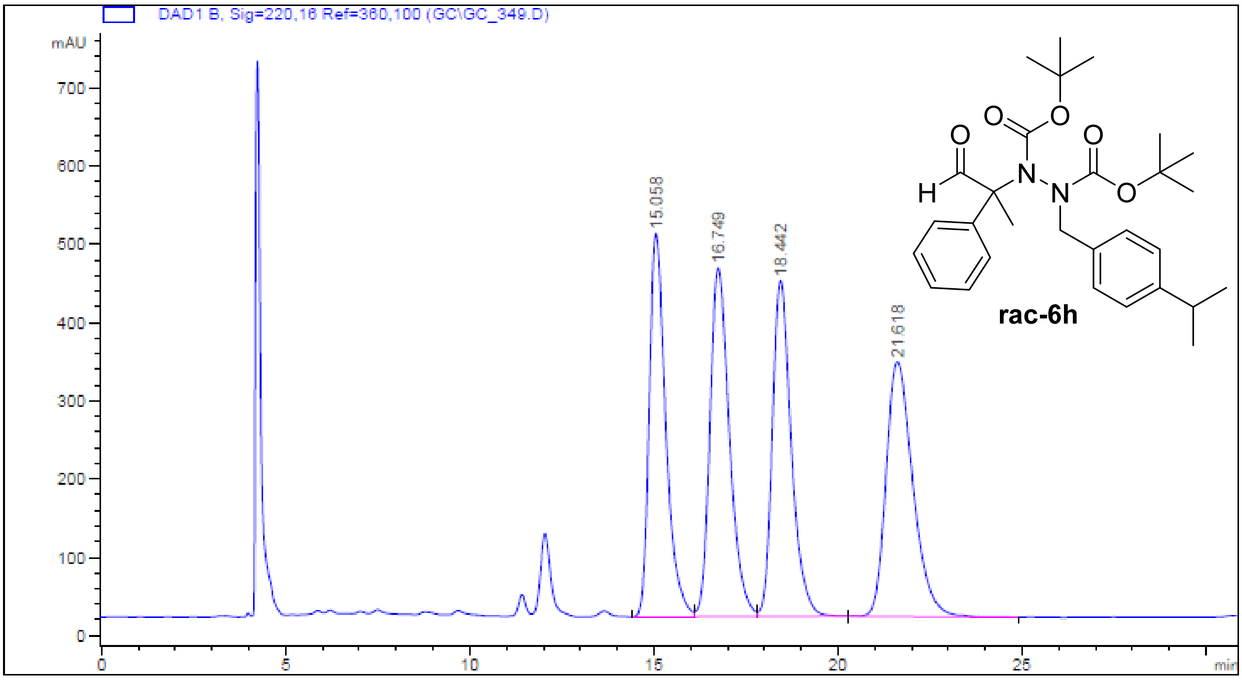

===
Area Percent Report
===

Sorted By : Signal
Multiplier: : 1.0000
Dilution: : 1.0000
Use Multiplier & Dilution Factor with ISTDs

Signal 1: DAD1 B, Sig=220,16 Ref=360,100

Peak #	RetTime [min]	Type	Width [min]	Area [mAU*s]	Height [mAU]	Area %
1	15.058	BV	0.4770	1.53105e4	489.37558	24.0669
2	16.749	VV	0.5620	1.64188e4	445.48318	25.8092
3	18.442	VB	0.5415	1.53625e4	429.32986	24.1488
4	21.618	BB	0.7770	1.65244e4	325.08359	25.9751

Totals : 6.36161e4 1689.27222

Data File C:\CHEM32\1\DATA\GC\GC_391_1.D
Sample Name: GC_391

===

Acq. Operator : Giovanni
Acq. Instrument : HPLC-1 Location : Vial 1
Injection Date : 28/02/2022 15:33:21
Acq. Method : C:\CHEM32\1\METHODS\DEF_LC.M
Last changed : 28/02/2022 15:31:53 by Giovanni
(modified after loading)
Analysis Method : C:\CHEM32\1\METHODS\DEF_LC.M
Last changed : 03/05/2022 10:58:27 by Chiara
(modified after loading)
Sample Info : GC_391, 1 mL/min, 98:2 hex:ipr, 25°C, IC

Additional Info : Peak(s) manually integrated

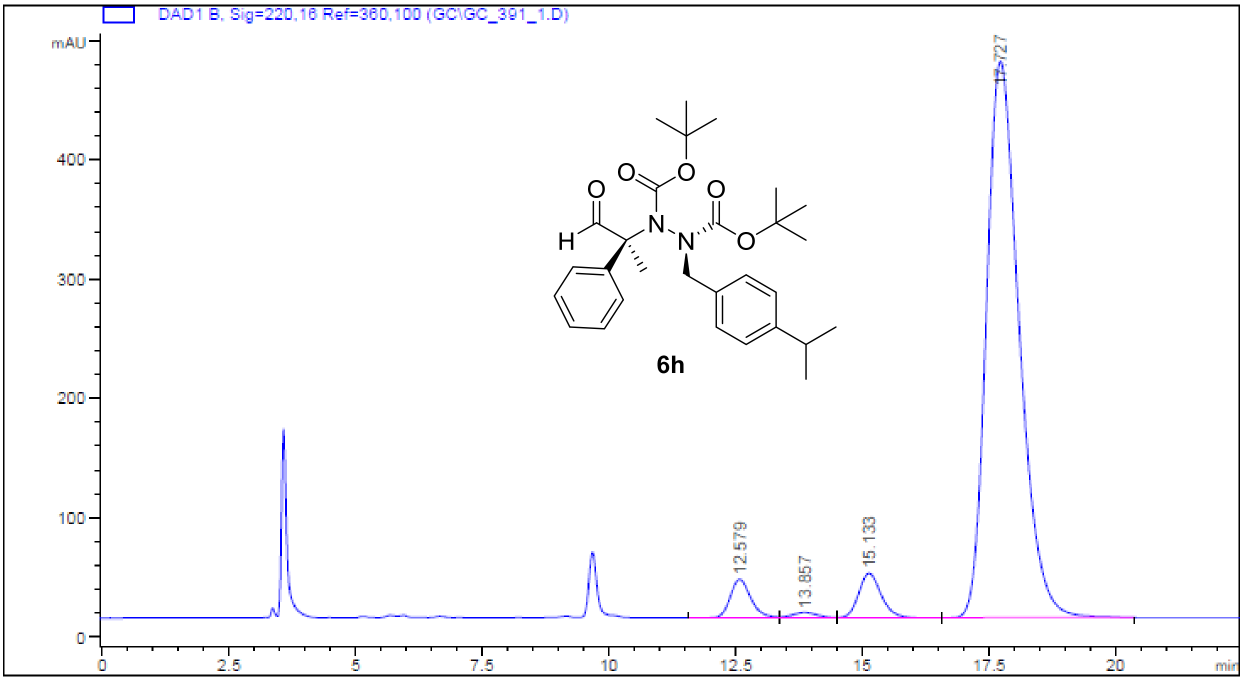

===

Area Percent Report

===

Sorted By : Signal
Multiplier: : 1.0000
Dilution: : 1.0000
Use Multiplier & Dilution Factor with ISTDs

Signal 1: DAD1 B, Sig=220,16 Ref=360,100

Peak #	RetTime [min]	Type	Width [min]	Area [mAU*s]	Height [mAU]	Area %
1	12.579	BV	0.4320	908.97858	32.13691	3.9359
2	13.857	VV	0.5014	154.03836	4.43269	0.6670
3	15.133	VB	0.4727	1154.63843	37.34182	4.9996
4	17.727	BB	0.6884	2.08769e4	466.26382	90.3975

Totals : 2.30946e4 540.17524

Data File C:\CHEM32\1\DATA\GC\GC_361_RID.D
Sample Name: GC_361_rid

===

Acq. Operator : Giovanni
Acq. Instrument : HPLC-1 Location : Vial 1
Injection Date : 22/02/2022 09:15:51
Acq. Method : C:\CHEM32\1\METHODS\DEF_LC.M
Last changed : 22/02/2022 09:13:58 by Chiara
(modified after loading)
Analysis Method : C:\CHEM32\1\METHODS\DEF_LC.M
Last changed : 03/05/2022 10:44:55 by Chiara
(modified after loading)
Sample Info : GC_361_ridotto, 1 mL/min, 90:10 hex:ipr, 25°C, IC

Additional Info : Peak(s) manually integrated

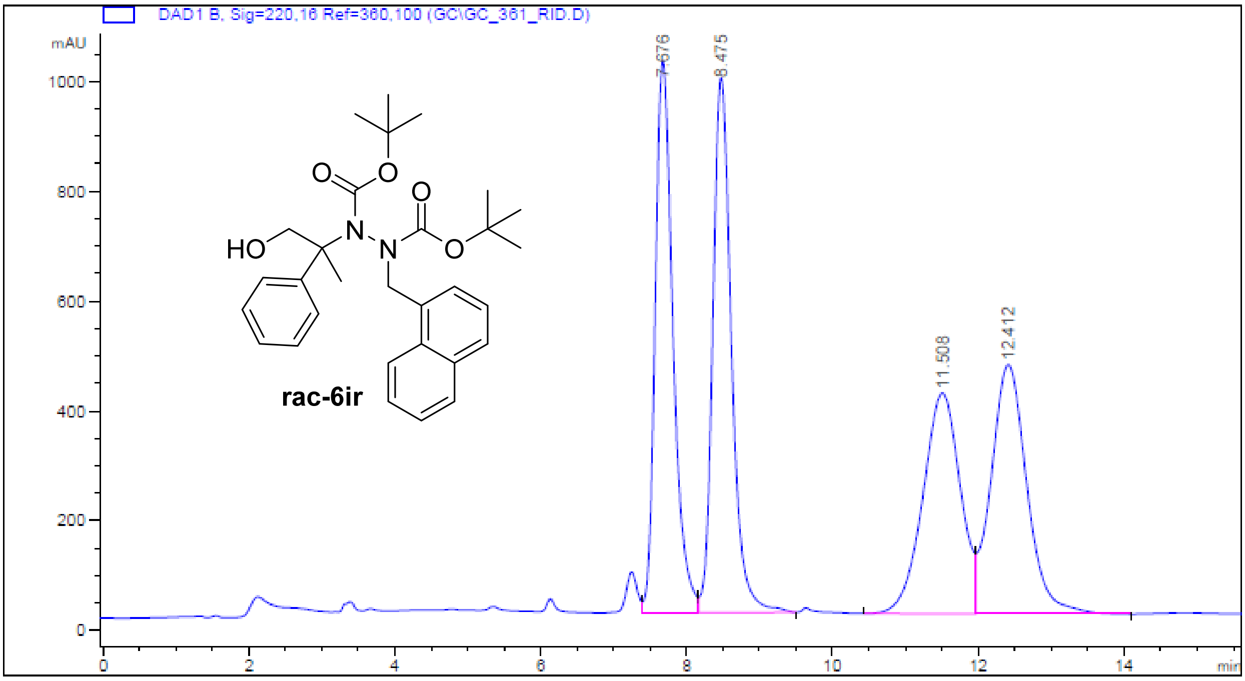

===
Area Percent Report
===

Sorted By : Signal
Multiplier: : 1.0000
Dilution: : 1.0000
Use Multiplier & Dilution Factor with ISTDs

Signal 1: DAD1 B, Sig=220,16 Ref=360,100

Peak #	RetTime [min]	Type	Width [min]	Area [mAU*s]	Height [mAU]	Area %
1	7.676	VV	0.2601	1.70587e4	1004.81989	26.5610
2	8.475	VB	0.2733	1.73305e4	975.15918	26.9842
3	11.508	BV	0.5450	1.43717e4	402.06247	22.3772
4	12.412	VB	0.5194	1.54638e4	453.99808	24.0776

Totals : 6.42247e4 2836.03961

```
Data File H:\HPLC\1\DATA\GC\GC_387_RID_1.D
Sample Name: GC_387_rid

    =====================================================================
    Acq. Operator   : Giovanni
    Acq. Instrument : HPLC-1                          Location : Vial 1
    Injection Date  : 01/03/2022 16.37.40
    Acq. Method     : C:\CHEM32\1\METHODS\DEF_LC.M
    Last changed    : 01/03/2022 16.28.33 by Giovanni
                      (modified after loading)
    Analysis Method : H:\HPLC\1\METHODS\DEF_LC.M
    Last changed    : 21/05/2022 14.11.49
                      (modified after loading)
    Sample Info     : GC_387_rid, 1 mL/min, 90:10 hex:ipr, 25°C, IC

    Additional Info : Peak(s) manually integrated
```

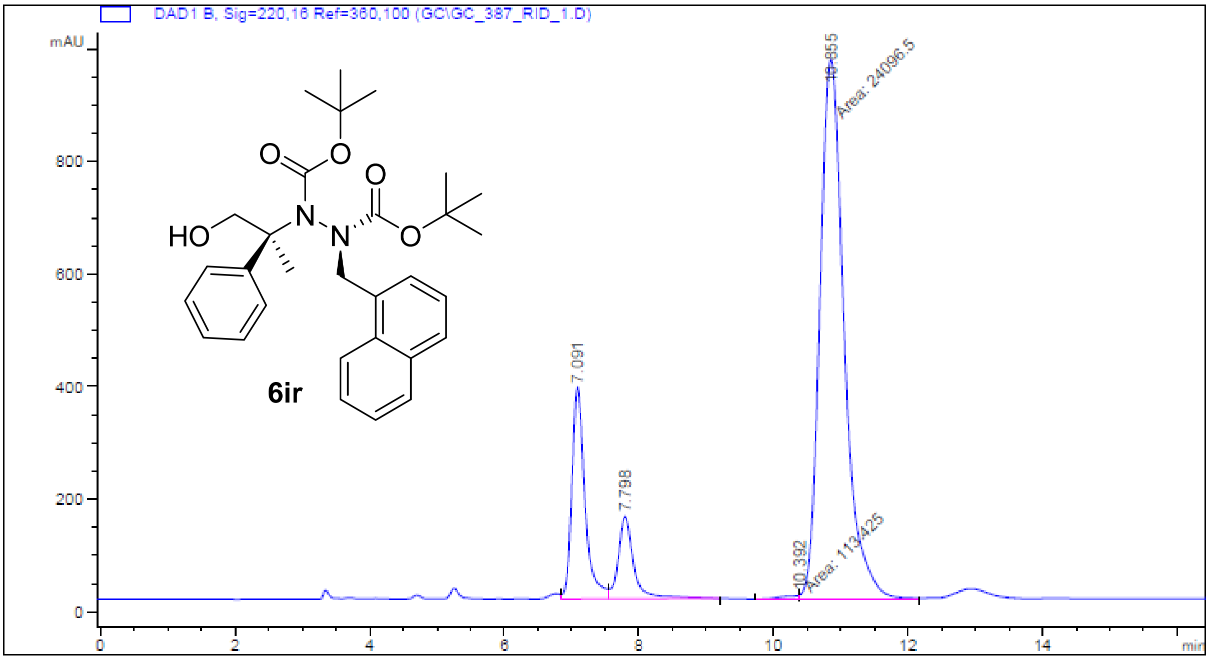


```
    =====================================================================
                         Area Percent Report
    =====================================================================

    Sorted By             :      Signal
    Multiplier:                    :      1.0000
    Dilution:                      :      1.0000
    Use Multiplier & Dilution Factor with ISTDs


    Signal 1: DAD1 B, Sig=220,16 Ref=360,100
```

Peak #	RetTime [min]	Type	Width [min]	Area [mAU*s]	Height [mAU]	Area %
1	7.091	VV	0.2050	5130.84180	376.39688	16.1940
2	7.798	VB	0.2372	2342.85303	146.00117	7.3945
3	10.392	MF	0.2664	113.42534	7.09741	0.3580
4	10.855	FM	0.4188	2.40965e4	958.92871	76.0535

Totals : 3.16836e4 1488.42417

Data File H:\HPLC\1\DATA\GC\GC_343_10.D
Sample Name: GC_343

===

Acq. Operator : Giovanni
Acq. Instrument : HPLC-1 Location : Vial 1
Injection Date : 05/05/2022 15.46.17
Acq. Method : C:\CHEM32\1\METHODS\DEF_LC.M
Last changed : 05/05/2022 15.38.27 by Giovanni
(modified after loading)
Analysis Method : H:\HPLC\1\METHODS\DEF_LC.M
Last changed : 21/05/2022 14.11.49
(modified after loading)
Sample Info : GC_343, 0.5 mL/min, 98:2 hex:ipr, 25°C, OD-H

Additional Info : Peak(s) manually integrated

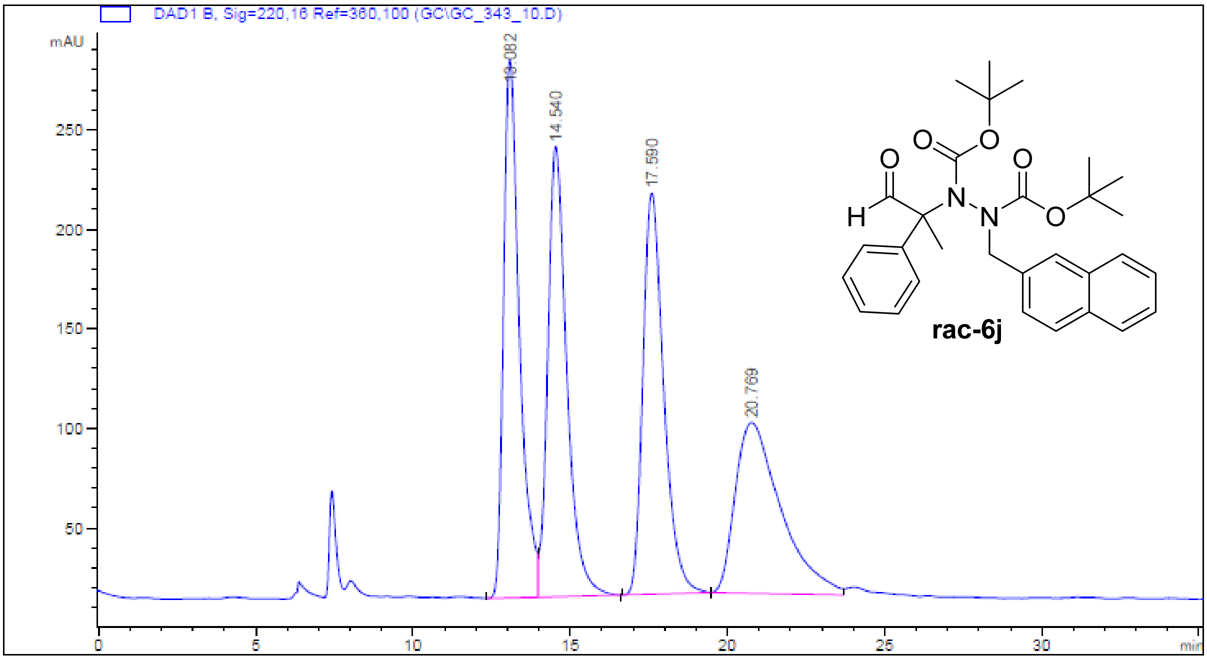

===
Area Percent Report
===

Sorted By : Signal
Multiplier: : 1.0000
Dilution: : 1.0000
Use Multiplier & Dilution Factor with ISTDs

Signal 1: DAD1 B, Sig=220,16 Ref=360,100

Peak #	RetTime [min]	Type	Width [min]	Area [mAU*s]	Height [mAU]	Area %
1	13.082	BV	0.5315	9539.87891	270.45291	25.7755
2	14.540	VB	0.6315	9531.51367	226.25156	25.7529
3	17.590	BB	0.7061	9286.42773	201.33719	25.0907
4	20.769	BV	1.4861	8653.54199	85.69472	23.3808

Totals : 3.70114e4 783.73637

Data File H:\HPLC\1\DATA\GC\GC_389_2.D
Sample Name: GC_389

===
Acq. Operator   : Giovanni
Acq. Instrument : HPLC-1   Location : Vial 1
Injection Date : 05/05/2022 16.49.21
Acq. Method   : C:\CHEM32\1\METHODS\DEF_LC.M
Last changed   : 05/05/2022 16.49.02 by Giovanni
                  (modified after loading)
Analysis Method : H:\HPLC\1\METHODS\DEF_LC.M
Last changed   : 21/05/2022 14.11.49
                  (modified after loading)
Sample Info   : GC_389, 0.5 mL/min, 98:2 hex:ipr, 25°C, OD-H

Additional Info : Peak(s) manually integrated

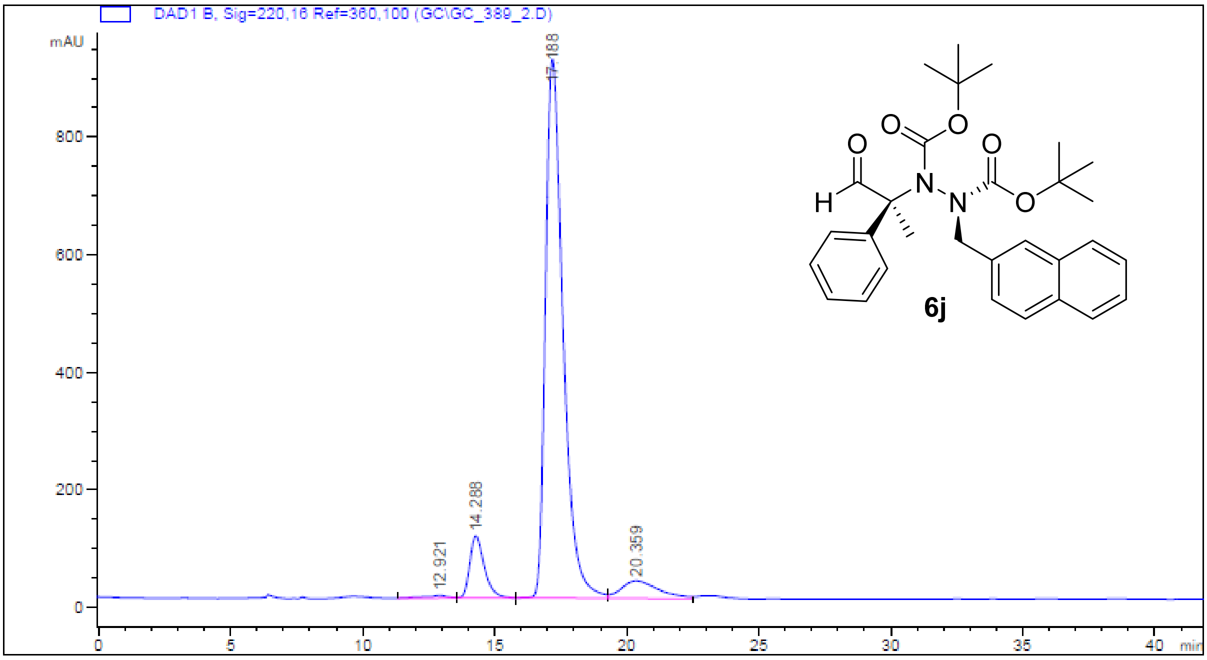

===
Area Percent Report
===

Sorted By   :   Signal
Multiplier: :   1.0000
Dilution: :   1.0000
Use Multiplier & Dilution Factor with ISTDs

Signal 1: DAD1 B, Sig=220,16 Ref=360,100

Peak #	RetTime [min]	Type	Width [min]	Area [mAU*s]	Height [mAU]	Area %
1	12.921	BV	0.7794	263.05069	4.55725	0.5297
2	14.288	VB	0.5767	4019.73462	105.45032	8.0942
3	17.188	BV	0.7050	4.24463e4	915.39722	85.4701
4	20.359	VV	1.4307	2933.07007	29.84169	5.9061

Totals :   4.96621e4   1055.24648

```
Data File H:\HPLC\1\DATA\GC\GC_385_RID_5.D
Sample Name: GC_385_rid

    =====================================================================
    Acq. Operator   : Giovanni
    Acq. Instrument : HPLC-1                          Location : Vial 1
    Injection Date  : 29/03/2022 10.57.33
    Acq. Method     : C:\CHEM32\1\METHODS\DEF_LC.M
    Last changed    : 29/03/2022 10.05.29 by Giovanni
                      (modified after loading)
    Analysis Method : H:\HPLC\1\METHODS\DEF_LC.M
    Last changed    : 07/05/2022 14.02.09
                      (modified after loading)
    Sample Info     : GC_385_rid, 1 mL/min, 90:10 hex:ipr, 25°C, Lux 5u cellu
                      lose-2

    Additional Info : Peak(s) manually integrated
```

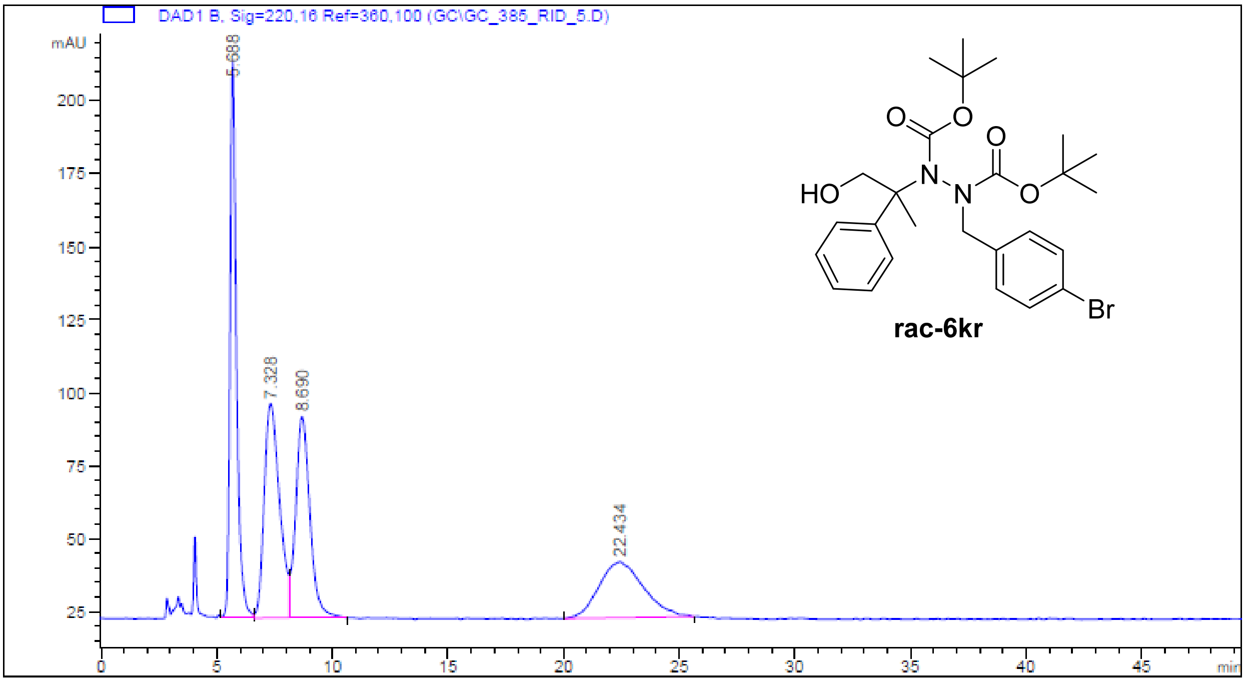


```
    =====================================================================
                         Area Percent Report
    =====================================================================

    Sorted By             :      Signal
    Multiplier:                  :      1.0000
    Dilution:                    :      1.0000
    Use Multiplier & Dilution Factor with ISTDs


    Signal 1: DAD1 B, Sig=220,16 Ref=360,100
```

Peak #	RetTime [min]	Type	Width [min]	Area [mAU*s]	Height [mAU]	Area %
1	5.688	VV	0.2934	3714.81152	190.60124	29.4185
2	7.328	VV	0.7050	3387.54224	73.32783	26.8268
3	8.690	VB	0.6535	2960.59082	68.86859	23.4456
4	22.434	BB	1.6152	2564.52075	19.18918	20.3091

Totals : 1.26275e4 351.98684

Data File H:\HPLC\1\DATA\GC\GC_376_RID_2.D
Sample Name: GC_376_RID

===

```
Acq. Operator   : Giovanni
Acq. Instrument : HPLC-1                          Location : Vial 1
Injection Date  : 29/03/2022 13.01.29
Acq. Method     : C:\CHEM32\1\METHODS\DEF_LC.M
Last changed    : 29/03/2022 12.57.10 by Giovanni
                  (modified after loading)
Analysis Method : H:\HPLC\1\METHODS\DEF_LC.M
Last changed    : 07/05/2022 14.10.49
                  (modified after loading)
Sample Info     : GC_376_RID, 1 mL/min, 90:10 hex:ipr, 25°C, Lux 5u cellu
                  lose-2
```

Additional Info : Peak(s) manually integrated

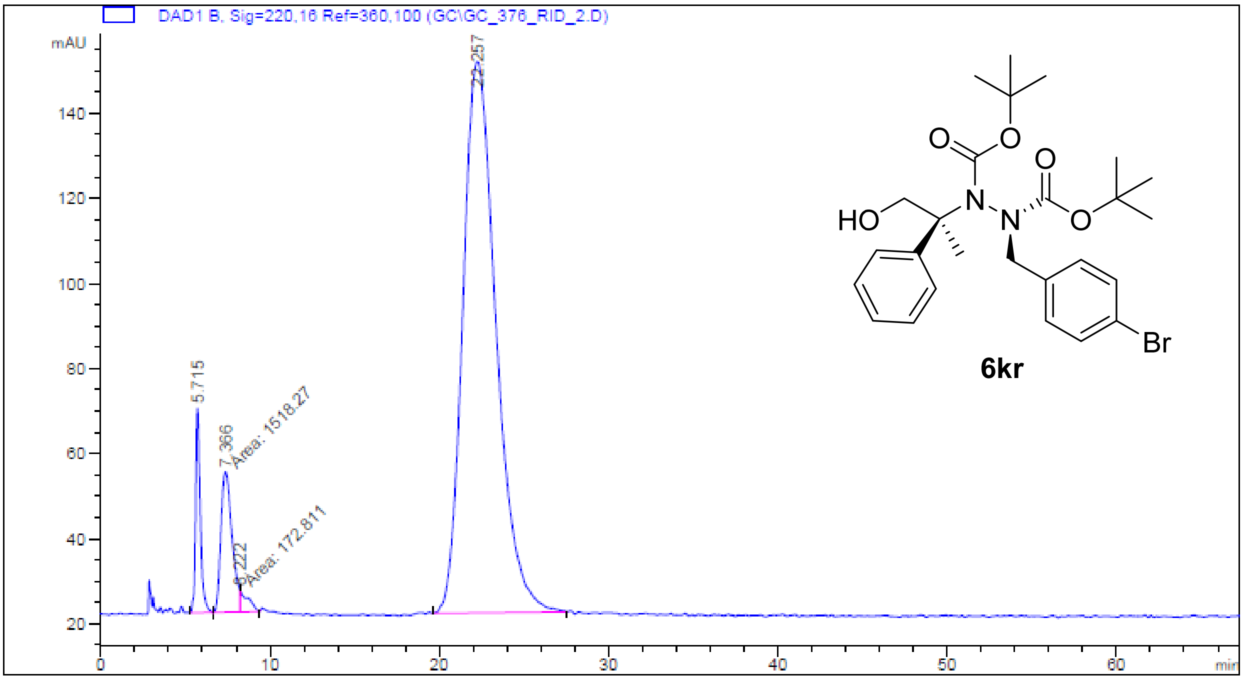

===

Area Percent Report

===

```
Sorted By             :      Signal
Multiplier:                  :      1.0000
Dilution:                    :      1.0000
Use Multiplier & Dilution Factor with ISTDs
```

Signal 1: DAD1 B, Sig=220,16 Ref=360,100

Peak #	RetTime [min]	Type	Width [min]	Area [mAU*s]	Height [mAU]	Area %
1	5.715	BB	0.2904	950.22357	48.12112	4.6728
2	7.366	MF	0.7660	1518.27429	33.03587	7.4662
3	8.222	FM	0.5561	172.81067	5.17892	0.8498
4	22.257	BB	1.8979	1.76941e4	129.67358	87.0113

Totals : 2.03354e4 216.00949

Data File C:\CHEM32\1\DATA\GC\GC_400_RF_MIN_1D.D
Sample Name: GC_400_rf_min

===

Acq. Operator : Giovanni
Acq. Instrument : HPLC-1 Location : Vial 1
Injection Date : 18/03/2022 10:04:28
Acq. Method : C:\CHEM32\1\METHODS\DEF_LC.M
Last changed : 18/03/2022 09:31:54 by Giovanni
(modified after loading)
Analysis Method : C:\CHEM32\1\METHODS\DEF_LC.M
Last changed : 03/05/2022 10:49:20 by Chiara
(modified after loading)
Sample Info : GC_400_rf_min, 1 mL/min, 90:10 hex:ipr, 25°C, IC

Additional Info : Peak(s) manually integrated

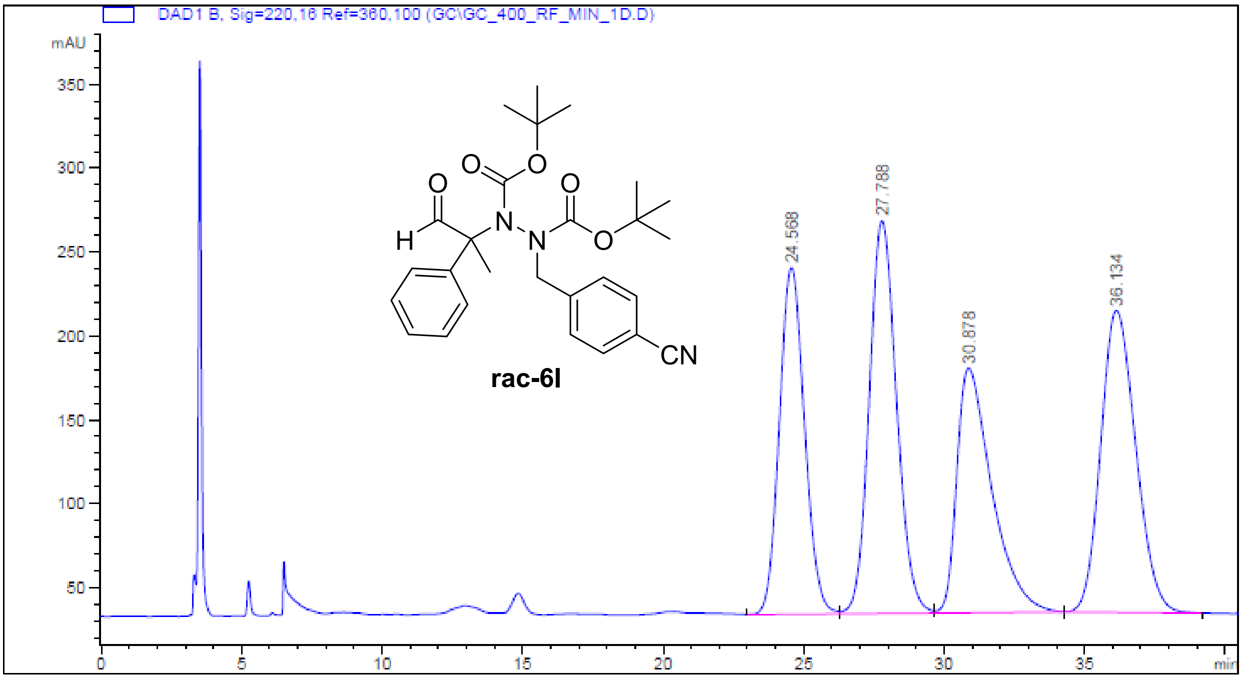

===
Area Percent Report
===

Sorted By : Signal
Multiplier: : 1.0000
Dilution: : 1.0000
Use Multiplier & Dilution Factor with ISTDs

Signal 1: DAD1 B, Sig=220,16 Ref=360,100

Peak #	RetTime [min]	Type	Width [min]	Area [mAU*s]	Height [mAU]	Area %
1	24.568	BV	0.9609	1.28655e4	206.47943	22.6232
2	27.788	VV	1.0316	1.58008e4	234.06561	27.7847
3	30.878	VB	1.2935	1.26204e4	145.85873	22.1921
4	36.134	BB	1.3308	1.55820e4	179.84575	27.4000

Totals : 5.68686e4 766.24953

Data File H:\HPLC\1\DATA\GC\GC_418.D
Sample Name: GC_418

===

Acq. Operator : Giovanni
Acq. Instrument : HPLC-1 Location : Vial 1
Injection Date : 06/05/2022 16.24.53
Acq. Method : C:\CHEM32\1\METHODS\DEF_LC.M
Last changed : 06/05/2022 16.23.51 by Giovanni
(modified after loading)
Analysis Method : H:\HPLC\1\METHODS\DEF_LC.M
Last changed : 21/05/2022 14.11.49
(modified after loading)
Sample Info : GC_418, 1 mL/min, 90:10 hex:ipr, 25°C, IC

Additional Info : Peak(s) manually integrated

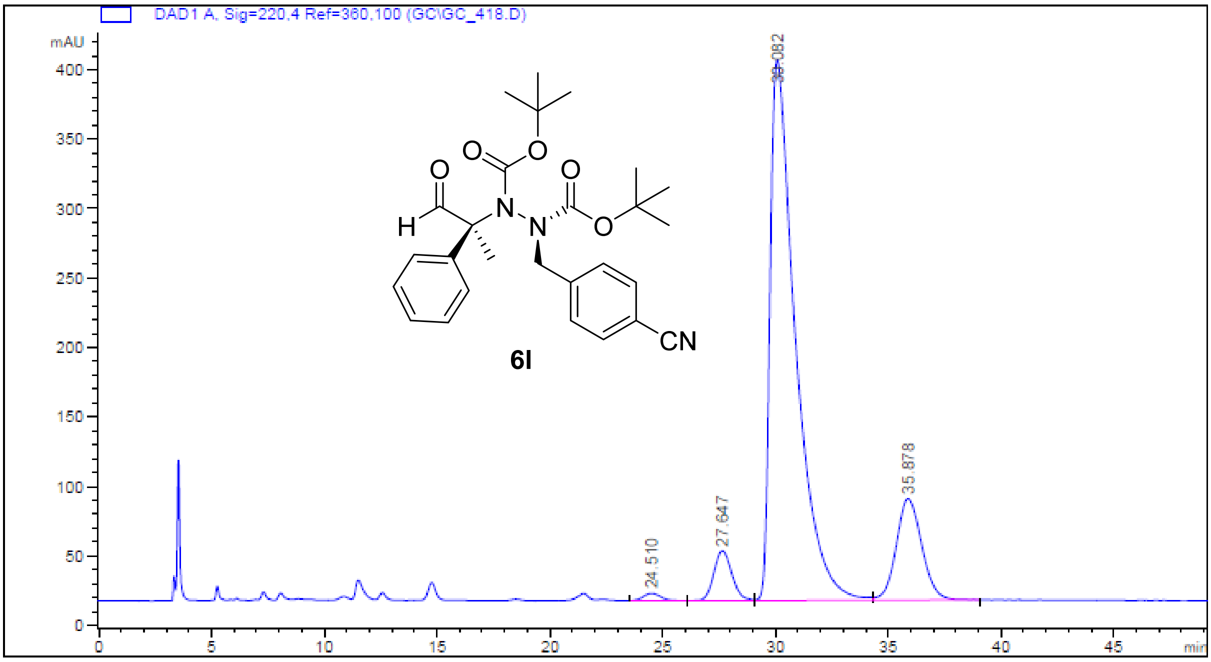

===
Area Percent Report
===

Sorted By : Signal
Multiplier: : 1.0000
Dilution: : 1.0000
Use Multiplier & Dilution Factor with ISTDs

Signal 1: DAD1 A, Sig=220,4 Ref=360,100

Peak #	RetTime [min]	Type	Width [min]	Area [mAU*s]	Height [mAU]	Area %
1	24.510	VB	0.8072	292.92017	5.31335	0.7620
2	27.647	BV	0.8593	1989.95520	35.86528	5.1770
3	30.082	VV	1.1524	3.04310e4	389.10516	79.1676
4	35.878	VB	1.1941	5724.81250	73.06770	14.8934

Totals : 3.84387e4 503.35150

Data File H:\HPLC\1\DATA\GC\GC_401_RF_MIN_4.D
Sample Name: GC_401_RF_MIN

===

Acq. Operator : Giovanni
Acq. Instrument : HPLC-1 Location : Vial 1
Injection Date : 01/04/2022 12.30.28
Acq. Method : C:\CHEM32\1\METHODS\DEF_LC.M
Last changed : 01/04/2022 12.16.07 by Giovanni
(modified after loading)
Analysis Method : H:\HPLC\1\METHODS\DEF_LC.M
Last changed : 07/05/2022 14.10.49
(modified after loading)
Sample Info : GC_401_RF_MIN, 1 mL/min, 90:10 hex:ipr, 25°C, Lux 5u cellulose-2

Additional Info : Peak(s) manually integrated

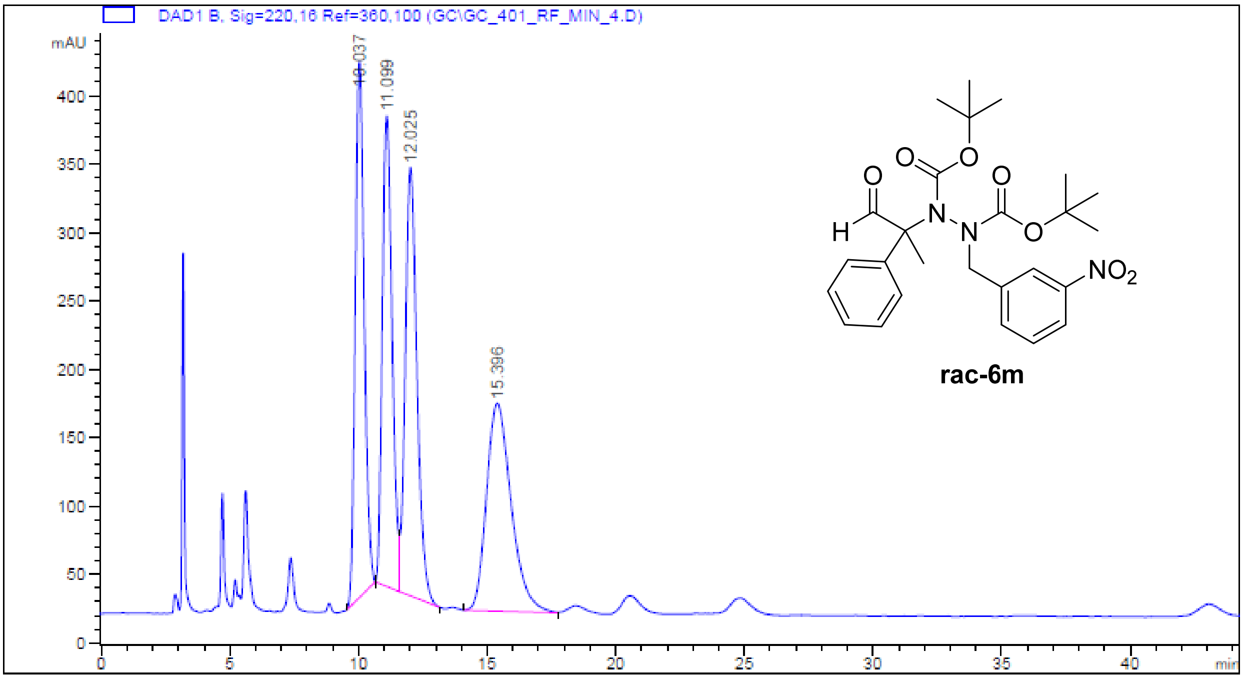

===

Area Percent Report

===

Sorted By : Signal
Multiplier: : 1.0000
Dilution: : 1.0000
Use Multiplier & Dilution Factor with ISTDs

Signal 1: DAD1 B, Sig=220,16 Ref=360,100

Peak #	RetTime [min]	Type	Width [min]	Area [mAU*s]	Height [mAU]	Area %
1	10.037	BB	0.3726	9422.60742	391.78265	24.4395
2	11.099	BV	0.4078	9071.34180	343.88449	23.5284
3	12.025	VB	0.4813	9911.47363	313.10593	25.7074
4	15.396	BV	1.0271	1.01495e4	151.97836	26.3247

Totals : 3.85549e4 1200.75143

Data File H:\HPLC\1\DATA\GC\GC_406.D
Sample Name: GC_406

===

Acq. Operator : Giovanni
Acq. Instrument : HPLC-1 Location : Vial 1
Injection Date : 05/04/2022 15.54.52
Acq. Method : C:\CHEM32\1\METHODS\DEF_LC.M
Last changed : 05/04/2022 15.14.26 by alberto
(modified after loading)
Analysis Method : H:\HPLC\1\METHODS\DEF_LC.M
Last changed : 07/05/2022 14.10.49
(modified after loading)
Sample Info : GC_406, 1 mL/min, 90:10 hex:ipr, 25°C, Lux 5u cellulose -2

Additional Info : Peak(s) manually integrated

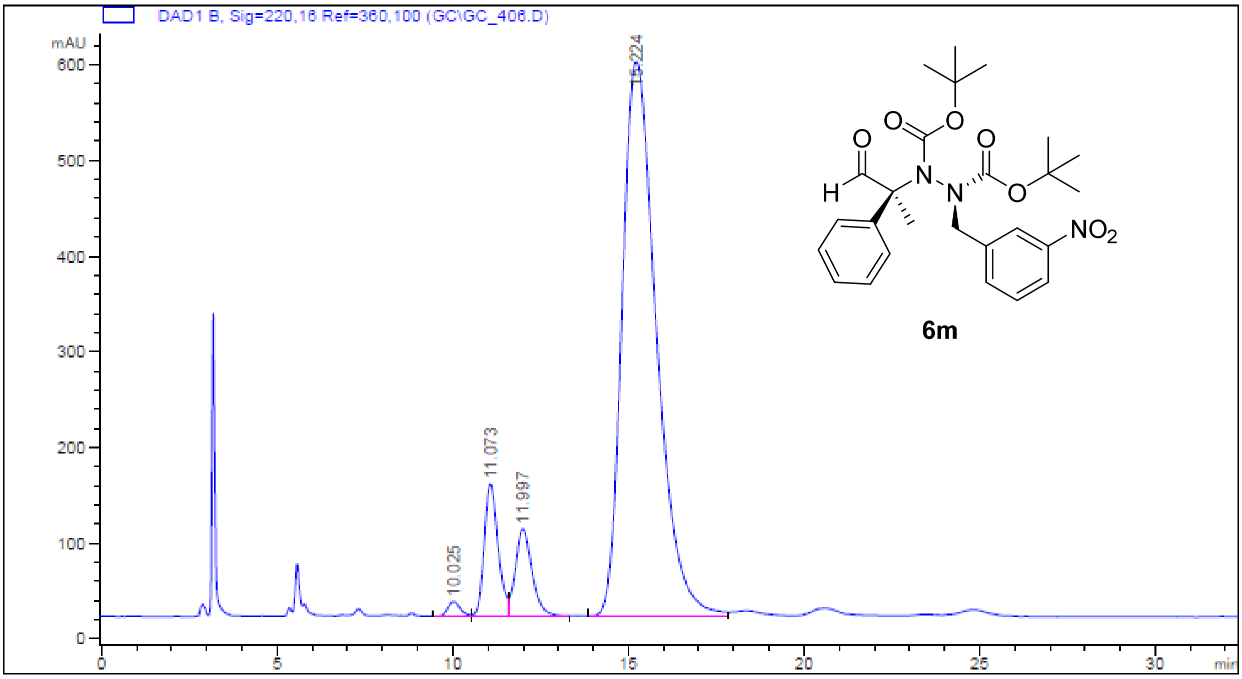

===

Area Percent Report

===

Sorted By : Signal
Multiplier: : 1.0000
Dilution: : 1.0000
Use Multiplier & Dilution Factor with ISTDs

Signal 1: DAD1 B, Sig=220,16 Ref=360,100

Peak #	RetTime [min]	Type	Width [min]	Area [mAU*s]	Height [mAU]	Area %
1	10.025	BV	0.3781	375.53873	15.20502	0.8082
2	11.073	VV	0.4216	3810.45801	138.24240	8.2010
3	11.997	VB	0.4963	3002.30493	91.13885	6.4617
4	15.224	BV	1.0347	3.92751e4	579.49292	84.5291

Totals : 4.64634e4 824.07919

```
Data File H:\HPLC\1\DATA\CP\CP_237_2.D
Sample Name: CP_237_2

    =====================================================================
    Acq. Operator   : Chiara
    Acq. Instrument : HPLC-1                          Location : Vial 1
    Injection Date  : 21/03/2022 10:00:30
    Acq. Method     : C:\CHEM32\1\METHODS\DEF_LC.M
    Last changed    : 21/03/2022 09:40:25 by Chiara
                      (modified after loading)
    Analysis Method : H:\HPLC\2\METHODS\DEF_LC.M
    Last changed    : 06/06/2022 15:16:29
                      (modified after loading)
    Sample Info     : CP_237_2, 1 mL/min, 90:10 hex:ipr, 25°C, IC

    Additional Info : Peak(s) manually integrated
```

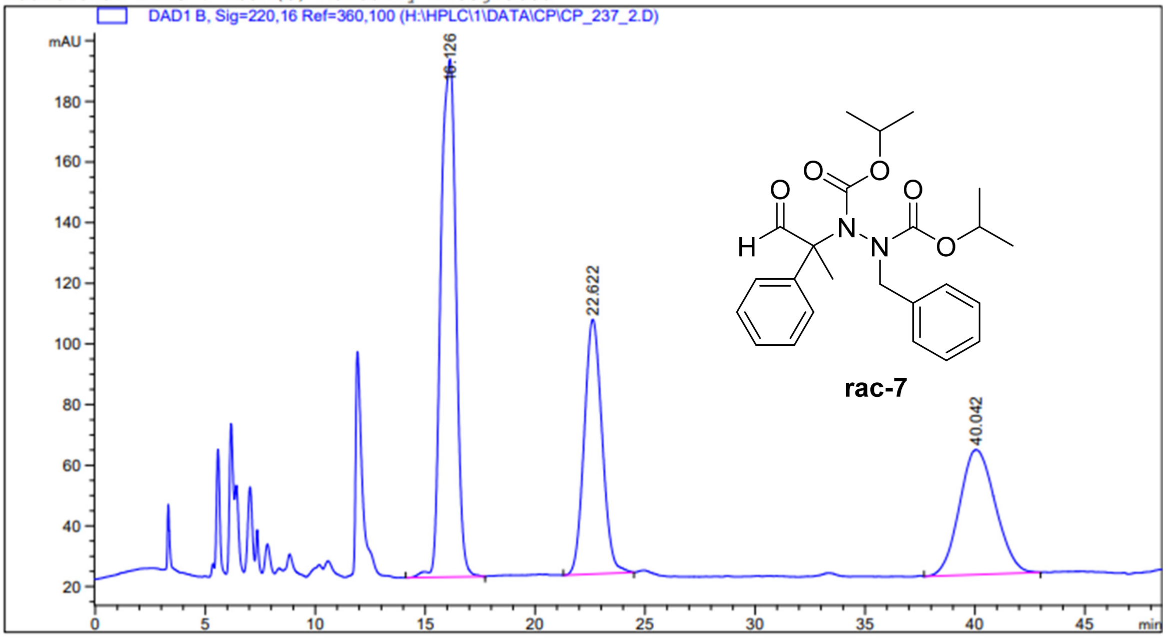


```
    =====================================================================
                            Area Percent Report
    =====================================================================

    Sorted By             :      Signal
    Multiplier:                   :      1.0000
    Dilution:                     :      1.0000
    Use Multiplier & Dilution Factor with ISTDs


    Signal 1: DAD1 B, Sig=220,16 Ref=360,100

    Peak RetTime Type  Width     Area      Height     Area
      #   [min]        [min]   [mAU*s]     [mAU]       %
    ----|-------|----|-------|----------|----------|--------|
       1  16.126 BB     0.6750 8181.92627  170.75758  46.5221
       2  22.622 BB     0.8831 4731.23242   84.00115  26.9016
       3  40.042 BB     1.6944 4674.01611   41.17331  26.5763

    Totals :                   1.75872e4   295.93204
```

```
Data File H:\HPLC\1\DATA\CP\CP_238_2.D
Sample Name: CP_238_2

=====================================================================
Acq. Operator   : Chiara
Acq. Instrument : HPLC-1                          Location : Vial 1
Injection Date  : 21/03/2022 10:51:00
Acq. Method     : C:\CHEM32\1\METHODS\DEF_LC.M
Last changed    : 21/03/2022 10:49:26 by Chiara
                  (modified after loading)
Analysis Method : H:\HPLC\2\METHODS\DEF_LC.M
Last changed    : 06/06/2022 15:17:37
                  (modified after loading)
Sample Info     : CP_238_2, 1 mL/min, 90:10 hex:ipr, 25°C, IC

Additional Info : Peak(s) manually integrated
```

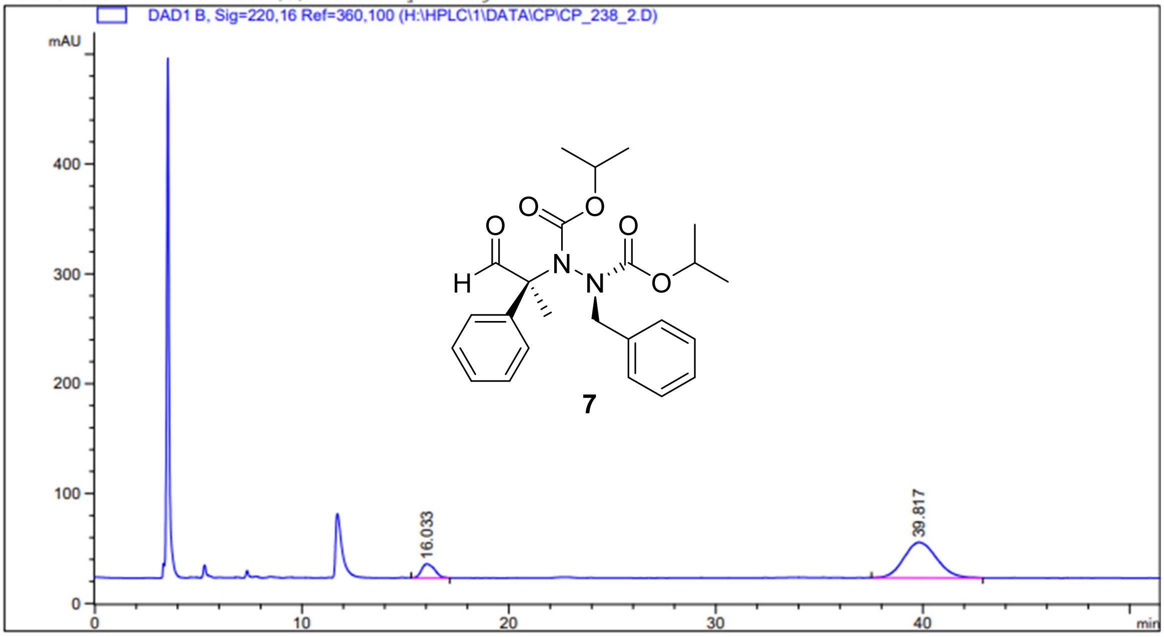


```
=====================================================================
                          Area Percent Report
=====================================================================

Sorted By             :      Signal
Multiplier:                  :      1.0000
Dilution:                    :      1.0000
Use Multiplier & Dilution Factor with ISTDs


Signal 1: DAD1 B, Sig=220,16 Ref=360,100
```

Peak #	RetTime [min]	Type	Width [min]	Area [mAU*s]	Height [mAU]	Area %
1	16.033	BB	0.7152	556.44891	12.65098	13.5177
2	39.817	BB	1.4642	3560.00342	32.39307	86.4823

Totals : 4116.45233 45.04405

Data File C:\CHEM32\1\DATA\GC\GC_368_RID_3.D
Sample Name: GC_368_rid

===

Acq. Operator : Giovanni
Acq. Instrument : HPLC-1 Location : Vial 1
Injection Date : 22/03/2022 17:31:10
Acq. Method : C:\CHEM32\1\METHODS\DEF_LC.M
Last changed : 22/03/2022 17:06:28 by Giovanni
(modified after loading)
Analysis Method : C:\CHEM32\1\METHODS\DEF_LC.M
Last changed : 03/05/2022 10:51:32 by Chiara
(modified after loading)
Sample Info : GC_368_rid, 1 mL/min, 97.5:2.5 hex:ipr, 25°C, IC

Additional Info : Peak(s) manually integrated

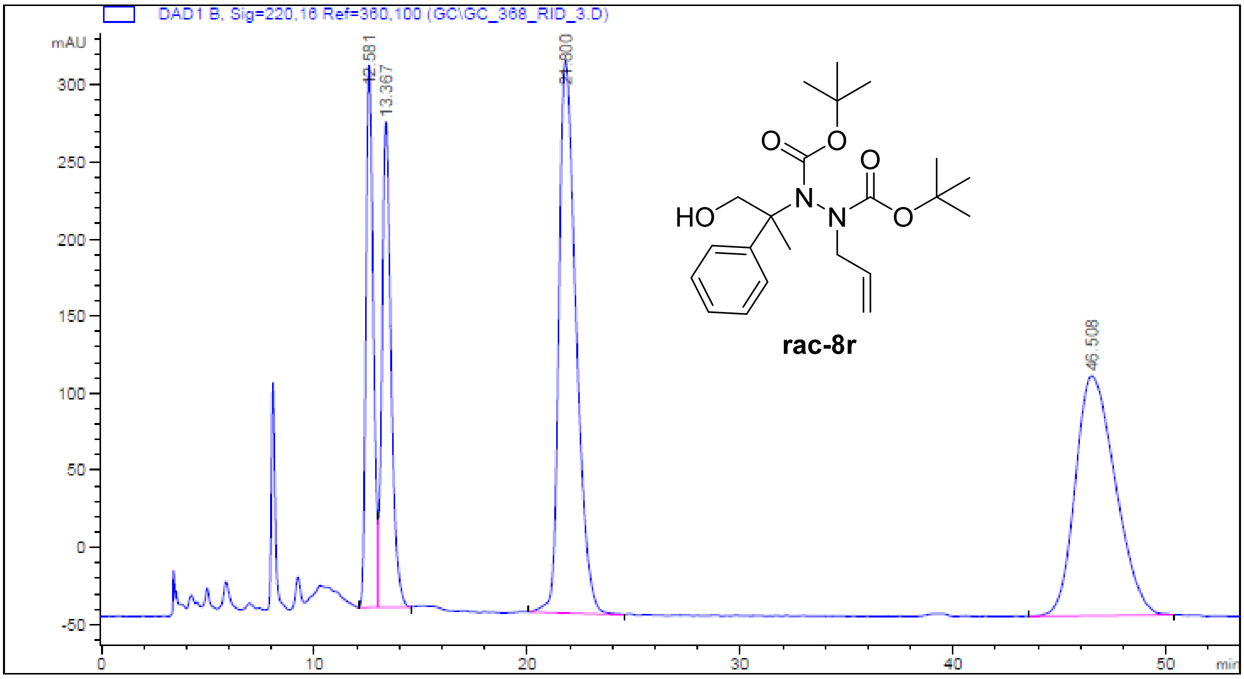

rac-8r

===
Area Percent Report
===

Sorted By : Signal
Multiplier: : 1.0000
Dilution: : 1.0000
Use Multiplier & Dilution Factor with ISTDs

Signal 1: DAD1 B, Sig=220,16 Ref=360,100

Peak #	RetTime [min]	Type	Width [min]	Area [mAU*s]	Height [mAU]	Area %
1	12.581	BV	0.3729	8461.94434	351.49106	14.5415
2	13.367	VB	0.4481	9285.16797	314.79404	15.9561
3	21.800	BV	0.8519	2.01369e4	358.18231	34.6044
4	46.508	BB	1.8148	2.03078e4	155.58533	34.8980

Totals : 5.81918e4 1180.05273

Data File H:\HPLC\1\DATA\GC\GC_417_RID.D
Sample Name: GC_417_rid

===

Acq. Operator : Giovanni
Acq. Instrument : HPLC-1 Location : Vial 1
Injection Date : 04/05/2022 12.55.12
Acq. Method : C:\CHEM32\1\METHODS\DEF_LC.M
Last changed : 04/05/2022 12.50.07 by Giovanni
(modified after loading)
Analysis Method : H:\HPLC\1\METHODS\DEF_LC.M
Last changed : 21/05/2022 14.11.49
(modified after loading)
Sample Info : GC_417_rid, 1 mL/min, 97.5:2.5 hex:ipr, 25°C, IA

Additional Info : Peak(s) manually integrated

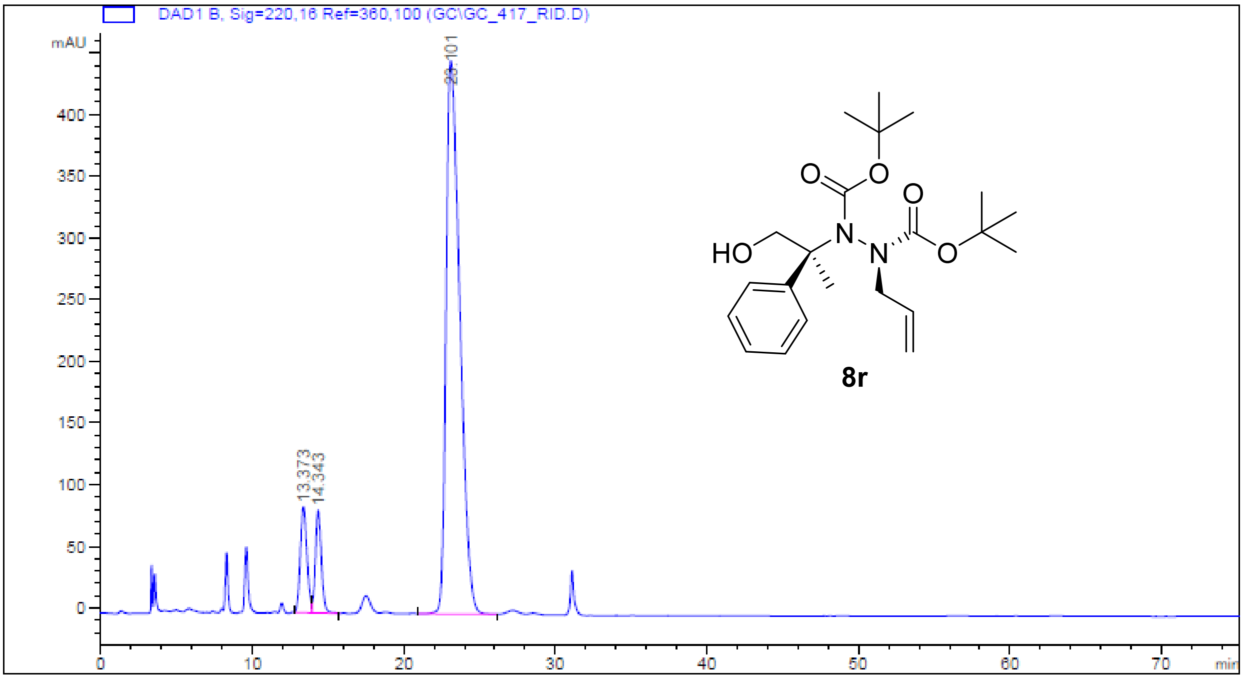

===
Area Percent Report
===

Sorted By : Signal
Multiplier: : 1.0000
Dilution: : 1.0000
Use Multiplier & Dilution Factor with ISTDs

Signal 1: DAD1 B, Sig=220,16 Ref=360,100

Peak #	RetTime [min]	Type	Width [min]	Area [mAU*s]	Height [mAU]	Area %
1	13.373	BV	0.4676	2614.81958	85.78152	7.8636
2	14.343	VB	0.4280	2304.41504	82.99025	6.9301
3	23.101	BB	0.9853	2.83329e4	448.30264	85.2063

Totals : 3.32521e4 617.07441

```
Data File H:\HPLC\1\DATA\GC\GC_355.D
Sample Name: GC_355

=====================================================================
Acq. Operator   : Giovanni
Acq. Instrument : HPLC-1                          Location : Vial 1
Injection Date  : 17/12/2021 15.41.03
Acq. Method     : C:\CHEM32\1\METHODS\DEF_LC.M
Last changed    : 17/12/2021 14.54.51 by Giovanni
                  (modified after loading)
Analysis Method : H:\HPLC\1\METHODS\DEF_LC.M
Last changed    : 07/05/2022 14.10.49
                  (modified after loading)
Sample Info     : GC_355, 1 mL/min, 98:2 hex:ipr, 25°C, IC

Additional Info : Peak(s) manually integrated
```

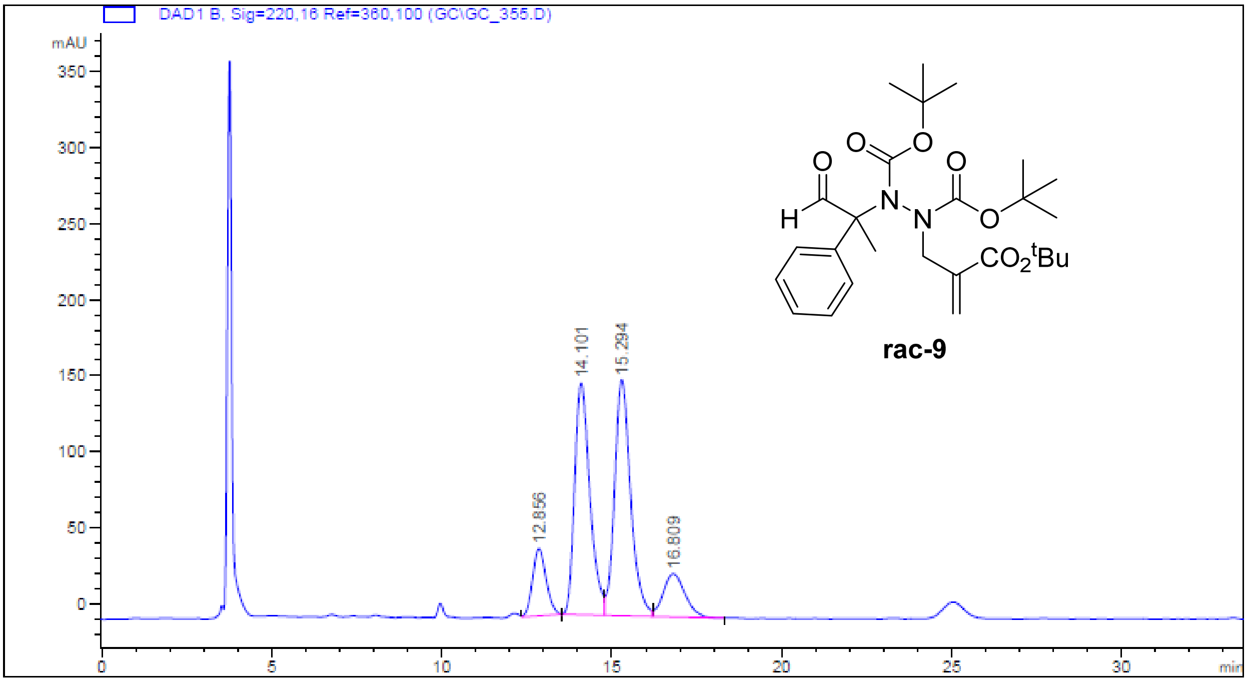


```
=====================================================================
                         Area Percent Report
=====================================================================

Sorted By             :      Signal
Multiplier:                   :      1.0000
Dilution:                     :      1.0000
Use Multiplier & Dilution Factor with ISTDs


Signal 1: DAD1 B, Sig=220,16 Ref=360,100
```

Peak #	RetTime [min]	Type	Width [min]	Area [mAU*s]	Height [mAU]	Area %
1	12.856	VB	0.4324	1228.60205	44.18421	9.9325
2	14.101	BV	0.4758	4765.50781	152.84039	38.5261
3	15.294	VV	0.4998	5114.30322	155.46941	41.3459
4	16.809	VB	0.6908	1261.14856	28.36058	10.1956

Totals : 1.23696e4 380.85459

Data File H:\HPLC\1\DATA\GC\GC_397.D
Sample Name: GC_397

===

Acq. Operator : Giovanni
Acq. Instrument : HPLC-1 Location : Vial 1
Injection Date : 11/03/2022 17.15.19
Acq. Method : C:\CHEM32\1\METHODS\DEF_LC.M
Last changed : 11/03/2022 17.10.50 by Giovanni
(modified after loading)
Analysis Method : H:\HPLC\1\METHODS\DEF_LC.M
Last changed : 07/05/2022 14.10.49
(modified after loading)
Sample Info : GC_397, 1 mL/min, 98:2 hex:ipr, 25°C, IC

Additional Info : Peak(s) manually integrated

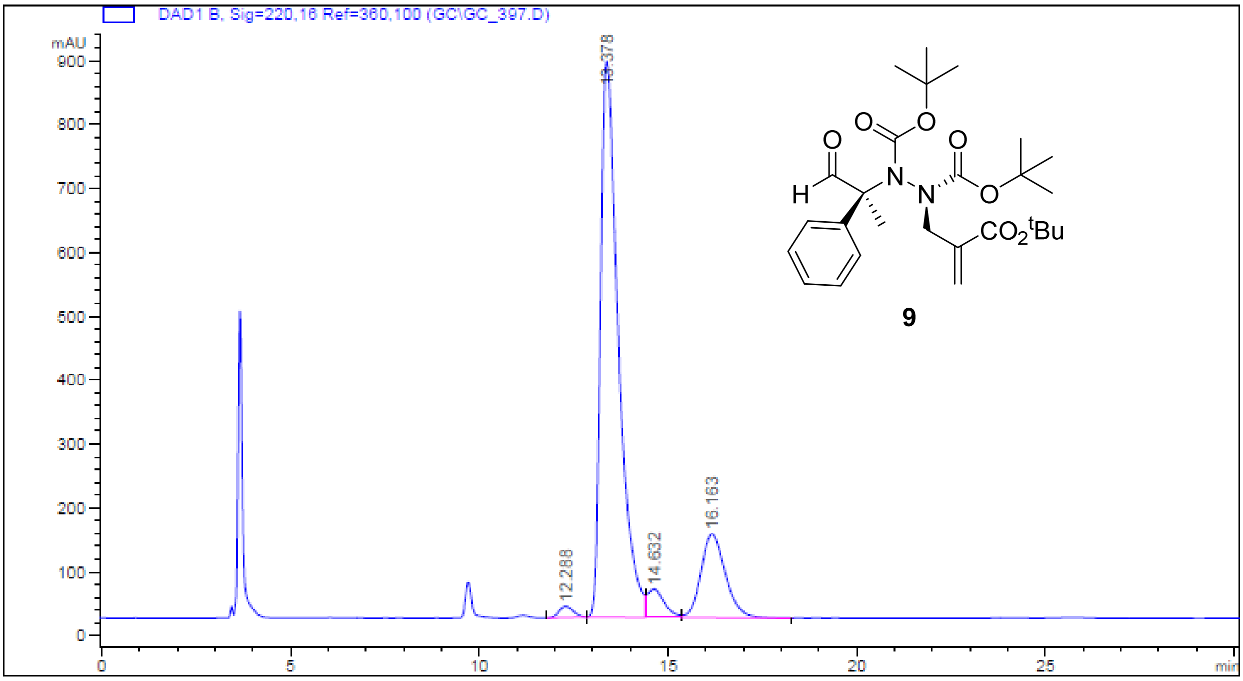

===
Area Percent Report
===

Sorted By : Signal
Multiplier: : 1.0000
Dilution: : 1.0000
Use Multiplier & Dilution Factor with ISTDs

Signal 1: DAD1 B, Sig=220,16 Ref=360,100

Peak #	RetTime [min]	Type	Width [min]	Area [mAU*s]	Height [mAU]	Area %
1	12.288	BB	0.4159	454.48734	17.21891	1.2502
2	13.378	BV	0.4958	2.86133e4	869.67395	78.7122
3	14.632	VV	0.4677	1395.18188	43.79060	3.8380
4	16.163	VB	0.6886	5888.84521	130.95662	16.1996

Totals : 3.63518e4 1061.64008

```
Data File H:\HPLC\1\DATA\CP\CP_242R_3.D
Sample Name: CP_242R_3

=====================================================================
Acq. Operator   : Chiara
Acq. Instrument : HPLC-1                          Location : Vial 1
Injection Date  : 17/05/2022 10:14:46
Acq. Method     : C:\CHEM32\1\METHODS\DEF_LC.M
Last changed    : 17/05/2022 09:44:18 by Chiara
                  (modified after loading)
Analysis Method : H:\HPLC\2\METHODS\DEF_LC.M
Last changed    : 01/06/2022 09:29:28
                  (modified after loading)
Sample Info     : CP_242R_3, pulito con colonna e ridotto, 1 mL/min, 98:2
                   hex:ipr, 25°C, Lux Cellulose

Additional Info : Peak(s) manually integrated
```

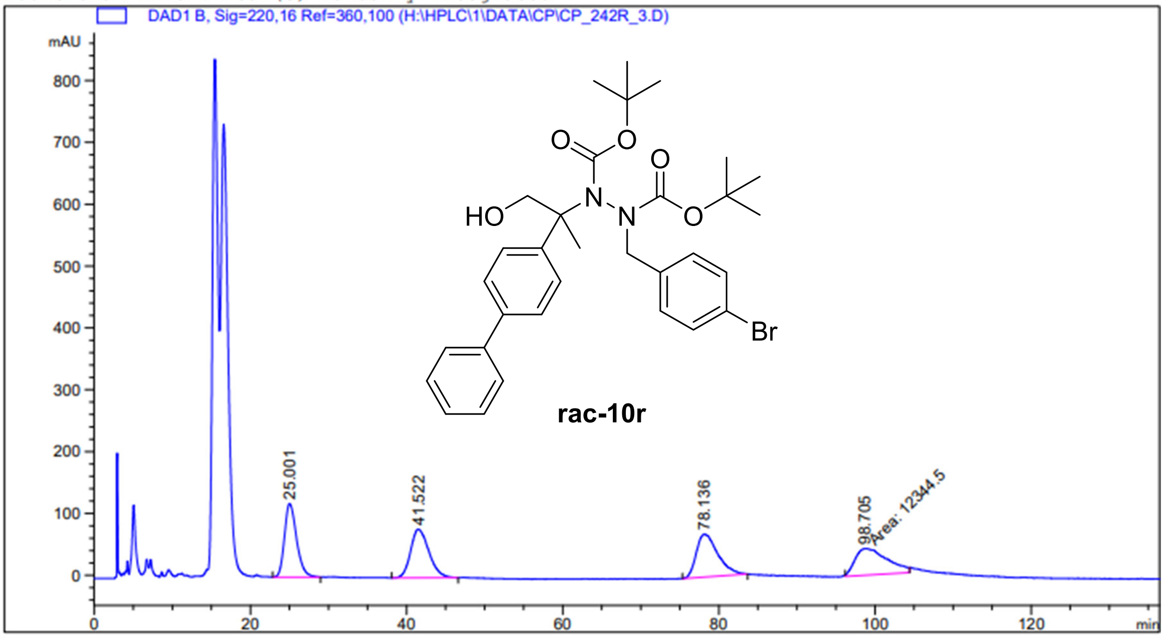


```
=====================================================================
                         Area Percent Report
=====================================================================

Sorted By              :      Signal
Multiplier:                    :       1.0000
Dilution:                      :       1.0000
Use Multiplier & Dilution Factor with ISTDs


Signal 1: DAD1 B, Sig=220,16 Ref=360,100
```

Peak #	RetTime [min]	Type	Width [min]	Area [mAU*s]	Height [mAU]	Area %
1	25.001	BB	1.6558	1.27946e4	118.86250	24.9910
2	41.522	BB	2.1606	1.27472e4	78.33117	24.8982
3	78.136	BB	2.3740	1.33108e4	69.32594	25.9991
4	98.705	MM	4.7758	1.23445e4	43.07943	24.1117
Totals :				5.11970e4	309.59903	

Data File H:\HPLC\1\DATA\CP\CP_236R_3_LUX.D
Sample Name: CP_236R_3

===

Acq. Operator : Chiara
Acq. Instrument : HPLC-1 Location : Vial 1
Injection Date : 17/05/2022 13:10:17
Acq. Method : C:\CHEM32\1\METHODS\DEF_LC.M
Last changed : 17/05/2022 12:31:35 by Chiara
(modified after loading)
Analysis Method : H:\HPLC\2\METHODS\DEF_LC.M
Last changed : 01/06/2022 09:31:13
(modified after loading)
Sample Info : CP_236R_3, 1 mL/min, 98:2 hex:ipr, 25°C, Lux Cellulose

Additional Info : Peak(s) manually integrated

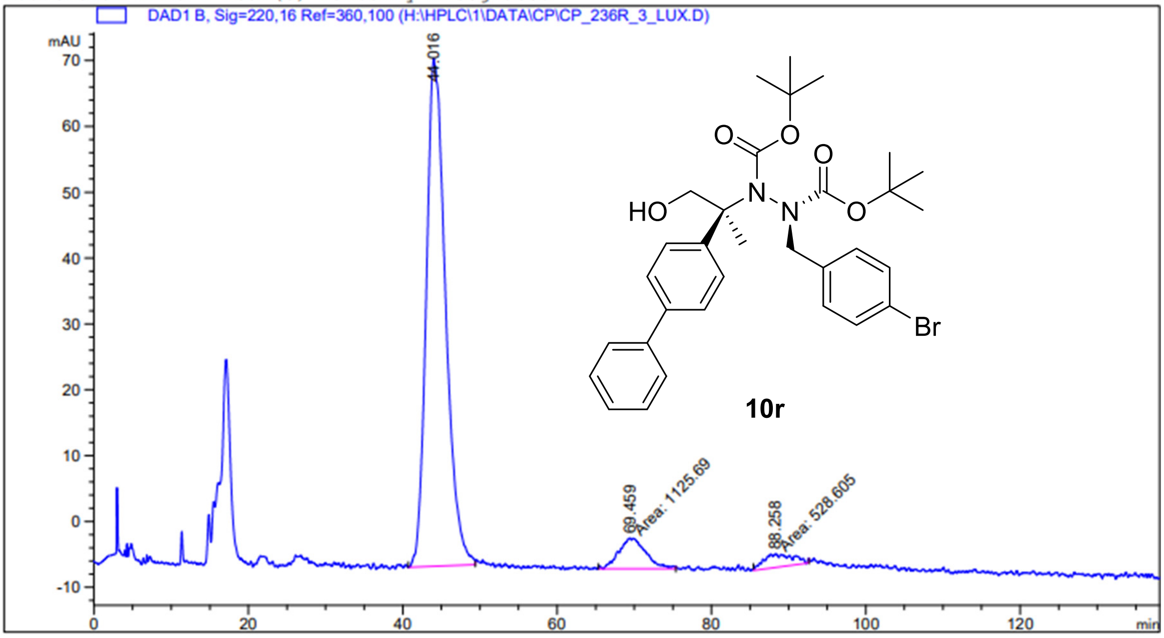

===

Area Percent Report

===

Sorted By : Signal
Multiplier: : 1.0000
Dilution: : 1.0000
Use Multiplier & Dilution Factor with ISTDs

Signal 1: DAD1 B, Sig=220,16 Ref=360,100

Peak #	RetTime [min]	Type	Width [min]	Area [mAU*s]	Height [mAU]	Area %
1	44.016	BV	2.2391	1.33638e4	77.06377	88.9847
2	69.459	MM	4.0484	1125.68591	4.63424	7.4955
3	88.258	MM	4.2324	528.60461	2.08156	3.5198

Totals : 1.50181e4 83.77956

IR traces

5a

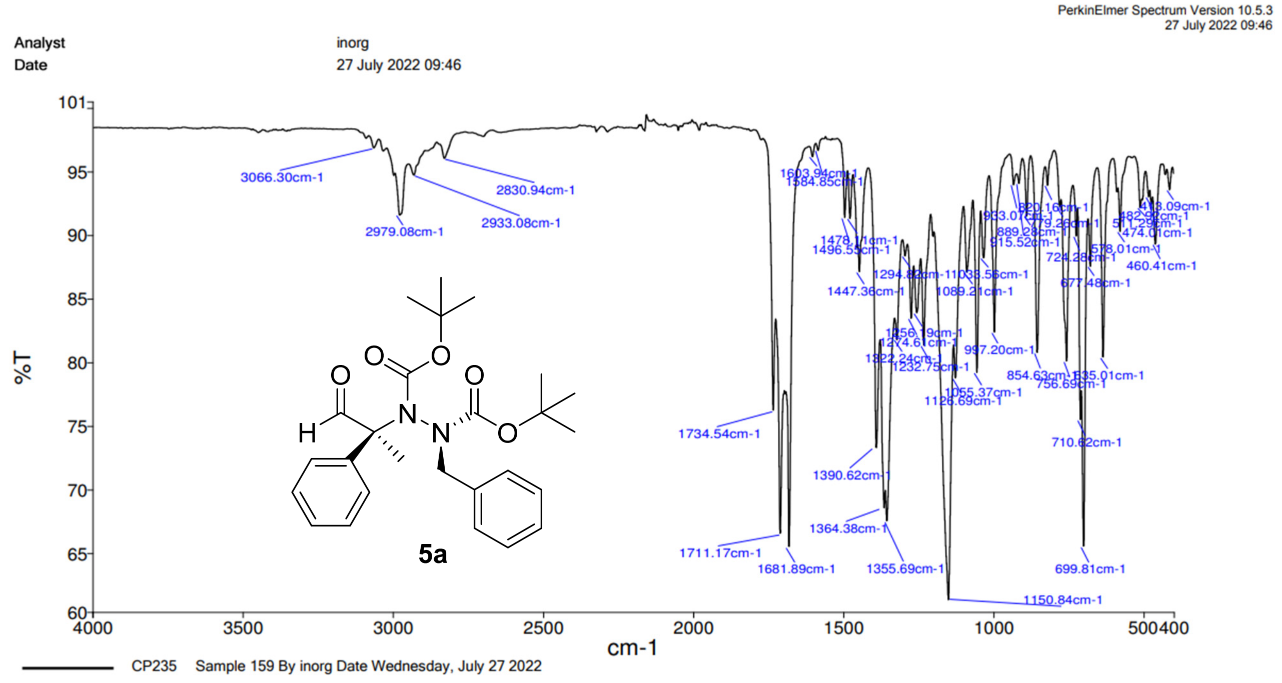
PerkinElmer Spectrum Version 10.5.3
27 July 2022 09:46
Analyst inorg
Date 27 July 2022 09:46
%T
cm-1
1734.54cm-1
1711.17cm-1
1681.89cm-1
1390.62cm-1
1364.38cm-1
1355.69cm-1
1150.84cm-1
699.81cm-1
710.62cm-1
2979.08cm-1
2933.08cm-1
2830.94cm-1
3066.30cm-1
5a
CP235 Sample 159 By inorg Date Wednesday, July 27 2022

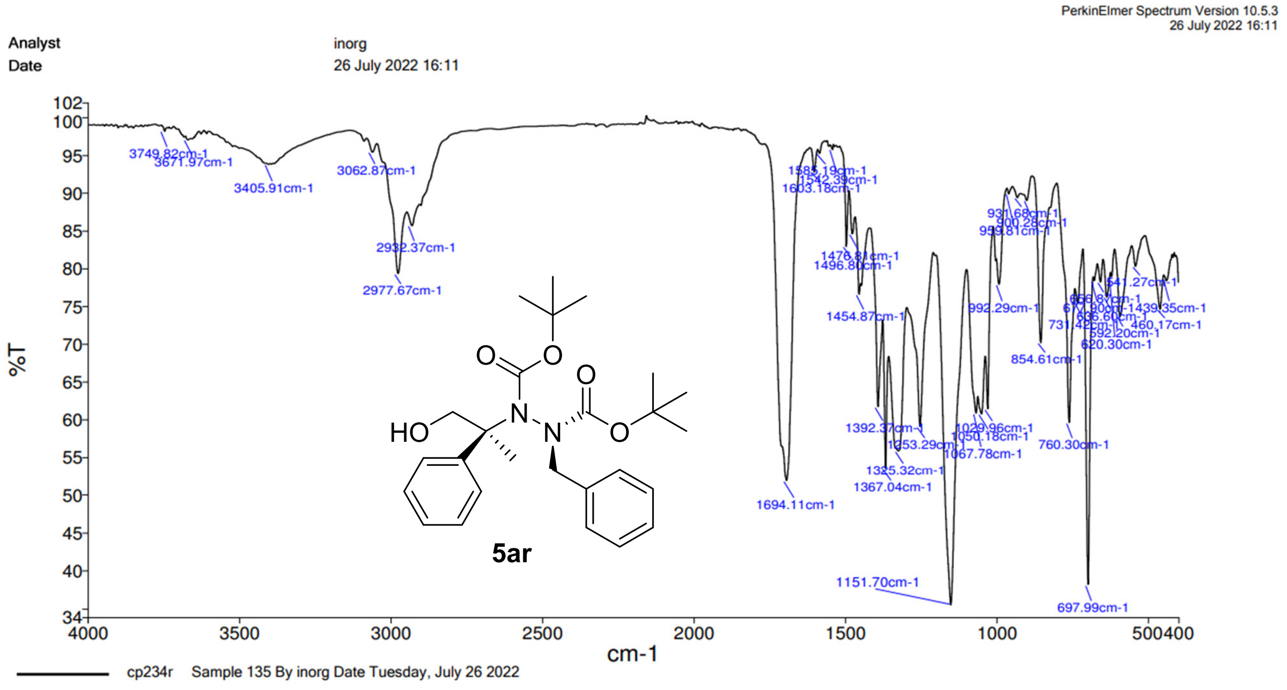
PerkinElmer Spectrum Version 10.5.3
26 July 2022 16:11
Analyst inorg
Date 26 July 2022 16:11
%T
cm-1
3405.91cm-1
2977.67cm-1
1694.11cm-1
1454.87cm-1
1392.37cm-1
1367.04cm-1
1151.70cm-1
992.29cm-1
854.61cm-1
760.30cm-1
697.99cm-1
5ar
cp234r Sample 135 By inorg Date Tuesday, July 26 2022

PerkinElmer Spectrum Version 10.5.3
26 July 2022 17:50

Analyst inorg
Date 26 July 2022 17:50

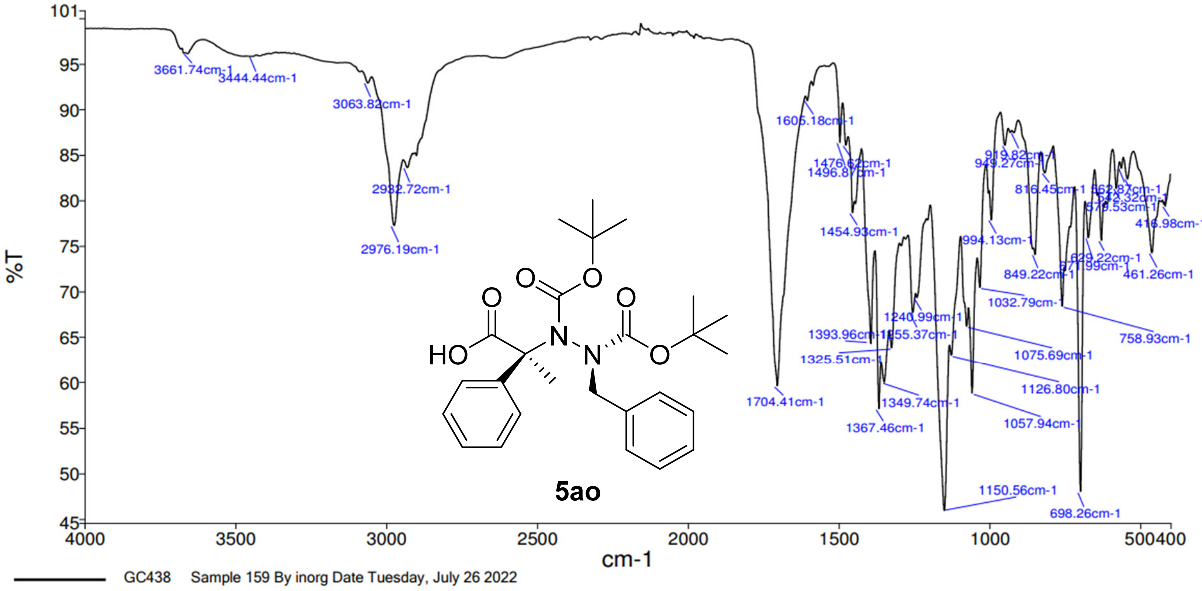

GC438 Sample 159 By inorg Date Tuesday, July 26 2022

PerkinElmer Spectrum Version 10.5.3
26 July 2022 16:30

Analyst inorg
Date 26 July 2022 16:30

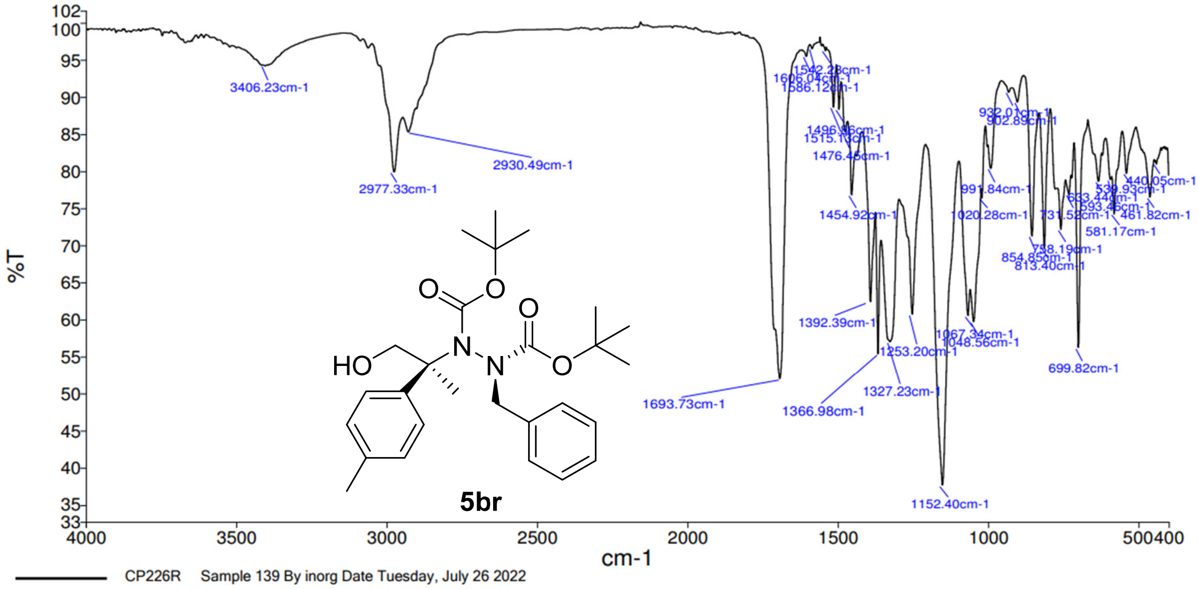

CP226R Sample 139 By inorg Date Tuesday, July 26 2022

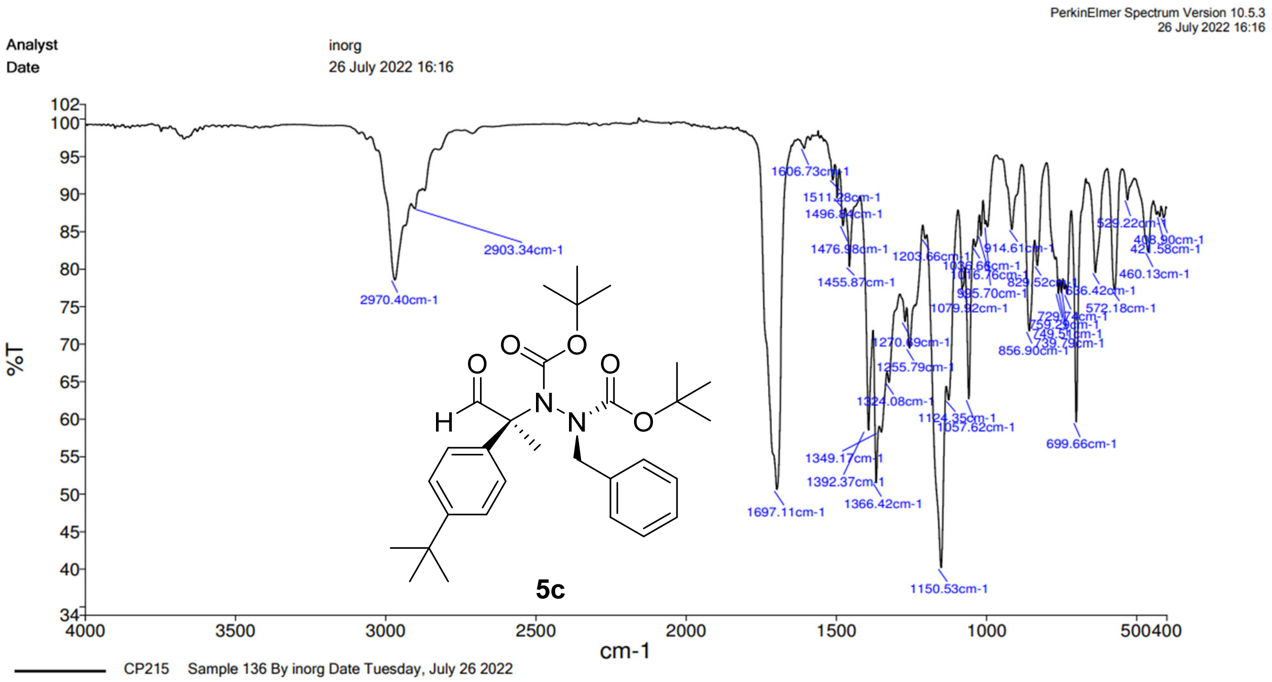
PerkinElmer Spectrum Version 10.5.3
26 July 2022 16:16
Analyst inorg
Date 26 July 2022 16:16
%T
cm-1
2970.40cm-1
2903.34cm-1
1697.11cm-1
1150.53cm-1
699.66cm-1
5c
CP215 Sample 136 By inorg Date Tuesday, July 26 2022

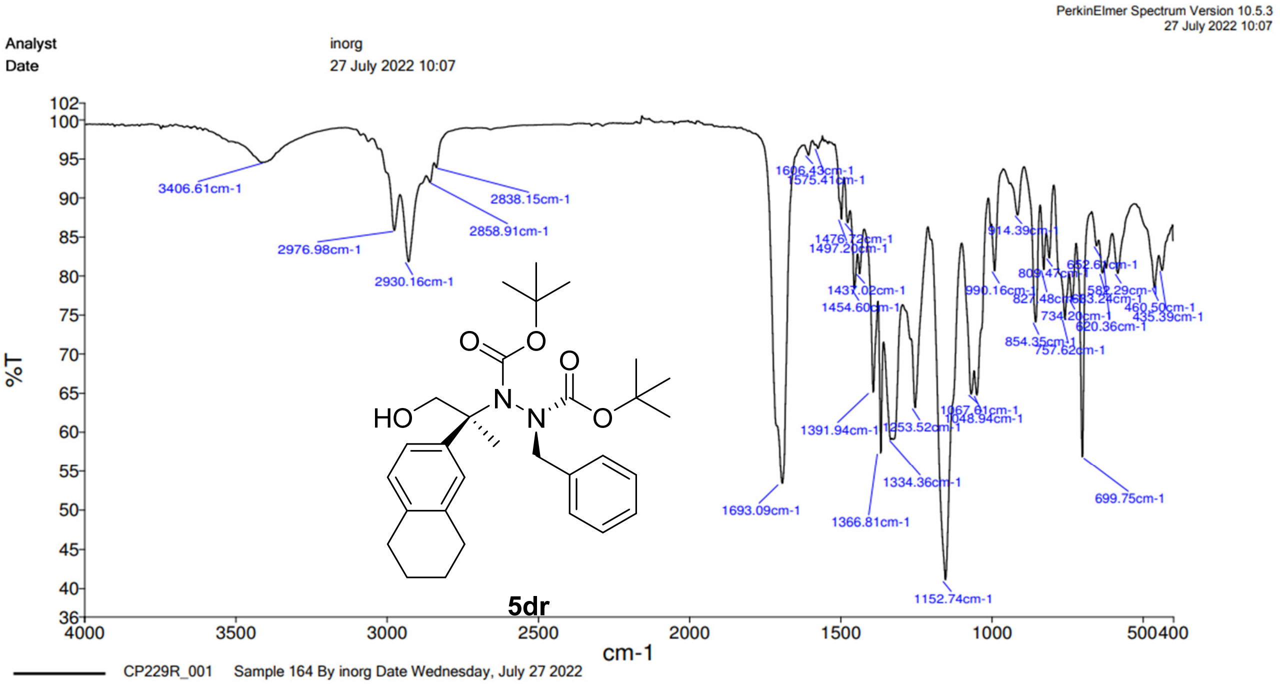
PerkinElmer Spectrum Version 10.5.3
27 July 2022 10:07
Analyst inorg
Date 27 July 2022 10:07
%T
cm-1
3406.61cm-1
2976.98cm-1
2930.16cm-1
2858.91cm-1
2838.15cm-1
1693.09cm-1
1366.81cm-1
1152.74cm-1
699.75cm-1
5dr
CP229R_001 Sample 164 By inorg Date Wednesday, July 27 2022

PerkinElmer Spectrum Version 10.5.3
26 July 2022 16:05

Analyst inorg

Date 26 July 2022 16:05

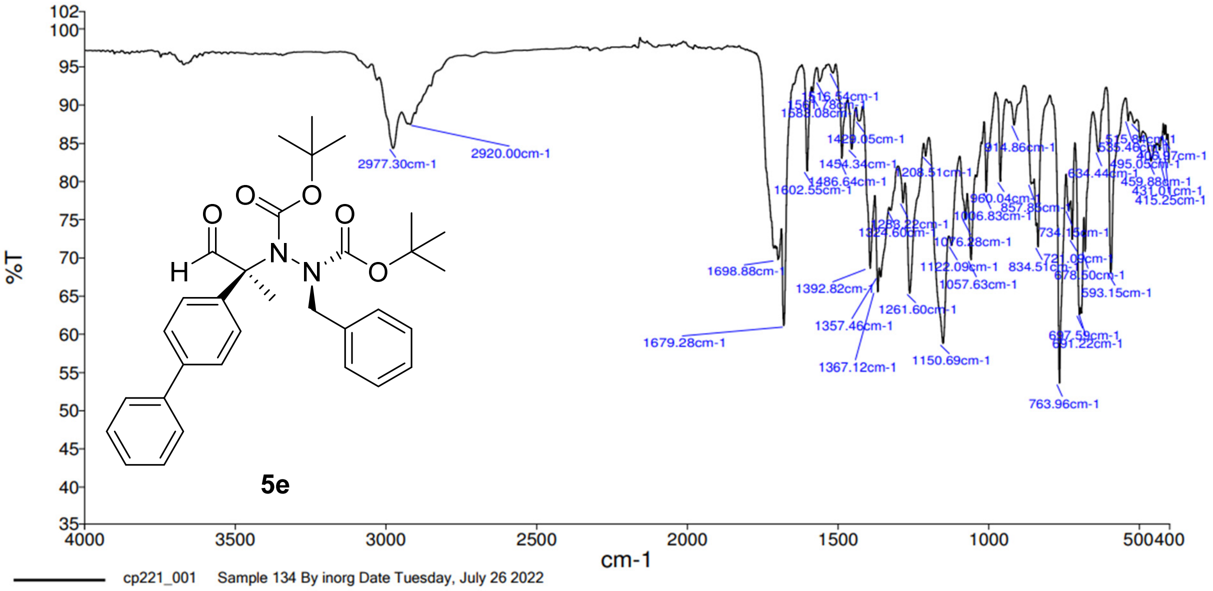

cp221_001 Sample 134 By inorg Date Tuesday, July 26 2022

PerkinElmer Spectrum Version 10.5.3
27 July 2022 10:03

Analyst inorg

Date 27 July 2022 10:03

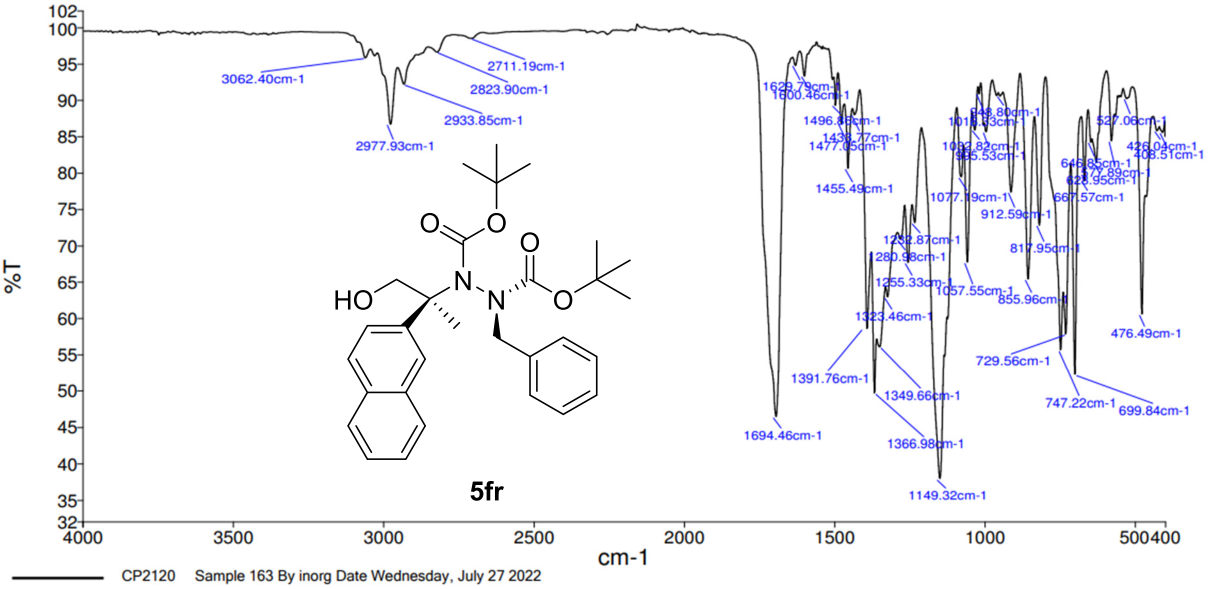

CP2120 Sample 163 By inorg Date Wednesday, July 27 2022

PerkinElmer Spectrum Version 10.5.3
26 July 2022 16:46

Analyst inorg
Date 26 July 2022 16:46

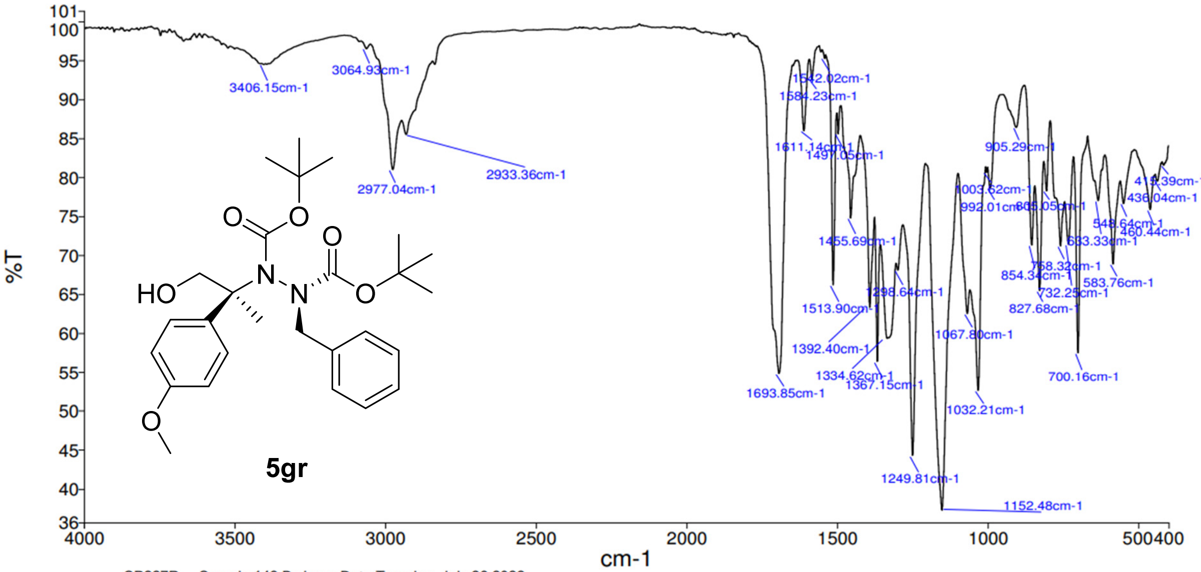

CP227R Sample 143 By inorg Date Tuesday, July 26 2022

PerkinElmer Spectrum Version 10.5.3
26 July 2022 16:34

Analyst inorg
Date 26 July 2022 16:34

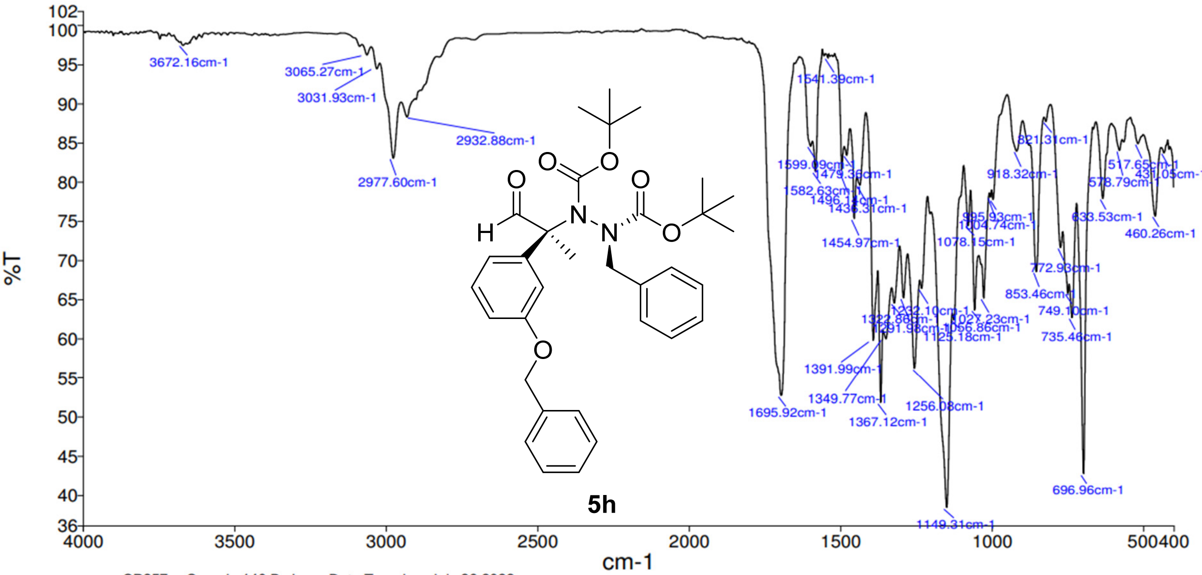

CP257 Sample 140 By inorg Date Tuesday, July 26 2022

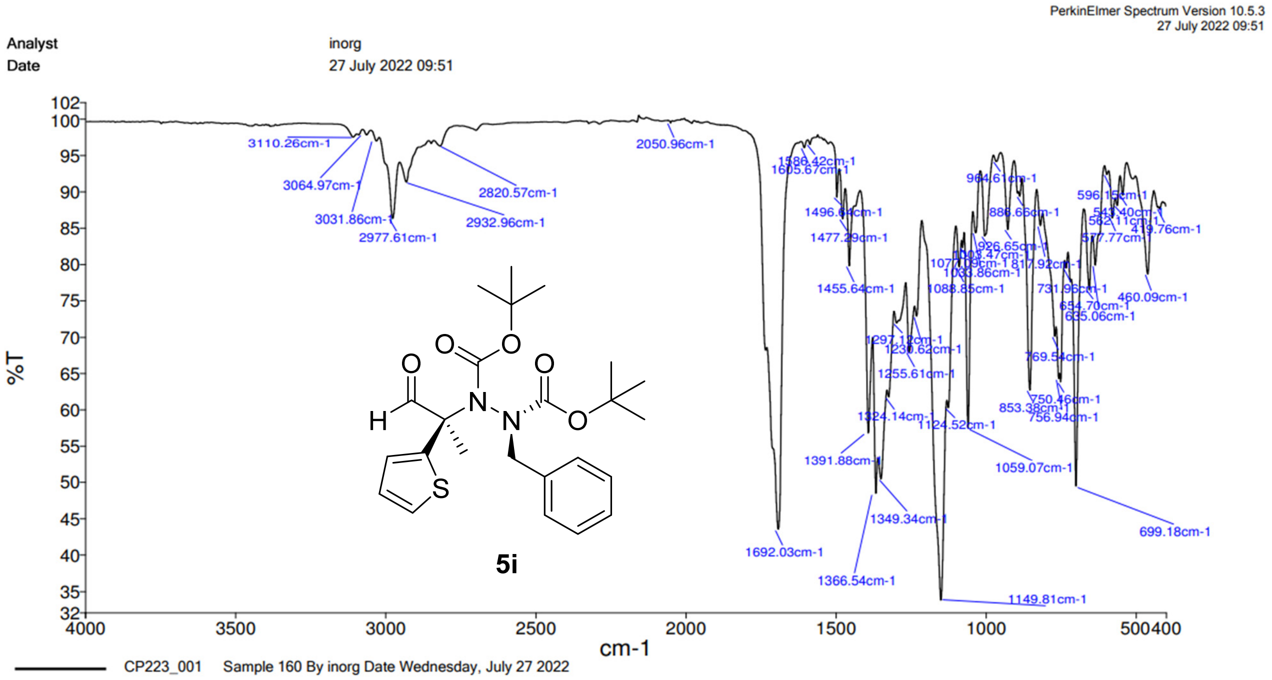
PerkinElmer Spectrum Version 10.5.3
27 July 2022 09:51
Analyst inorg
Date 27 July 2022 09:51
%T
cm-1
3110.26cm-1
3064.97cm-1
3031.86cm-1
2977.61cm-1
2932.96cm-1
2820.57cm-1
2050.96cm-1
1692.03cm-1
1477.29cm-1
1455.64cm-1
1391.88cm-1
1366.54cm-1
1349.34cm-1
1255.61cm-1
1149.81cm-1
1124.52cm-1
1088.85cm-1
1059.07cm-1
769.54cm-1
756.94cm-1
699.18cm-1
460.09cm-1
5i
CP223_001 Sample 160 By inorg Date Wednesday, July 27 2022

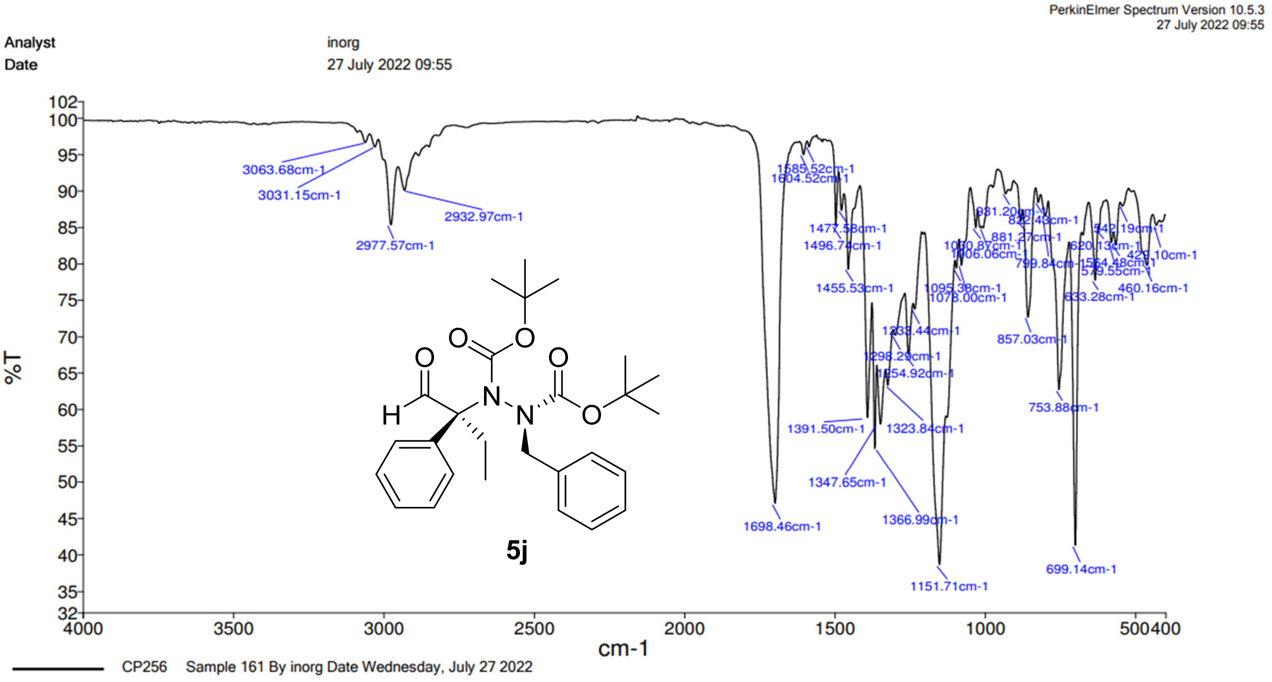
PerkinElmer Spectrum Version 10.5.3
27 July 2022 09:55
Analyst inorg
Date 27 July 2022 09:55
%T
cm-1
3063.68cm-1
3031.15cm-1
2977.57cm-1
2932.97cm-1
1698.46cm-1
1496.74cm-1
1455.53cm-1
1391.50cm-1
1347.65cm-1
1366.99cm-1
1323.84cm-1
1151.71cm-1
857.03cm-1
753.88cm-1
699.14cm-1
460.16cm-1
5j
CP256 Sample 161 By inorg Date Wednesday, July 27 2022

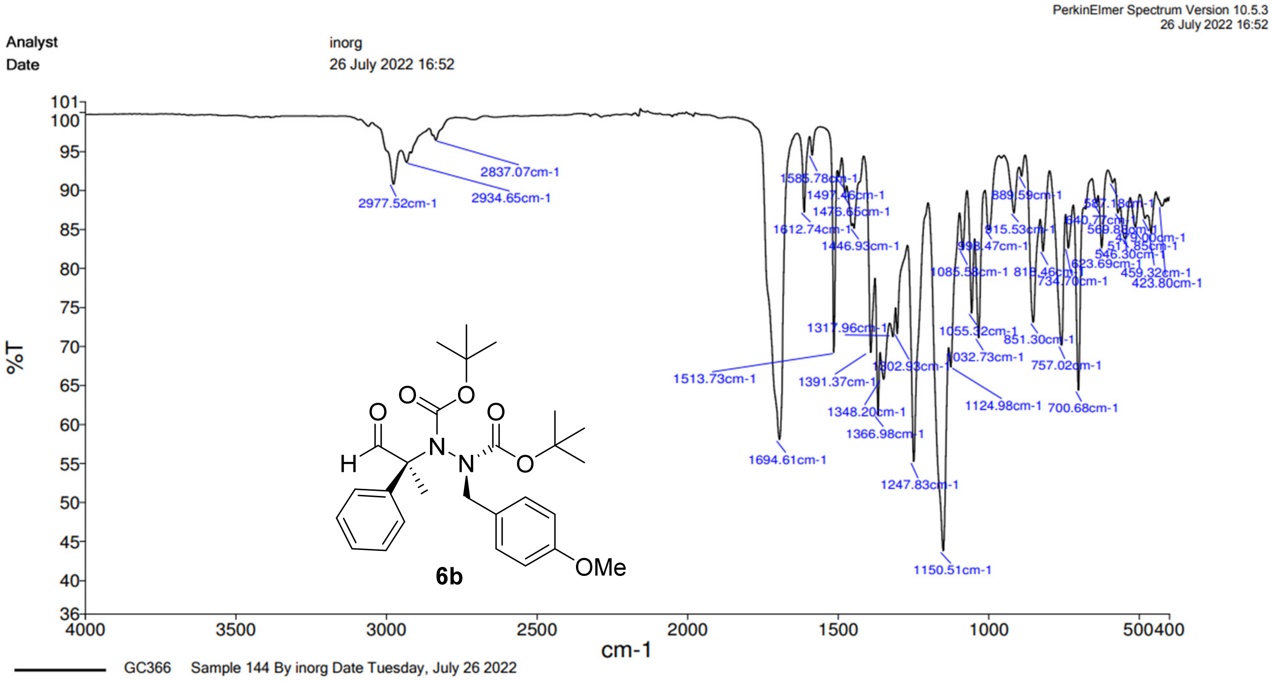
PerkinElmer Spectrum Version 10.5.3
26 July 2022 16:52
Analyst inorg
Date 26 July 2022 16:52
%T
cm-1
2977.52cm-1
2934.65cm-1
2837.07cm-1
1694.61cm-1
1513.73cm-1
1391.37cm-1
1348.20cm-1
1366.98cm-1
1247.83cm-1
1150.51cm-1
1124.98cm-1
1032.73cm-1
851.30cm-1
757.02cm-1
700.68cm-1
6b
OMe
GC366 Sample 144 By inorg Date Tuesday, July 26 2022

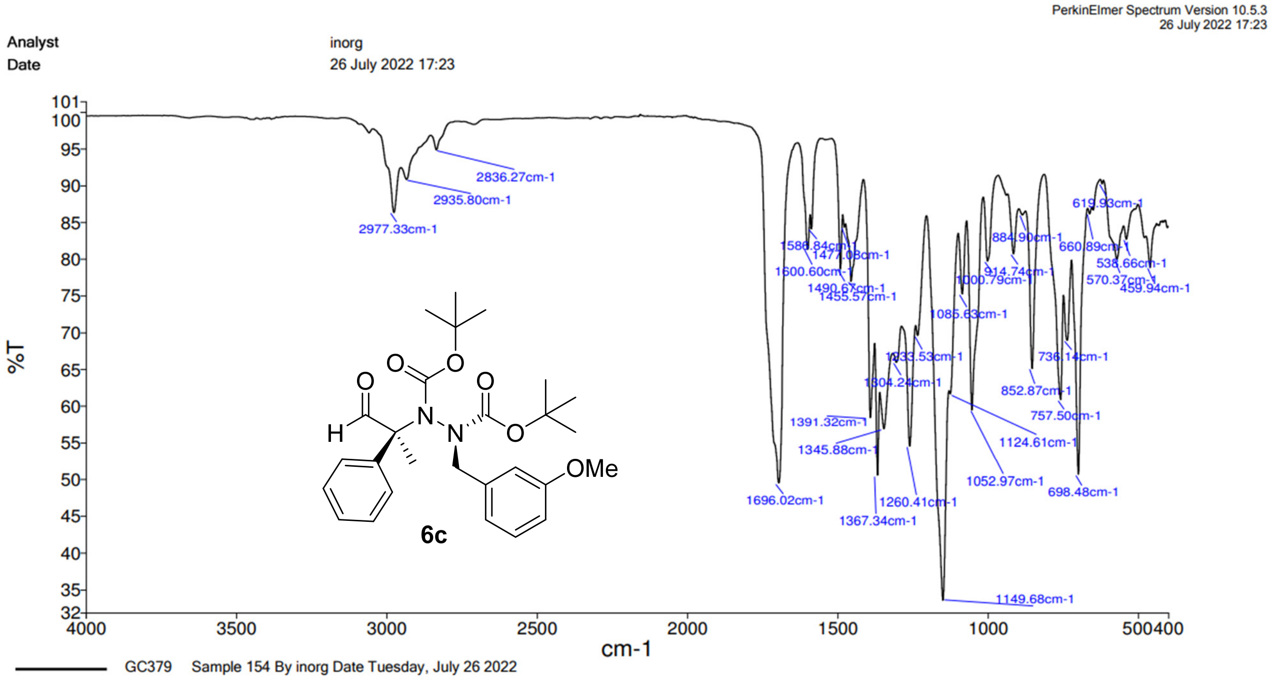
PerkinElmer Spectrum Version 10.5.3
26 July 2022 17:23
Analyst inorg
Date 26 July 2022 17:23
%T
cm-1
2977.33cm-1
2935.80cm-1
2836.27cm-1
1696.02cm-1
1391.32cm-1
1345.88cm-1
1367.34cm-1
1260.41cm-1
1149.68cm-1
1124.61cm-1
1052.97cm-1
852.87cm-1
757.50cm-1
698.48cm-1
6c
OMe
GC379 Sample 154 By inorg Date Tuesday, July 26 2022

PerkinElmer Spectrum Version 10.5.3
26 July 2022 17:08

Analyst inorg
Date 26 July 2022 17:08

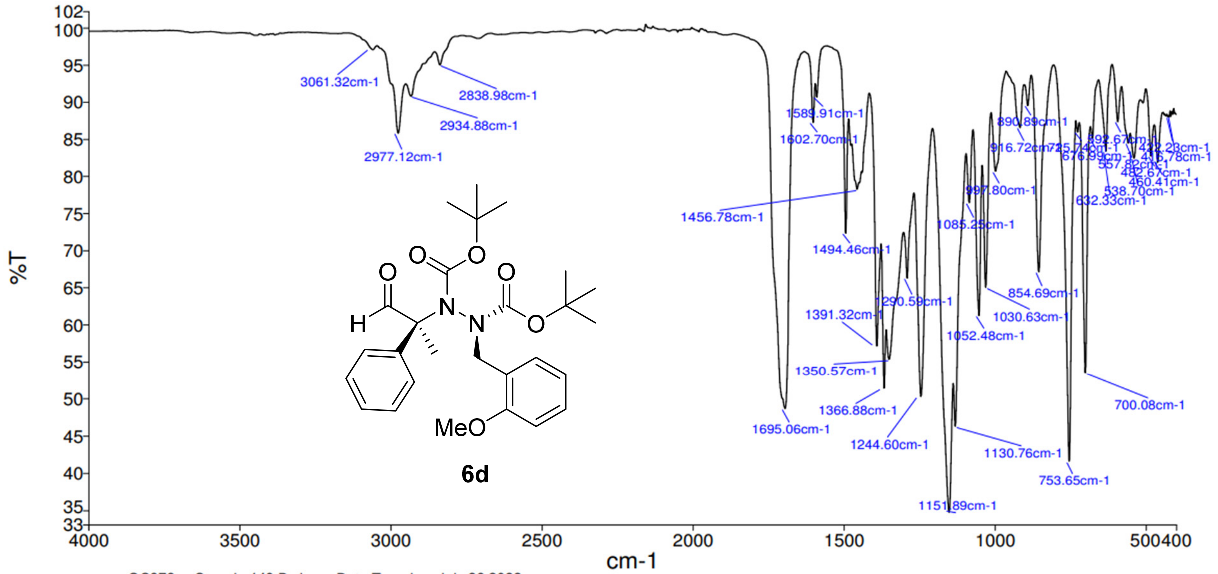

GC378 Sample 149 By inorg Date Tuesday, July 26 2022

PerkinElmer Spectrum Version 10.5.3
26 July 2022 16:56

Analyst inorg
Date 26 July 2022 16:56

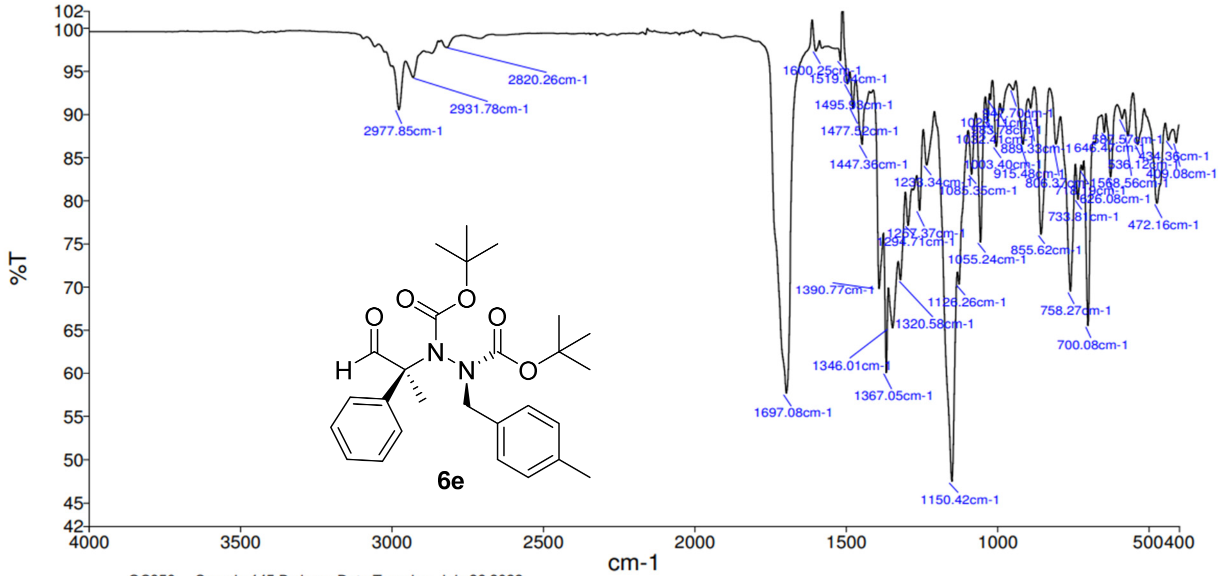

GC350 Sample 145 By inorg Date Tuesday, July 26 2022

PerkinElmer Spectrum Version 10.5.3
26 July 2022 17:19

Analyst inorg
Date 26 July 2022 17:19

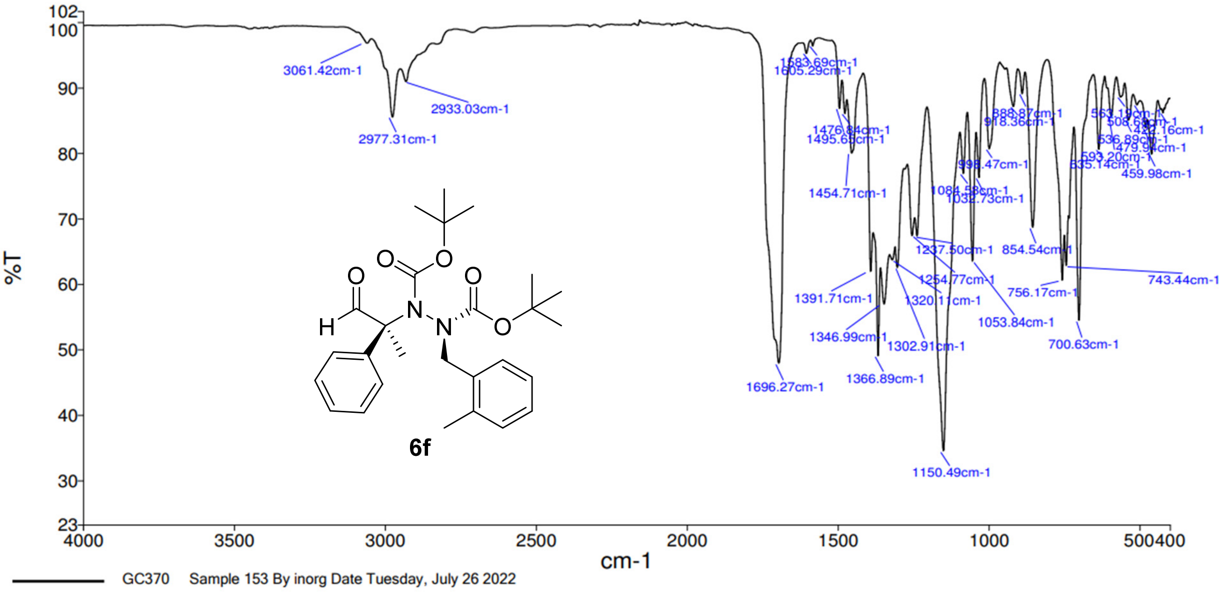

GC370 Sample 153 By inorg Date Tuesday, July 26 2022

PerkinElmer Spectrum Version 10.5.3
26 July 2022 17:00

Analyst inorg
Date 26 July 2022 17:00

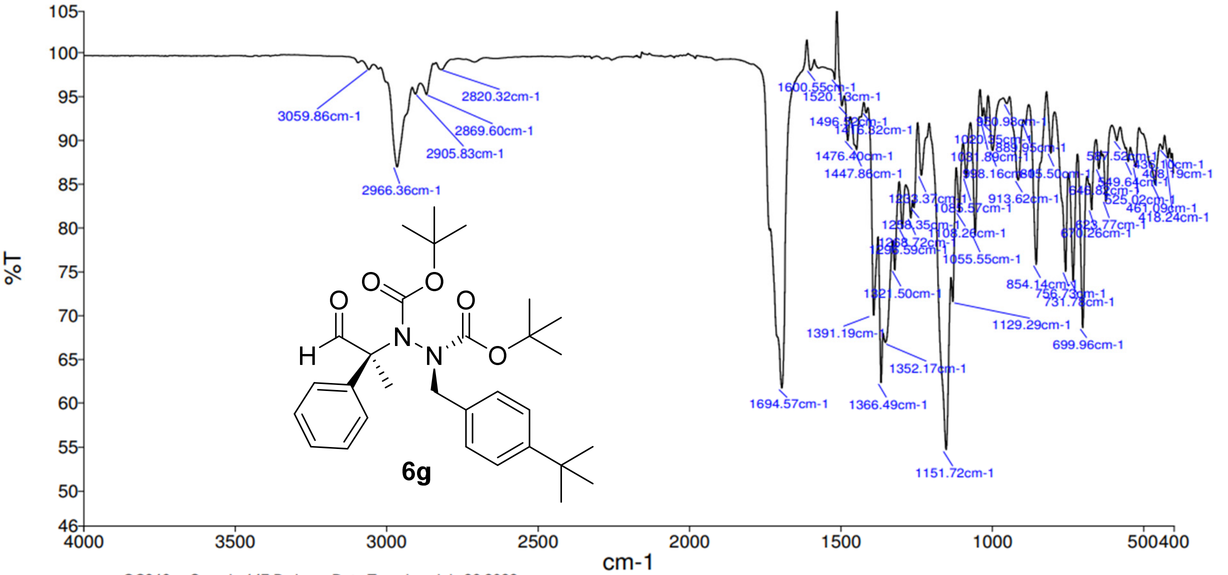

GC346 Sample 147 By inorg Date Tuesday, July 26 2022

PerkinElmer Spectrum Version 10.5.3
26 July 2022 17:10

Analyst inorg
Date 26 July 2022 17:10

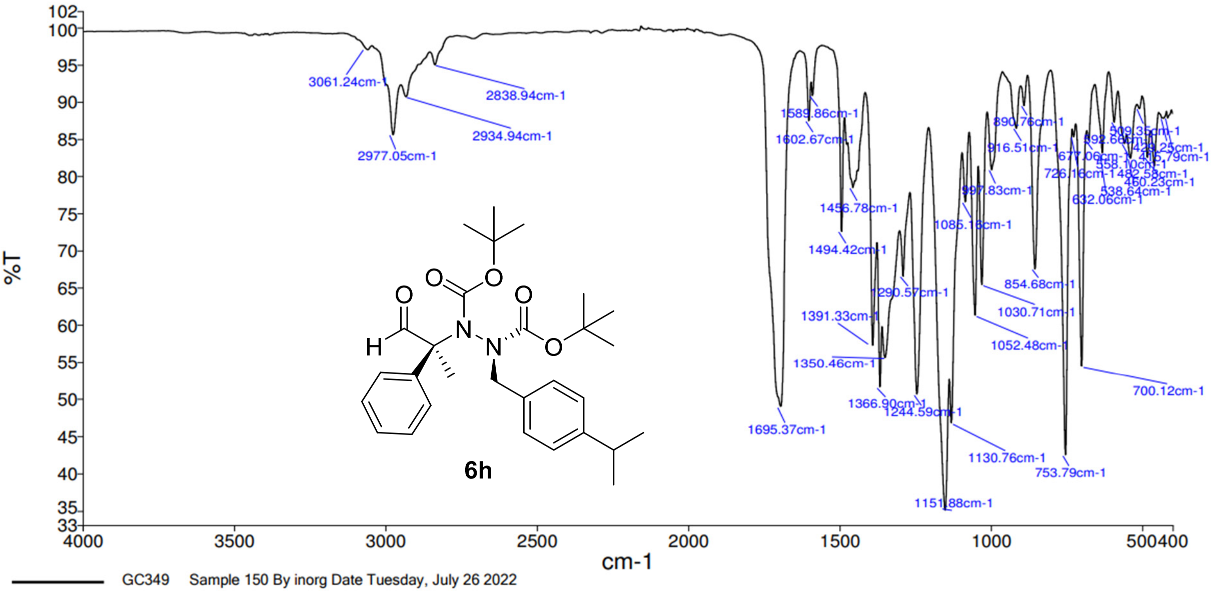

GC349 Sample 150 By inorg Date Tuesday, July 26 2022

PerkinElmer Spectrum Version 10.5.3
26 July 2022 17:13

Analyst inorg
Date 26 July 2022 17:13

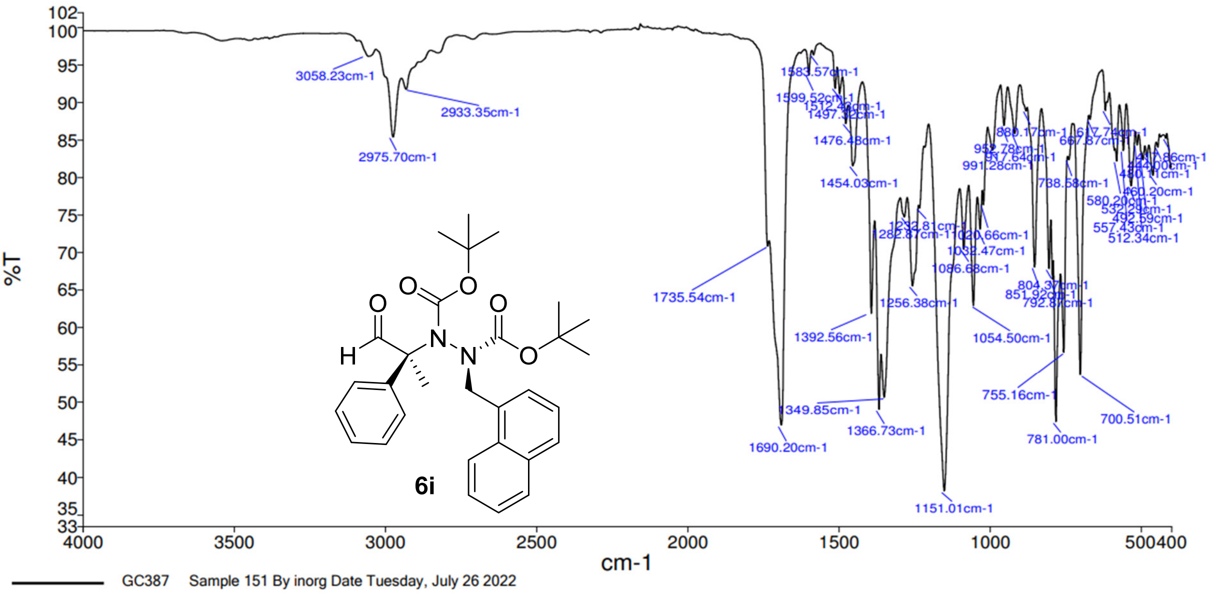

GC387 Sample 151 By inorg Date Tuesday, July 26 2022

PerkinElmer Spectrum Version 10.5.3
26 July 2022 17:34

Analyst inorg
Date 26 July 2022 17:34

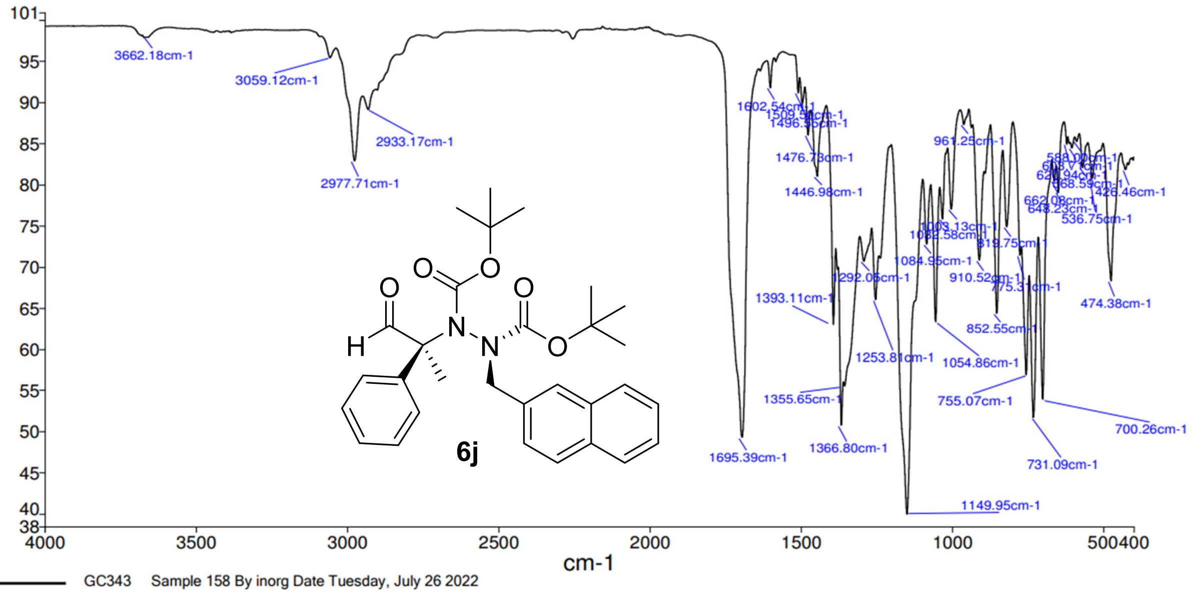

GC343 Sample 158 By inorg Date Tuesday, July 26 2022

PerkinElmer Spectrum Version 10.5.3
26 July 2022 17:31

Analyst inorg
Date 26 July 2022 17:31

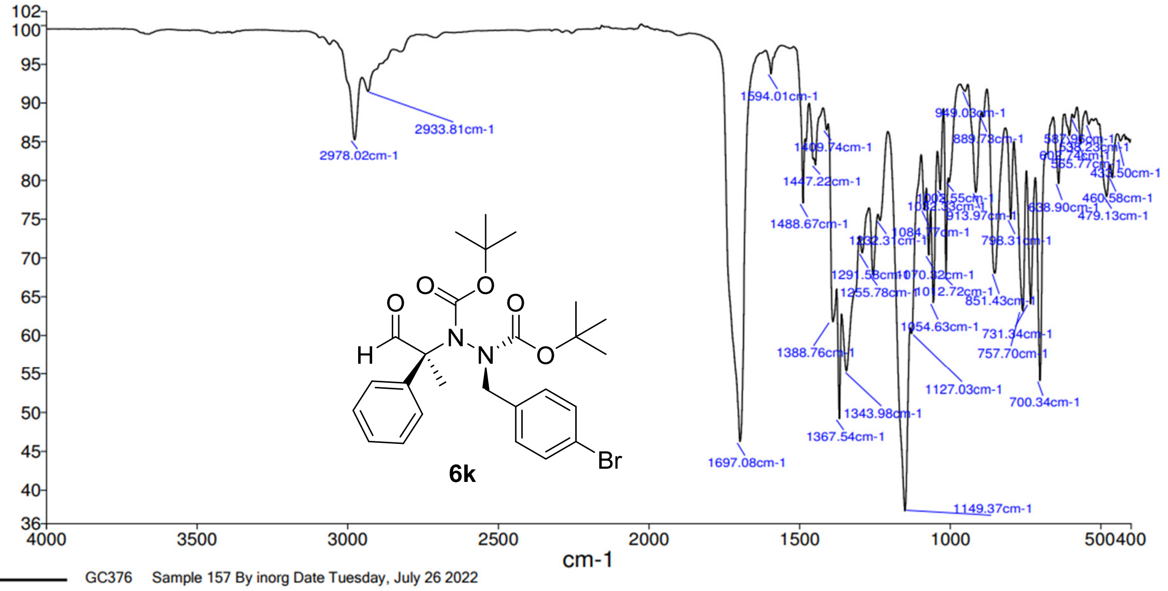

GC376 Sample 157 By inorg Date Tuesday, July 26 2022

PerkinElmer Spectrum Version 10.5.3
26 July 2022 17:25

Analyst inorg
Date 26 July 2022 17:25

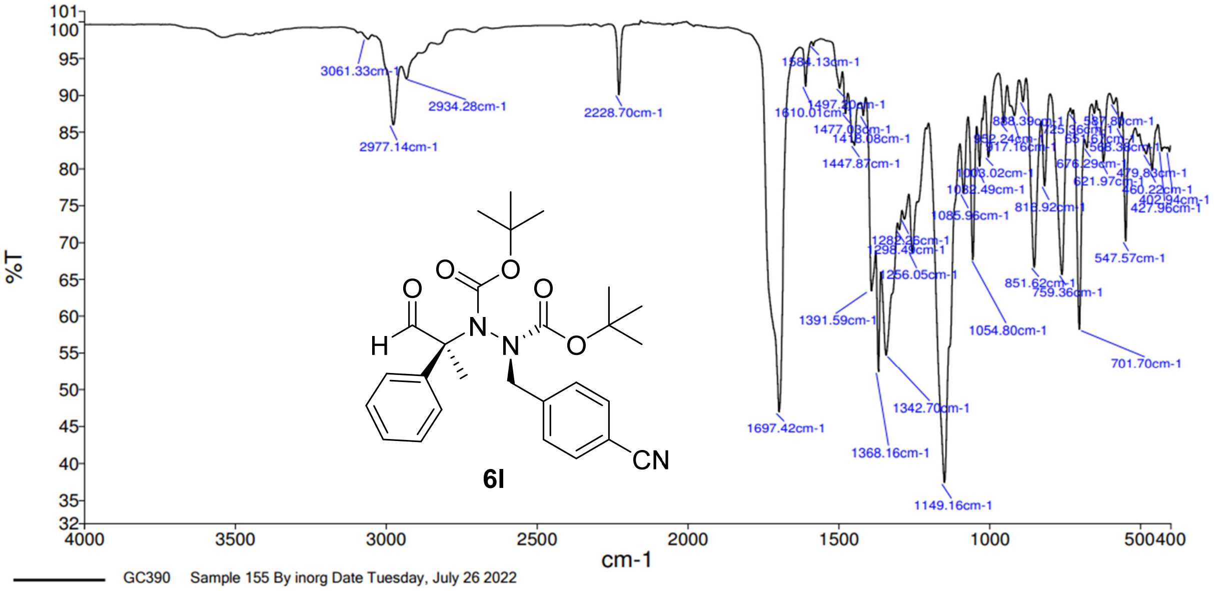

GC390 Sample 155 By inorg Date Tuesday, July 26 2022

PerkinElmer Spectrum Version 10.5.3
26 July 2022 17:28

Analyst inorg
Date 26 July 2022 17:28

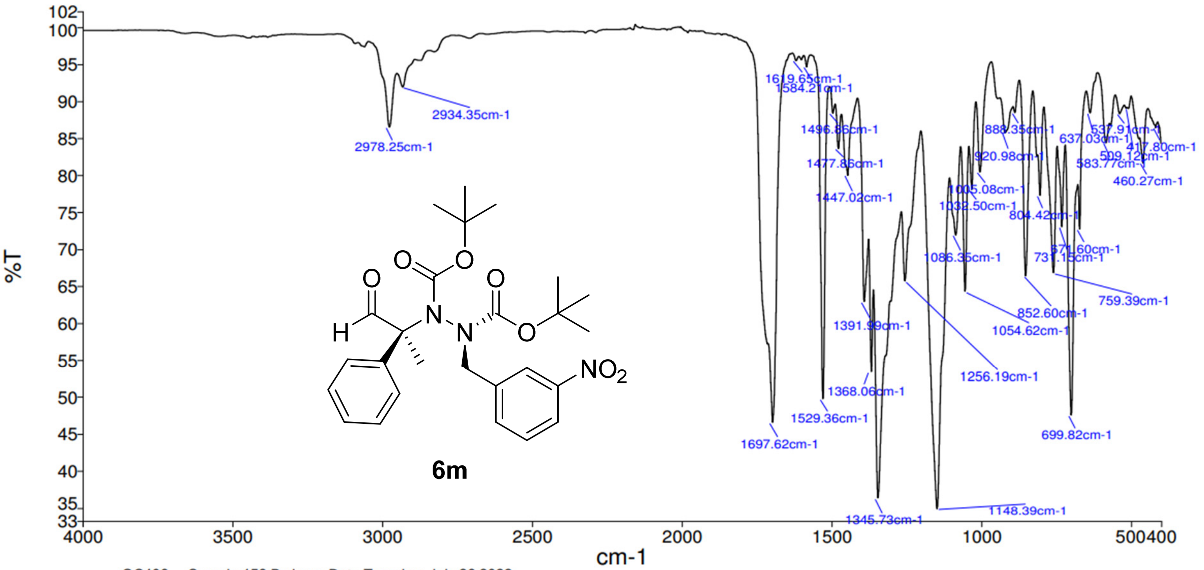

GC406 Sample 156 By inorg Date Tuesday, July 26 2022

PerkinElmer Spectrum Version 10.5.3
27 July 2022 09:59

Analyst inorg
Date 27 July 2022 09:59

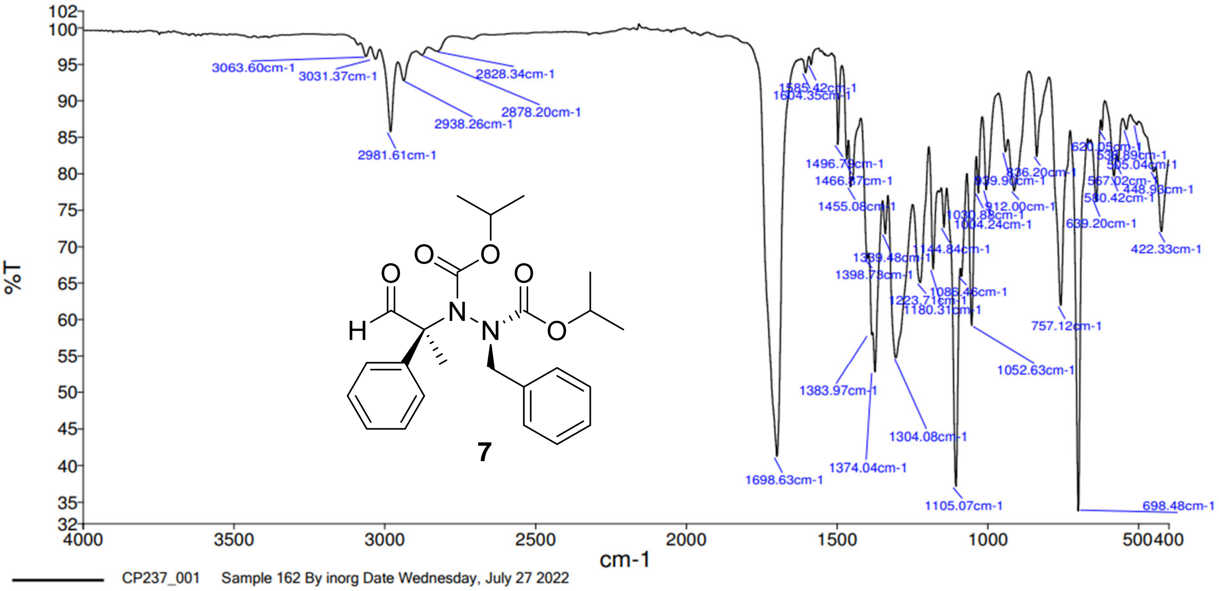

CP237_001 Sample 162 By inorg Date Wednesday, July 27 2022

PerkinElmer Spectrum Version 10.5.3
26 July 2022 17:04

Analyst inorg
Date 26 July 2022 17:04

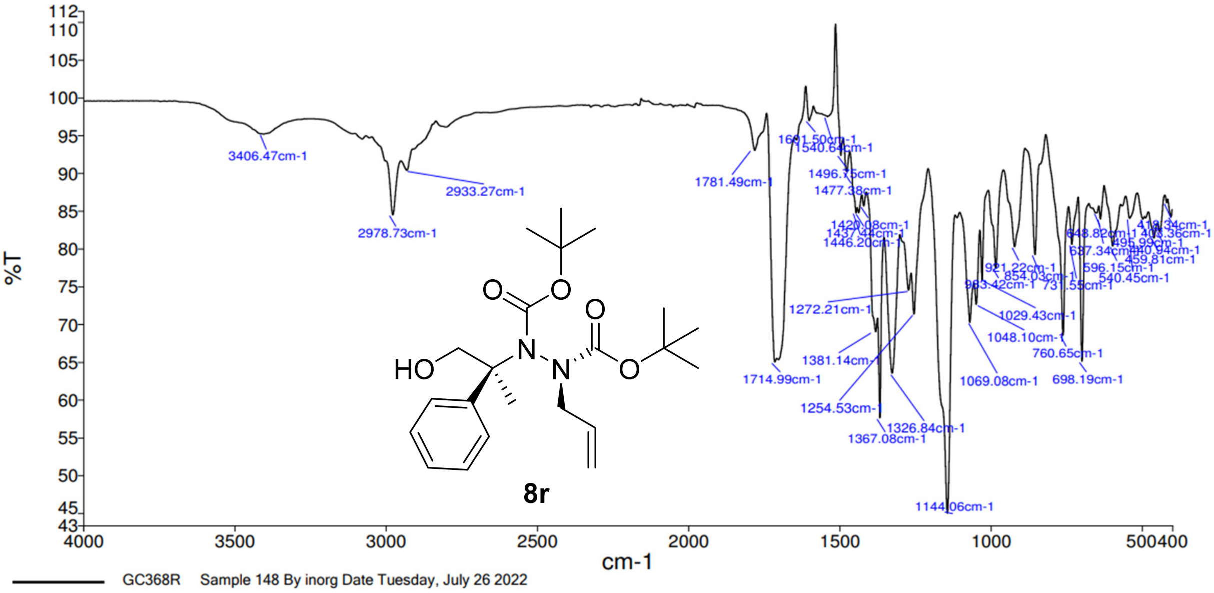

GC368R Sample 148 By inorg Date Tuesday, July 26 2022

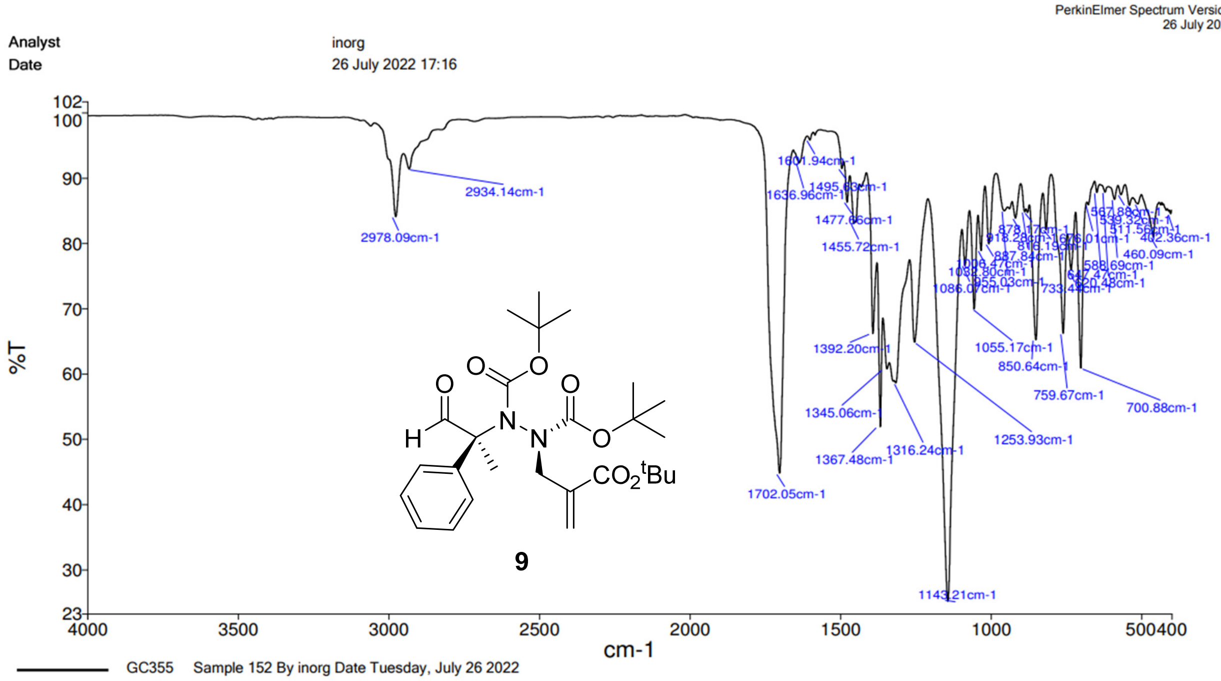
PerkinElmer Spectrum Version 10.5.3
26 July 2022 17:16
Analyst inorg
Date 26 July 2022 17:16
%T
cm-1
2978.09cm-1
2934.14cm-1
1702.05cm-1
1143.21cm-1
9
GC355 Sample 152 By inorg Date Tuesday, July 26 2022

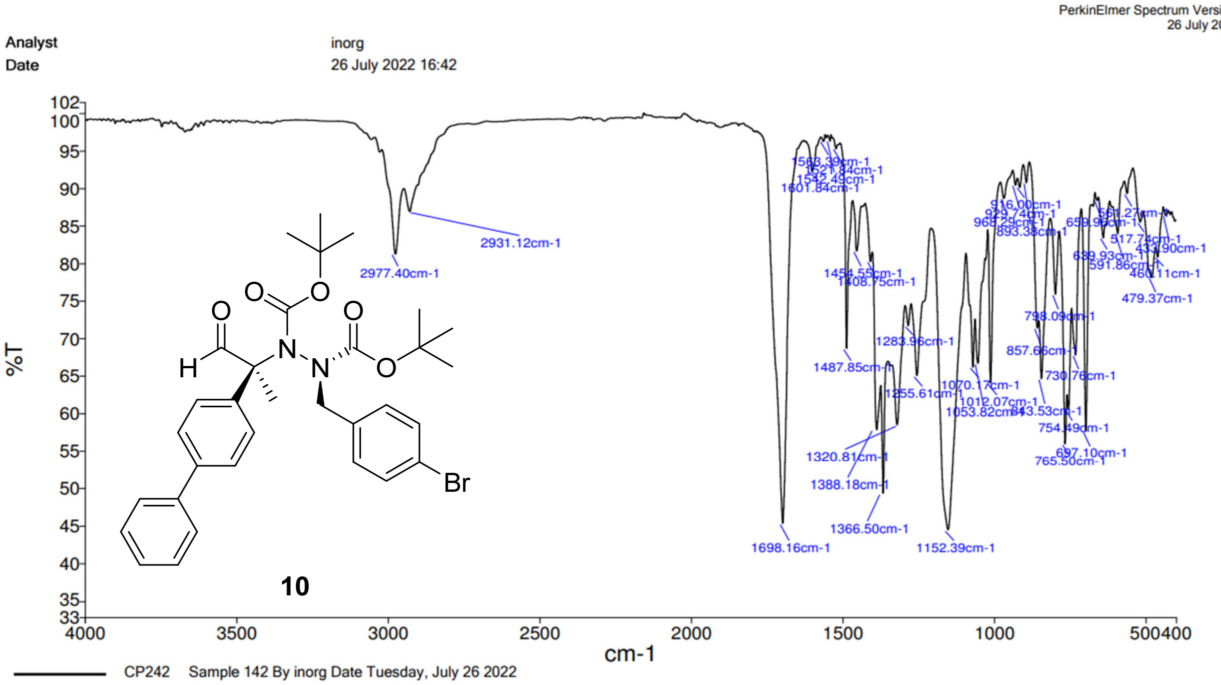
PerkinElmer Spectrum Version 10.5.3
26 July 2022 16:42
Analyst inorg
Date 26 July 2022 16:42
%T
cm-1
2977.40cm-1
2931.12cm-1
1698.16cm-1
1152.39cm-1
Br
10
CP242 Sample 142 By inorg Date Tuesday, July 26 2022
